# Main Group-Catalyzed Cationic Claisen Rearrangements via Vinyl Carbocations

**DOI:** 10.1021/acs.orglett.4c00837

**Published:** 2024-06-06

**Authors:** Chloe G. Williams, Sepand K. Nistanaki, Krista Dong, Woojin Lee, Kendall N. Houk, Hosea M. Nelson

**Affiliations:** † Division of Chemistry and Chemical Engineering, California Institute of Technology, Pasadena, California 91125, United States; ‡ Department of Chemistry and Biochemistry, University of California, Los Angeles, Los Angeles, California 90095, United States

## Abstract

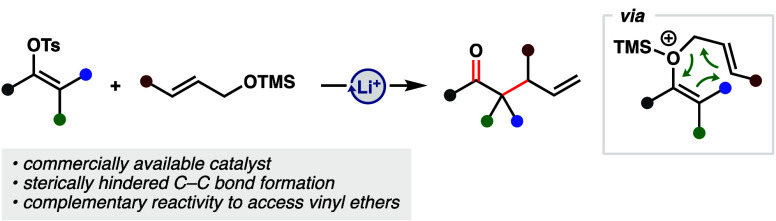

We report a catalytic C–O coupling/Claisen cascade reaction enabled by interception of vinyl carbocations with allyl ethers. The reaction utilizes commercially available borate salts as catalysts and is effective at constructing sterically hindered C–C bonds. The reaction mechanism is studied experimentally and computationally to support a charge-accelerated [3,3] rearrangement of a silyloxonium cation. Our reaction is also applied to the highly stereoselective synthesis of fully substituted vinyl ethers.

Since its discovery 1912, the Claisen rearrangement has earned considerable attention due to its synthetic utility and intriguing mechanism.^1,2^ One of the challenges associated with the classical Claisen rearrangement is the synthesis of allyl vinyl ether substrates. Enolate alkylation can be problematic due to unselective O- vs. C-alkylation, and potential *E/Z* olefin isomer products, rendering selective substrate synthesis challenging.^3^ Other strategies for preparation of allyl vinyl ether substrates include alkyne hydroalkoxylation,^4^ carbonyl alkenylation,^5,6^ olefin isomerization,^7,8^ leaving group elimination,^9^ C–O cross coupling,^10^ and metal-catalyzed vinyl ether exchange.^11^ Methods for the *in situ* generation and subsequent direct [3,3] rearrangement of allyl vinyl ethers eliminates the need for their isolation (which can be challenging due to their sensitivity to chromatography),^12^ while offering an attractive strategy to rapidly generate complexity from simple reaction partners. Such an approach has been applied to several transition metal-catalyzed reactions (Figure 1A), such as Buchwald’s Cu-catalyzed C(*sp*^*2*^)–O cross coupling of vinyl iodides with allyl alcohols.^13^ Other approaches to intermolecular Claisen rearrangements include Au-catalyzed hydroalkoxylation of alkynes,^14^ Pd-catalyzed vinyl ether exchange,^15^ Rh-catalyzed elimination,^16^ and O–H insertion of diazo compounds.^17^ While these reports demonstrate the synthetic utility of intermolecular Claisen cascade reactions, they require transition metal catalysts and high reaction temperatures (>100 °C) to affect the thermal [3,3] rearrangement of unactivated substrates.

**Figure 1:**
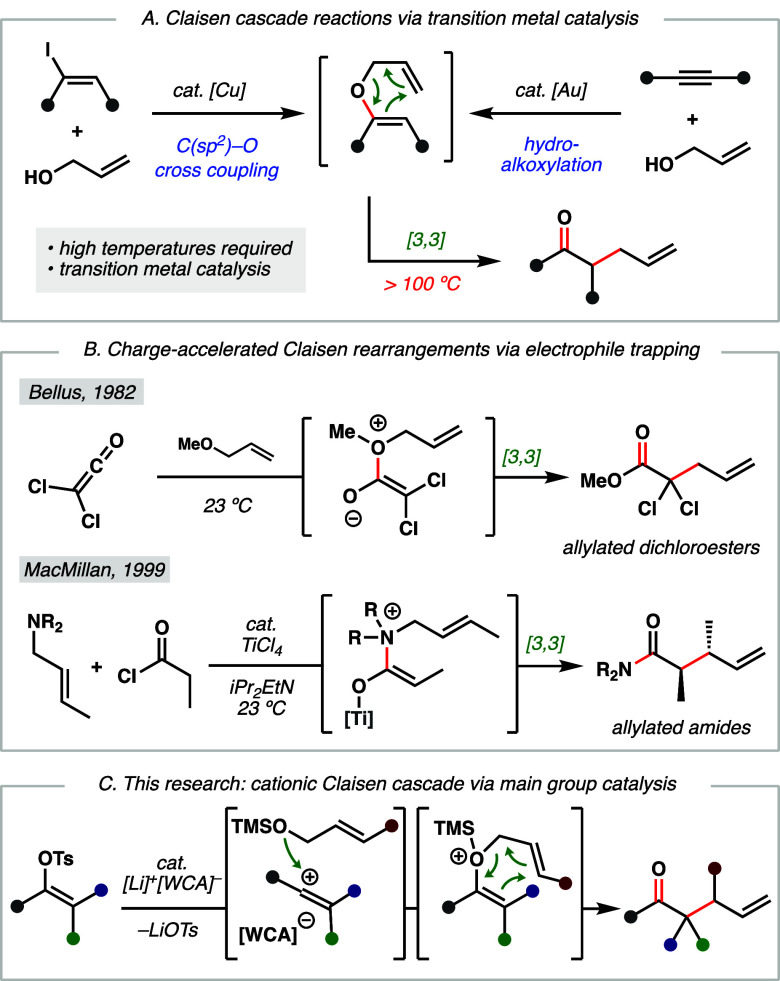
Strategies toward Claisen-type rearrangements. (A) Traditional methods enabled by transition metal catalysis. (B) Selected examples of charge accelerated Claisen rearrangements facilitated by electrophile trapping. (C) This work: a cationic Claisen rearrangement via vinyl cations.

An alternative approach stems from Bellus and co-workers’ report that highly electrophilic dichloroketenes can be trapped by allyl ethers to form zwitterionic intermediates that undergo fast [3,3] sigmatropic rearrangements (Figure 1B).^18^ MacMillan and Nubbemeyer have expanded on this work by demonstrating that simpler acyl chlorides could similarly engage allylamines via Lewis acid catalysis, wherein a charged intermediate undergoes rearrangement at room temperature (Figure 1B).^19,20^ This aza-Claisen approach has been expanded to Lewis acid activation of allenoates^21^ and additions to ketiminium ions,^22,23^ all of which have several attractive features including (1) the ability to couple two components in an intermolecular Claisen cascade reaction and (2) an acceleration effect imparted by charge, enabling rearrangement to occur at significantly lower temperatures (e.g., bracketed intermediate, Figure 1B). However, these aza-Claisen-type reactions are limited in scope, precluding their application to products analogous to those accessed by classical aliphatic Claisen rearrangements, which have found significant utility in synthetic chemistry.^48^

Inspired by the ability to generate allyl vinyl ethers through transition metal-catalyzed cross coupling reactions and the documented accelerating effects of charge in sigmatropic rearrangements,^23−25^ we envisioned a strategy that could merge the two in a transition metal-free catalytic platform. Drawing on previous work,^26^ we hypothesized that generation of a high energy vinyl carbocation and subsequent reaction with weakly nucleophilic allyl ethers would generate a vinyl oxonium cation poised to undergo a charge-accelerated [3,3] sigmatropic rearrangement (Figure 1C). Utilizing Li^+^ weakly coordinating anion (WCA) salts to ionize vinyl sulfonates,^27^ we began exploring the reactions of allyl ethers with vinyl tosylates in the presence of commercially available [Ph_3_C]^+^[B(C_6_F_5_)_4_]^−^, which generates Lewis acidic [Li]^+^[B(C_6_F_5_)_4_]^−^ upon reaction with LiHMDS. After extensive optimization (see Supporting Information), we found that 10 mol % [Ph_3_C]^+^[B(C_6_F_5_)_4_]^−^ catalyzed the reaction of trimethylsilyl (TMS) allyl ethers (2 equiv) with vinyl tosylates (**1**) in the presence of stoichiometric LiHMDS, furnishing α-allylated ketones (**2**) after 2 h of heating at 80 °C (Figure 2). In control reactions, it was validated that the presence of both catalyst and LiHMDS was crucial for productive chemistry (see Supporting Information).

**Figure 2:**
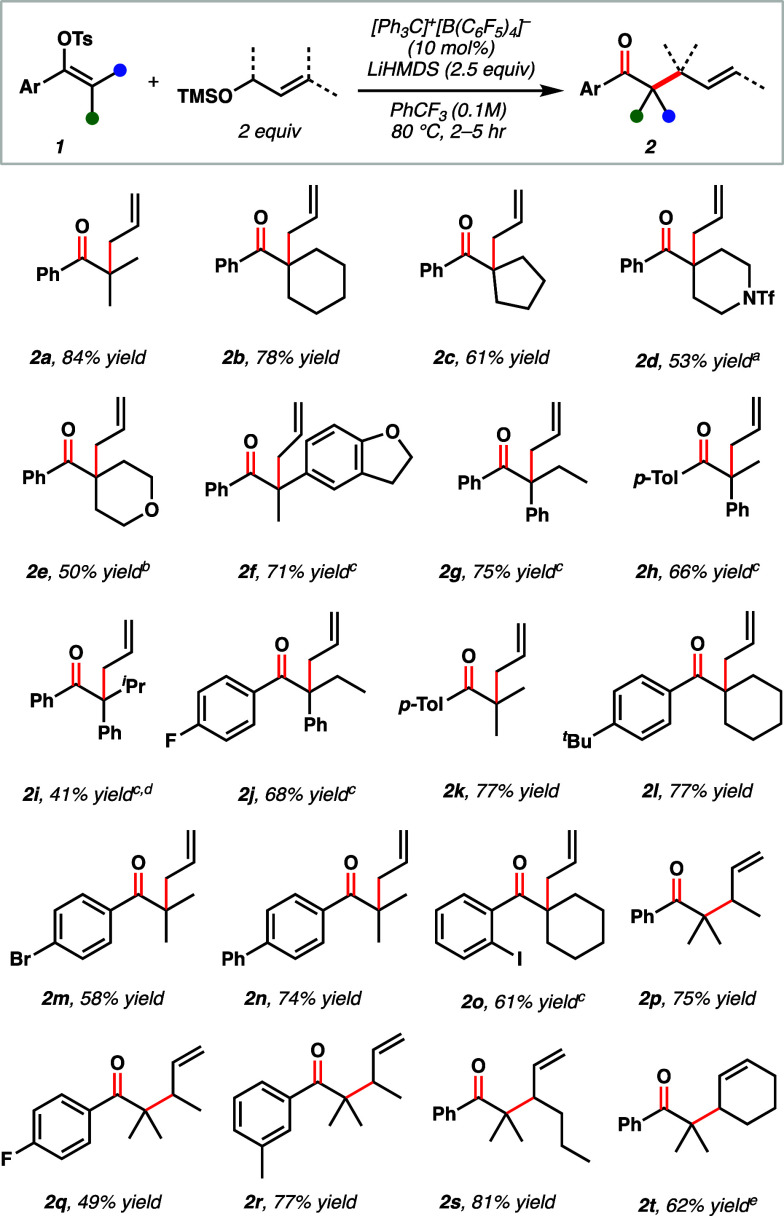
Scope of studies of catalytic Claisen reaction. Reactions were run on a 0.2 mmol scale, and reported yields are isolated yields. ^*a*^Reaction run at 100 °C for 24 h and 0.05 M with 20 mol % catalyst and 1.5 equiv LiHMDS. *^b^*0.05 M, 3 equiv LiHMDS. *^c^*Diallyl ether (2 equiv) used instead of silyl ether. *^d^*95 °C. *^e^*5 equiv silyl ether.

To explore the scope of this reaction, a range of vinyl tosylates were prepared and subjected to the optimized reaction conditions. We were pleased to find that a range of sterically congested products could be accessed in moderate-to-good yields. (Figure 2). Lewis basic heterocyclic substrates containing piperidine (**2d**), tetrahydropyran (**2e**), and dihydrobenzofuran (**2f**) groups were compatible with these Lewis acidic conditions, delivering allylated products in 50–71% yield. Both electron-rich (**2h** and **2k**) and -deficient (**2j**, **2m**, **2o**, and **2q**) vinyl tosylates led to the desired products in moderate-to-good yields. Notably, aryl bromide **2m** and iodide **2o**, which can be labile under many transition metal-catalyzed processes, were also well-tolerated. Diaryl vinyl tosylates could also undergo the tandem C–O coupling/Claisen rearrangement reaction to form products **2f**–**2j** in good yields. However, through optimization it was found that better yields were obtained with diallyl ethers instead of TMS allyl ethers with this substrate class (see Supporting Information). Variation of the alkyl substituents was demonstrated, wherein sterically congested isopropyl product **2i** could be accessed with slightly diminished yield. The allyl ether component could also be varied to selectively produce branched (**2p**–**2s**) or cyclohexenylated products (**2t**) in up to 81% yield by employing the requisite allyl ether. Given the use of strong base in this chemistry, nonfully substituted vinyl tosylate substrates were incompatible. Moreover, our scope studies elucidated that stabilizing aromatic groups are necessary for vinyl cation generation using [Li]^+^[B(C_6_F_5_)_4_]^−^.^27^

Following our substrate scope studies, we carried out experiments to probe the mechanism. Vinyl sulfonate ionization by Li-WCA salts has been demonstrated previously by our group as an effective strategy to catalytically generate vinyl carbocations.^27^ Moreover, we observed in the present study that running the reaction in benzene solvent resulted in significant Friedel–Crafts reactivity to form **3**, which is a known reaction pathway of vinyl carbocations (Figure 3A).^28^

**Figure 3:**
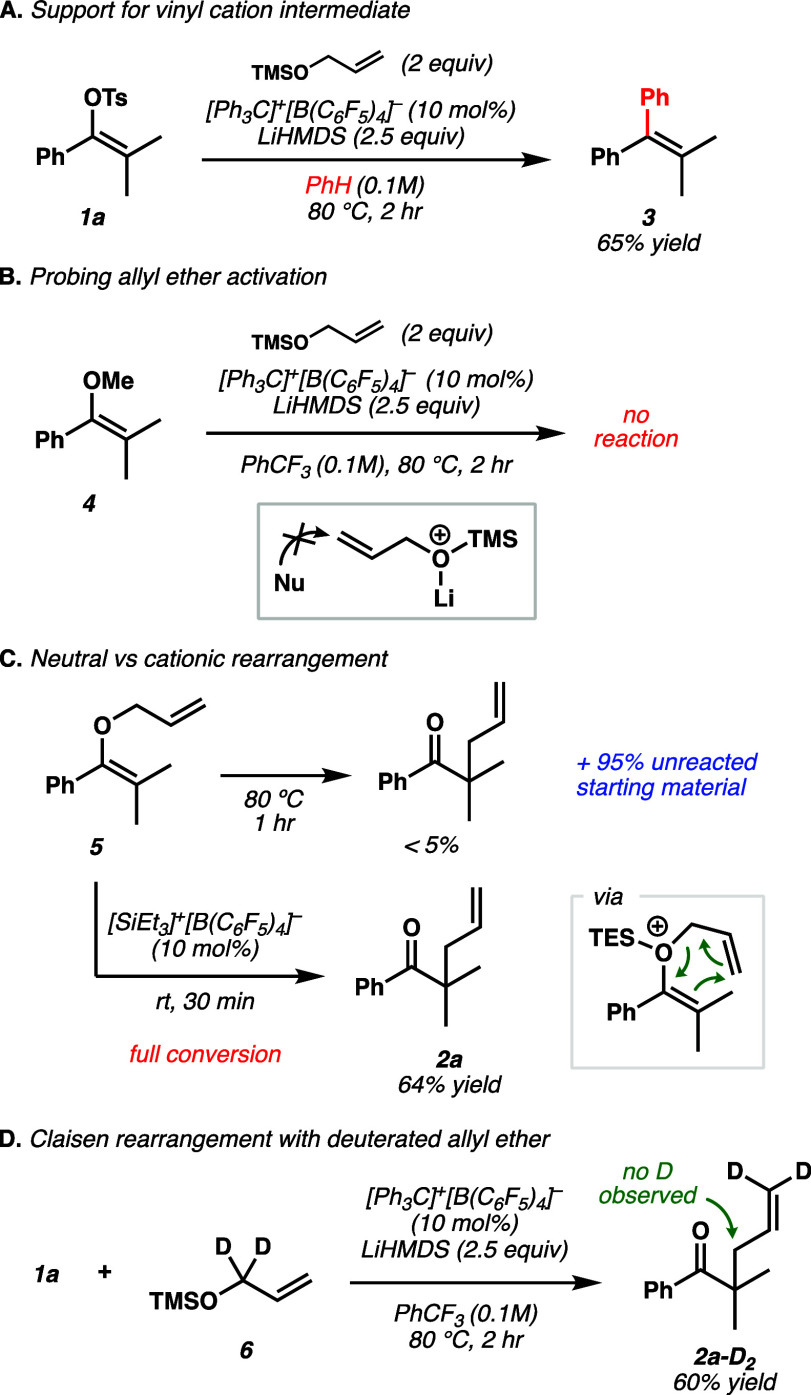
Mechanistic studies. Yields determined by NMR using nitromethane as an internal standard. (A) Observation of Friedel–Crafts arylation in benzene. (B) Evidence for cationic rearrangement via [SiR_3_]^+^[B(C_6_F_5_)_4_]^−^. (C) Evidence against enol addition to activated allyl ether via S_N_2′. (D) Isotopic labeling experiment with D_2_-allyl ether (**6**).

We propose that in non-nucleophilic solvents such as trifluorotoluene, weakly nucleophilic silyl ethers are capable of trapping electrophilic vinyl cations. While an alternative reaction pathway could involve Lewis acid activation of the allyl ether followed by nucleophilic attack from an enol species, no reaction with vinyl ether **4** was observed under the reaction conditions (Figure 3B). We next wanted to address our hypothesis that an initial cationic vinyl silyloxonium intermediate was undergoing a charge accelerated Claisen rearrangement (Figure 1C). While the reactions in this study are heated to 80 °C, we hypothesize this temperature is required for vinyl cation formation.

Furthermore, the proposed cationic [3,3] rearrangement is likely to be facile at lower temperatures. To probe these hypotheses, we prepared allyl vinyl ether **5** and found that the neutral Claisen rearrangement is indeed sluggish at 80 °C (<5% yield after 1 h); however, the addition of catalytic [SiEt_3_]^+^[B(C_6_F_5_)_4_]^−^ resulted in rapid conversion to the Claisen product (**2a**) at room temperature in 30 min (Figure 3C). These findings are consistent with our mechanistic proposal and reported accelerating effects of Claisen rearrangements induced by positive charge.^23−25^ Finally, deuterated allyl TMS ether **6** furnished product **2a-D**_**2**_ with no sign of deuterium incorporation at the allylic position by NMR, consistent with a concerted [3,3] rearrangement (Figure 3D).

The reaction pathway was further studied utilizing density functional theory (DFT) calculations, wherein the proposed cationic rearrangement was found to possess a significantly lower barrier (**TS1’**, Δ*G*^‡^ = 13.6 kcal/mol) compared to the neutral pathway (**TS1**, Δ*G*^‡^ = 28.9 kcal/mol) (Supporting Information, Figure S2). Additionally, CM5 calculations were conducted on **TS1** and **TS1’** to compare the charge delocalization in the allyl substructures in the transition states (see Supporting Information, Figure S1). The carbocation in the allyl substructure of **TS1’** is found to be more delocalized, and the lower kinetic barrier is attributed to significant destabilization of the vinyl ether **INT1’** relative to its transition state (**TS1’**). Furthermore, intrinsic reaction coordinate (IRC) calculations predicted the concerted [3,3] rearrangement with oxonium **INT1’**, consistent with the isotopic labeling experiments.

Based on these results, our proposed mechanism commences with *in situ* formation of Lewis acidic [Li]^+^[B(C_6_F_5_)_4_]^−^ (Figure 4).^27^ Ionization of vinyl tosylate (**1**) generates a vinyl carbocation (**7**),^29^ which is trapped by the allyl ether nucleophile to afford a silyloxonium (**INT1’**) that is poised to undergo a cationic [3,3] sigmatropic rearrangement. Desilylation by LiHMDS produces N(TMS)_3_ (observed by GC) and ketone product **2**, while also regenerating catalytic [Li]^+^[B(C_6_F_5_)_4_]^−^.

**Figure 4:**
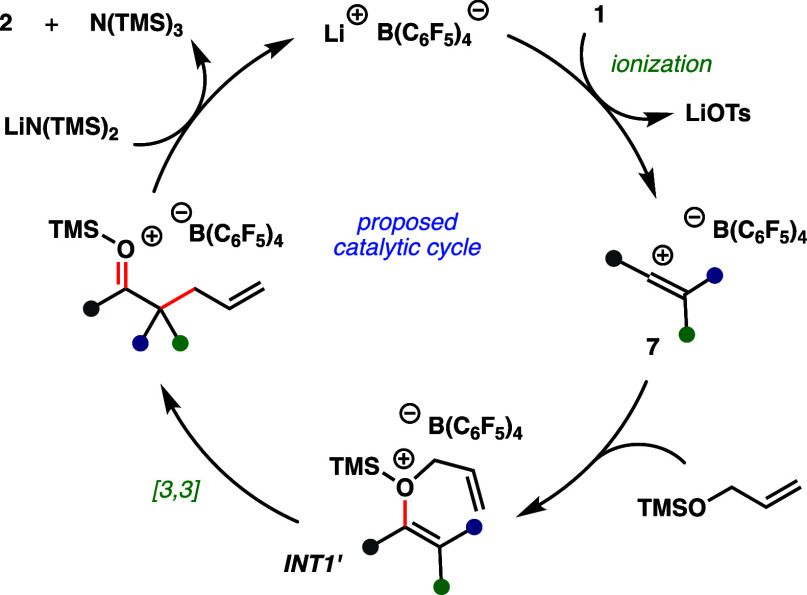
Proposed mechanism.

During our scope studies, we found that prenyl ether **SI-1** generated low yields of the expected [3,3] product **SI-2**, instead forming α-prenylated **SI-3** as a major product (see Supporting Information, Figure S2). While deuterated allyl ether **6** demonstrated clean conversion to a single observable isotopomer, this result was unexpected and suggested a competing [1,3] rearrangement could be operative. Ether **SI-1** was computed to undergo a stepwise rearrangement involving fragmentation and subsequent recapture to access **SI-2**, perhaps due to increased stabilization of the prenyl carbocation. The energy barrier for this (3,3) rearrangement appears to still be favored relative to the barrier of a concerted [1,3] rearrangement; however, the (3,3) intermediate **INT3a** is significantly less stable than [1,3] intermediate **INT2a’** given its vicinal all-carbon quaternary centers. Based on these energies, the (3,3) rearrangement can be reversible if desilylation of **INT3a** by LiHMDS is slow. In fact, we found experimentally that the product distribution of the reaction using ether **SI-1** was dependent on LiHMDS concentration, wherein higher equivalents resulted in significantly increased amounts of the (3,3) product (1:4 ratio **SI-2**-to-**SI-3**) (see Supporting Information, Figure S2). Based on these experimental and computational results, the observed preference for product **SI-3** could be thermodynamically driven. Thus, these findings highlight that increased substitution on the allyl fragment results in increasing deviation from a concerted [3,3] pathway.

We recognized that our developed methodology could be extended toward a simple platform for accessing highly substituted vinyl ethers. These are useful functional groups in organic synthesis, employed beyond the Claisen rearrangement in a range of chemical reactions such as cycloadditions,^30,31^ Nazarov cyclization,^32^ and polymerization.^33,34^ While the geometry of vinyl ethers can dictate stereochemical outcomes in their reactions, methods for the stereoselective synthesis of vinyl ethers are limited, especially for highly substituted substrates.^5^ Recently, Yoshikai and co-workers reported a method for the stereoselective iodo(III)etherification of alkynes to access fully substituted iodanyl vinyl ethers.^35^ However, this reaction required excess I(III) reagent (3 equiv of IBX), large excess of alcohol nucleophile (as solvent or in some cases 5 equiv), and a second step to convert the benziodoxole moiety into a more desirable functional group.

To this end, we found that catalytic quantities (8–10 mol %) of [Ph_3_C]^+^[B(C_6_F_5_)_4_]^−^ promoted coupling of TMS alkoxyethers (1.5 equiv) with vinyl tosylates to generate fully substituted enol ether products with high selectivity (up to 62:1/ *E/Z*) at 35 °C (Figure 5). Electron-poor (**8b**, **8f**), -rich (**8c**), and heterocyclic (**8e**) substrates were all tolerated and demonstrated high stereoselectivities. Aryl iodide-containing substrates (**8f**, **8i**), which can be problematic in the case of many transition metal-based C(*sp*^*2*^)–O coupling protocols, were compatible under standard conditions. This reaction was compared to published enolate O-alkylation approaches, which demonstrated comparable yields but low selectivity for all substrates tested in this study, ranging between 1:1 to 2.2:1 *E*/*Z* (see Supporting Information).^36^ When steric discrimination between the aryl and alkyl groups becomes less pronounced, as in the case of substrate **8d**, the selectivity is diminished to 12:1. This is consistent with a kinetically controlled nucleophilic addition to the vinyl cation. Furthermore, this reaction proved tolerant of a variety of other silyl ethers, including natural product derivatives (**8j**) and thioethers (**8n**). Interestingly, silyl enol ether substrates show high preference for C–O bond formation instead of C–C formation, generating divinyl ether **8k** in good yield.

**Figure 5:**
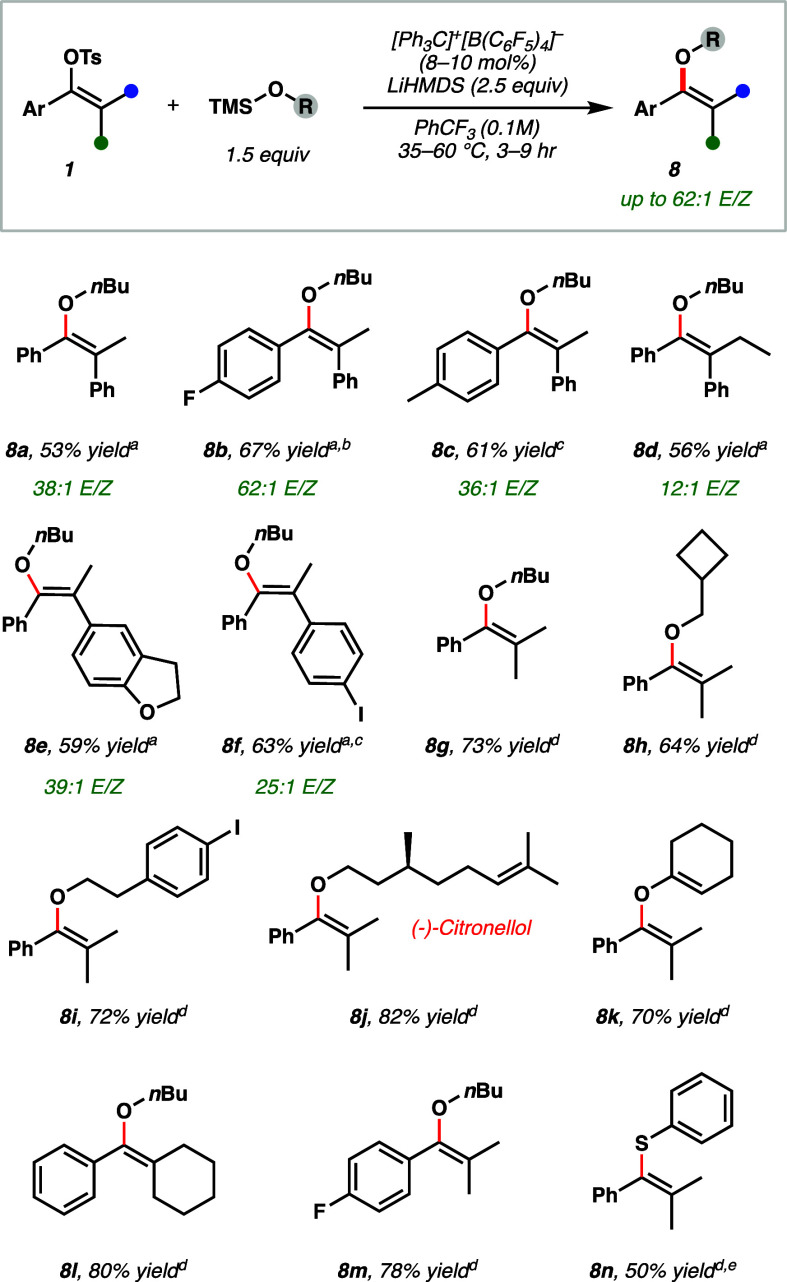
Scope of studies of catalytic vinyl ether synthesis. Reactions run on a 0.2 mmol scale of a single isomer (as drawn in the product), and reported yields are isolated yields. *E*/*Z* ratio was determined by GC-FID of an authentic sample of the minor *Z* isomer. *^a^*Reaction run at 35 °C for 6 h with 10 mol % catalyst. *^b^*9 h. *^c^* 60 °C. *^d^*Reaction run at 60 °C for 3 h with 8 mol % catalyst. *^e^*TMS–SPh (1.5 equiv) used, run for 6 h.

In summary, we have disclosed a new catalytic C–O coupling/Claisen rearrangement cascade reaction using simple, commercially available borate salts as catalysts. The reaction was demonstrated on various substrates, showcasing the ability to construct sterically hindered C–C bonds. Mechanistic experiments and DFT calculations support a cationic [3,3] rearrangement of a silyloxonium intermediate produced upon trapping of a catalytically generated vinyl cation by allyl ethers. Finally, our reaction was applied to the stereoselective synthesis of fully substituted vinyl ethers.

## Data Availability

The data underlying this study are available in the published article and its Supporting Information.

## Supporting Information Available

Experimental procedures, characterization data, NMR spectra, and computational data (PDF)
