# Supplementary material for: Main Group-Catalyzed Cationic Claisen Rearrangements via Vinyl Carbocations
Source: Org Lett. 2024 Jun 6;26(23):4847–52. doi: 10.1021/acs.orglett.4c00837 (PMC11187624; doi:10.1021/acs.orglett.4c00837)
Supplement: Supplementary file 1 — ol4c00837_si_001.pdf [file ol4c00837_si_001.pdf]

Supplementary Materials for

Main-Group Catalyzed Cationic Claisen Rearrangements *via* Vinyl Carbocations

Chloe G. Williams†#, Sepand K. Nistanaki†#, Krista Dong†, Woojin Lee‡, Kendall N. Houk‡*, and Hosea M. Nelson†*

†*Division of Chemistry and Chemical Engineering, California Institute of Technology, Pasadena, CA 91125, USA.*

‡*Department of Chemistry and Biochemistry, University of California, Los Angeles, Los Angeles, CA 90095, USA.*

*Corresponding authors. Email: hosea@caltech.edu (H.M.N.), houk@chem.ucla.edu (K.N.H)
#C.G.W. and S.K.N. contributed equally.

Table of Contents

1. Figures S1 and S2 3
2. Materials and Methods 4–5
3. Preparation of Vinyl Tosylate Substrates 5–17
4. Preparation of Silyl Ethers 18–20
5. Catalytic Claisen Cascade Coupling Reactions 21–32
5.1 Reaction Optimization 21
5.2 Scope Studies and Product Characterization 21–32
6. Catalytic Stereoselective Vinyl Ether Synthesis 32–50
6.1 Reaction Optimization 32
6.2 Scope Studies and Product Characterization 33–41
6.3 Preparation of Authentic Minor Isomers via Enolate Alkylation 41–45
6.4 GC-FID Data for Stereoselective Vinyl Ether Synthesis 46–51
7. Mechanistic Studies 52–60
7.1 Support for Vinyl Cation Intermediacy 52
7.2 Neutral vs Cationic Claisen Rearrangement 52–54
7.3 Activation of Allyl Ether 54
7.4 Claisen with Deuterated Allyl Ether 54–56
7.5 Product Distribution with Substituted Ethers 56–60
8. Computational Methods 61–76
8.1 Computational Methods 61

8.2 Calculated Energies 61–62

8.3 Calculated Cartesian coordinates 62–75

8.4 3-D Figures of Calculated Structures 75–77

8.5 Charge calculations for TS1 and TS1' 78

9. References 79–80

10. NMR Spectra 81–227

1- Figures S1 and S2

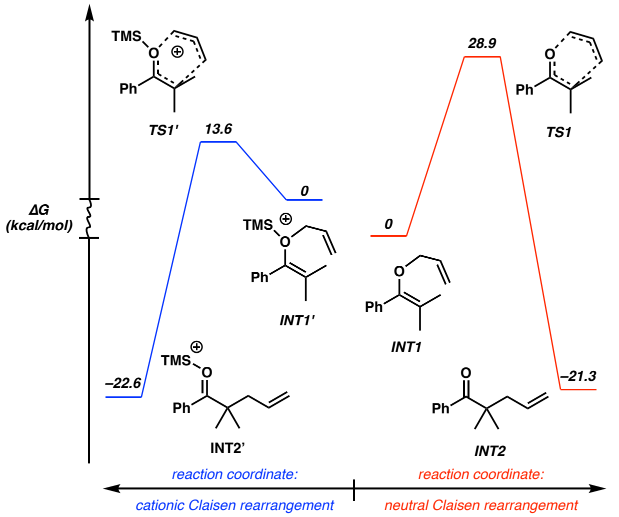

Figure S1. Cationic and neutral pathway DFT calculations, (ωB97X-D/def2-TZVPP/CPCM(CyH) // ωB97X-D/def2-SVP/CPCM(CyH) 353.15K).

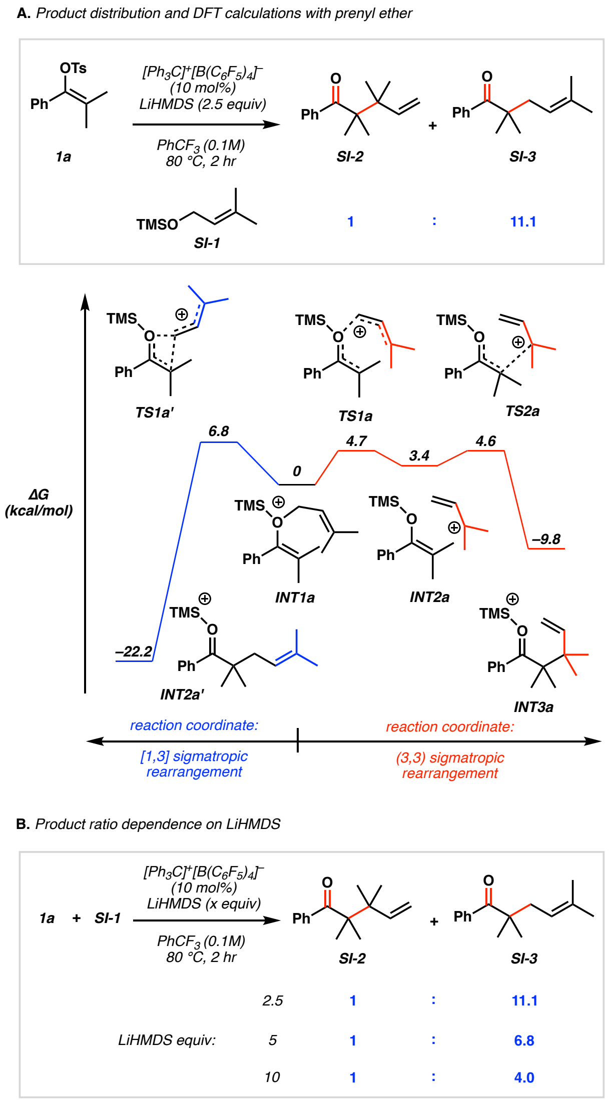

Figure S2. Study of prenyl allyl ether reactivity. (A) Reaction outcome and DFT analysis of prenyl allyl ether. Product ratios were determined by ^{1}H NMR. (B) Experimental LiHMDS-dependence on product ratio. DFT, (ωB97X-D/def2-TZVPP/CPCM(CyH) // ωB97X-D/def2-SVP/CPCM(CyH) 353.15K).

2- Materials & Methods

Unless otherwise stated, all reactions were performed in an MBraun or VAC glovebox under nitrogen atmosphere with ≤ 0.5 ppm O_2 levels. All glassware and stir-bars were dried in a 160 °C oven for at least 12 hours and cycled directly into the glovebox for use. Solid substrates were dried on high vacuum over P_2O_5 overnight, and liquid substrates were dried in a glovebox by passing through activated neutral alumina. All solvents were rigorously dried before use. Benzene and trifluorotoluene were degassed and dried in a JC Meyer solvent system and stored inside a glovebox. Cyclohexane was distilled over potassium. *o*-Difluorobenzene was distilled over CaH_2. All other solvents used for substrate synthesis were dried in a JC Meyer solvent system. Preparatory thin layer chromatography (TLC) was performed using Millipore silica gel 60 F_{254} pre-coated plates (0.25 mm) and visualized by UV fluorescence quenching. Silia P60 silica gel (230-400 mesh) was used for column chromatography. NMR spectra were recorded on a Bruker 400 MHz with Prodigy cryoprobe (^{1}H, ^{13}C), a Bruker 400 MHz (^{1}H, ^{19}F), and a Varian 500 MHz (^{1}H). ^{1}H NMR spectra are reported relative to $CDCl_3$ (7.26 ppm) unless noted otherwise. Data for ^{1}H NMR spectra are as follows: chemical shift (ppm), multiplicity, coupling constant (Hz), integration. Multiplicities are as follows: s = singlet, d = doublet, t = triplet, dd = doublet of doublet, dt = doublet of triplet, ddd = doublet of doublet of doublet, td = triplet of doublet, m = multiplet. ^{13}C NMR spectra are reported relative to $CDCl_3$ (77.1 ppm) unless noted otherwise. Structural assignments were made with additional information from gNOESY and gCOSY experiments. IR Spectra were record on a Thermo Scientific Nicolet iS50 FT-IR and are reported in terms of frequency absorption (cm^{-1}). High resolution mass spectra (HR-MS) were recorded on an Agilent 6230 time-of-flight LC/MS (LC/TOF) using electrospray ionization (ESI) or acquired by the Caltech Mass Spectral Facility by Field Ionization/Field Desorption mass spectrometry using a JEOL AccuTOF GC-Alpha (JMS-T2000GC) mass spectrometer interfaced with an Agilent 8890 GC system. Ions were detected as M+ (radical cations). All commercial chemicals and reagents were used as received, unless otherwise noted. Solid lithium hexamethyldisilazide and potassium hexamethyldisilazide were purchased from Sigma Aldrich and brought in the glovebox as received. Trityl tetrakis(pentafluorophenyl)borate was purchased from TCI and brought in the glovebox and used as received. Commercial allyloxytrimethylsilane (Sigma Aldrich) and diallylether (TCI) were dried by passing through activated neutral alumina in a glovebox. TMSCl was distilled prior to use. Other reagents include: imidazole (Fisher Scientific), KO*t*Bu (Sigma Aldrich), iodomethane and iodoethane (Oakwood Chemicals), Ts_2O (Oakwood Chemicals), and DMEA (Oakwood

Chemicals). Commercial alcohols were purchased from Sigma Aldrich, Oakwood Chemicals, and Fisher Scientific.

3- Preparation of Vinyl Tosylate Substrates

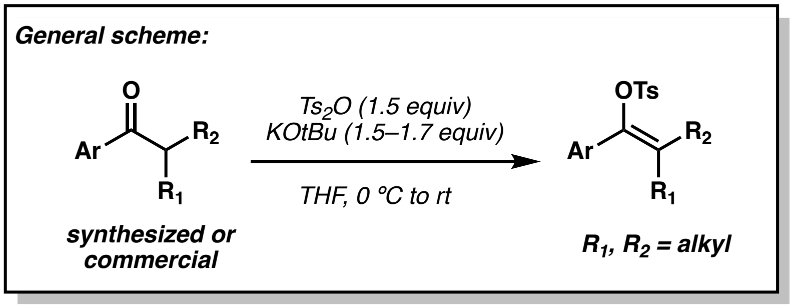

General Procedure 1: *Vinyl tosylate substrates were prepared from ketones that were either commercially available or synthetically made according to a published literature procedure. This procedure for dialkyl vinyl tosylates is as follows:* To a flame-dried flask, commercially available ketone or otherwise synthetically made (10.0 mmol, 1.0 equivalent), was dissolved in THF (0.33 M). The solution was cooled to 0 ºC, and then a solution of KOtBu (15.0–17.0 mmol, 1.5–1.7 equivalents, 1 M THF) was added dropwise. The resulting solution was then stirred at 0 ºC for 2 hours. Next, Ts_2O (15.0 mmol, 1.5 equivalents) was added as a solution in THF (0.6 M) to the enolate solution with vigorous stirring, and then the solution was allowed to warm to room temperature and stirred until completion. Once starting material is consumed, the reaction was diluted with EtOAC and water. The organic layer was separated, and the aqueous layer was extracted 3x, dried over Na_2SO_4, filter, concentrated *in vacuo*, and purified by silica gel column chromatography (ether/hexanes) to give vinyl tosylate.

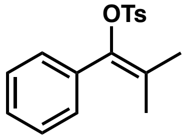

2-methyl-1-phenylprop-1-en-1-yl 4-methylbenzenesulfonate (SI-4) was prepared according to *General Procedure 1* from commercially available ketone. Spectra matched the NMR data in the literature.[1]

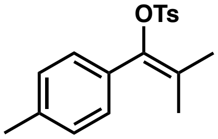

2-methyl-1-(*p*-tolyl)prop-1-en-1-yl 4-methylbenzenesulfonate (SI-5) was prepared according to the *General Procedure 1* from 2-methyl-1-(*p*-tolyl)propan-1-one. Spectra matched the NMR data in the literature.[1]

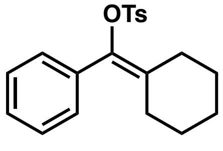

cyclohexylidene(phenyl)methyl 4-methylbenzenesulfonate (SI-6) was prepared according to *General Procedure 1* from commercially available ketone. Spectra matched the NMR data in the literature.[1]

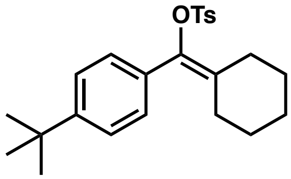

(4-(*tert*-butyl)phenyl)(cyclohexylidene)methyl 4-methylbenzenesulfonate (SI-7) was prepared according to *General Procedure 1* from (4-(*tert*-butyl)phenyl)(cyclohexyl)methanone**.** Spectra matched the NMR data in the literature.[1]

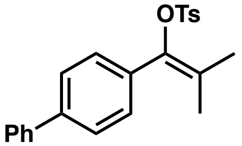

1-([1,1'-biphenyl]-4-yl)-2-methylprop-1-en-1-yl 4-methylbenzenesulfonate (SI-8)
was prepared according to *General Procedure 1* from **(**1-([1,1'-biphenyl]-4-yl)-2-methylpropan-1-one. Spectra matched the NMR data in the literature.[1]

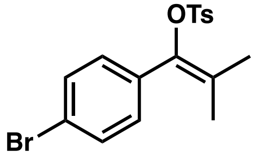

1-(4-bromophenyl)-2-methylprop-1-en-1-yl 4-methylbenzenesulfonate (SI-9) was prepared according to *General Procedure 1* from commercially available ketone. Spectra matched the NMR data in the literature.[1]

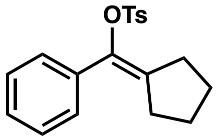

cyclopentylidene(phenyl)methyl 4-methylbenzenesulfonate (SI-10) was prepared according to *General Procedure 1* from commercially available ketone on a 15.0 mmol scale (2.6g, 53% yield).

^{1}H NMR (400 MHz, $CDCl_3$) δ 7.59 – 7.49 (m, 2H), 7.20 – 7.07 (m, 7H), 2.54 (dtd, *J* = 7.4, 3.6, 1.4 Hz, 2H), 2.42 (qt, *J* = 5.6, 2.6 Hz, 2H), 2.35 (s, 3H), 1.68 (dqd, *J* = 5.5, 4.3, 2.0 Hz, 4H).

^{13}C NMR (101 MHz, $CDCl_3$) δ 144.4, 138.9, 138.4, 134.8, 134.4, 129.3, 128.1, 127.8, 127.8, 127.7, 127.6, 31.6, 31.0, 27.2, 25.7, 21.6.

FT-IR (neat film NaCl): 3056, 2956, 2869, 1598, 1494, 1445, 1366, 1292, 1259, 1189, 1175, 1095, 997, 951, 820, 805, 784, 697, 552 cm^{-1}.

HR-MS (FI^+) m/z: [M+] Calculated for $C_{19}H_{20}O_3S$ 328.1133; Found 328.1139.

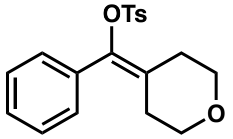

phenyl(tetrahydro-4*H*-pyran-4-ylidene)methyl 4-methylbenzenesulfonate (SI-11) was prepared according to *General Procedure 1* from commercially available ketone on a 15.8 mmol scale (3.5 g, 64% yield).

^{1}H NMR (400 MHz, $CDCl_3$) δ 7.50 – 7.39 (m, 2H), 7.15 (dddd, *J* = 12.3, 7.9, 6.5, 3.3 Hz, 5H), 7.11 – 7.04 (m, 2H), 3.72 (t, *J* = 5.5 Hz, 2H), 3.67 – 3.59 (m, 2H), 2.53 (dd, *J* = 5.9, 5.1 Hz, 2H), 2.34 (s, 3H), 2.32 (m, 2H).

^{13}C NMR (101 MHz, $CDCl_3$) δ 144.5, 140.2, 134.1, 133.1, 129.6, 129.3, 128.6, 128.4, 128.1, 128.0, 68.3, 68.2, 30.5, 29.3, 21.6.

FT-IR (neat film NaCl): 3057, 2962, 2910, 2847, 1598, 14, 1366, 1295, 1188, 1174, 1094, 1014, 988, 785, 699, 574, 557 cm^{-1}.

HR-MS (ESI) m/z: [M+Na]+ Calculated for $C_{19}H_{20}NaO_4S$ 367.0975; Found 367.0982.

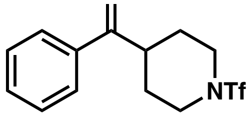

phenyl(1-((trifluoromethyl)sulfonyl)piperidin-4-yl)methanone (SI-12)

To a flame dried flask, commercially available 4-Benzoylpiperidine hydrochloride (1.10 g, 1.0 equivalent, 4.9 mmol) was added followed by dry DCM (16 mL, 0.3 M). Dry triethylamine (3.4 mL, 5 equivalents, 24.4 mmol) was added next and the reaction was cooled to 0 ºC. Anhydrous Tf_2O (900 uL, 1.1 equivalents, 5.4 mmol) was then added and the reaction was allowed to warm to room temperature and stir overnight. The next day water was added and the organic layer was washed 3x with water and then dried over Na_2SO_4. The crude reaction mixture was purified via column chromatography 10 → 15% ethyl acetate/hexanes to afford **SI-12** as a solid (600 mg, 38%).

^{1}H NMR (500 MHz, $CDCl_3$) δ 7.95 – 7.91 (m, 2H), 7.64 – 7.56 (m, 1H), 7.50 (t, *J* = 7.8 Hz, 2H), 3.99 (d, *J* = 13.2 Hz, 2H), 3.53 – 3.45 (m, 1H), 3.29 (s, 2H), 2.05 – 1.99 (m, 2H), 1.92 (dtd, *J* = 14.4, 10.6, 4.1 Hz, 2H).

^{13}C NMR (101 MHz, $CDCl_3$) δ 200.9, 135.4, 133.5, 128.9, 128.25, 120.1 (q, *J* = 323.1 Hz), 45.9, 28.1.

^{19}F NMR (376 MHz, $CDCl_3$) δ -74.2.

FT-IR (neat film NaCl): 2951, 2871, 1677, 1597, 1582, 1448, 1381, 1367, 1337, 1313, 1296, 1269, 1228, 1183, 1145, 1110, 1062, 950, 844, 784, 763, 703, 688, 667, 586, 578, 469 cm^{-1}.

HR-MS (ESI) m/z: [M+H]+ Calculated for $C_{13}H_{15}F_3NO_3S^+$ 322.0720; Found 322.0734.

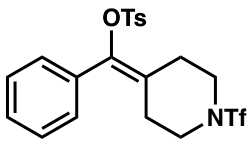

phenyl(1-((trifluoromethyl)sulfonyl)piperidin-4-ylidene)methyl 4-methylbenzenesulfonate (SI-13) was prepared according to *General Procedure 1* from **SI-12** on a 1.8 mmol scale (500 mg, 56% yield).

^{1}H NMR (400 MHz, $CDCl_3$) δ 7.48 – 7.37 (m, 2H), 7.25 – 7.14 (m, 3H), 7.14 – 7.03 (m, 4H), 3.89 – 3.14 (broad m, 4H), 2.66 (broad, 2H), 2.42 (t, *J* = 5.8 Hz, 2H), 2.35 (s, 3H).

^{13}C NMR (101 MHz, $CDCl_3$) δ 144.8, 142.1, 133.8, 132.4, 129.6, 129.5, 128.9, 128.2, 128.0, 126.1, 120.1 (q, *J* = 323.2 Hz), 47.3, 47.1, 29.4, 28.3, 21.6.

^{19}F NMR (376 MHz, $CDCl_3$) δ -75.6.

FT-IR (neat film NaCl): 3059, 2926, 2878, 1598, 1388, 1370, 1226, 1187, 1175, 1150, 1095, 1017, 947, 867, 785, 701, 676, 590, 553 cm^{-1}.

HR-MS (ESI) m/z: [M+Na]+ Calculated for $C_{20}H_{20}F_3NNaO_5S_2$ 498.0627; Found 498.0626.

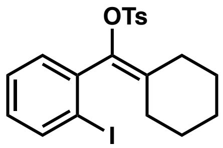

cyclohexylidene(2-iodophenyl)methyl 4-methylbenzenesulfonate (SI-14) was prepared according to *General Procedure 1* from cyclohexyl(2-iodophenyl)methanone**.** Spectra matched the NMR data in the literature.[1]

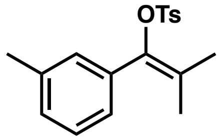

2-methyl-1-(*m*-tolyl)prop-1-en-1-yl 4-methylbenzenesulfonate (SI-15) was prepared according to *General Procedure 1* from 2-methyl-1-(*m*-tolyl)propan-1-one on a 15.4 mmol scale (0.546 g, 11% yield).

^{1}H NMR (400 MHz, $CDCl_3$) δ 7.46 – 7.38 (m, 2H), 7.07 – 7.01 (m, 3H), 7.00 – 6.91 (m, 2H), 6.84 (tt, *J* = 1.7, 0.8 Hz, 1H), 2.34 (s, 3H), 2.15 (s, 3H), 1.90 (s, 3H), 1.75 (s, 3H).

^{13}C NMR (101 MHz, $CDCl_3$) δ 144.1, 141.5, 137.3, 134.5, 133.6, 130.1, 129.1, 128.6, 128.0, 127.6, 126.9, 126.2, 21.6, 21.2, 20.1, 19.1.

FT-IR (neat film NaCl): 2993, 2918, 2860, 1599, 1450, 1364, 1189, 1175, 1083, 1019, 911, 822, 805, 791, 714, 674, 587, 569, 549 cm^{-1}.

HR-MS (ESI) m/z: [M+Na]+ Calculated for $C_{18}H_{20}NaO_3S$ 339.1025; Found 339.1027.

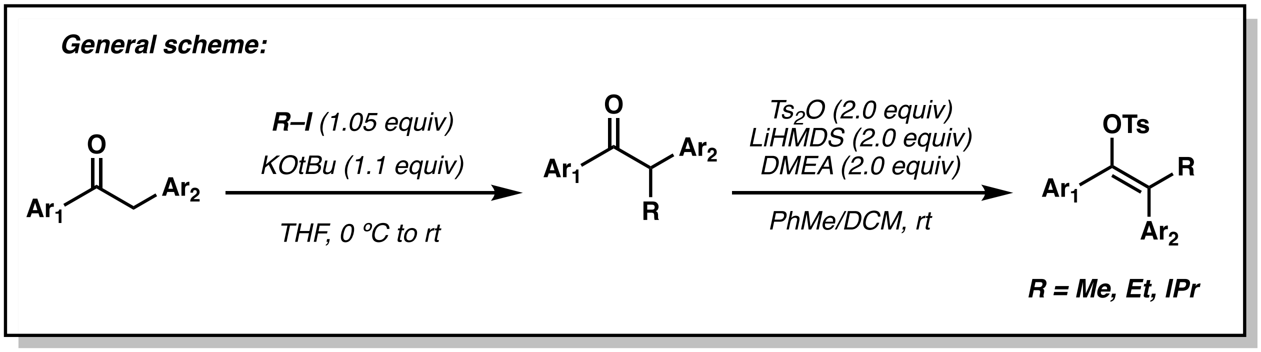

General Procedure 2: *Diaryl vinyl tosylate substrates were synthesized according to published literature procedures from the corresponding Weinreb amide or commercially available ketones. The tosylation step follows a known literature procedure for diaryl vinyl tosylate substrates.*[2,3]

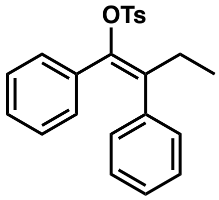

(*E*)-1,2-diphenylbut-1-en-1-yl 4-methylbenzenesulfonate (SI-16) was prepared according to known literature procedures from commercially available 1,2-diphenylbutan-1-one and spectra matched the reported literature.[3]

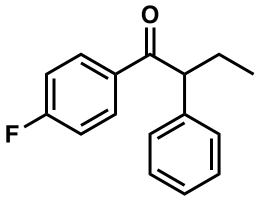

1-(4-fluorophenyl)-2-phenylbutan-1-one (SI-17)

To a flame dried flask, commercially available 1-(4-fluorophenyl)-2-phenylethan-1-one (750 mg, 1 equivalent, 3.50 mmol) was added followed by THF (7 mL, 0.5 M) and cooled to 0 ºC. Then, a solution of KOtBu (471 mg, 1.2 equivalents, 4.20 mmol) in THF (3.8 ml, 1.1 M) was added dropwise. The reaction was allowed to stir for 20 minutes, and then iodoethane (0.37 mL, 1.3 equivalents, 4.55 mmol) was added dropwise. The reaction was allowed to warm to room temperature and stirred until starting material was consumed as determined by TLC (5% diethyl ether/hexanes). Then, 2M HCl was added and the reaction was extracted EtOAc 3x. The combined organics were washed with water, brine, and then dried of MgSO4. The crude mixture was purified via column chromatography 3% diethyl ether/pentanes to afford **SI-17** (780 mg, 92% yield), which matched the NMR data in the literature.[3]

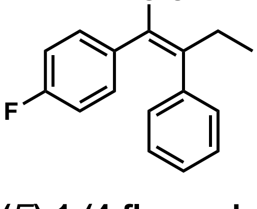

(*E*)-1-(4-fluorophenyl)-2-phenylbut-1-en-1-yl 4-methylbenzenesulfonate (SI-18) was prepared according to known literature procedures from commercially available **SI-17.** Spectra matched the NMR data in the literature.[3]

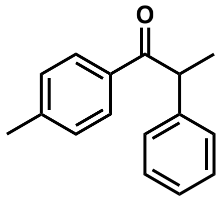

2-phenyl-1-(*p*-tolyl)propan-1-one (SI-19)

To a flame dried flask, commercially available 2-phenyl-1-(*p*-tolyl)ethan-1-one (1.17 g, 1 equivalent, 5.54 mmol) was added followed by THF (11 mL, 0.5 M) and cooled to 0 ºC. Then, a solution of KOtBu (747 mg, 1.2 equivalents, 6.65 mmol) in THF (7 ml, 0.95 M) was added dropwise. The reaction was allowed to stir for 20 minutes, and then iodomethane (0.38 mL, 1.1 equivalents, 6.1 mmol) was added dropwise. The reaction was allowed to warm to room temperature and stirred until starting material was consumed as determined by TLC (5% diethyl ether/hexanes). Then, 2M HCl was added and the reaction was extracted EtOAc 3x. The combined organics were washed with water, brine, and then dried of MgSO4. The crude mixture was purified via column chromatography 3% diethyl ether/pentanes to afford **SI-19** (0.852 g, 73% yield), which matched the NMR data in the literature.[4]

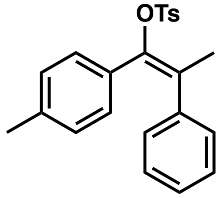

(*E*)-2-phenyl-1-(p**-tolyl)prop-1-en-1-yl 4-methylbenzenesulfonate (SI-20)** was prepared according to known literature procedure for similar vinyl tosylates: to a flame dried Schlenk flask was added LiHMDS (1.27 g, 2 equivalents, 7.6 mmol) inside of a glovebox. The Schlenk flask was capped, removed, and anhydrous PhMe (8.4 mL, 0.90 M) was added followed by DMEA (0.82 mL, 2 equivalents, 7.6 mmol). To another flame dried flask was added **SI-19** (0.852 g, 1 equivalent, 3.8 mmol) followed by anhydrous PhMe (3.8 mL, 1 M). The ketone solution was then added dropwise to the LiHMDS solution at room temperature. The reaction was allowed to stir for 20 minutes. To another flame dried flask was added Ts_2O (2.48 g, 2 equivalents, 7.6 mmol) with anhydrous DCM (20 mL, 0.38 M). The Ts_2O solution was then added dropwise to the enolate solution with vigorous stirring. *Note: the solution becomes very thick.* The reaction was monitored by TLC (20% diethyl ether/hexanes), and after 1 hour it was complete. A few mLs of 1M NaOH was then added, and the solution became homogenous. Additional water was added, and then reaction was extracted 3x with diethyl ether. The combined organics were then dried over Na_2SO_4

and the crude mixture was concentrated in vacuo. The material was purified by column chromatography (5% diethyl ether/hexanes) to afford pure **SI-20** as a white solid (600 mg, 43% yield).

^{1}H NMR (400 MHz, $CDCl_3$) δ 7.55 – 7.47 (m, 2H), 7.20 – 7.14 (m, 3H), 7.13 – 7.09 (m, 2H), 7.07 – 7.01 (m, 2H), 6.80 (d, *J* = 8.2 Hz, 2H), 6.75 – 6.69 (m, 2H), 2.37 (s, 3H), 2.18 (s, 3H), 2.17 (s, 3H).

^{13}C NMR (101 MHz, $CDCl_3$) δ 144.4, 143.6, 140.2, 137.7, 134.5, 131.1, 130.2, 129.8, 129.3, 128.8, 128.2, 128.1, 127.1, 21.7, 21.3, 19.9.

FT-IR (neat film NaCl): 3054, 3028, 2921, 2861, 1598, 1442, 1367, 1190, 1176, 1085, 1040, 967, 854, 823, 814, 768, 760, 700, 670, 581, 558, 545 cm^{-1}.

HR-MS (ESI) m/z: [M+Na]+ Calculated for $C_{23}H_{22}NaO_3S$ 401.1182; Found 401.1184.

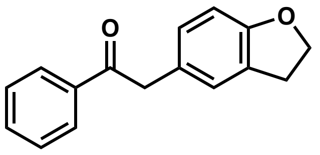

2-(2,3-dihydrobenzofuran-5-yl)-1-phenylethan-1-one (SI-21) was prepared according to a known literature procedure from commercially available 2,3-dihydrobenzofuran-5-acetic acid.[5]

^{1}H NMR (400 MHz, $CDCl_3$) δ 8.10 – 7.96 (m, 2H), 7.61 – 7.51 (m, 1H), 7.46 (ddt, *J* = 8.3, 6.7, 1.2 Hz, 2H), 7.10 (d, *J* = 1.9 Hz, 1H), 6.99 (ddt, *J* = 8.2, 2.0, 0.8 Hz, 1H), 6.74 (d, *J* = 8.1 Hz, 1H), 4.54 (t, *J* = 8.7 Hz, 2H), 4.21 (s, 2H), 3.18 (t, *J* = 8.7 Hz, 2H).

^{13}C NMR (101 MHz, $CDCl_3$) δ 198.2, 159.2, 136.7, 133.2, 129.2, 128.7, 128.7, 127.6, 126.3, 126.1, 109.4, 71.4, 44.9, 29.8.

FT-IR (neat film NaCl): 3057, 2961, 2894, 1675, 1615, 1596, 1579, 1489, 1447, 1321, 1275, 1241, 1197, 1103, 982, 941, 926, 804, 750, 689, 591, 518 cm^{-1}.

HR-MS (ESI) m/z: [M+H]+ Calculated for $C_{16}H_{15}O_2$ 239.1067; Found 237.1070.

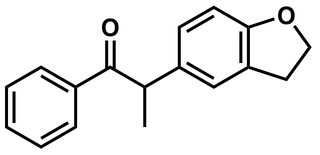

2-(2,3-dihydrobenzofuran-5-yl)-1-phenylpropan-1-one (SI-22)

To a flame dried flask was added **SI-21** (600 mg, 1 equivalent, 2.52 mmol) followed by THF (5 mL, 0.5 M) and cooled to 0 ºC. Then, a solution of KOtBu (367 mg, 1.3 equivalents, 3.27 mmol) in THF (3 ml, 1.1 M) was added dropwise. The reaction was allowed to stir for 20 minutes, and

then iodomethane (0.2 mL, 1.3 equivalents, 3.27 mmol) was added dropwise. The reaction was allowed to warm to room temperature and stirred until starting material was consumed as determined by TLC (15% diethyl ether/hexanes). Then, 2M HCl was added and the reaction was extracted EtOAc 3x. The combined organics were washed with water, brine, and then dried of MgSO4. The crude mixture was purified via column chromatography 3% diethyl ether/pentanes to afford a white solid **SI-22**, 0.380 g, 60% yield.

^{1}H NMR (400 MHz, $CDCl_3$) δ 8.01 – 7.92 (m, 2H), 7.50 – 7.44 (m, 1H), 7.42 – 7.34 (m, 2H), 7.10 (t, *J* = 1.5 Hz, 1H), 7.03 (ddd, *J* = 8.2, 2.0, 1.0 Hz, 1H), 6.70 (d, *J* = 8.2 Hz, 1H), 4.63 (q, *J* = 6.8 Hz, 1H), 4.51 (td, *J* = 8.7, 1.2 Hz, 2H), 3.14 (td, *J* = 8.6, 3.0 Hz, 2H), 1.50 (d, *J* = 6.9 Hz, 3H).

^{13}C NMR (101 MHz, $CDCl_3$) δ 200.7, 159.2, 136.6, 133.5, 132.8, 128.8, 128.5, 127.9, 127.7, 124.2, 109.6, 71.4, 47.3, 29.8, 19.8.

FT-IR (neat film NaCl): 3059, 2972, 2929, 2895, 1679, 1596, 1490, 1448, 1371, 1341, 1234, 1107, 1002, 982, 957, 944, 811, 739, 693 cm^{-1}.

HR-MS (ESI) m/z: [M+H]+ Calculated for $C_{17}H_{17}O_2$ 253.1223; Found 253.1214.

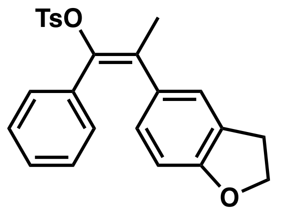

(*E*)-2-(2,3-dihydrobenzofuran-5-yl)-1-phenylprop-1-en-1-yl 4-methylbenzenesulfonate (SI-23) was prepared according to known literature procedure for similar vinyl tosylates from **SI-22**: to a flame dried Schlenk flask was added LiHMDS (2.8 g, 2 equivalents, 16.6 mmol) inside of a glovebox. The Schlenk flask was capped, removed, and anhydrous PhMe (18.5 mL, 0.9 M) was added followed by DMEA (2.1 mL, 2 equivalents, 16.6 mmol). To another flame dried flask was added **SI-22** (2.1 g, 1 equivalent, 8.3 mmol) followed by anhydrous PhMe (8.3 mL, 1.0 M). The ketone solution was then added dropwise to the LiHMDS solution at room temperature. The reaction was allowed to stir for 20 minutes. To another flame dried flask was added Ts_2O (5.4 g, 2 equivalents, 16.6 mmol) with anhydrous DCM (42 mL, 0.4 M). The Ts_2O solution was then added dropwise to the enolate solution with vigorous stirring. *Note: the solution becomes very thick.* The reaction was monitored by TLC (20% diethyl ether/hexanes), and after 1 hour it was complete. A few mLs of 1M NaOH was then added, and the solution became homogenous. Additional water was added, and then reaction was extracted 3x with diethyl ether. The combined organics were then dried over Na_2SO_4 and the crude mixture was concentrated in vacuo. The

material was purified by column chromatography (15% diethyl ether/hexanes) to afford pure **SI-23** as a white solid (2.0 g, 59% yield).

^{1}H NMR (400 MHz, $CDCl_3$) δ 7.55 – 7.43 (m, 2H), 7.11 – 7.06 (m, 2H), 7.04 – 6.97 (m, 1H), 6.95 – 6.90 (m, 4H), 6.89 (q, *J* = 1.3 Hz, 1H), 6.75 (ddd, *J* = 8.3, 1.9, 0.9 Hz, 1H), 6.55 (d, *J* = 8.2 Hz, 1H), 4.51 (t, *J* = 8.7 Hz, 2H), 3.06 (t, *J* = 8.7 Hz, 2H), 2.35 (s, 3H), 2.18 (s, 3H).

^{13}C NMR (101 MHz, $CDCl_3$) δ 159.3, 144.4, 142.8, 134.5, 134.3, 132.1, 130.9, 129.9, 129.3, 128.9, 128.1, 127.5, 127.0, 125.4, 109.0, 71.4, 29.6, 21.6, 20.2.

FT-IR (neat film NaCl): 3055, 2919, 2858, 1609, 1598, 1490, 1444, 1366, 1236, 1189, 1176, 1038, 972, 943, 834, 773, 698, 676, 559 cm^{-1}.

HR-MS (ESI) m/z: [M+K]+ Calculated for $C_{24}H_{26}NO_4S$ 424.1577; Found 424.1577.

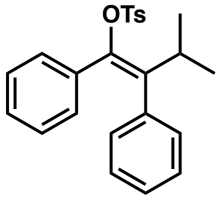

(*E*)-3-methyl-1,2-diphenylbut-1-en-1-yl 4-methylbenzenesulfonate (SI-24) was prepared according to literature procedure and matched the NMR data in the literature.[2]

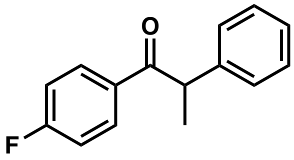

1-(4-fluorophenyl)-2-phenylpropan-1-one (SI–25)

To a flame dried flask, commercially available 1-(4-fluorophenyl)-2-phenylethan-1-one (5.00 g, 1 equivalent, 23.3 mmol) followed by THF (47 mL, 0.5 M) and cooled to 0 ºC. Then, a solution of KOtBu (3.1 g, 1.2 equivalents, 28 mmol) in THF (25 ml, 1.1 M) was added dropwise. The reaction was allowed to stir for 20 minutes, and then iodomethane (2.9 mL, 2.0 equivalents, 46.7 mmol) was added dropwise. The reaction was allowed to warm to room temperature and stirred until starting material was consumed as determined by TLC (5% diethyl ether/hexanes). Then, 2M HCl was added and the reaction was extracted EtOAc 3x. The combined organics were washed with water, brine, and then dried of MgSO4. The crude mixture was purified via column chromatography 3% diethyl ether/pentanes to afford **SI-25** (3.3 g, 62% yield. Spectra matched the NMR data in the literature.[6]

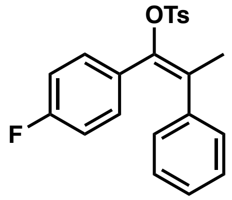

(*E*)-1-(4-fluorophenyl)-2-phenylprop-1-en-1-yl 4-methylbenzenesulfonate (SI-26) was prepared according to known literature procedure for similar vinyl tosylates from **SI–25**: to a flame dried Schlenk flask was added LiHMDS (733 mg, 2 equivalents, 4.38 mmol) inside of a glovebox. The Schlenk flask was capped, removed, and anhydrous PhMe (4.9 mL, 0.9 M) was added followed by DMEA (0.48 mL, 2 equivalents, 4.38 mmol). To another flame dried flask was added **SI-25** (500 mg, 1 equivalent, 2.2 mmol) followed by anhydrous PhMe (2.2 mL, 1.0 M). The ketone solution was then added dropwise to the LiHMDS solution at room temperature. The reaction was allowed to stir for 20 minutes. To another flame dried flask was added Ts_2O (1.43 g, 2 equivalents, 4.38 mmol) with anhydrous DCM (11 mL, 0.4 M). The Ts_2O solution was then added dropwise to the enolate solution with vigorous stirring. *Note: the solution becomes very thick.* The reaction was monitored by TLC (15% diethyl ether/hexanes), and after 1 hour it was complete. A few mLs of 1M NaOH was then added, and the solution became homogenous. Additional water was added, and then reaction was extracted 3x with diethyl ether. The combined organics were then dried over Na_2SO_4 and the crude mixture was concentrated in vacuo. The material was purified by column chromatography (8% diethyl ether/hexanes) to afford pure **SI-26** as a white solid (500 mg, 60% yield).

^{1}H NMR (400 MHz, $CDCl_3$) δ 7.59 – 7.45 (m, 2H), 7.21 – 7.11 (m, 5H), 7.05 – 6.98 (m, 2H), 6.93 – 6.84 (m, 2H), 6.66 – 6.56 (m, 2H), 2.38 (s, 3H), 2.19 (s, 3H).

^{13}C NMR (101 MHz, $CDCl_3$) δ 162.1 (d, *J* = 248.6 Hz), 144.7, 142.4, 139.8, 134.4, 131.7 (d, *J* = 8.2 Hz), 131.3, 130.2 (d, *J* = 3.4 Hz), 129.4, 128.8, 128.4, 128.1, 127.3, 114.6 (d, *J* = 21.7 Hz), 21.7, 20.0.

^{19}F NMR (376 MHz, $CDCl_3$) δ -112.9.

FT-IR (neat film NaCl): 3056, 3030, 2922, 1599, 1507, 1442, 1369, 1230, 1190, 1176, 1084, 1041, 969, 857, 840, 806, 768, 758, 700, 670, 581, 558, 545 cm^{-1}.

HR-MS (ESI) m/z: [M+Na]+ Calculated for $C_{22}H_{19}FNaO_3S$ 405.0931; Found 405.0934.

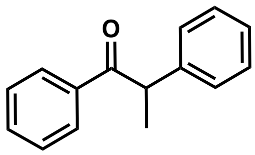

1,2-diphenylpropan-1-one (SI–27)

To a flame dried flask, commercially available 1,2-diphenylethan-1-one (3.0 g, 1 equivalent, 15.3 mmol) followed by THF (30 mL, 0.51 M) and cooled to 0 ºC. Then, a solution of KOtBu (1.89 g, 1.1 equivalents, 16.8 mmol) in THF (25 ml, 0.67 M) was added dropwise. The reaction was allowed to stir for 20 minutes, and then iodomethane (1.0 mL, 1.10 equivalents, 16.8 mmol) was added dropwise. The reaction was allowed to warm to room temperature and stirred until starting material was consumed as determined by TLC (5% diethyl ether/hexanes). Then, 2M HCl was added and the reaction was extracted EtOAc 3x. The combined organics were washed with water, brine, and then dried of MgSO4. The crude mixture was purified via column chromatography 3% diethyl ether/pentanes to afford **SI-27** (1.78 g, 55% yield). Spectra matched the NMR data in the literature.[6]

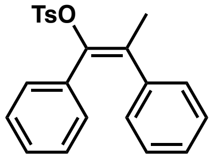

(*E*)-1,2-diphenylprop-1-en-1-yl 4-methylbenzenesulfonate (SI-28) was prepared according to known literature procedures from **SI-27** and spectra matched reported literature.[3]

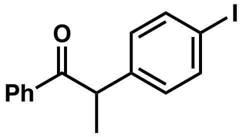

2-(4-iodophenyl)-1-phenylpropan-1-one (SI-29)

To a flame dried flask, 2-(4-iodophenyl)-1-phenylethan-1-one (1.90 g, 1 equivalent, 5.90 mmol) followed by THF (11.8 mL, 0.5 M) and cooled to 0 ºC. Then, a solution of KOtBu (860 mg, 1.3 equivalents, 7.67 mmol) in THF (7.7 ml, 1.0 M) was added dropwise. The reaction was allowed to stir for 20 minutes, and then iodomethane (0.48 mL, 1.3 equivalents, 7.67 mmol) was added dropwise. The reaction was allowed to warm to room temperature and stirred until starting material was consumed as determined by TLC (10% diethyl ether/hexanes). Then, 2M HCl was added and the reaction was extracted EtOAc 3x. The combined organics were washed with water, brine,

and then dried of MgSO4. The crude mixture was purified via column chromatography 8% diethyl ether/pentanes to afford **SI-29** as a solid (1.2 g, 61% yield. Spectra matched reported literature.[7]

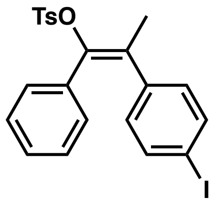

(*E*)-2-(4-iodophenyl)-1-phenylprop-1-en-1-yl 4-methylbenzenesulfonate (SI-30) was prepared according to known literature procedure for similar vinyl tosylates from **SI-29:** to a flame dried Schlenk flask was added LiHMDS (398 mg, 2 equivalents, 2.38 mmol) inside of a glovebox. The Schlenk flask was capped, removed, and anhydrous PhMe (2.6 mL, 0.91 M) was added followed by DMEA (0.26 mL, 2 equivalents, 2.38 mmol). To another flame dried flask was added **SI-29** (400 mg, 1 equivalent, 1.2 mmol) followed by anhydrous PhMe (1.2 mL, 1.0 M). The ketone solution was then added dropwise to the LiHMDS solution at room temperature. The reaction was allowed to stir for 20 minutes. To another flame dried flask was added Ts_2O (0.78 g, 2 equivalents, 2.38 mmol) with anhydrous DCM (6 mL, 0.4 M). The Ts_2O solution was then added dropwise to the enolate solution with vigorous stirring. *Note: the solution becomes very thick.* The reaction was monitored by TLC (15% diethyl ether/hexanes), and after 1 hour it was complete. A few mLs of 1M NaOH was then added, and the solution became homogenous. Additional water was added, and then reaction was extracted 3x with diethyl ether. The combined organics were then dried over Na_2SO_4 and the crude mixture was concentrated in vacuo. The material was purified by column chromatography (10% diethyl ether/hexanes) to afford pure **SI-30** as a white solid (280 mg, 48% yield).

^{1}H NMR (400 MHz, $CDCl_3$) δ 7.52 – 7.42 (m, 4H), 7.11 – 7.00 (m, 3H), 6.99 – 6.86 (m, 4H), 6.82 – 6.74 (m, 2H), 2.35 (s, 3H), 2.19 (s, 3H).

^{13}C NMR (101 MHz, C_6D_6) δ 144.6, 143.8, 139.6, 137.4, 134.4, 133.6, 130.8, 129.9, 129.4, 128.1, 127.8, 92.8, 21.7, 19.7.

FT-IR (neat film NaCl): 2920, 1598, 1484, 1369, 1189, 1176, 1041, 969, 850, 776, 709, 581, 558 cm^{-1}.

HR-MS (ESI) m/z: [M•]+ Calculated for $C_{22}H_{19}INaO_3S^+$: 512.9992; Found 513.0000.

4- Preparation of Silyl Ethers

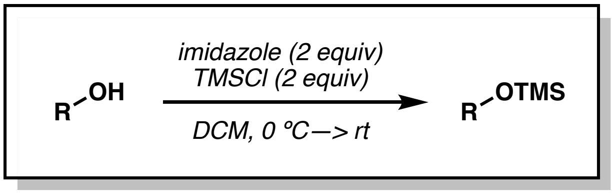

Silyl ethers were prepared according to reported procedure.[8] To a flame-dried flask was added solid imidazole (20.0 mmol, 2 equivalents) followed by dry DCM solvent (3.33 mL, 6 M). While under N_2 atmosphere, alcohol was added neat (10.0 mmol, 1 equivalent) then cooled to 0 ºC. Freshly distilled TMS–Cl (20.0 mmol, 2 equivalents) was added slowly dropwise while at 0 ºC, and the stirring mixture was allowed to warm to room temperature slowly overnight. The next morning, the reaction was quenched with water, then the DCM layer was separated out. The aqueous layer was washed once more with pentane, and the combined organics were washed with brine then filtered through a short pad of silica gel. The filtrate was concentrated cold (0 ºC) and purified by either distillation or silica column chromatography as specified below. All pure silyl ethers were cycled into a nitrogen-filled glovebox and dried further by passing the neat material through activated neutral alumina.

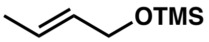

(*E*)-(but-2-en-1-yloxy)trimethylsilane (SI-31)

Prepared according to above procedure on 3.8 g (53.00 mmol) scale of starting commercially available alcohol. The product was purified via silica gel chromatography (2% diethyl ether in pentane, visualized by $KMnO_4$ stain) and concentrated at 0 ºC to obtain pure silyl ether as a colorless oil, 4.8 g (53 % yield). Spectra matched reported literature.[9]

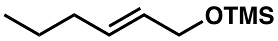

(*E*)-(hex-2-en-1-yloxy)trimethylsilane (SI-32)

Prepared according to above procedure on 2.00 g (20.00 mmol) scale of starting commercially available alcohol. The product was purified via silica gel chromatography (2% diethyl ether in pentane, visualized by $KMnO_4$ stain) and concentrated at 0 ºC to obtain pure silyl ether as a colorless oil, 2.50 g (72% yield). Spectra matched reported literature.[9]

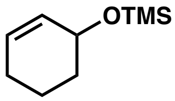

(cyclohex-2-en-1-yloxy)trimethylsilane (SI-33)

Prepared according to above procedure on 1.00 g (10.20 mmol) scale of starting commercially available alcohol. The product was purified via silica gel chromatography (2% diethyl ether in pentane, visualized by $KMnO_4$ stain) and concentrated at 0 ºC to obtain pure silyl ether as a colorless oil, 1.40 g (81 % yield). Spectra matched reported literature.[10]

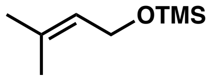

trimethyl((3-methylbut-2-en-1-yl)oxy)silane (SI-34)

Prepared according to above procedure on 3.40 g (39.00 mmol) scale of starting commercially available alcohol. The product was purified via silica gel chromatography (2% diethyl ether in pentane, visualized by $KMnO_4$ stain) and concentrated at 0 ºC to obtain pure silyl ether as a colorless oil, 3.20 g (52% yield). Spectra matched reported literature.[9]

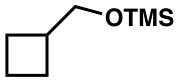

(cyclobutylmethoxy)trimethylsilane (SI-35)

Prepared according to above procedure on 2.50 g (29.00 mmol) scale of starting commercially available alcohol. The product was purified by Kugelrohr distillation (60 ºC under 1 atm N_2) to obtain pure silyl ether as a colorless oil, 3.15 g (69% yield).

^{1}H NMR (400 MHz, $CDCl_3$) δ 3.53 (d, *J* = 6.8 Hz, 2H), 2.54 – 2.38 (m, 1H), 2.07 – 1.95 (m, 2H), 1.94 – 1.75 (m, 2H), 1.75 – 1.60 (m, 2H).

^{13}C NMR (101 MHz, $CDCl_3$) δ 67.2, 37.4, 24.7, 18.4, -0.2.

FT-IR (neat film NaCl): 2956, 1249, 1079, 1027, 871, 836, 745 cm^{-1}.

HR-MS (FI) m/z: [M•]+ Calculated for $C_8H_{18}OSi$: 158.1127; Found 158.1135

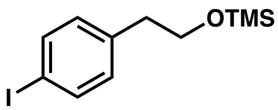

(4-iodophenethoxy)trimethylsilane (SI-36)

Prepared according to above procedure on 1.00 g (4.03 mmol) scale of starting commercially available alcohol. The product was purified via silica gel chromatography (2.5% diethyl ether in hexanes) to obtain pure silyl ether as a colorless oil, 1.26 g (97% yield).

^{1}H NMR (400 MHz, $CDCl_3$) δ 7.64 – 7.56 (m, 2H), 7.00 – 6.92 (m, 2H), 3.74 (td, *J* = 7.0, 0.8 Hz, 2H), 2.76 (t, *J* = 7.0 Hz, 2H), 0.06 (d, *J* = 0.9 Hz, 9H).

^{13}C NMR (101 MHz, $CDCl_3$) δ 138.9, 137.4, 131.3, 91.4, 63.5, 39.0, -0.4.

FT-IR (neat film NaCl): 2953, 1484, 1399, 1249, 1090, 1006, 879, 839, 809, 746 cm^{-1}.

HR-MS (FI) m/z: [M•]+ Calculated for $C_{11}H_{17}OSiI$: 320.0093; Found 320.0115.

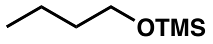

***n*-butoxytrimethylsilane (SI-37)**

Prepared according to above procedure on 2.22 g (30.00 mmol) scale of starting commercially available alcohol. The product was purified via silica gel chromatography (3% diethyl ether in pentane, visualized by I_2) and concentrated at 0 ºC to obtain pure silyl ether as a colorless oil, 2.80 g (64% yield). Spectra matched reported literature.[12]

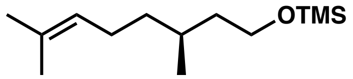

(*S*)-((3,7-dimethyloct-6-en-1-yl)oxy)trimethylsilane (SI-38)

Prepared according to above procedure on 2.50 g (16.00 mmol) scale of starting commercially available alcohol. The product was purified via distillation (160 ºC at 20 Torr) and concentrated at 0 ºC to obtain pure silyl ether as a colorless oil, 3.51 g (96% yield). Spectra matched reported literature.[13]

5- Catalytic Claisen Cascade Coupling Reaction

5.1 Reaction Optimization

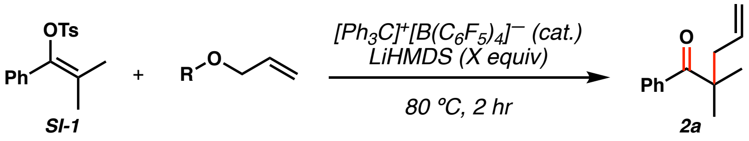

Entry	R	Ether	Catalyst	LiHMDS	Yield
1	*Et*	*2.0 equiv*	*10 mol%*	*1.5 equiv*	*41%*
2	*allyl*	*2.0 equiv*	*10 mol%*	*1.5 equiv*	*58%*
3	*TIPS*	*2.0 equiv*	*10 mol%*	*1.5 equiv*	*0%*
4	*TES*	*2.0 equiv*	*10 mol%*	*1.5 equiv*	*56%*
5	*TMS*	*2.0 equiv*	*10 mol%*	*1.5 equiv*	*78%*
6	*TMS*	*1.5 equiv*	*10 mol%*	*1.5 equiv*	*44%*
7	*TMS*	*2.0 equiv*	*10 mol%*	*2.5 equiv*	*87%*
8	*TMS*	*2.0 equiv*	*5 mol%*	*2.5 equiv*	*59%*
9	*TMS*	*2.0 equiv*	*0 mol%*	*2.5 equiv*	*0%*
10	*TMS*	*2.0 equiv*	*10 mol%*	*0 equiv*	*trace*

NMR yields determined using as an internal standard (freshly prepared nitromethane solution in $CDCl_3$). Reactions were run on 0.05 mmol scale.

5.2 Scope Studies and Product Characterization

Procedure A:

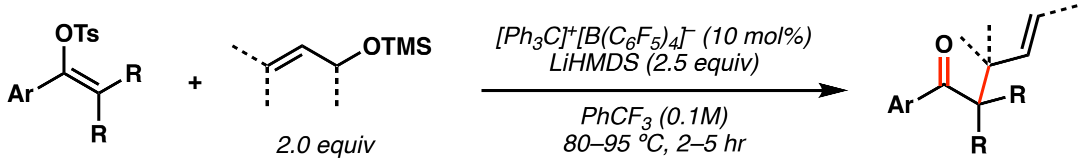

Procedure B:

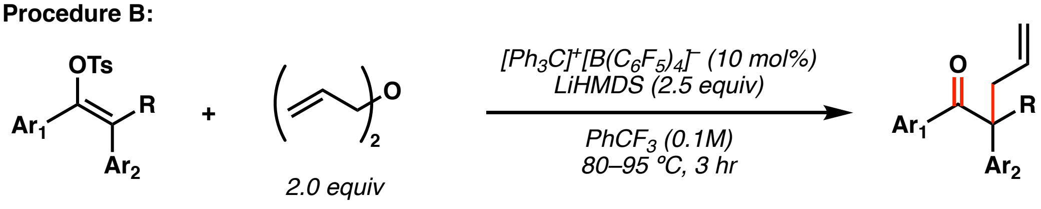

General Procedure A: All catalytic Claisen cascade coupling reactions were conducted in a well-maintained glove box (O_2, H_2O <0.5 ppm) on 0.2 mmol scale (1 equivalent of vinyl tosylate substrate). To a dram vial equipped with a magnetic stir bar was added $[Ph_3C]^+[B(C_6F_5)_4]^-$ catalyst (0.02 mmol, 10 mol%), followed by LiHMDS (0.5 mmol, 2.5 equivalents), followed by trifluorotoluene solvent (2 mL, 0.1 M). To this mixture was then added neat silyl allyl ether (0.4 mmol, 2.0 equivalents) followed by solid vinyl tosylate (0.2 mmol, 1 equivalent). The reaction was

then sealed with a Teflon cap and heated in a reaction block for 2–5 hours depending on the substrate (reaction temperatures were typically 80 ºC, but for a few substrates higher reaction temperatures were required as indicated below). The reactions were monitored by TLC, typically using 10% ethyl acetate in hexanes for the mobile phase. Once the reaction was completed, the vial was removed from the glovebox. The reaction was diluted with diethyl ether, filtered through a pad of silica, and concentrated in vacuo. The crude material was purified by column chromatography, (typically 2–10% diethyl ether/hexanes, depending on the product polarity) then dried on high vacuum to obtain material that is pure by ^{1}H NMR.

General Procedure B: A slightly modified procedure was followed when substrates contained an additional aryl group in the α-position. While Procedure A still worked for this substrate class, it was found through optimization that diallyl ethers provided improved yields over silyl ally ethers. Therefore, the only modification for General Procedure B as compared to General Procedure A is that diallyl ether is used instead of TMS allyl ether, according to the graphic above.

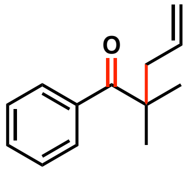

2,2-dimethyl-1-phenylpent-4-en-1-one (2a)

Following the *General Procedure A*. The reaction was complete after 2 hr. The product was purified by silica gel column chromatography (2% diethyl ether/hexanes) to obtain 31.7 mg of a pale yellow oil (84% yield). The spectra matched reported literature.[14]

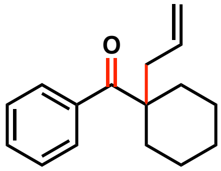

(1-allylcyclohexyl)(phenyl)methanone (2b)

Following the *General Procedure A*. The reaction was complete after 2 hr. The product was purified by silica gel column chromatography (3% diethyl ether/hexanes) to obtain 35.4 mg of a pale yellow oil (78% yield). The spectra matched reported literature.[15]

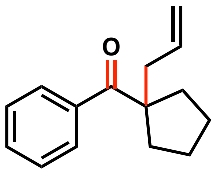

(1-allylcyclopentyl)(phenyl)methanone (2c)

Following the *General Procedure A*. The reaction was complete after 2 hr. The product was purified by silica gel column chromatography (3% diethyl ether/hexanes) to obtain 26.0 mg of a pale yellow oil (61% yield).

^{1}H NMR (400 MHz, $CDCl_3$) δ 7.89 – 7.82 (m, 2H), 7.52 – 7.46 (m, 1H), 7.45 – 7.37 (m, 2H), 5.59 (ddt, *J* = 17.3, 10.1, 7.2 Hz, 1H), 4.96 – 4.82 (m, 2H), 2.60 (dt, *J* = 7.2, 1.3 Hz, 2H), 2.32 (dddd, *J* = 14.5, 7.5, 4.0, 1.5 Hz, 2H), 1.78 (dddd, *J* = 13.0, 7.2, 3.6, 1.2 Hz, 2H), 1.72 – 1.61 (m, 4H).

^{13}C NMR (101 MHz, $CDCl_3$) δ 205.7, 137.2, 134.4, 131.8, 128.9, 128.3, 117.8, 58.9, 44.4, 35.9, 25.7.

FT-IR (neat film NaCl): 3074, 2953, 2868, 1672, 1597, 1578, 1446, 1275, 1216, 1179, 1009, 918, 716, 693, 430 cm^{-1}.

HR-MS (ESI) m/z: [M+H]+ Calculated for $C_{15}H_{19}O$ 215.1430; Found 215.1428.

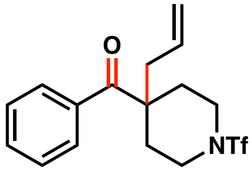

(4-allyl-1-((trifluoromethyl)sulfonyl)piperidin-4-yl)(phenyl)methanone (2d)

Following the *General Procedure A* with slight modification. Reaction was ran at 0.05M with 20 mol% $[Ph_3C]^+[B(C_6F_5)_4]^-$ and 1.5 equivalents of LiHMDS at 100 ºC for 24 hr. The product was purified by silica gel column chromatography (10% diethyl ether/hexanes then 40% DCM/hexanes) to obtain 38.0 mg of clear oil (53% yield).

^{1}H NMR (400 MHz, $CDCl_3$) δ 7.76 – 7.69 (m, 2H), 7.57 – 7.51 (m, 1H), 7.48 – 7.42 (m, 2H), 5.65 (ddt, *J* = 16.8, 10.2, 7.4 Hz, 1H), 5.15 – 5.06 (m, 2H), 3.74 (dd, *J* = 12.9, 4.4 Hz, 2H), 3.09 (broad singlet, 2H), 2.66 (dt, *J* = 7.4, 1.2 Hz, 2H), 2.49 (d, *J* = 14.0 Hz, 2H), 1.71 (ddd, *J* = 14.1, 11.5, 4.2 Hz, 2H).

^{13}C NMR (101 MHz, $CDCl_3$) δ 205.3, 138.1, 132.1, 131.5, 128.8, 127.9, 120.2 (q, *J* = 323.1 Hz), 119.6, 50.3, 44.2, 33.8.

^{19}F NMR (282 MHz, $CDCl_3$) δ -75.63.

FT-IR (neat film NaCl): 3078, 2977, 2927, 2886, 1672, 1597, 1449, 1387, 1342, 1226, 1184, 1135, 1053, 1004, 945, 765, 705, 591, 495 cm^{-1}.

HR-MS (ESI) m/z: [M+H]+ Calculated for $C_{16}H_{19}F_3NO_3S$ 362.1032; Found 362.1031.

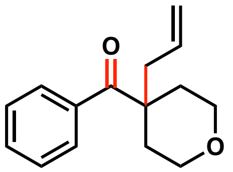

(4-allyltetrahydro-2*H*-pyran-4-yl)(phenyl)methanone (2e)

Following the *General Procedure A* with slight modification. Reaction was run at 0.05M with 3.0 equivalents of LiHMDS. The reaction was complete after 12 hr. The product was purified by silica gel column chromatography (15% diethyl ether/hexanes) to obtain 22.3 mg of a pale yellow oil (50% yield).

^{1}H NMR (400 MHz, $CDCl_3$) δ 7.72 – 7.66 (m, 2H), 7.52 – 7.46 (m, 1H), 7.45 – 7.39 (m, 2H), 5.69 (ddt, *J* = 16.8, 10.2, 7.4 Hz, 1H), 5.11 – 5.03 (m, 2H), 3.77 (dt, *J* = 11.9, 4.2 Hz, 2H), 3.44 (ddd, *J* = 11.9, 10.1, 2.6 Hz, 2H), 2.65 (dt, *J* = 7.4, 1.2 Hz, 2H), 2.33 – 2.27 (m, 2H), 1.71 (ddd, *J* = 14.1, 10.2, 4.1 Hz, 2H).

^{13}C NMR (101 MHz, $CDCl_3$) δ 206.9, 139.0, 132.5, 131.4, 128.5, 127.8, 118.9, 65.1, 50.2, 43.6, 34.5.

FT-IR (neat film NaCl): 3075, 2957, 2922, 2850, 1672, 1447, 1299, 1218, 1107, 1032, 976, 919, 793, 733, 699, 553 cm^{-1}.

HR-MS (ESI) m/z: [M+H]+ Calculated for $C_{15}H_{19}O_2$ 231.1380; Found 231.1375.

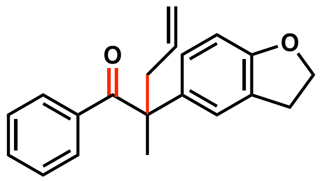

2-(2,3-dihydrobenzofuran-5-yl)-2-methyl-1-phenylpent-4-en-1-one (2f)

Following the *General Procedure B*. The reaction was complete after 3 hr. The product was purified by silica gel column chromatography (7% diethyl ether/hexanes) to obtain 41.2 mg of a pale yellow oil (71% yield).

^{1}H NMR (400 MHz, $CDCl_3$) δ 7.53 – 7.42 (m, 2H), 7.41 – 7.34 (m, 1H), 7.23 (ddt, *J* = 7.9, 6.7, 1.2 Hz, 2H), 7.14 – 7.01 (m, 2H), 6.78 (d, *J* = 8.3 Hz, 1H), 5.52 (dddd, *J* = 17.1, 10.2, 7.7, 6.9 Hz, 1H),

5.04 – 4.82 (m, 2H), 4.58 (t, *J* = 8.7 Hz, 2H), 3.24 – 3.14 (m, 2H), 2.79 (ddt, *J* = 13.7, 7.6, 1.1 Hz, 1H), 2.71 (ddt, *J* = 13.7, 6.8, 1.3 Hz, 1H), 1.52 (s, 3H).

^{13}C NMR (101 MHz, $CDCl_3$) δ 203.7, 159.2, 137.2, 135.4, 134.4, 131.6, 129.6, 128.1, 127.9, 125.8, 123.1, 118.3, 109.6, 71.5, 53.8, 44.9, 29.9, 23.9.

FT-IR (neat film NaCl): 3072, 2979, 2941, 2361, 2342, 2292, 2252, 1674, 1639, 1614, 1596, 1493, 1445, 1374, 1235, 1183, 1110, 1039, 982, 972, 943, 918, 822, 798, 735, 716, 695, 660, 626 cm^{-1}.

HR-MS (ESI) m/z: [M+H]+ Calculated for $C_{20}H_{21}O_2$ 293.1536; Found 293.1543.

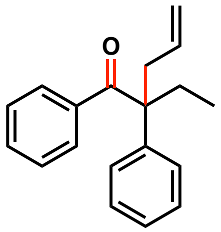

2-ethyl-1,2-diphenylpent-4-en-1-one (2g)

Following the *General Procedure B*. The reaction was complete after 3 hr. The product was purified by silica gel column chromatography (3% diethyl ether/hexanes) to obtain 39.6 mg of a pale yellow oil (75% yield).

^{1}H NMR (400 MHz, $CDCl_3$) δ 7.44 – 7.39 (m, 2H), 7.39 – 7.32 (m, 3H), 7.28 (ddd, *J* = 5.7, 4.4, 2.6 Hz, 3H), 7.20 (td, *J* = 7.5, 1.1 Hz, 2H), 5.38 (dddd, *J* = 16.9, 10.3, 8.1, 6.5 Hz, 1H), 4.99 – 4.85 (m, 2H), 2.90 – 2.77 (m, 2H), 2.21 – 2.10 (m, 2H), 0.69 (t, *J* = 7.4 Hz, 3H).

^{13}C NMR (101 MHz, $CDCl_3$) δ 203.3, 142.7, 137.3, 133.6, 131.7, 129.5, 129.0, 128.1, 127.1, 127.0, 118.3, 58.2, 39.3, 26.9, 8.0.

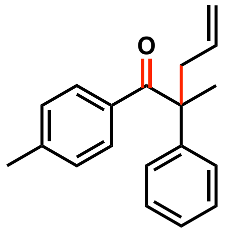

2-methyl-2-phenyl-1-(*p*-tolyl)pent-4-en-1-one (2h)

Following the *General Procedure B*. The reaction was complete after 3 hr. The product was purified by silica gel column chromatography (3% diethyl ether/hexanes) to obtain 35.0 mg of a pale yellow oil (66% yield).

^{1}H NMR (400 MHz, CDCl$_3$) δ 7.42 – 7.31 (m, 4H), 7.30 – 7.26 (m, 3H), 7.07 – 6.97 (m, 2H), 5.51 (dddd, *J* = 17.0, 10.2, 7.6, 6.9 Hz, 1H), 5.00 – 4.90 (m, 2H), 2.83 (ddt, *J* = 13.8, 7.7, 1.1 Hz, 1H), 2.74 (ddt, *J* = 13.7, 6.9, 1.3 Hz, 1H), 2.29 (s, 3H), 1.57 (s, 3H).
^{13}C NMR (101 MHz, CDCl$_3$) δ 202.7, 144.0, 142.4, 134.2, 133.9, 129.9, 129.0, 128.9, 128.7, 126.9, 126.3, 118.4, 54.3, 44.9, 23.9, 21.5.
FT-IR (neat film NaCl): 3061, 3026, 2977, 2924, 1672, 1606, 1496, 1446, 1376, 1241, 1183, 966, 972, 916, 830, 745, 702, 597 cm^{-1}.
HR-MS (ESI) m/z: [M+H]+ Calculated for $C_{19}H_{21}O$ 265.1587; Found 265.1588.

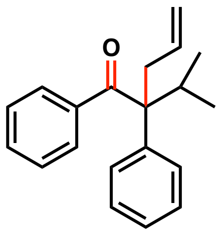

2-isopropyl-1,2-diphenylpent-4-en-1-one (2i)
Following the *General Procedure B* at 95 ºC. The reaction was complete after 3 hr. The product was purified by silica gel column chromatography (3% diethyl ether/hexanes) to obtain 23.0 mg of a pale yellow oil (41% yield).
^{1}H NMR (400 MHz, CDCl$_3$) δ 7.51 – 7.40 (m, 2H), 7.37 – 7.26 (m, 4H), 7.23 – 7.12 (m, 4H), 5.60 (dddd, *J* = 17.1, 10.2, 7.6, 6.9 Hz, 1H), 5.01 – 4.78 (m, 2H), 3.22 (ddt, *J* = 14.4, 7.5, 1.2 Hz, 1H), 2.89 (ddt, *J* = 14.4, 6.9, 1.5 Hz, 1H), 2.65 (hept, *J* = 6.7 Hz, 1H), 0.82 (dd, *J* = 6.7, 3.9 Hz, 6H).
^{13}C NMR (101 MHz, CDCl$_3$) δ 201.8, 139.8, 137.0, 133.4, 131.7, 130.2, 128.9, 128.0, 127.8, 127.0, 118.3, 61.8, 38.7, 30.8, 30.4, 19.7, 17.4, 15.4.
FT-IR (neat film NaCl): 3059, 3023, 2962, 2877, 1675, 1596, 1578, 1445, 1384, 1265, 1212, 1181, 916, 747, 705, 692, 647 cm^{-1}.
HR-MS (ESI) m/z: [M+H]+ Calculated for $C_{20}H_{23}O$ 279.1743; Found 279.1739.

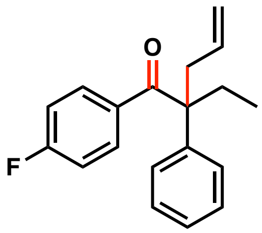

2-ethyl-1-(4-fluorophenyl)-2-phenylpent-4-en-1-one (2j)

Following the *General Procedure B*. The reaction was complete after 3 hr. The product was purified by silica gel column chromatography (3% diethyl ether/hexanes) to obtain 38.0 mg of a pale yellow oil (68% yield). The spectra matched the reported literature.[16]

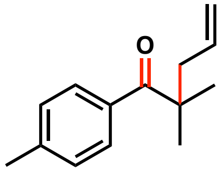

2,2-dimethyl-1-(*p*-tolyl)pent-4-en-1-one (2k)

Following the *General Procedure A*. The reaction was complete after 2 hr. The product was purified by silica gel column chromatography (3% diethyl ether/hexanes) to obtain 31.2 mg of a pale yellow oil (77% yield).

^{1}H NMR (400 MHz, $CDCl_3$) δ 7.67 – 7.57 (m, 2H), 7.24 – 7.16 (m, 2H), 5.71 (ddt, *J* = 16.7, 10.2, 7.3 Hz, 1H), 5.06 – 4.96 (m, 2H), 2.50 (dt, *J* = 7.3, 1.2 Hz, 2H), 2.38 (s, 3H), 1.32 (s, 6H).

^{13}C NMR (101 MHz, $CDCl_3$) δ 207.9, 141.6, 136.1, 134.3, 128.9, 128.3, 118.2, 47.7, 45.3, 26.0, 21.6.

FT-IR (neat film NaCl): 3076, 2977, 2927, 2873, 1671, 1640, 1608, 1468, 1386, 1251, 1171, 963, 917, 827, 755, 564 cm^{-1}.

HR-MS (ESI) m/z: [M+H]+ Calculated for $C_{14}H_{19}O$ 203.1430; Found 203.1428.

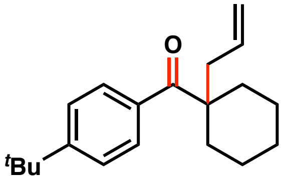

(1-allylcyclohexyl)(4-(*tert*-butyl)phenyl)methanone (2l)

Following the *General Procedure A*. The reaction was complete after 2 hr. The product was purified by silica gel column chromatography (3% diethyl ether/hexanes) to obtain 44.0 mg of a pale yellow oil (77% yield).

^{1}H NMR (400 MHz, $CDCl_3$) δ 7.65 – 7.60 (m, 2H), 7.42 – 7.36 (m, 2H), 5.70 (ddt, *J* = 15.9, 11.0, 7.4 Hz, 1H), 5.05 – 4.98 (m, 2H), 2.56 (dt, *J* = 7.4, 1.3 Hz, 2H), 2.26 – 2.18 (m, 2H), 1.58 – 1.35 (m, 6H), 1.33 (s, 9H), 1.31 – 1.25 (m, 2H).

^{13}C NMR (101 MHz, $CDCl_3$) δ 208.3, 154.2, 137.1, 133.7, 127.7, 125.1, 117.9, 52.5, 43.6, 34.9, 34.5, 31.3, 26.1, 23.0.

FT-IR (neat film NaCl): 3076, 2960, 2931, 2865, 1668, 1639, 1605, 1452, 1364, 1269, 1222, 1192, 1109, 995, 913, 844, 834, 716 cm^{-1}.

HR-MS (ESI) m/z: [M+H]+ Calculated for $C_{21}H_{31}O$ 285.2213; Found 285.2212.

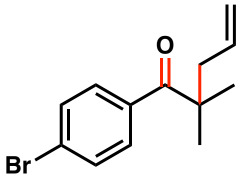

1-(4-bromophenyl)-2,2-dimethylpent-4-en-1-one (2m)

Following the *General Procedure A*. The reaction was complete after 6 hr. The product was purified by silica gel column chromatography (3% diethyl ether/hexanes) to obtain 31.2 mg of a pale yellow oil (58% yield).

^{1}H NMR (400 MHz, $CDCl_3$) δ 7.54 (s, 4H), 5.69 (ddt, *J* = 16.9, 10.2, 7.3 Hz, 1H), 5.06 – 4.97 (m, 2H), 2.47 (dt, *J* = 7.3, 1.2 Hz, 2H), 1.31 (s, 6H).

^{13}C NMR (101 MHz, $CDCl_3$) δ 207.7, 137.8, 134.0, 131.6, 129.7, 125.9, 118.6, 47.9, 45.2, 25.9.

FT-IR (neat film NaCl): 3076, 2977, 2931, 2873, 1675, 1584, 1483, 1468, 1393, 1249, 1073, 1010, 919, 836, 758 cm^{-1}.

HR-MS (FD^+) m/z: [M] Calculated for $C_{13}H_{15}BrO$ 266.0306; Found 266.0292.

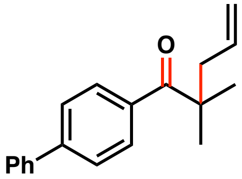

1-([1,1'-biphenyl]-4-yl)-2,2-dimethylpent-4-en-1-one (2n)

Following the *General Procedure A*. The reaction was complete after 2 hr. The product was purified by silica gel column chromatography (3% diethyl ether/hexanes) to obtain 39.2 mg of a white solid (74% yield).

^{1}H NMR (400 MHz, $CDCl_3$) δ 7.81 – 7.77 (m, 2H), 7.64 – 7.60 (m, 4H), 7.49 – 7.44 (m, 2H), 7.42 – 7.36 (m, 1H), 5.75 (ddt, *J* = 16.8, 10.3, 7.3 Hz, 1H), 5.08 – 5.00 (m, 2H), 2.53 (dt, *J* = 7.4, 1.2 Hz, 2H), 1.37 (s, 6H).

^{13}C NMR (101 MHz, $CDCl_3$) δ 208.1, 143.8, 140.2, 137.6, 134.3, 129.1, 128.7, 128.1, 127.3, 126.9, 118.3, 47.8, 45.2, 26.0.

FT-IR (neat film NaCl): 3076, 3031, 2977, 2931, 1670, 1604, 1487, 1468, 1387, 1251, 1222, 1173, 965, 918, 850, 749, 679, 415 cm^{-1}.

HR-MS (ESI) m/z: [M+H]+ Calculated for $C_{19}H_{21}O$ 265.1587; Found 265.1588.

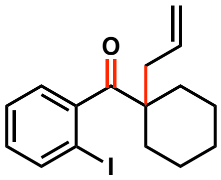

(1-allylcyclohexyl)(2-iodophenyl)methanone (2o)

Following the *General Procedure B*. The reaction was complete after 2 hr. The product was purified by silica gel column chromatography (3% diethyl ether/hexanes) to obtain 43.4 mg of a colorless oil (61% yield).

^{1}H NMR (400 MHz, $CDCl_3$) δ 7.89 (dd, *J* = 8.0, 1.1 Hz, 1H), 7.34 (td, *J* = 7.5, 1.2 Hz, 1H), 7.22 (dd, *J* = 7.7, 1.6 Hz, 1H), 7.06 (ddd, *J* = 8.0, 7.3, 1.7 Hz, 1H), 5.84 (ddt, *J* = 16.0, 11.3, 7.4 Hz, 1H), 5.14 – 5.07 (m, 2H), 2.57 (dt, *J* = 7.3, 1.2 Hz, 2H), 1.91 (ddd, *J* = 13.2, 9.0, 4.0 Hz, 2H), 1.67 – 1.60 (m, 2H), 1.56 – 1.46 (m, 5H), 1.38 – 1.29 (m, 1H).

^{13}C NMR (101 MHz, $CDCl_3$) δ 210.9, 145.5, 140.6, 134.2, 130.4, 127.2, 125.9, 118.1, 92.6, 52.2, 39.8, 33.2, 25.6, 22.1.

FT-IR (neat film NaCl): 3073, 2929, 2856, 1688, 1638, 1581, 1453, 1425, 1278, 1218, 1016, 996, 913, 765, 744, 653, 632 cm^{-1}.

HR-MS (ESI) m/z: [M+H]+ Calculated for $C_{16}H_{20}IO$ 355.0553; Found 355.0553.

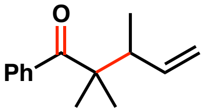

2,2,3-trimethyl-1-phenylpent-4-en-1-one (2p)

Following the *General Procedure A*. The reaction was complete after 2 hr. The product was purified by silica gel column chromatography (15–18% benzene/hexanes) to obtain 30.5 mg of a pale yellow oil (75% yield).

^{1}H NMR (400 MHz, $CDCl_3$) δ 7.66 – 7.62 (m, 2H), 7.48 – 7.43 (m, 1H), 7.42 – 7.37 (m, 2H), 5.75 (ddd, *J* = 17.0, 10.4, 8.1 Hz, 1H), 5.06 – 4.98 (m, 2H), 2.88 (dqt, *J* = 7.8, 6.8, 1.0 Hz, 1H), 1.26 (s, 3H), 1.22 (s, 3H), 0.97 (d, *J* = 6.8 Hz, 3H).

^{13}C NMR (101 MHz, $CDCl_3$) δ 209.7, 139.7, 130.8, 128.2, 127.7, 116.1, 50.9, 44.4, 24.1, 21.6, 15.4.

FT-IR (neat film NaCl): 3075, 2970, 2927, 2874, 1674, 1598, 1462, 1444, 1389, 1253, 1176, 1135, 1002, 965, 915, 714, 699 cm^{-1}.

HR-MS (ESI) m/z: [M+H]+ Calculated for $C_{14}H_{19}O$ 203.1430; Found 203.1430.

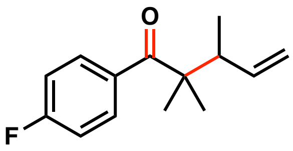

1-(4-fluorophenyl)-2,2,3-trimethylpent-4-en-1-one (2q)

Following the *General Procedure A*. The reaction was complete after 4.5 hr. The product was purified by silica gel column chromatography (3% diethyl ether/hexanes) to obtain 21.5 mg of a pale yellow oil (49% yield).

^{1}H NMR (400 MHz, $CDCl_3$) δ 7.77 – 7.70 (m, 2H), 7.11 – 7.05 (m, 2H), 5.73 (ddd, *J* = 17.1, 10.4, 8.2 Hz, 1H), 5.06 – 4.96 (m, 2H), 2.86 (dqt, *J* = 7.8, 6.8, 1.0 Hz, 1H), 1.26 (s, 3H), 1.22 (s, 3H), 0.96 (d, *J* = 6.9 Hz, 3H).

^{13}C NMR (101 MHz, $CDCl_3$) δ 207.5, 164.3 (d, *J* = 252.2 Hz), 139.5, 135.3 (d, *J* = 3.5 Hz), 130.6 (d, *J* = 8.7 Hz), 116.2, 115.3 (d, *J* = 21.4 Hz), 50.9, 44.6, 24.0, 21.8, 15.4.

^{19}F NMR (282 MHz, $CDCl_3$) δ -108.5.

FT-IR (neat film NaCl): 3076, 2974, 2935, 2876, 1674, 1600, 1506, 1463, 1236, 1158, 967, 590 cm^{-1}.

HR-MS (ESI) m/z: [M+Na]+ Calculated for $C_{14}H_{17}FNaO$ 243.1156; Found. 243.1162.

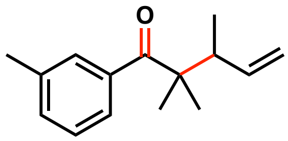

2,2,3-trimethyl-1-(*m*-tolyl)pent-4-en-1-one (2r)

Following the *General Procedure A*. The reaction was complete after 2.5 hr. The product was purified by silica gel column chromatography (3% diethyl ether/hexanes) to obtain 33.3 mg of a pale yellow oil (77% yield).

^{1}H NMR (400 MHz, $CDCl_3$) δ 7.47 – 7.39 (m, 2H), 7.29 – 7.26 (m, 2H), 5.75 (ddd, *J* = 16.9, 10.3, 8.1 Hz, 1H), 5.07 – 4.98 (m, 2H), 2.88 (dqt, *J* = 7.8, 6.8, 1.0 Hz, 1H), 2.38 (s, 3H), 1.25 (s, 3H), 1.20 (s, 3H), 0.97 (d, *J* = 6.8 Hz, 3H).

^{13}C NMR (101 MHz, $CDCl_3$) δ 210.0, 139.8, 138.0, 131.5, 128.4, 127.9, 124.6, 116.1, 50.9, 44.3, 24.2, 21.63, 21.61, 15.4.

FT-IR (neat film NaCl): 3076, 2974, 2934, 2876, 1673, 1602, 1464, 1388, 1261, 1161, 1132, 999, 975, 916, 846, 749, 698, 531 cm^{-1}.

HR-MS (ESI) m/z: [M+H]+ Calculated for $C_{15}H_{21}O$ 217.1587; Found 217.1585.

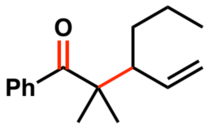

2,2-dimethyl-1-phenyl-3-vinylhexan-1-one (2s)

Following the *General Procedure A*. The reaction was complete after 2 hr. The product was purified by silica gel column chromatography (15–18% benzene/hexanes) to obtain 37.1 mg of a pale yellow oil (81% yield).

^{1}H NMR (400 MHz, $CDCl_3$) δ 7.65 – 7.60 (m, 2H), 7.47 – 7.36 (m, 3H), 5.55 (ddd, *J* = 17.0, 10.2, 9.5 Hz, 1H), 5.14 – 4.95 (m, 2H), 2.61 (td, *J* = 9.8, 3.3 Hz, 1H), 1.37 – 1.26 (m, 2H), 1.25 (s, 3H), 1.24 – 1.22 (m, 1H), 1.21 (s, 3H), 1.09 – 0.99 (m, 1H), 0.77 (t, *J* = 7.2 Hz, 3H).

^{13}C NMR (101 MHz, $CDCl_3$) δ 209.8, 139.7, 138.2, 130.7, 128.2, 127.7, 118.1, 50.9, 32.1, 25.0, 21.4, 20.9, 13.9.

FT-IR (neat film NaCl): 3074, 2959, 2932, 2872, 1674, 1597, 1466, 1444, 1387, 1250, 1177, 1001, 974, 955, 916, 699 cm^{-1}.

HR-MS (ESI) m/z: [M+H]+ Calculated for $C_{16}H_{23}O$ 231.1743; Found 231.1742.

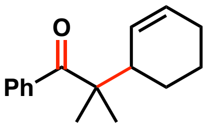

2-(cyclohex-2-en-1-yl)-2-methyl-1-phenylpropan-1-one (2t)

Following the *General Procedure A*. The reaction was complete after 4 hr. The product was purified by silica gel column chromatography (3% diethyl ether/hexanes) to obtain 28.5 mg of a pale yellow oil (62% yield), and the spectra matched the reported literature.[17]

^{1}H NMR (400 MHz, $CDCl_3$) δ 7.72 – 7.58 (m, 2H), 7.47 – 7.43 (m, 1H), 7.42 – 7.36 (m, 2H), 5.81 – 5.74 (m, 1H), 5.51 (dp, *J* = 10.2, 2.0 Hz, 1H), 2.94 – 2.81 (m, 1H), 1.96 (dpd, *J* = 11.0, 3.5, 2.3 Hz, 2H), 1.81 – 1.75 (m, 1H), 1.71 – 1.64 (m, 1H), 1.54 – 1.45 (m, 1H), 1.37 – 1.30 (m, 1H), 1.28 (s, 3H), 1.23 (s, 3H).

^{13}C NMR (101 MHz, $CDCl_3$) δ 209.8, 139.6, 130.8, 129.4, 128.2, 127.73, 127.71, 50.9, 42.5, 25.3, 24.43, 22.8, 22.6, 22.8.

6- Catalytic Vinyl Ether Synthesis

6.1 Reaction Optimization

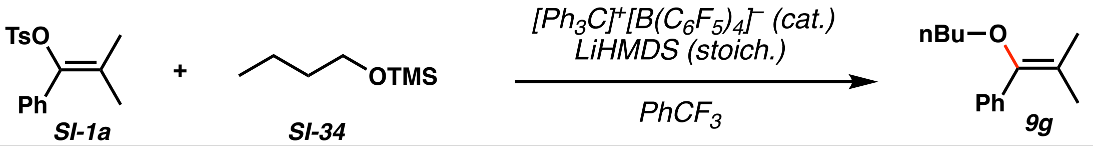

[Tosylate]	Catalyst	LiHMDS	Ether	Temp.	Time	NMR Yield
0.1 M	10 mol%	1.5 equiv	2.0 equiv	80 ºC	3 hr	76%
0.1 M	10 mol%	1.5 equiv	1.5 equiv	80 ºC	3 hr	69%
0.1 M	10 mol%	2.5 equiv	1.5 equiv	80 ºC	3 hr	75%
0.1 M	10 mol%	2.5 equiv	1.0 equiv	80 ºC	3 hr	52%
0.2 M	10 mol%	2.5 equiv	1.5 equiv	80 ºC	3 hr	65%
0.05 M	10 mol%	2.5 equiv	1.5 equiv	80 ºC	3 hr	68%
0.1 M	12 mol%	2.5 equiv	1.5 equiv	60 ºC	3 hr	71%
0.1 M	10 mol%	2.5 equiv	1.5 equiv	60 ºC	3 hr	71%
0.1 M	8 mol%	2.5 equiv	1.5 equiv	60 ºC	3 hr	72%
0.1 M	6 mol%	2.5 equiv	1.5 equiv	60 ºC	3 hr	69%
0.1 M	0 mol%	2.5 equiv	1.5 equiv	60 ºC	3 hr	<5%
0.1 M	8 mol%	0 equiv	1.5 equiv	60 ºC	3 hr	<5%
0.1 M	*8 mol%	0 equiv	1.5 equiv	60 ºC	3 hr	<5%

NMR yields determined using as an internal standard (freshly prepared nitromethane solution in $CDCl_3$). $CDCl_3$ was neutralized by storing over K_2CO_3. Reactions were run on 0.05 mmol scale.
** $[Li]^+[B(C_6F_5)_4]^-$ used as catalyst*

6.2 Scope Studies and Product Characterization

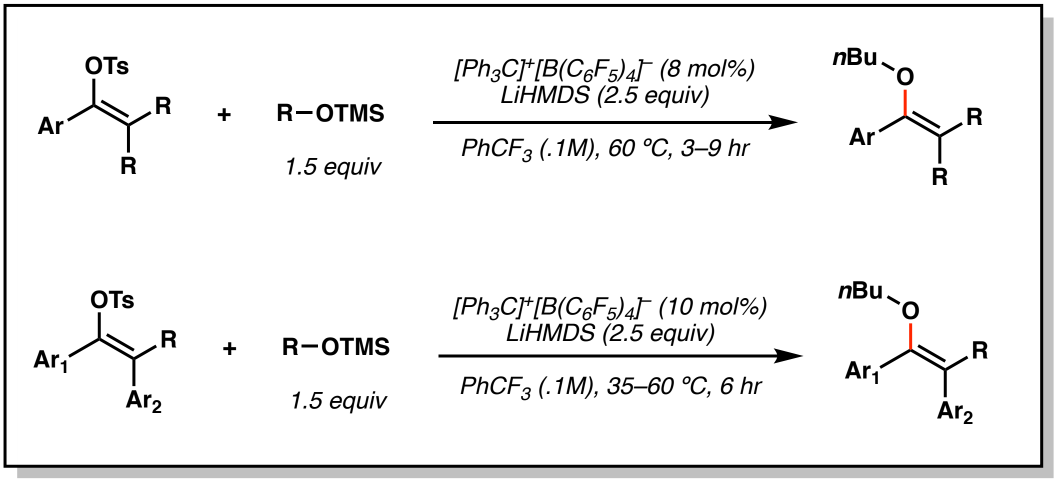

General Procedure C: All catalytic vinyl ether forming reactions were conducted in a glove box on 0.2 mmol scale (1 equivalent of vinyl tosylate substrate). To a dram vial equipped with a magnetic stir bar was added $[Ph_3C]^+[B(C_6F_5)_4]^-$ catalyst (8–10 mol%, as specified below), followed by LiHMDS (0.5 mmol, 2.5 equivalents), followed by trifluorotoluene solvent (2 mL, 0.1 M). To this mixture was then added neat silyl ether (0.3 mmol, 1.5 equivalents) followed by solid vinyl tosylate (0.2 mmol, 1 equivalent). The reaction was then sealed with a Teflon cap and heated in a reaction block for 3–9 hours depending on the substrate (reaction temperature also varies depending on substrate, see below). In general, reactions that do not produce mixtures of E/Z isomers were conducted at 60 ºC for 3 hours with 8 mol% catalyst loading, unless otherwise noted. Reactions that do produce isomers were conducted at 35 ºC for 6 hours with 10 mol% catalyst loading, unless otherwise noted (lower temperatures provided better selectivity, but required extended reaction times and slightly higher catalyst loadings). The reactions were monitored by TLC, typically using 10% ethyl acetate (+0.1% NEt_3) in hexanes for the mobile phase (vinyl ether products are typically higher in R_f than the starting vinyl tosylate). Once the reaction was completed, the vial was removed from the glovebox. The reaction was diluted with diethyl ether containing 1% triethylamine and filtered through a pad of silica gel (pushing through 1% triethylamine in diethyl ether) and concentrated in vacuo. If the reactions produces a mixture of *E/Z* isomers, an aliquot of the crude was taken for GC-FID analysis to determine *E/Z* ratio (minor isomer retention time was validated by individual preparation of authentic sample of the isomer, see section **5.3**).The crude material was purified by column chromatography, using 8–10% benzene in hexanes (+0.1% NEt_3) or 1% diethyl ether in hexanes (+0.1% NEt_3), then dried on high vacuum to obtain material that is pure by ^{1}H NMR. In cases where sample is contaminated

with higher boiling silyl ether, heat is applied *via* reaction block while under high vacuum (45–90 ºC, see below) to remove the excess silyl ether.

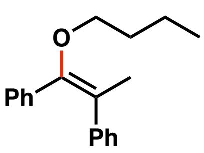

(*E*)-(1-butoxyprop-1-ene-1,2-diyl)dibenzene (8a)

Following *General Procedure C* using 10 mol% $[Ph_3C]^+[B(C_6F_5)_4]^-$ catalyst at 35 ºC for 6 hours. An aliquot of the crude material was taken for analysis of *E*/*Z* ratio by GC-FID, which was determined to be 38:1 *E*/*Z* (minor isomer retention time was confirmed by preparation of authentic material via enolate alkylation, see section **5.3**). The crude material was then purified by silica gel column chromatography (8% benzene + 0.1% triethylamine in hexanes) to obtain 28.1 mg of a colorless oil (53% yield). The major isomer was assigned as *E* on the basis of 2D ^{1}H NOESY NMR.

^{1}H NMR (400 MHz, C_6D_6) δ 7.33 – 7.24 (m, 2H), 7.13 – 7.08 (m, 2H), 7.04 – 6.87 (m, 6H), 3.49 (t, *J* = 6.5 Hz, 2H), 2.33 (s, 2H), 1.61 – 1.50 (m, 2H), 0.83 (t, *J* = 7.4 Hz, 3H).

^{13}C NMR (101 MHz, C_6D_6) δ 150.9, 142.5, 136.0, 130.1, 129.5, 127.7, 127.5, 127.3, 125.9, 120.9, 68.8, 32.2, 19.3, 18.3, 13.8.

FT-IR (neat film NaCl): 2957, 2931, 2871, 1443, 1239, 1125, 1072, 775, 698 cm $^{-1}$.

HR-MS (FD) m/z: [M$^\bullet$]+ Calculated for $C_{19}H_{22}O$: 266.1670: Found 266.1675.

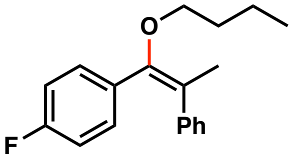

(*E*)-1-(1-butoxy-2-(cyclohexa-1,5-dien-1-yl)prop-1-en-1-yl)-4-fluorobenzene (8b)

Following *General Procedure C* using 10 mol% $[Ph_3C]^+[B(C_6F_5)_4]^-$ catalyst at 35 ºC for 9 hours. An aliquot of the crude material was taken for analysis of *E*/*Z* ratio by GC-FID, which was determined to be 62:1 *E*/*Z* (minor isomer retention time was confirmed by preparation of authentic material via enolate alkylation, see section **5.3**). The crude material was then purified by silica gel column chromatography (8% → 10% benzene + 0.1% triethylamine in hexanes) to obtain 38.0 mg of a pale yellow oil (67% yield). The major isomer was assigned as *E* on the basis of 2D ^{1}H NOESY NMR.

^{1}H NMR (400 MHz, C_6D_6) δ 7.29 – 7.11 (m, 7H), 6.79 (t, *J* = 8.8 Hz, 2H), 3.61 (t, *J* = 6.5 Hz, 2H), 2.48 (s, 3H), 1.78 – 1.70 (m, 2H), 1.58 – 1.52 (m, 2H), 1.04 (t, *J* = 7.4 Hz, 3H).

^{13}C NMR (101 MHz, C_6D_6) δ 162.3 (d, *J* = 246.9 Hz), 150.1, 142.5, 132.3 (d, *J* = 3.4 Hz), 132.1 (d, *J* = 8.0 Hz), 129.7, 126.3, 121.5, 115.1 (d, *J* = 21.4 Hz), 69.1, 32.4, 19.6, 18.6, 14.1.p

^{19}F NMR (376 MHz, C_6D_6) δ -113.8.

FT-IR (neat film NaCl): 3055, 3021, 2959, 2933, 2872, 1640, 1603, 1506, 1442, 1380, 1311, 1225, 1156, 1124, 1093, 1072, 1026, 842, 763, 700, 586, 524 cm^{-1}.

HR-MS (ESI) m/z: [M+H]+ Calculated for $C_{19}H_{22}FO$ 285.1649; Found 285.1644

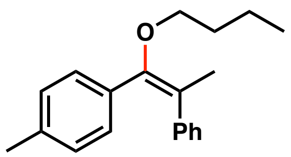

(*E*)-1-(1-butoxy-2-phenylprop-1-en-1-yl)-4-methylbenzene (8c)

Following *General Procedure C* using 10 mol% $[Ph_3C]^+[B(C_6F_5)_4]^-$ catalyst at 35 ºC for 6 hours. An aliquot of the crude material was taken for analysis of *E*/*Z* ratio by GC-FID, which was determined to be 36:1 *E*/*Z* (minor isomer retention time was confirmed by preparation of authentic material via enolate alkylation, see section **5.3**). The crude material was then purified by silica gel column chromatography (8% benzene + 0.1% triethylamine in hexanes) to obtain 34.0 mg of a colorless oil (61% yield). The major isomer was assigned as *E* on the basis of 2D ^{1}H NOESY NMR.

^{1}H NMR (400 MHz, C_6D_6) δ 7.02 – 6.95 (m, 2H), 6.94 – 6.90 (m, 4H), 6.78 (tq, *J* = 8.3, 1.2 Hz, 2H), 6.74 – 6.66 (m, 1H), 6.60 – 6.52 (m, 2H), 3.31 (t, *J* = 6.5 Hz, 2H), 2.12 (s, 3H), 1.74 (s, 3H), 1.41 – 1.29 (m, 2H), 1.21 – 1.07 (m, 2H), 0.61 (t, *J* = 7.4 Hz, 3H).

^{13}C NMR (101 MHz, C_6D_6) δ 151.3, 143.0, 137.1, 133.4, 130.4, 129.8, 128.9, 128.3, 126.0, 120.7, 69.0, 32.5, 21.1, 19.6, 18.6, 14.1.

FT-IR (neat film NaCl): 2957, 2929, 2870, 1508, 1441, 1239, 1124, 1109, 1071, 1025, 825, 762, 698 cm^{-1}.

HR-MS (ESI) m/z: [M+H]$^+$ Calculated for $C_{20}H_{25}O^+$: 281.1900; Found 281.1896.

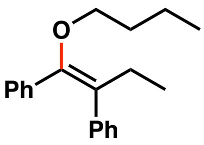

(*E*)-(1-butoxybut-1-ene-1,2-diyl)dibenzene (8d)

Following *General Procedure C* using 10 mol% $[Ph_3C]^+[B(C_6F_5)_4]^-$ catalyst at 35 ºC for 6 hours. An aliquot of the crude material was taken for analysis of *E*/*Z* ratio by GC-FID, which was determined to be 12:1 *E*/*Z* (minor isomer retention time was confirmed by preparation of authentic material via enolate alkylation, see section **5.3**). The crude material was then purified by silica gel column chromatography (10% benzene + 0.1% triethylamine in hexanes) to obtain 31.5 mg of a colorless oil (56% yield). The major isomer was assigned as *E* on the basis of 2D ^{1}H NOESY NMR.

^{1}H NMR (400 MHz, C_6D_6) δ 7.32 – 7.24 (m, 2H), 7.15 – 7.11 (m, 2H), 7.05 – 6.87 (m, 6H), 3.50 (t, *J* = 6.5 Hz, 2H), 2.86 (q, *J* = 7.5 Hz, 2H), 1.57 (ddt, *J* = 8.8, 7.9, 6.4 Hz, 2H), 1.42 – 1.27 (m, 1H), 1.15 (t, *J* = 7.5 Hz, 3H), 0.83 (t, *J* = 7.4 Hz, 3H).

^{13}C NMR (101 MHz, C_6D_6) δ 150.8, 141.4, 136.3, 130.4, 130.3, 128.1, 127.9, 127.5, 126.2, 69.4, 32.5, 25.8, 19.6, 14.0, 13.2. *one peak appears to be missing due to overlap with solvent.

FT-IR (neat film NaCl): 2959, 2930, 2871, 1490, 1443, 1236, 1126, 1094, 1072, 1024, 763, 698 cm^{-1}.

HR-MS (ESI) m/z: [M+H]+ Calculated for $C_{20}H_{25}O^+$: 281.1900; Found 281.1903.

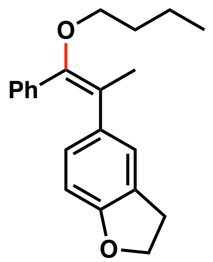

(*E*)-5-(1-butoxy-1-phenylprop-1-en-2-yl)-2,3-dihydrobenzofuran (8e)

Following *General Procedure C* using 10 mol% $[Ph_3C]^+[B(C_6F_5)_4]^-$ catalyst at 35 ºC for 6 hours. An aliquot of the crude material was taken for analysis of *E*/*Z* ratio by GC-FID, which was determined to be 39:1 *E*/*Z* (minor isomer retention time was confirmed by preparation of authentic material via enolate alkylation, see section **5.3**). The crude material was then purified by silica gel column chromatography (6% diethyl ether + 0.1% triethylamine in hexanes) to obtain 36.4 mg of a pale yellow oil (59% yield). The major isomer was assigned as *E* on the basis of 2D ^{1}H NOESY NMR.

^{1}H NMR (400 MHz, C_6D_6) δ 7.39 – 7.34 (m, 2H), 7.04 – 6.99 (m, 2H), 6.96 – 6.87 (m, 3H), 6.71 – 6.61 (m, 1H), 3.96 (t, *J* = 8.7 Hz, 2H), 3.53 (t, *J* = 6.5 Hz, 2H), 2.48 – 2.41 (m, 2H), 2.36 (s, 3H), 1.59 (ddt, *J* = 8.9, 8.1, 6.3 Hz, 2H), 1.42 – 1.33 (m, 2H), 0.85 (t, *J* = 7.4 Hz, 3H).

^{13}C NMR (101 MHz, C_6D_6) δ 159.2, 150.4, 136.8, 134.7, 130.3, 129.7, 128.0, 127.3, 126.9, 126.1, 121.5, 109.2, 70.8, 69.1, 32.5, 29.6, 19.6, 19.1, 14.0.

FT-IR (neat film NaCl): 3054, 2957, 2931, 2871, 1637, 1610, 1599, 1490, 1443, 1379, 1240, 1120, 1103, 1072, 984, 945, 818, 774, 701, 609 cm^{-1}.

HR-MS (ESI) m/z: [M+H]+ Calculated for $C_{21}H_{25}O_2$ 309.1849; Found 309.1848.

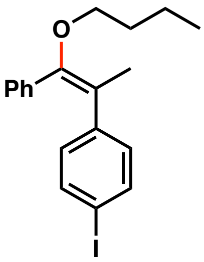

(*E*)-1-(1-butoxy-1-phenylprop-1-en-2-yl)-4-iodobenzene (8f)

Following *General Procedure C* using 10 mol% $[Ph_3C]^+[B(C_6F_5)_4]^-$ catalyst at 60 ºC for 6 hours. An aliquot of the crude material was taken for analysis of *E*/*Z* ratio by GC-FID, which was determined to be 25:1 *E*/*Z* (minor isomer retention time was confirmed by preparation of authentic material via enolate alkylation, see section **5.3**). The crude material was then purified by silica gel column chromatography (10% benzene + 0.1% triethylamine in hexanes) to obtain 49.2 mg of a colorless oil (63% yield). The major isomer was assigned as *E* on the basis of 2D ^{1}H NOESY NMR.

^{1}H NMR (400 MHz, C_6D_6) δ 7.32 – 7.24 (m, 2H), 7.23 – 7.12 (m, 4H), 6.99 – 6.87 (m, 2H), 6.69 – 6.60 (m, 2H), 3.45 (t, *J* = 6.5 Hz, 2H), 2.21 (s, 3H), 1.59 – 1.47 (m, 2H), 1.42 – 1.27 (m, 2H), 0.83 (t, *J* = 7.4 Hz, 3H).

^{13}C NMR (101 MHz, C_6D_6) δ 151.6, 142.1, 137.3, 137.0, 135.7, 131.6, 130.3, 127.8, 119.7, 91.4, 69.0, 32.3, 19.5, 18.1, 14.0.

FT-IR (neat film NaCl): 2956, 2929, 2870, 1483, 1241, 1100, 1129, 1070, 1030, 820, 774, 698 cm^{-1}.

HR-MS (FD) m/z: [M•]$^+$ Calculated for $C_{19}H_{21}OI^+$: 392.0637; Found: 392.0639.

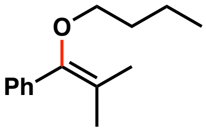

(1-butoxy-2-methylprop-1-en-1-yl)benzene (8g)

Following the *General Procedure C* using 8 mol% $[Ph_3C]^+[B(C_6F_5)_4]^-$ catalyst at 60 ºC for 3 hours. The product was purified by silica gel column chromatography (1% diethyl ether + 0.1% triethylamine in pentane) to obtain 30.0 mg of a colorless oil (73% yield).

^{1}H NMR (400 MHz, $CDCl_3$) δ 7.37 – 7.29 (m, 4H), 7.28 – 7.23 (m, 1H), 3.38 (t, *J* = 6.5 Hz, 2H), 1.84 (s, 3H), 1.66 (s, 3H), 1.60 – 1.49 (m, 2H), 1.45 – 1.30 (m, 2H), 0.88 (t, *J* = 7.3 Hz, 3H).

^{13}C NMR (101 MHz, $CDCl_3$) δ 148.0, 136.2, 129.6, 127.9, 127.3, 115.7, 69.2, 32.1, 19.8, 19.4, 17.8, 14.0.

FT-IR (neat film NaCl): 2958, 2929, 2871, 1443, 1291, 1141, 1072, 1047, 775, 699 cm^{-1}.

HR-MS (FI) m/z: [M•]+ Calculated for $C_{14}H_{20}O^+$: 204.1514; Found 204.1524.

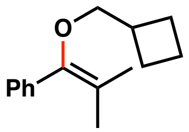

(1-(cyclobutylmethoxy)-2-methylprop-1-en-1-yl)benzene (8h)

Following the above procedure using 8 mol% $[Ph_3C]^+[B(C_6F_5)_4]^-$ catalyst at 60 ºC for 3 hours. The product was purified by silica gel column chromatography (1% diethyl ether + 0.1% triethylamine in hexanes) to obtain 27.8 mg of a slightly yellow-colored oil (64% yield).

^{1}H NMR (400 MHz, CD_2Cl_2) δ 7.36 – 7.23 (m, 5H), 3.34 (d, *J* = 6.8 Hz, 2H), 2.62 – 2.50 (m, 1H), 2.07 – 1.95 (m, 2H), 1.94 – 1.69 (m, 7H), 1.65 (s, 3H).

^{13}C NMR (101 MHz, CD_2Cl_2) δ 148.1, 136.5, 129.9, 128.1, 127.5, 115.9, 73.9, 35.6, 25.3, 19.8, 18.8, 17.8.

FT-IR (neat film NaCl): 2974, 2928, 2857, 1442, 1290, 1139, 1046, 1024, 773, 700 cm^{-1}.

HR-MS (ESI) m/z: $[M+K]^+$ Calculated for $C_{15}H_{20}KO^+$: 255.1146; Found 255.1140.

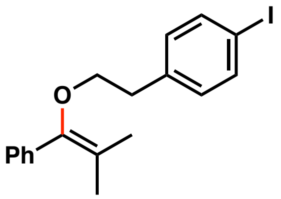

1-iodo-4-(2-((2-methyl-1-phenylprop-1-en-1-yl)oxy)ethyl)benzene (9i)

Following *General Procedure C* using 8 mol% $[Ph_3C]^+[B(C_6F_5)_4]^-$ catalyst at 60 ºC for 3 hours. The product was purified by silica gel column chromatography (1% diethyl ether + 0.1% triethylamine in hexanes) and subjected to high vacuum at 90 ºC (to remove excess silyl ether) to obtain 54.1 mg of a colorless oil (72% yield).

^{1}**H NMR** (400 MHz, $CDCl_3$) δ 7.61 – 7.53 (m, 2H), 7.36 – 7.20 (m, 5H), 6.95 – 6.87 (m, 2H), 3.58 (t, *J* = 6.9 Hz, 2H), 2.81 (t, *J* = 6.9 Hz, 2H), 1.75 (s, 3H), 1.64 (s, 3H).
^{13}**C NMR** (101 MHz, $CDCl_3$) δ 147.6, 138.6, 137.4, 135.7, 131.1, 129.6, 128.0, 127.5, 116.2, 91.4, 69.6, 36.0, 19.8, 17.8 cm^{-1}.
FT-IR (neat film NaCl): 2916, 2856, 1485, 1442, 1290, 1137, 1025, 1006, 807, 773, 700 cm^{-1}.
HR-MS (FD) m/z: [M•]+ Calculated for $C_{18}H_{19}IO$: 378.0481; Found 378.0489.

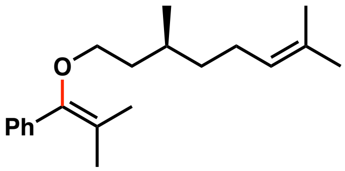

(*S*)-(1-((3,7-dimethyloct-6-en-1-yl)oxy)-2-methylprop-1-en-1-yl)benzene (8j)
Following *General Procedure C* using 8 mol% $[Ph_3C]^+[B(C_6F_5)_4]^-$ catalyst at 60 ºC for 3 hours. The product was purified by silica gel column chromatography (1% diethyl ether + 0.1% triethylamine in hexanes) and subjected to high vacuum at 45 ºC (to remove excess silyl ether) to obtain 47.2 mg of a colorless oil (82% yield).
^{1}**H NMR** (400 MHz, $CDCl_3$) δ 7.39 – 7.30 (m, 4H), 7.30 – 7.20 (m, 1H), 5.08 (tp, *J* = 7.2, 1.4 Hz, 1H), 3.49 – 3.35 (m, 2H), 2.06 – 1.87 (m, 2H), 1.85 (s, 3H), 1.71 – 1.53 (m, 10H), 1.43 – 1.24 (m, 3H), 1.11 (dddd, *J* = 13.4, 9.5, 7.7, 5.9 Hz, 1H), 0.83 (d, *J* = 6.5 Hz, 3H).
^{13}**C NMR** (101 MHz, $CDCl_3$) δ 148.0, 136.2, 131.2, 129.6, 127.9, 127.4, 124.9, 115.7, 67.6, 37.2, 37.0, 29.3, 25.8, 25.5, 19.8, 19.6, 17.9, 17.7.
FT-IR (neat film NaCl): 2961, 2922, 2856, 1443, 1377, 1290, 1141, 1071, 1058, 1022, 773, 700 cm^{-1}.
HR-MS (ESI) m/z: [M+H]+ Calculated for $C_{20}H_{31}O^+$: 287.2369; Found 287.2368.

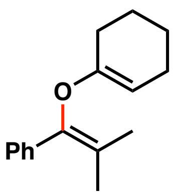

(1-(cyclohex-1-en-1-yloxy)-2-methylprop-1-en-1-yl)benzene (8k)
Following *General Procedure C* using 8 mol% $[Ph_3C]^+[B(C_6F_5)_4]^-$ catalyst at 60 ºC for 3 hours. The product was purified by silica gel column chromatography (1% diethyl ether + 0.1% triethylamine in hexanes) and subjected to high vacuum at 45 ºC (to remove excess silyl enol ether) to obtain 31.9 mg of a colorless oil (70% yield).

^{1}H NMR (400 MHz, CD_2Cl_2) δ 7.40 – 7.27 (m, 4H), 7.27 – 7.17 (m, 1H), 4.67 (tt, J = 3.9, 1.3 Hz, 1H), 2.09 (ttd, J = 6.3, 2.2, 1.3 Hz, 2H), 1.89 (tdt, J = 6.1, 4.2, 2.2 Hz, 2H), 1.81 (s, 3H), 1.76 (s, 3H), 1.63 (dtd, J = 12.4, 6.4, 3.0 Hz, 2H), 1.50 – 1.37 (m, 2H).

^{13}C NMR (101 MHz, CD_2Cl_2) δ 152.5, 143.6, 137.1, 129.2, 128.0, 127.4, 120.7, 98.7, 27.6, 23.7, 23.2, 22.8, 19.9, 18.4.

FT-IR (neat film NaCl): 2927, 2857, 2840, 1673, 1443, 1372, 1174, 1151, 1137, 1018, 776, 716, 698 cm^{-1}.

HR-MS (FI) m/z: [M•]+ Calculated for $C_{16}H_{20}O$: 228.1514; Found 228.1526.

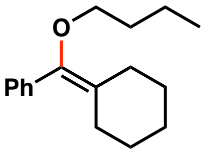

(butoxy(cyclohexylidene)methyl)benzene (8l)

Following *General Procedure C* using 8 mol% $[Ph_3C]^+[B(C_6F_5)_4]^-$ catalyst at 60 ºC for 3 hours. The product was purified by silica gel column chromatography (1% diethyl ether + 0.1% triethylamine in hexanes) to obtain 39.1 mg of a colorless oil (80% yield).

^{1}H NMR (400 MHz, $CDCl_3$) δ 7.33 – 7.17 (m, 5H), 3.32 (t, *J* = 6.6 Hz, 2H), 2.37 (t, *J* = 5.7 Hz, 2H), 2.08 – 2.01 (m, 2H), 1.59 – 1.39 (m, 8H), 1.37 – 1.23 (m, 2H), 0.82 (t, *J* = 7.4 Hz, 3H).

^{13}C NMR (101 MHz, $CDCl_3$) δ 145.6, 136.1, 129.7, 127.9, 127.4, 123.9, 69.2, 32.0, 30.0, 28.3, 27.9, 27.7, 27.0, 19.4, 14.0.

FT-IR (neat film NaCl): 2958, 2924, 2851, 1444, 1210, 1121, 1080, 775, 700 cm^{-1}.

HR-MS (FD) m/z: [M$^{\cdot}$]+ Calculated for $C_{17}H_{24}O$: 244.1827; Found 244.1838.

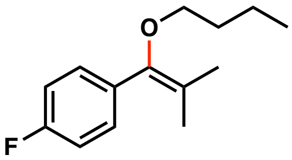

1-(1-butoxy-2-methylprop-1-en-1-yl)-4-fluorobenzene (8m)

Following *General Procedure C* using 8 mol% $[Ph_3C]^+[B(C_6F_5)_4]^-$ catalyst at 60 ºC for 3 hours. The product was purified by silica gel column chromatography (1% diethyl ether + 0.1% triethylamine in hexanes) to obtain 34.5 mg of a colorless oil (78% yield).

^{1}H NMR (400 MHz, $CDCl_3$) δ 7.26 – 7.16 (m, 2H), 7.00 – 6.90 (m, 2H), 3.29 (t, *J* = 6.6 Hz, 2H), 1.76 (s, 3H), 1.56 (s, 3H), 1.53 – 1.41 (m, 2H), 1.35 – 1.22 (m, 2H), 0.81 (t, *J* = 7.3 Hz, 3H).

^{13}C NMR (101 MHz, $CDCl_3$) δ 161.9 (d, *J* = 246.2 Hz), 146.9, 132.1 (d, *J* = 3.3 Hz), 131.1 (d, *J* = 8.0 Hz), 115.8, 114.8 (d, *J* = 21.3 Hz), 69.0, 31.9, 19.6, 19.2, 17.7, 13.8.

^{19}F NMR (282 MHz, $CDCl_3$) δ -114.6.

FT-IR (neat film NaCl): 2958, 2929, 2871, 1602, 1506, 1289, 1224, 1154, 1140, 1091, 1039, 842, 811 cm^{-1}.

HR-MS (FD) m/z: $[M^{\bullet}]^+$ Calculated for $C_{14}H_{19}OF$: 222.1419; Found 222.1440.

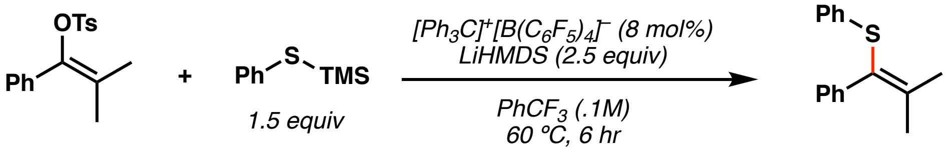

(2-methyl-1-phenylprop-1-en-1-yl)(phenyl)sulfane (8n)

Following *General Procedure C* using 8 mol% $[Ph_3C]^+[B(C_6F_5)_4]^-$ catalyst at 60 ºC for 6 hours. The crude material was then purified by silica gel column chromatography (10% benzene + 0.1% triethylamine in hexanes) then dried under high vacuum at 55 ºC to obtain 24.1 mg of a colorless oil (50% yield) which matched the reported NMR spectra.[13]

6.3 Preparation of Authentic Minor Isomers via Enolate Alkylation

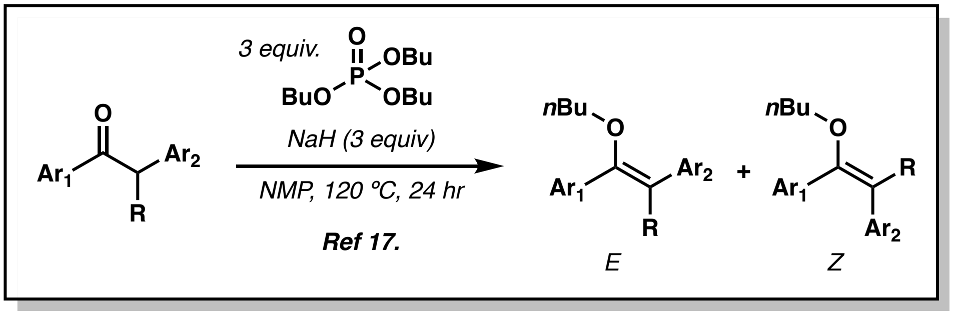

To validate that a minor peak on GC-FID in the catalytic vinyl ether synthesis above corresponded to a minor *Z* olefin isomer, authentic standards were prepared via enolate alkylation using a reported procedure.[18] Moreover, this provided a means to compare our reported methodology to alternative methods for preparing vinyl ethers by comparing stereoselectivities. To a flame-dried 3-neck flask equipped with a magnetic stir bar and reflux condenser was added NaH (6.0 mmol, 3.0 equivalents), ketone (2.0 mmol, 1.0 equivalent), then NMP (0.17 M). Then, while under N_2 atmosphere, commercially-available tributyl phosphate (6.0 mmol, 3.0 equivalents) was added dropwise while at room temperature. The mixture was then heated in an oil bath to 120 ºC

overnight and monitored by TLC to assess complete consumption of starting material. Upon completion of the reaction, the mixture was slowly diluted with diethyl ether, then water was added dropwise to the stirring mixture. The mixture was then extracted with diethyl ether three times, and the combined organics were washed with water three times then with saturated brine once. An aliquot of the crude material was taken for analysis of *E*/*Z* ratio by GC-FID for comparison to our developed methodology. The crude material was then purified by silica gel column chromatography with 2–5% diethyl ether (+0.1% triethylamine) then dried under high vacuum at 60 ºC to obtain vinyl ether as a mixture of isomers. We found it difficult to separate the isomers using silica gel chromatography, so further separation of the isomers was achieved by prepatory reverse phase HPLC using an Agilent 1200 series instrument with a reverse phase Alltima C18 (5μ, 25 cm length, 1 cm internal diameter) column and water/acetonitrile mobile phase to achieve sufficient material for characterization (in some cases, separation for was very poor and required repetitive subjection to HPLC purification). The minor isomer was characterized and its retention time on GC-FID was obtained to validate the formation of the minor *Z* isomer using our catalytic method.

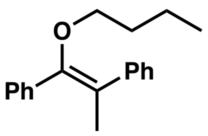

(*Z*)-(1-butoxyprop-1-ene-1,2-diyl)dibenzene (SI-39)

Prepared according to above reported procedure.[18] The selectivity of the reaction was determined to be 1.7:1 *E*/*Z* by GC-FID. The desired *Z* isomer was isolated by reverse phase HPLC using 80:20 MeCN/Water.

^{1}H NMR (400 MHz, C_6D_6) δ 7.65 – 7.58 (m, 2H), 7.47 – 7.39 (m, 2H), 7.34 – 7.25 (m, 2H), 7.22 – 7.18 (m, 2H), 7.14 – 7.07 (m, 2H), 3.36 (t, *J* = 6.4 Hz, 2H), 1.98 (s, 3H), 1.34 (ddt, *J* = 8.9, 8.0, 6.3 Hz, 2H), 1.17 – 1.03 (m, 2H), 0.66 (t, *J* = 7.4 Hz, 3H).

^{13}C NMR (101 MHz, $CDCl_3$) δ 150.1, 141.1, 136.3, 129.8, 128.5, 128.2, 127.9, 127.8, 126.2, 118.4, 69.6, 31.8, 19.7, 19.1, 13.8.

FT-IR (neat film NaCl): 2957, 2931, 1492, 1442, 1249, 1123, 760, 698 cm^{-1}.

HR-MS (FD) m/z: [M•]+ Calculated for $C_{19}H_{22}O$: 266.1671; Found 266.1682.

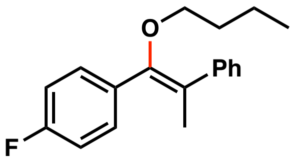

(*Z*)-1-(1-butoxy-2-phenylprop-1-en-1-yl)-4-fluorobenzene (SI-40)

Prepared according to above reported procedure.[18] The selectivity of the reaction was determined to be 2.2:1 *E*/*Z* by GC-FID. The desired *Z* isomer was isolated by reverse phase HPLC using 80:20 MeCN/Water.

^{1}H NMR (400 MHz, C_6D_6) δ 7.62 – 7.53 (m, 2H), 7.29 (dd, *J* = 8.5, 7.0 Hz, 2H), 7.22 – 7.17 (m, 3H), 6.85 – 6.76 (m, 2H), 3.27 (t, *J* = 6.4 Hz, 2H), 1.90 (s, 3H), 1.32 (ddt, *J* = 8.8, 8.0, 6.3 Hz, 2H), 1.14 – 1.05 (m, 2H), 0.67 (t, *J* = 7.4 Hz, 3H).

^{13}C NMR (101 MHz, $CDCl_3$) 162.3 (d, *J* = 246.9 Hz), 149.0, 141.4, 132.4 (d, *J* = 3.5 Hz), 131.4 (d, *J* = 7.9 Hz), 128.5, 126.2, 118.6, 114.9 (d, *J* = 21.4 Hz), 69.2, 31.7, 19.4, 19.0, 13.5.

^{19}F NMR (376 MHz, C_6D_6) δ –113.4.

FT-IR (neat film NaCl): 3054, 3020, 2958, 2933, 2871, 1640, 1602, 1507, 1442, 1380, 1300, 1223, 1156, 1124, 1093, 1076, 1028, 1005, 846, 763, 698, 567, 522 cm^{-1}.

HR-MS (ESI) m/z: [M+H]+ Calculated $C_{19}H_{22}FO$ 285.1649; Found 285.1653.

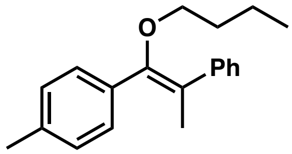

(*Z*)-1-(1-butoxy-2-phenylprop-1-en-1-yl)-4-methylbenzene (SI-41)

Prepared according to above reported procedure.[18] The selectivity of the reaction was determined to be 1.6:1 *E*/*Z* by GC-FID. The desired *Z* isomer was isolated by reverse phase HPLC using 75:25 MeCN/Water. While minimal separation was observed, collection of the right-most shoulder and repetitive subjection to HPLC purification enabled sufficiently pure material to be obtained for characterization and determination of retention time by GC-FID for comparison to our catalytic methodology. The material was contaminated with 8% of the *Z* isomer, which was difficult to remove.

^{1}H NMR (400 MHz, D_2-DCM) δ 7.47 – 7.40 (m, 2H), 7.37 – 7.27 (m, 4H), 7.26 – 7.16 (m, 3H), 3.34 (t, *J* = 6.6 Hz, 2H), 2.38 (s, 3H), 1.92 (s, 3H), 1.44 – 1.33 (m, 2H), 1.21 – 1.10 (m, 2H), 0.75 (t, *J* = 7.4 Hz, 3H).

^{13}C NMR (101 MHz, D_2-DCM) δ 150.5, 142.1, 138.1, 133.5, 129.9, 129.1, 128.7, 128.0, 126.3, 117.9, 69.7, 32.1, 21.4, 19.7, 19.4, 13.9.

FT-IR (neat film NaCl): 2956, 2922, 2853, 1632, 1509, 1258, 1125, 1109, 1075, 1004, 830, 761, 697 cm^{-1}.

HR-MS (FD) m/z: [M•]$^+$ Calculated for $C_{20}H_{24}O^+$: 280.1827; Found: 280.1824.

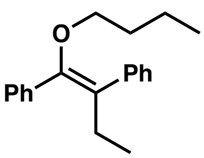

(*Z*)-(1-butoxybut-1-ene-1,2-diyl)dibenzene (SI-42)

Prepared according to above reported procedure.[18] The selectivity of the reaction was determined to be 1:1 *E*/*Z* by GC-FID. The desired *Z* isomer was isolated by reverse phase HPLC using 80:20 MeCN/Water.

^{1}H NMR (400 MHz, C_6D_6) δ 7.51 – 7.43 (m, 4H), 7.33 – 7.24 (m, 2H), 7.22 – 7.17 (m, 2H), 7.15 – 7.08 (m, 2H), 3.35 (t, *J* = 6.4 Hz, 2H), 2.42 (q, *J* = 7.4 Hz, 2H), 1.38 – 1.26 (m, 2H), 1.14 – 1.00 (m, 2H), 0.92 (t, *J* = 7.4 Hz, 3H), 0.65 (t, *J* = 7.4 Hz, 3H).

^{13}C NMR (101 MHz, C_6D_6) δ 150.2, 140.3, 136.8, 129.8, 129.5, 128.4, 128.2, 126.5, 125.8, 69.5, 32.1, 26.2, 19.3, 14.0, 13.8. *other peaks not apparent.

FT-IR (neat film NaCl): 2958, 2931, 2870, 1494, 1442, 1124, 1073, 760, 698, 424 cm^{-1}.

HR-MS (FD) m/z: [M•]$^+$ Calculated for $C_{20}H_{24}O^+$: 280.1827; Found: 280.1819.

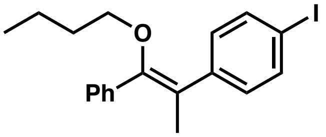

(*Z*)-1-(1-butoxy-1-phenylprop-1-en-2-yl)-4-iodobenzene (SI-43)

Prepared according to above reported procedure.[18] The selectivity of the reaction was determined to be 1.1:1 *E*/*Z* by GC-FID. The desired *Z* isomer was isolated by reverse phase HPLC using 80:20 MeCN/Water. While minimal separation was observed, collection of the right-most shoulder and repetitive subjection to HPLC purification enabled sufficiently pure material to be obtained for characterization and determination of retention time by GC-FID for comparison to our catalytic methodology.

^{1}H NMR (400 MHz, D_2-DCM) δ 7.70 – 7.62 (m, 2H), 7.44 – 7.36 (m, 4H), 7.35 (m, 1H), 7.29 – 7.21 (m, 2H), 3.35 (t, *J* = 6.6 Hz, 2H), 1.90 (d, *J* = 0.7 Hz, 3H), 1.46 – 1.34 (m, 2H), 1.24 – 1.10 (m, 2H), 0.77 (t, *J* = 7.4 Hz, 3H).

^{13}**C NMR** (101 MHz, D_2-DCM) δ 151.1, 141.5, 137.1, 136.2, 130.9, 130.0, 128.5, 128.4, 116.8, 91.2, 69.8, 32.0, 19.4, 19.3, 13.8.

FT-IR (neat film NaCl): 2956, 2922, 2851, 1632, 1486, 1467, 1258, 1130, 1100, 1005, 821, 774 cm^{-1}.

HR-MS (FD) m/z: $[M•]^+$ Calculated for $C_{19}H_{21}OI^+$: 392.0637; Found: 392.0644.

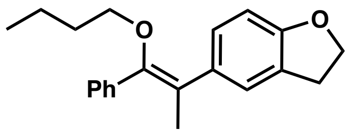

(*Z*)-5-(1-butoxy-1-phenylprop-1-en-2-yl)-2,3-dihydrobenzofuran (SI-44)

Prepared according to above reported procedure.[18] The selectivity of the reaction was determined to be 1.5:1 *E*/*Z* by GC-FID. The desired *Z* isomer was isolated by reverse phase HPLC using 80:20 MeCN/Water.

^{1}**H NMR** (400 MHz, C_6D_6) δ 7.50 – 7.46 (m, 2H), 7.45 (m, 1H), 7.38 (ddt, *J* = 8.3, 1.7, 0.8 Hz, 1H), 7.23 – 7.18 (m, 2H), 7.14 – 7.09 (m, 1H), 6.95 (d, *J* = 8.3 Hz, 1H), 4.11 (t, *J* = 8.7 Hz, 2H), 3.41 (t, *J* = 6.4 Hz, 2H), 2.69 (dd, *J* = 9.2, 8.2 Hz, 2H), 2.02 (s, 3H), 1.42 – 1.36 (m, 2H), 1.20 – 1.11 (m, 2H), 0.69 (t, *J* = 7.3 Hz, 3H).

^{13}**C NMR** (101 MHz, C_6D_6) δ 159.3, 149.8, 137.3, 134.0, 130.0, 128.7, 128.3, 127.8, 126.5, 125.6, 118.8, 108.9, 71.0, 69.5, 32.2, 29.9, 20.2, 19.4, 13.8.

FT-IR (neat film NaCl): 3055, 2957, 2932, 2871, 1611, 1599, 1492, 1442, 1227, 1121, 1106, 1073, 984, 944, 816, 780, 704. 615 cm^{-1}.

HR-MS (ESI) m/z: [M+H]+ Calculated for $C_{21}H_{25}O_2$ 309.1849; Found 309.1853.

6.4 GC-FID Data for Stereoselective Vinyl Ether Synthesis

The following GC-FID traces compares this method vs a reported enolate alkylation protocol[18] for the stereoselective synthesis of vinyl ethers.

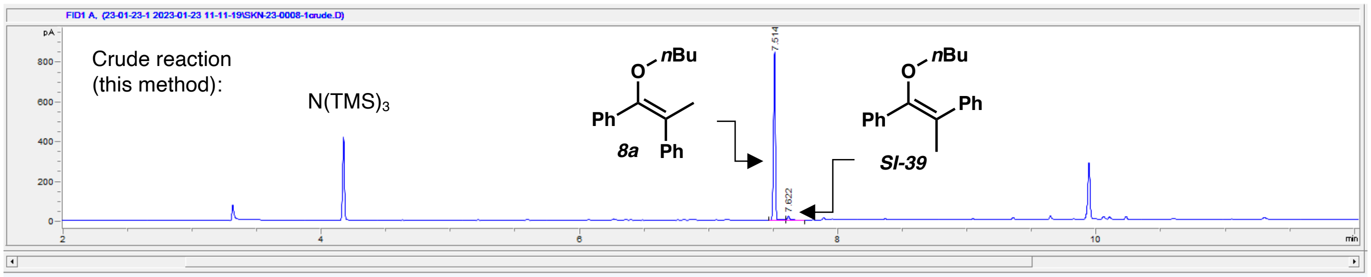

File Information

GC-File	SKN-23-0008-1crude.D
File Path	C:\Chem32\1\Data\23-01-23-1 2023-01-23 11-11-19

#	Time	Area	Height	Width	Area%	Symmetry
1	7.514	837.3	835.2	0.0167	97.468	1.113
2	7.622	21.8	21.2	0.016	2.532	0.851

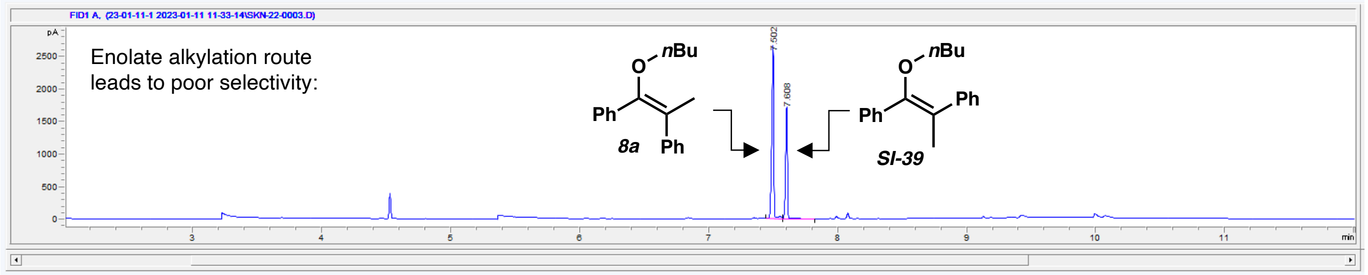

File Information

GC-File	SKN-22-0003.D
File Path	C:\Chem32\1\Data\23-01-11-1 2023-01-11 11-33-14

#	Time	Area	Height	Width	Area%	Symmetry
1	7.502	2838.9	2547.7	0.0167	62.932	1.539
2	7.608	1672.2	1667.7	0.0157	37.068	1.197

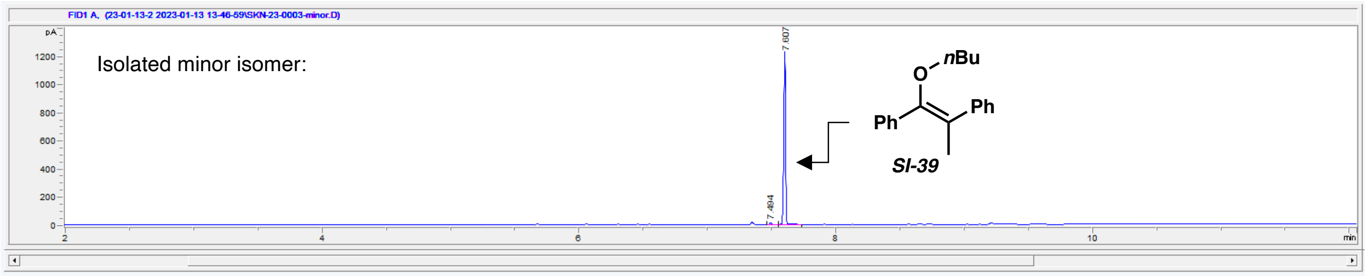

File Information

GC-File	SKN-23-0003-minor.D
File Path	C:\Chem32\1\Data\23-01-13-2 2023-01-13 13-46-59

#	Time	Area	Height	Width	Area%	Symmetry
1	7.494	10.6	10.5	0.0167	0.869	0.862
2	7.607	1203.2	1216	0.0165	99.131	1.133

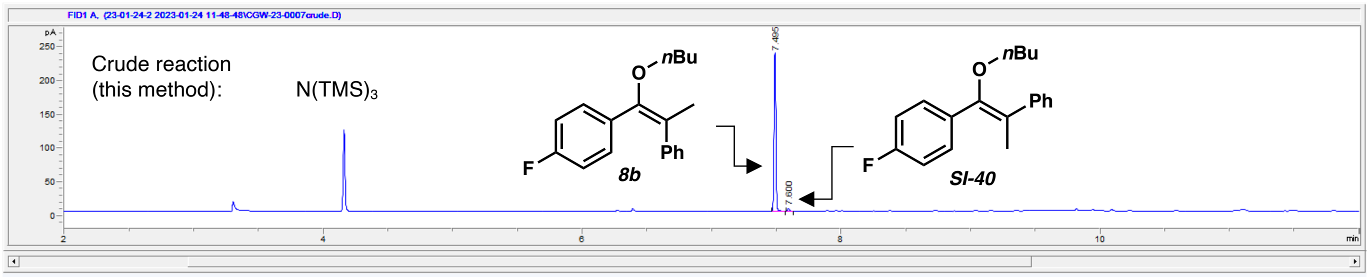

File Information

GC-File	CGW-23-0007crude.D
File Path	C:\Chem32\1\Data\23-01-24-2 2023-01-24 11-48-48

#	Time	Area	Height	Width	Area%	Symmetry
1	7.495	227.8	232.2	0.0154	98.413	0.93
2	7.6	3.7	3.7	0.0157	1.587	0.925

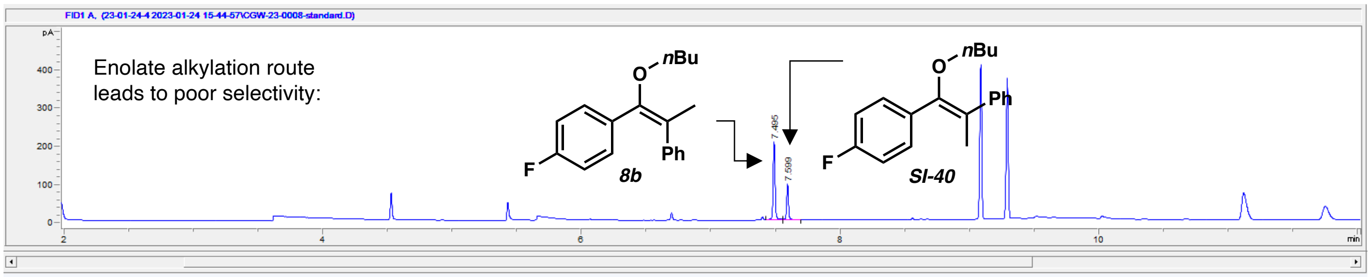

File Information

GC-File	CGW-23-0008-standard.D
File Path	C:\Chem32\1\Data\23-01-24-4 2023-01-24 15-44-57

#	Time	Area	Height	Width	Area%	Symmetry
1	7.495	220.5	202.3	0.0167	68.920	0.877
2	7.599	99.5	92.6	0.0165	31.080	0.9

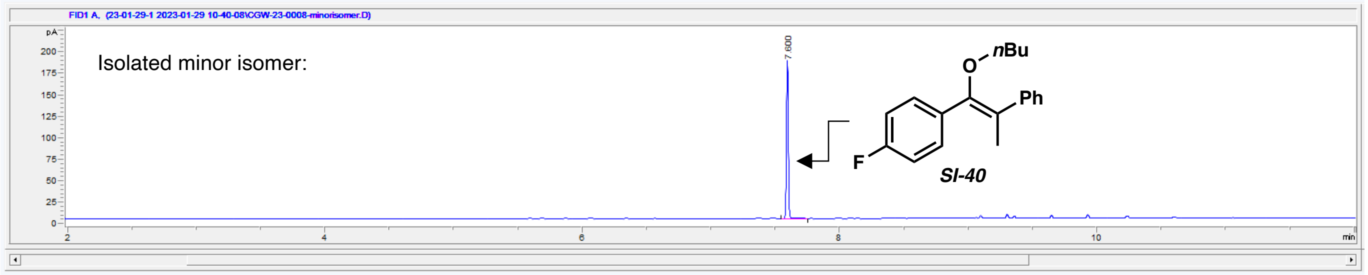

File Information

GC-File	CGW-23-0008-minorisomer.D

#	Time	Area	Height	Width	Area%	Symmetry
1	7.6	181.8	181.3	0.0157	100.000	0.94

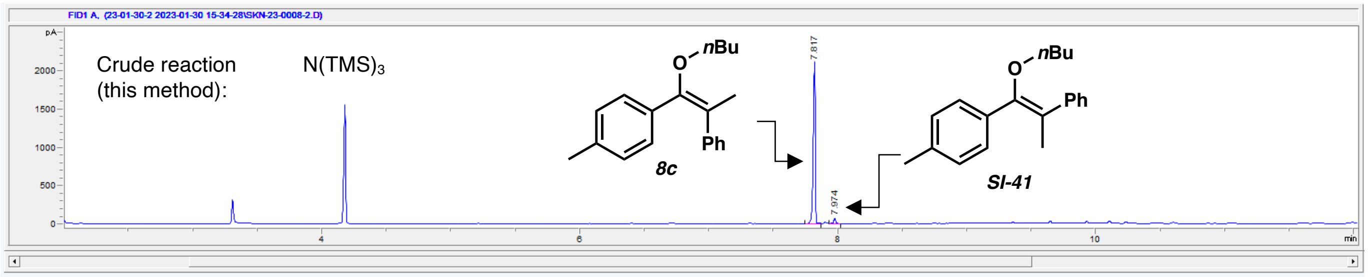

File Information

GC-File	SKN-23-0008-2.D
File Path	C:\Chem32\1\Data\23-01-30-2 2023-01-30 15-34-28

#	Time	Area	Height	Width	Area%	Symmetry
1	7.817	2334.3	2089.9	0.017	97.351	1.567
2	7.974	63.5	60.2	0.0173	2.649	0.98

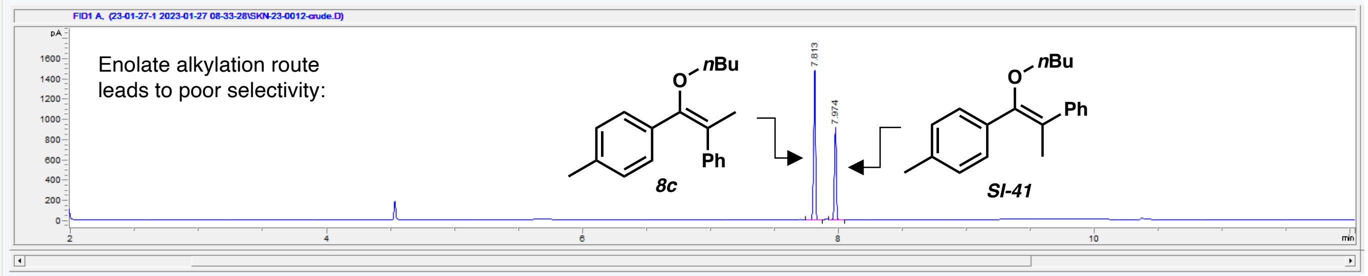

File Information

GC-File	SKN-23-0012-crude.D
File Path	C:\Chem32\1\Data\23-01-27-1 2023-01-27 08-33-28

#	Time	Area	Height	Width	Area%	Symmetry
1	7.813	1498.7	1478.6	0.0158	61.961	1.348
2	7.974	920.1	902.6	0.0169	38.039	1.148

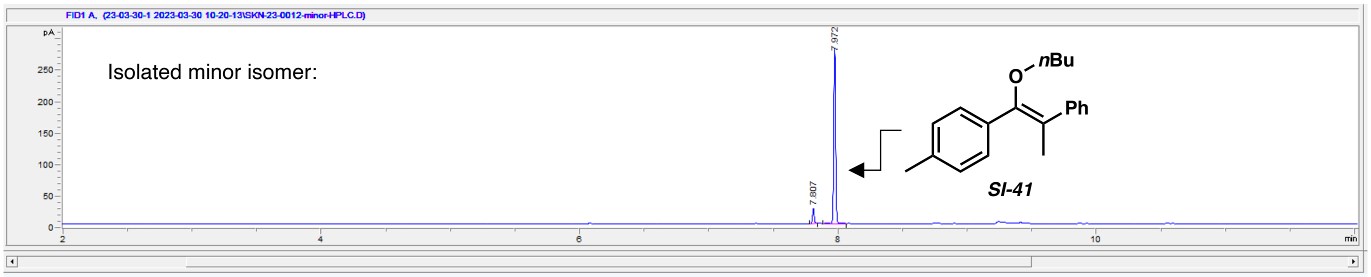

File Information

GC-File	SKN-23-0012-minor-HPLC.D
File Path	C:\Chem32\1\Data\23-03-30-1 2023-03-30 10-20-13

#	Time	Area	Height	Width	Area%	Symmetry
1	7.807	23.3	23.5	0.0165	7.808	0.932
2	7.972	275	271.6	0.0158	92.192	0.982

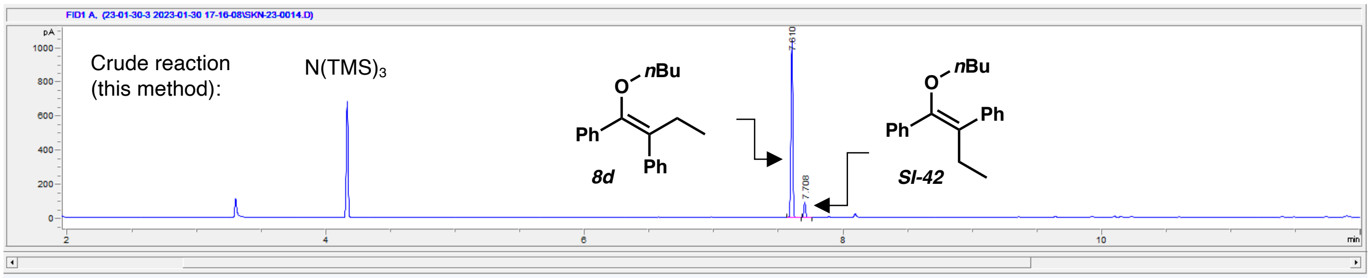

File Information	
GC-File	SKN-23-0014.D
File Path	C:\Chem32\1\Data\23-01-30-3 2023-01-30 17-16-08

#	Time	Area	Height	Width	Area%	Symmetry
1	7.61	1026.5	1029.3	0.0156	92.255	1.131
2	7.708	86.2	86.7	0.0156	7.745	0.924

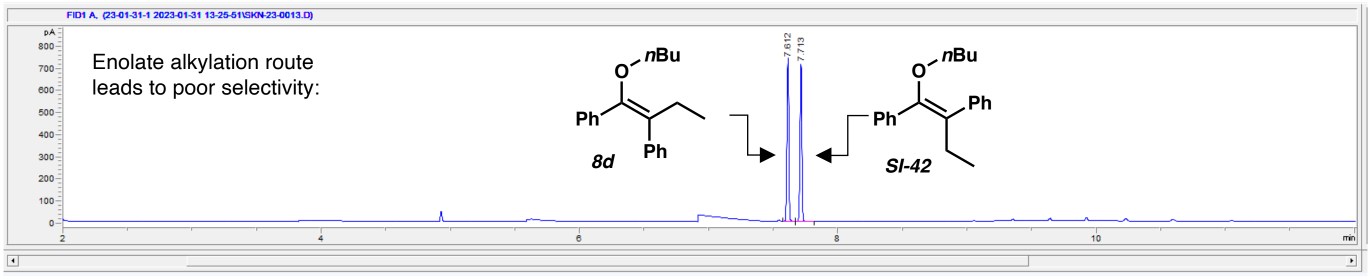

File Information	
GC-File	SKN-23-0013.D
File Path	C:\Chem32\1\Data\23-01-31-1 2023-01-31 13-25-51

#	Time	Area	Height	Width	Area%	Symmetry
1	7.612	722.7	727.2	0.0156	50.703	1.075
2	7.713	702.7	706.6	0.0156	49.297	1.097

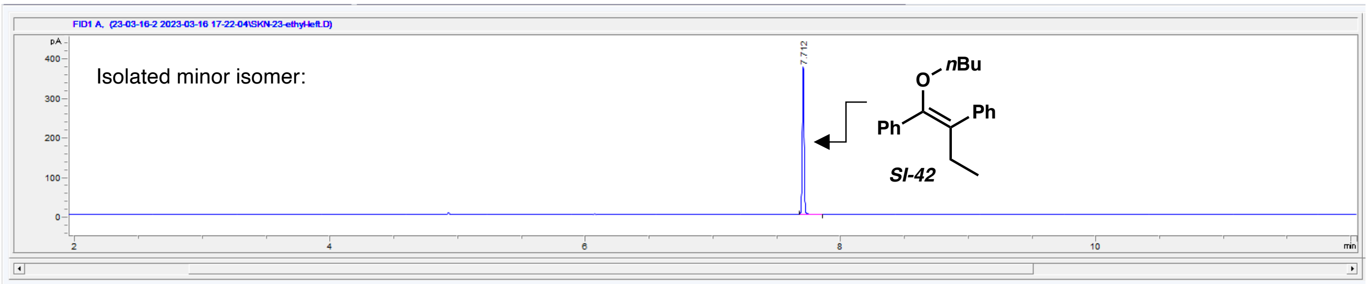

File Information	
GC-File	SKN-23-ethyl-left.D

#	Time	Area	Height	Width	Area%	Symmetry
1	7.712	365.7	369.3	0.0156	100.000	0.97

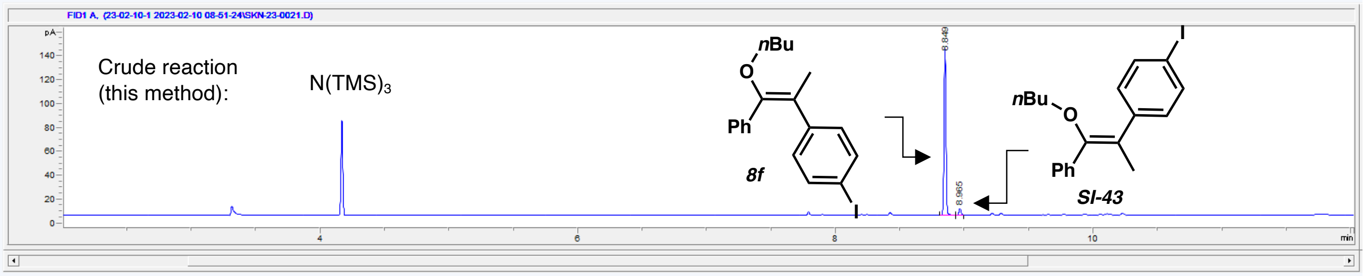

File Information

GC-File	SKN-23-0021.D
File Path	C:\Chem32\1\Data\23-02-10-1 2023-02-10 08-51-24

#	Time	Area	Height	Width	Area%	Symmetry
1	8.849	144.4	137.6	0.0162	96.154	0.937
2	8.965	5.8	5.4	0.0165	3.846	0.883

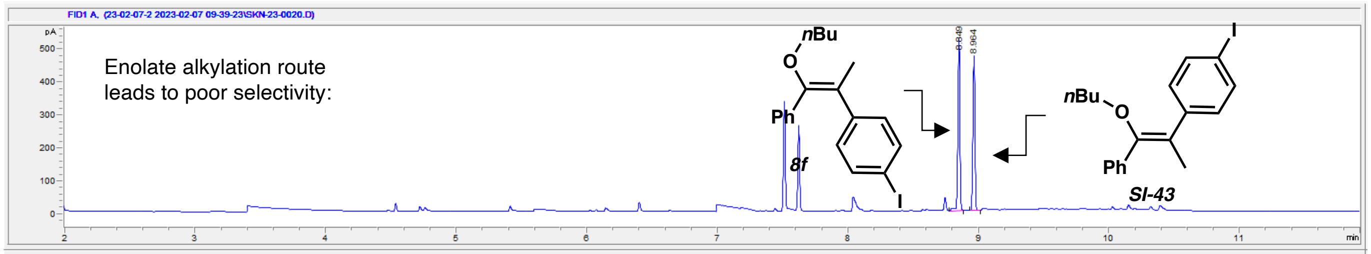

File Information

GC-File	SKN-23-0020.D
File Path	C:\Chem32\1\Data\23-02-07-2 2023-02-07 09-39-23

#	Time	Area	Height	Width	Area%	Symmetry
1	8.849	563.5	514.1	0.0164	53.320	1.256
2	8.964	493.3	465.2	0.0174	46.680	1.091

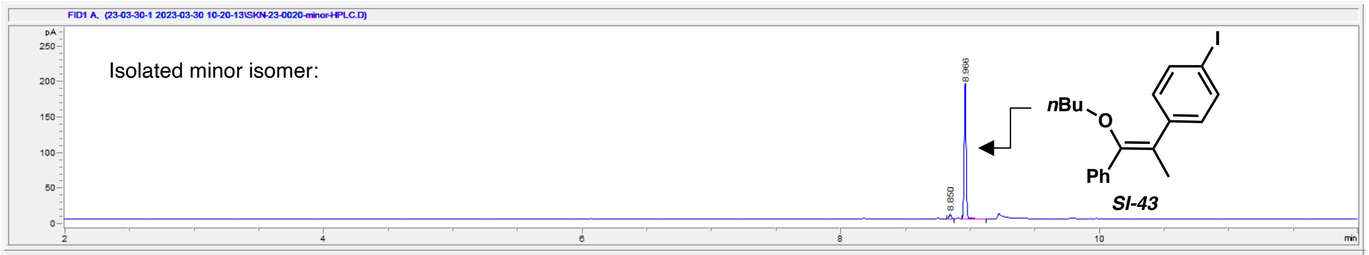

File Information

GC-File	SKN-23-0020-minor-HPLC.D
File Path	C:\Chem32\1\Data\23-03-30-1 2023-03-30 10-20-13

#	Time	Area	Height	Width	Area%	Symmetry
1	8.85	6.6	6.2	0.0174	3.288	0.91
2	8.966	193.9	187.5	0.0161	96.712	0.972

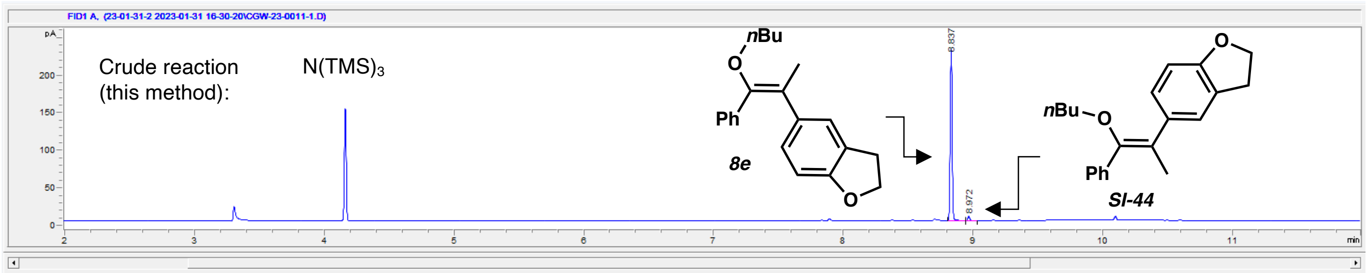

File Information	
GC-File	CGW-23-0011-1.D
File Path	C:\Chem32\1\Data\23-01-31-2 2023-01-31 16-30-20

#	Time	Area	Height	Width	Area%	Symmetry
1	8.837	237.3	226.4	0.0162	97.502	0.979
2	8.972	6.1	5.2	0.0188	2.498	0.888

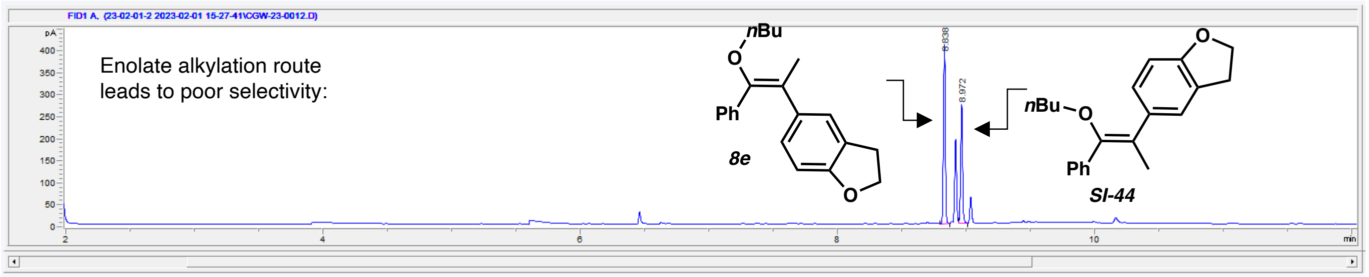

File Information	
GC-File	CGW-23-0012.D
File Path	C:\Chem32\1\Data\23-02-01-2 2023-02-01 15-27-41

#	Time	Area	Height	Width	Area%	Symmetry
1	8.838	420.7	401.2	0.0162	59.560	1.001
2	8.972	285.6	267.4	0.0165	40.440	0.97

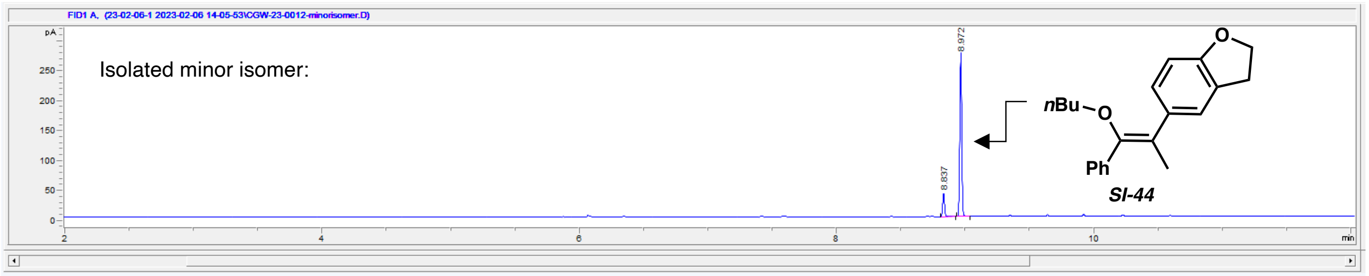

File Information	
GC-File	CGW-23-0012-minorisomer.D
File Path	C:\Chem32\1\Data\23-02-06-1 2023-02-06 14-05-53

#	Time	Area	Height	Width	Area%	Symmetry
1	8.837	38.6	37.8	0.0159	12.096	0.9
2	8.972	280.4	269.7	0.0171	87.904	0.994

7- Mechanistic Studies

7.1 Support for vinyl cation intermediacy:

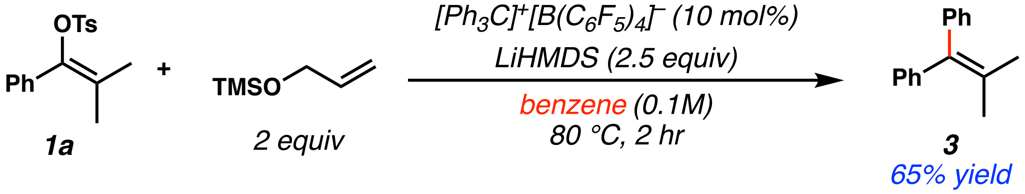

The following mechanistic experiment was performed on a 0.050 mmol scale inside a glovebox. To a dram vial equipped with a magnetic stir bar was added $[Ph_3C]^+[B(C_6F_5)_4]^-$ catalyst (4.6 mg, 0.005 mmol, 10 mol%), followed by LiHMDS (20.9 mg, 0.125 mmol, 2.5 equivalents), followed by benzene solvent (0.5 mL, 0.1 M). To this mixture was then added neat silyl allyl ether (13.0 mg, 0.100 mmol, 2.0 equivalents) followed by solid vinyl tosylate **1a** (15.1 mg, 0.050 mmol, 1 equivalent). The reaction was then sealed with a Teflon cap and heated in a reaction block for 2 hours at 80 ºC. Once the reaction was complete, the vial was removed from the glovebox. The reaction was diluted with diethyl ether and filtered through a pad of silica gel (pushing through with diethyl ether) and concentrated *in vacuo*. The yield of previously reported **3** was determined to be 65% yield by qNMR using nitromethane as an internal standard.

7.2 Neutral vs cationic Claisen rearrangement:

Allyl vinyl ether **5** was prepared according to a known literature procedure[19]:

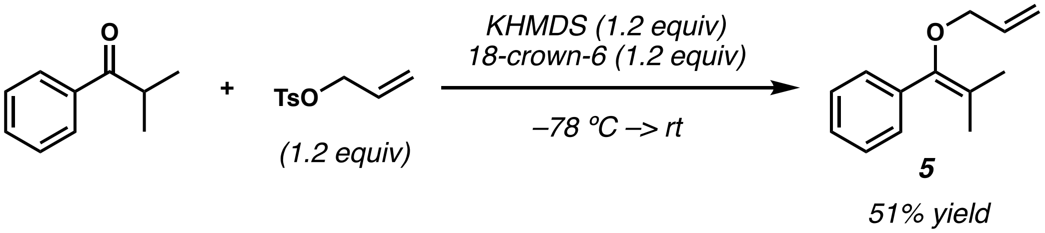

To a flame dried flask, 18-crown-6 (2.14 g, 1.2 equiv, 8.10 mmol) was added followed by THF (34 mL, 0.24 M). The solution was sparged with argon for 10 minutes. To another flame dried flask, KHMDS (1.62 g, 1.2 equiv, 8.10 mmol) was added inside of a glovebox and then brought outside of the glovebox. Toluene (16.2 mL, 0.5 M) was then added to the KHMDS, and this solution was then sparged with argon for 10 minutes. The solution of 18-crown-6 was then cooled to –78 ºC and the KHMDS solution was then added. Next, 2-methyl-1-phenylpropan-1-one was added dropwise. The yellow solution was allowed to stir at –78 ºC for 1 hour. Then, allyl 4-methylbenzenesulfonate (1.86 g, 1.3 equiv, 8.77 mmol) was added dropwise at –78 ºC. The reaction was allowed to slowly warm up to room temperature and stirred overnight. The reaction

was then analyzed by TLC (5% diethyl/hexanes), which showed complete consumption of starting material with one major, more polar spot formed. The reaction was then worked up by first cooling to 0 ºC and adding saturated NH_4Cl. Diethyl ether was then added, and the reaction was extracted 3x. The combined organic layers were dried with $MgSO_4$ and concentrated in vacuo. The crude material was then purified by column chromatography in 3% diethyl/ether with 0.1% triethylamine. Compound **5** was obtained as a pure oil (650 mg, 51% yield).

^{1}H NMR (400 MHz, C_6D_6) δ 7.41 – 7.31 (m, 2H), 7.17 – 7.11 (m, 3H), 7.09 – 7.03 (m, 1H), 5.83 (ddt, *J* = 17.2, 10.7, 5.4 Hz, 1H), 5.17 (dq, *J* = 17.2, 1.8 Hz, 1H), 5.00 (dq, *J* = 10.4, 1.5 Hz, 1H), 3.91 (dt, *J* = 5.4, 1.6 Hz, 2H), 1.92 (s, 3H), 1.62 (s, 3H).

^{13}C NMR (101 MHz, C_6D_6) δ 148.3, 136.4, 135.3, 129.9, 128.3, 127.7, 116.2, 115.7, 70.1, 19.8, 18.1.

FT-IR (neat film NaCl): 3080, 3059, 3021, 2987, 2913, 2857, 1672, 1647, 1600, 1490, 1443, 1421, 1381, 1287, 1214, 1136, 1072, 1047, 1024, 985, 920, 883, 843, 775, 700, 559, 419 cm^{-1}.

HR-MS (FI^+) m/z: [M] Calculated for $C_{13}H_{16}O$ 188.1201; Found 188.1203.

Neutral Claisen test:

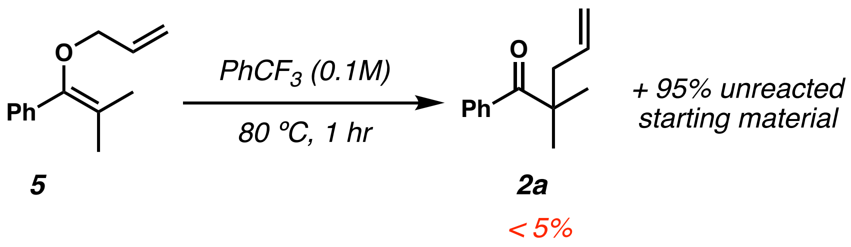

To a dram vial equipped with a magnetic stir bar inside the glovebox was added allyl vinyl ether **5** (9.5 mg, 0.050 mmol, 1 equivalent) followed by trifluorotoluene (0.5 mL, 0.1 M). The reaction was then sealed with a Teflon cap and heated in a reaction block at 80 ºC for 1 hour. After the hour, the reaction was brought outside of the glovebox and concentrated *in vacuo*. By qNMR (nitromethane as internal standard), <5% of the rearranged product (**2a**) was obtained with 95% of the starting allyl vinyl ether **5** remaining.

Cationic Claisen test:

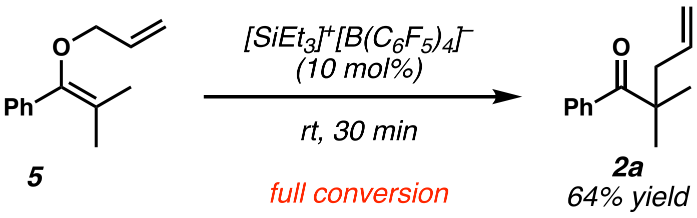

To a dram vial equipped with a magnetic stir bar inside the glovebox was added $[Ph_3C]^+[B(C_6F_5)_4]^-$ (4.6 mg, 0.005 mmol, 10 mol%). Trifluorotouene (0.5 mL, 0.1 M) was then added, followed by Et_3SiH (1.20 µL, 0.0075 mmol, 15 mol%). The mixture was then stirred at room temperature for 15 minutes. Then, allyl vinyl ether **5** (9.5 mg, 0.050 mmol, 1 equivalent) was added and the reaction was allowed to stir at room temperature for 30 minutes. The reaction was brought outside of the glovebox, filtered through a pad of silica gel and washed with diethyl ether, and concentrated *in vacuo*. By qNMR (nitromethane as internal standard), the starting allyl vinyl ether **5** was fully consumed and the rearranged product **2a** was observed in 64% yield.

7.3 Activation of allyl ether:

First, **(1-methoxy-2-methylprop-1-en-1-yl)benzene (4)** was prepared according to the procedure in section **5.3**, and 407 mg (84% yield) was obtained. Spectra matched the reported literature.[19]

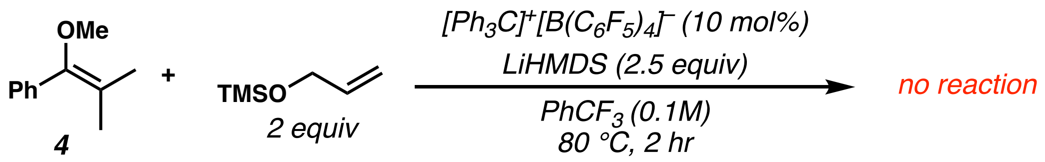

The above mechanistic experiment was performed on a 0.050 mmol scale inside a glovebox. To a dram vial equipped with a magnetic stir bar was added $[Ph_3C]^+[B(C_6F_5)_4]^-$ catalyst (4.6 mg, 0.005 mmol, 10 mol%), followed by LiHMDS (20.9 mg, 0.125 mmol, 2.5 equiv), followed by trifluorotoluene solvent (0.5 mL, 0.1 M). To this mixture was then added neat silyl allyl ether (13.0 mg, 0.100 mmol, 2.0 equiv) followed by **4** (8.11 mg, 0.050 mmol, 1 equivalent). The reaction was then sealed with a Teflon cap and heated in a reaction block for 2 hours at 80 ºC. After 2 hours, the reaction was removed from the glovebox. By TLC analysis, no new products were apparent (5% diethyl ether/hexanes).

7.4 Claisen rearrangement with deuterated allyl ether

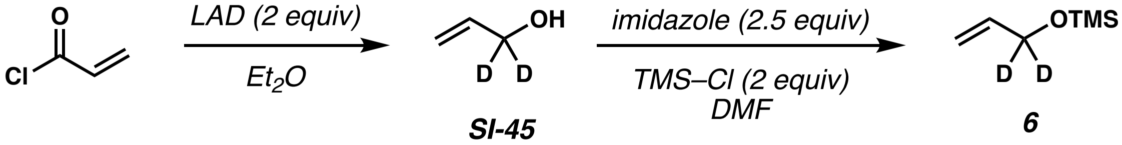

Allyl-1-d$_2$ alcohol (**SI-45**) was first prepared according to a known literature procedure.[20] To a flame dried flask was added LAD (2.52 g, 0.64 equiv, 60.1 mmol) followed by anhydrous diethyl ether (130 mL), and this solution was cooled to 0 ºC. Neat acrolyl chloride (8.50 g, 1 equiv,

93.9 mmol) was added dropwise to the LAD solution. After the addition was complete, the reaction was allowed to warm up to room temperature and stirred for 3.5 hours, and at this time starting material appeared to be consumed by TLC (visualization by $KMnO_4$ stain). The reaction was cooled back to 0 ºC, and 2.5 mL of 15% aq. NaOH was added dropwise. Then, 7.5 mL of H_2O was added dropwise. The reaction was then allowed to warm to room temperature and stir for an additional 15 minutes. $MgSO_4$ was added and stirred for 15 additional minutes. The reaction was then sonicated for 10 minutes and filtered. The solids were washed with twice with 25 mL of diethyl ether. The crude reaction was then carried forward to the protection step.

Next, **((allyl-1,1-*d*2)oxy)trimethylsilane (6)** was prepared by the procedure for silyl ethers in section **3** with slight modification. Assuming quantitative yield from the previous step, imidazole (15.2 g, 2.5 equiv, 223 mmol) was added to the allyl-1-d_2 alcohol in ether. The reaction was cooled to 0 ºC and distilled TMSCl (24 mL, 2.0 equiv, 178 mmol) was added dropwise. After addition was complete, the reaction was warmed up to room temperature and stirred until starting material had been fully consumed (~2 hours). 25 mL water was charged dropwise to quench residual TMSCl. After stirring for 10 minutes, the aqueous layer was extracted with 3 x 25 mL pentane. The combined organic layers were washed with brine and dried over Na_2SO_4. Due to the volatility of the product, the crude mixture was concentrated in vacuo at 0 ºC to approximately ~50mL. Then, the compound was purified via fractional distillation at 135 ºC.

^{1}H NMR (400 MHz, $CDCl_3$) δ 5.92 (dd, *J* = 17.1, 10.4 Hz, 1H), 5.25 (dd, *J* = 17.1, 1.8 Hz, 1H), 5.10 (dd, *J* = 10.4, 1.8 Hz, 1H), 0.13 (s, 9H).

^{13}C NMR (101 MHz, $CDCl_3$) δ 137.0, 114.6, -0.5.

HR-MS (ESI) m/z: [M+H]+ Calculated for $C_6H_{12}D_2OSi$ 132.0939; Found 132.0937.

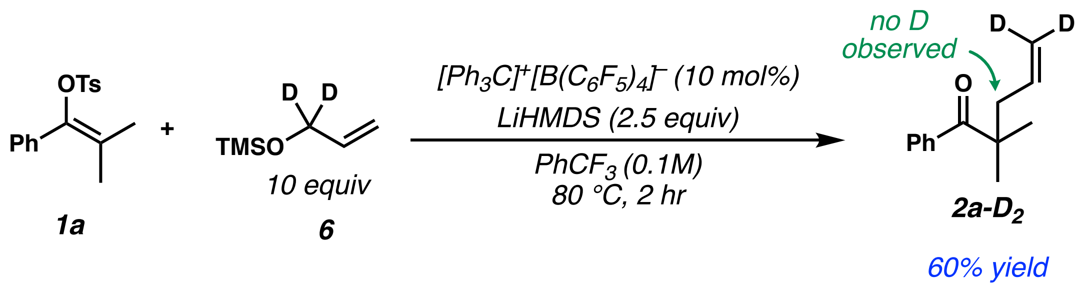

1a *6* *2a-D₂*

60% yield

The above mechanistic experiment was performed on a 0.050 mmol scale inside a glovebox. To a dram vial equipped with a magnetic stir bar was added $[Ph_3C]^+[B(C_6F_5)_4]^-$ catalyst (4.6 mg, 0.0050 mmol, 10 mol%), followed by LiHMDS (20.9 mg, 0.125 mmol, 2.5 equivalents), followed by trifluorotoluene solvent (0.5 mL, 0.1 M). To this mixture was then added **6** (66.1 mg,

0.500 mmol, 10.0 equivalents) followed by vinyl tosylate **1a** (15.1 mg, 0.05 mmol, 1 equivalent). The reaction was then sealed with a Teflon cap and heated in a reaction block for 2 hours at 80 ºC. After 2 hours, the reaction was removed from the glovebox, and the crude reaction mixture was filtered through pad of silica gel with diethyl ether and concentrated in vacuo. By qNMR, product **2a-D$_2$** was obtained in 60% yield with no observation of other deuterated isomers.

^{1}H NMR (400 MHz, $CDCl_3$) δ 7.69 – 7.62 (m, 2H), 7.48 – 7.36 (m, 3H), 5.77 – 5.65 (m, 1H), 2.49 (d, *J* = 7.3 Hz, 2H), 1.32 (s, 6H).

^{13}C NMR (101 MHz, $CDCl_3$) δ 209.0, 139.2, 134.0, 130.9, 128.2, 127.8, 47.8, 45.0, 25.9.

FT-IR (neat film NaCl): 2964, 2927, 2361, 2342, 1675, 1467, 1445, 1213, 960, 732, 701, 520, 505, 492, 459, 442, 427, 415 cm^{-1}.

HR-MS (FI$^+$) m/z: [M] Calculated for $C_{13}H_{14}D_2O$ 190.1327; Found 190.1328.

7.5 Product distribution of prenyl ether:

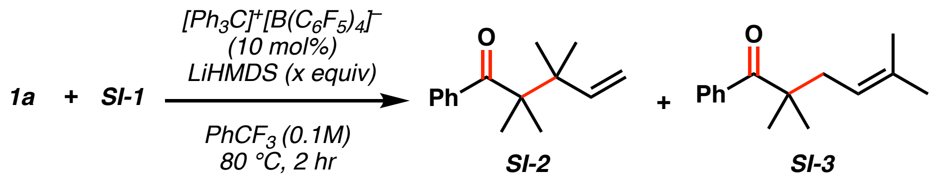

LiHMDS equiv:		SI-2		SI-3
	2.5	1	:	11.1
	5	1	:	6.8
	10	1	:	4.0

The following mechanistic experiments were performed on a 0.050 mmol scale inside a glovebox in parallel. To a dram vial equipped with a magnetic stir bar was added $[Ph_3C]^+[B(C_6F_5)_4]^-$ catalyst (4.6 mg, 0.0050 mmol, 10 mol%), followed by LiHMDS (2.5, 5, or 10 equivalents), followed by trifluorotoluene solvent (0.5 mL, 0.1 M). To this mixture was then added neat **prenyl silyl ether SI-1 (**15.8 mg, 0.100 mmol, 2.0 equivalents) followed by vinyl tosylate **SI-4/1a** (15.1 mg, 0.050 mmol, 1 equivalent). The reaction was then sealed with a Teflon cap and heated in a reaction block for 2 hours at 80 ºC. After 2 hours, the reactions were removed from the glovebox, filtered through a pad silica with diethyl ether, and concentrated in vacuo. By qNMR, the ratio between the two products was determined to be 1:11.1 ***SI-2**-to-**SI-3*** for 2.5 equivalents of LiHMDS, 1:6.8 for 5 equivalents of LiHMDS, and 1:4.0 for 10 equivalents of LiHMDS.

The following NMR spectrum is the purified mixture of prenyl products obtained by using **silyl ether SI-1**. Note, they are inseparable by HPLC (80:20 MeCN:H_2O):

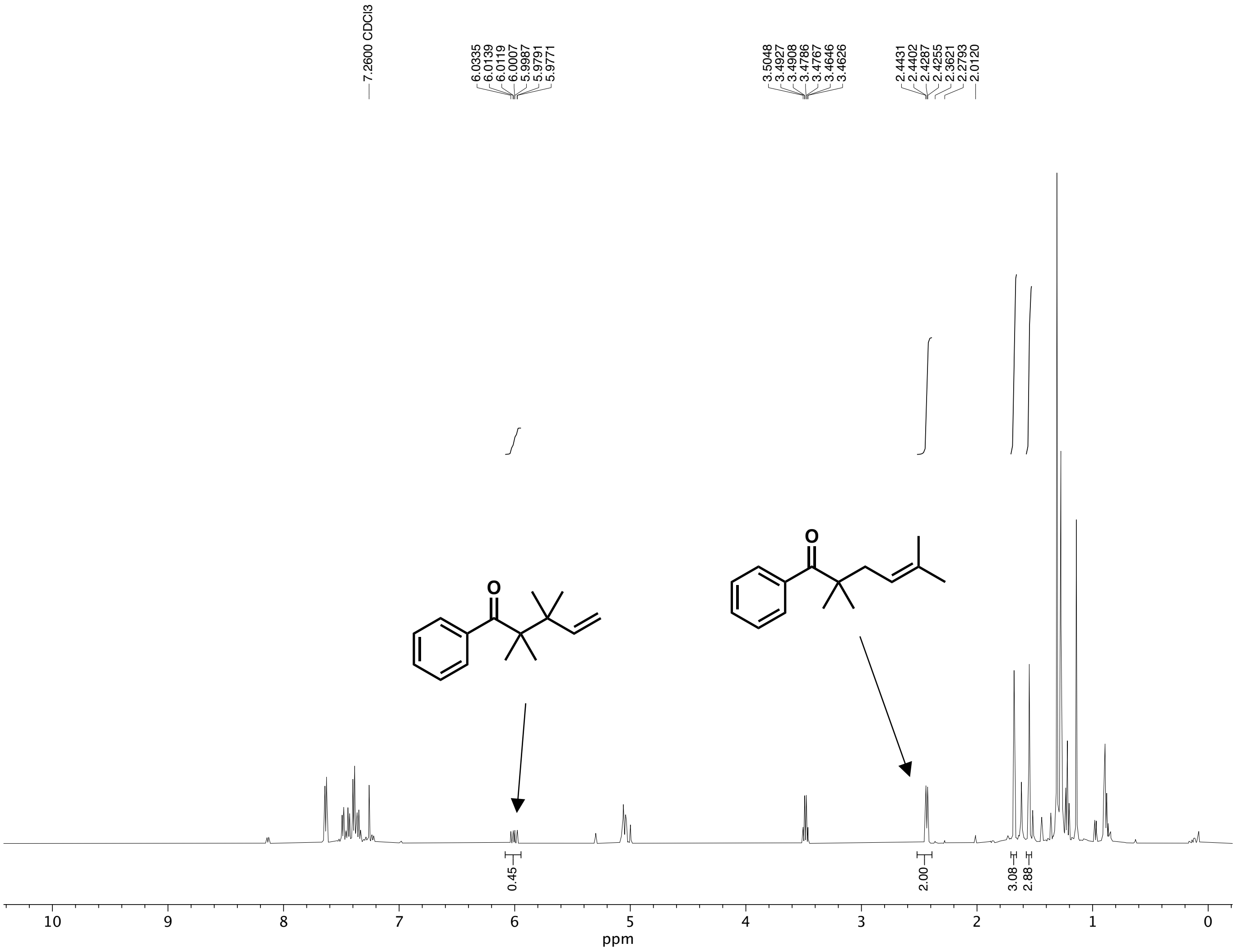

The following NMR spectrum is the crude reaction mixture using **silyl ether SI-1** and **2.5 equivalents LiHMDS** referenced to nitromethane as the internal standard:

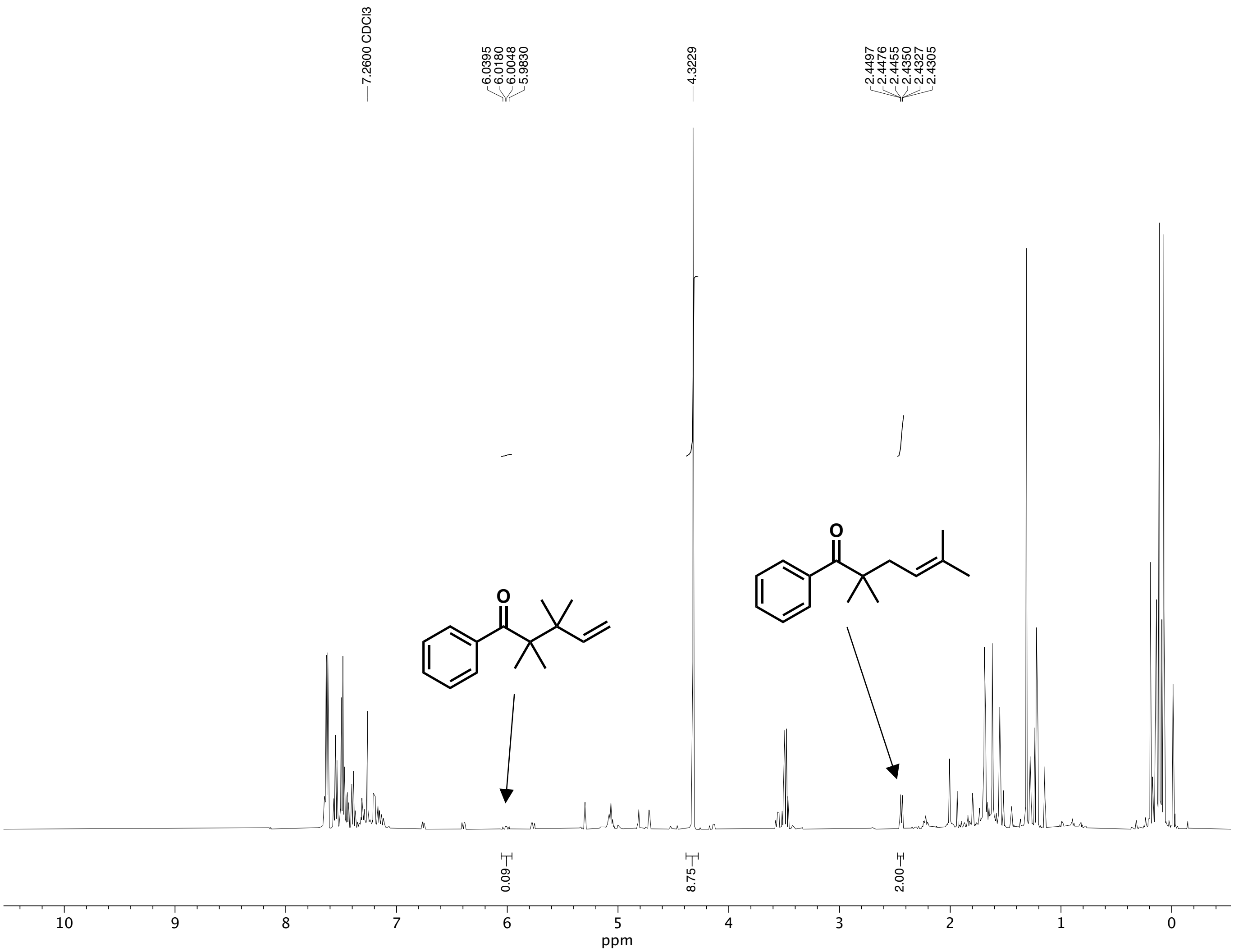

The following NMR spectrum is the crude reaction mixture using **silyl ether SI-1** and **5.0 equivalents LiHMDS** referenced to nitromethane as the internal standard:

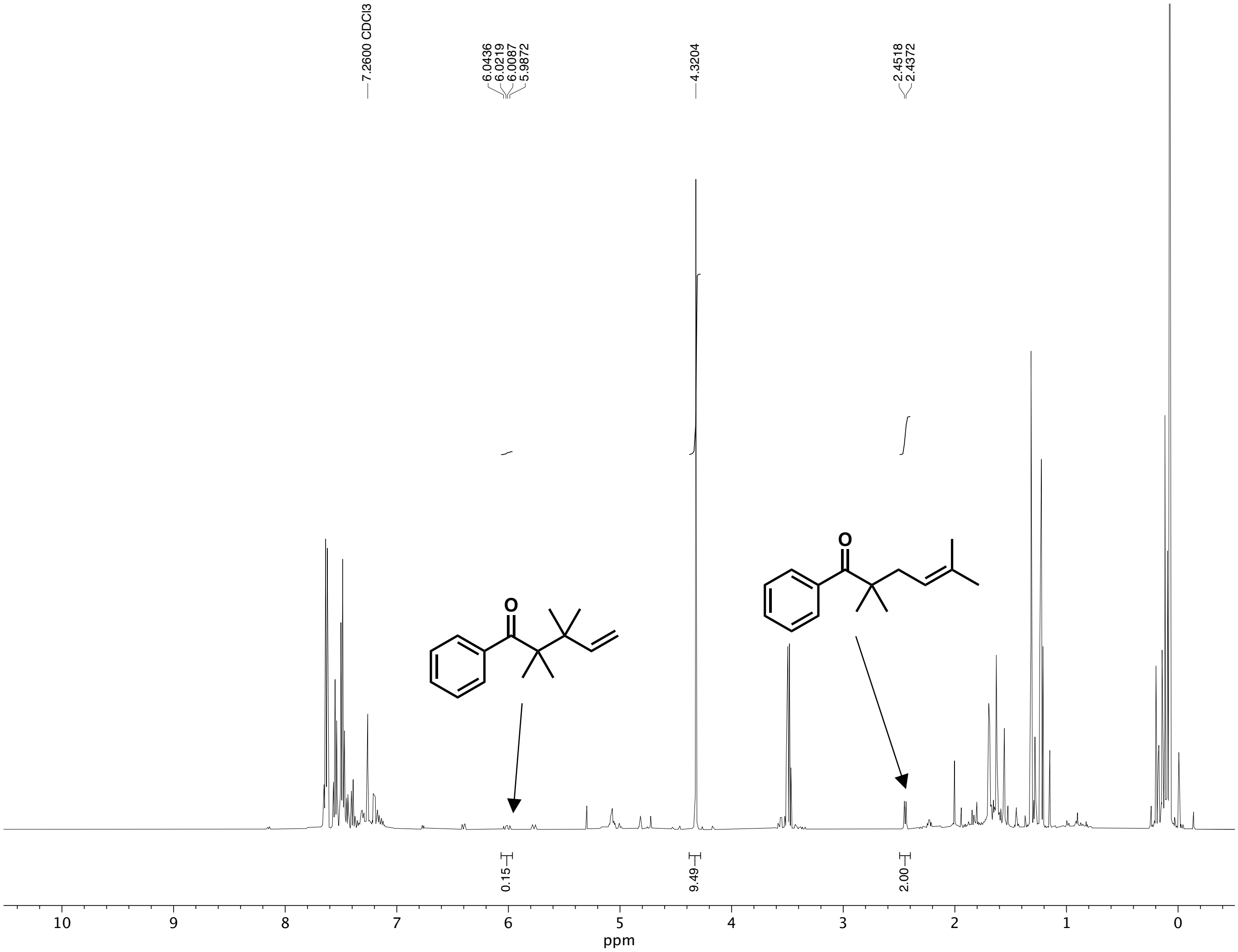

The following NMR spectrum is the crude reaction mixture using **silyl ether SI-1** and **10.0 equivalents LiHMDS** referenced to nitromethane as the internal standard:

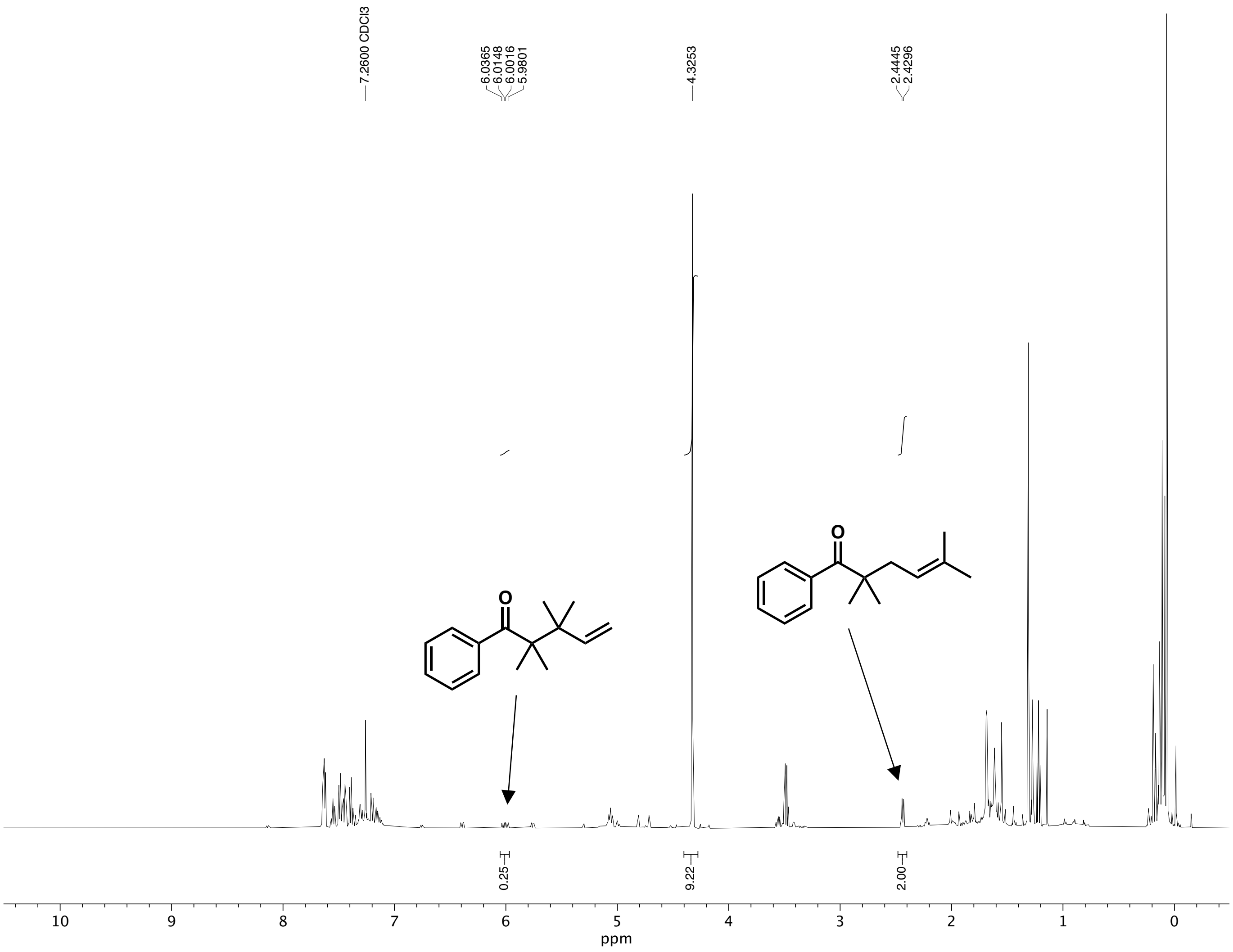

8- Computational Studies

8.1 Computational Methods

Density functional theory (DFT) computations were performed with Gaussian 16.[21] The ωB97X-D functional was used to optimize molecular geometries.[21] Geometry optimizations were completed with the def2-SVP basis set[22] and the cyclohexane CPCM solvation model accounting for the effect of solvation.[23] Frequency calculations were conducted at the same level of theory used for the geometry optimizations in order to obtain thermal Gibbs free energies and characterize the stationary points on the potential energy surface. Single point energies were obtained using the def2-TZVPP basis set.[22] Intrinsic reaction coordinate (IRC) calculations were performed to verify that a transition state (TS) connects the reactant and the product on the potential energy surface.

Conformation searches were executed by Grimme's Conformer-Rotamer Ensemble Sampling Tool (CREST) to find the lowest energy conformers.[24] CYLview was employed to visualize molecular structures.[25]

8.2 Calculated Energetics

Table S1. Energies of the optimized structures (ωB97X-D/def2-SVP/CPCM=cyclohexane)

Structure	E	ZPE	H	T.S	G(T)
INT1'	-988.484919	0.37131	-988.082421	0.096549	-988.178971
INT2'	-988.521545	0.37187	-988.118907	0.095439	-988.214346
TS1'	-988.461358	0.369156	-988.061705	0.093653	-988.155358
INT1	-579.588595	0.257201	-579.311026	0.073081	-579.384107
INT2	-579.625977	0.258179	-579.347889	0.071147	-579.419036
TS1	-579.545896	0.256513	-579.270457	0.06783	-579.338286
INT1a	-1067.050019	0.42804	-1066.587237	0.10289	-1066.690127
INT2a	-1067.035789	0.425117	-1066.575299	0.105027	-1066.680326
INT3a	-1067.067299	0.429182	-1066.604238	0.100643	-1066.704881
TS1a	-1067.035205	0.425039	-1066.575434	0.104008	-1066.679442
TS2a	-1067.035211	0.424894	-1066.575672	0.103476	-1066.679148
INT2a'	-1067.08363	0.428381	-1066.620624	0.10373	-1066.724353
TS1a'	-1067.021743	0.422658	-1066.563292	0.108866	-1066.672158

Table S2. Single point energies (ωB97X-D/def2-TZVPP/CPCM=cyclohexane)

Structure	E
SP_INT1'	-989.344287
SP_INT2'	-989.381506
SP_TS1'	-989.322652
SP_INT1	-580.214446
SP_INT2	-580.250791
SP_TS1	-580.171564
SP_INT1a	-1067.991952
SP_INT2a	-1067.982181
SP_INT3a	-1068.010092
SP_TS1a	-1067.980259

SP_TS2a	-1067.98086
SP_INT2a'	-1068.026655
SP_TS1a'	-1067.970848

8.3 Calculated Cartesian coordinates

INT1'
Charge: 1

C	2.638638	0.540844	-0.021775
C	3.850668	-0.113993	0.185836
C	3.912703	-1.505475	0.122999
C	2.759786	-2.240784	-0.153523
C	1.549765	-1.586139	-0.367969
C	1.475874	-0.187626	-0.308020
C	0.194080	0.528228	-0.503679
C	-0.091381	1.528189	-1.348205
C	-1.388534	2.282412	-1.338198
H	-1.179275	3.362912	-1.332759
H	-2.021367	2.053551	-0.474300
H	-1.959172	2.079333	-2.258619
C	0.892800	1.961206	-2.397488
H	0.382287	2.034570	-3.369966
H	1.277256	2.966360	-2.162975
H	1.743399	1.277391	-2.499258
O	-0.833165	0.099178	0.411922
C	-0.554421	0.367181	1.842954
C	-0.399108	1.827304	2.088725
C	0.733998	2.361197	2.542968
H	0.811535	3.429369	2.757329
H	1.620940	1.747361	2.728341
H	-1.280833	2.452087	1.913731
H	-1.419584	-0.048000	2.375662
H	0.343700	-0.208673	2.104777
Si	-2.211039	-1.044456	-0.051632
C	-3.744615	-0.122520	0.434189
H	-4.602119	-0.804379	0.312564
H	-3.923116	0.756538	-0.200788
H	-3.729616	0.195549	1.487269
C	-1.918405	-2.558224	0.981370
H	-2.651226	-3.321893	0.673284
H	-2.072886	-2.372250	2.054183
H	-0.914348	-2.982437	0.840530
C	-1.999949	-1.293747	-1.872563
H	-2.763520	-2.021067	-2.194177
H	-2.161610	-0.365677	-2.437935
H	-1.014951	-1.701270	-2.139939
H	0.661235	-2.175140	-0.602593

H	2.804564	-3.329921	-0.210718
H	4.861450	-2.019168	0.289742
H	4.749333	0.465725	0.404898
H	2.586387	1.629772	0.043477

There are no imaginary frequencies

INT1
Charge: 0

C	2.256792	0.717145	-0.594210
C	1.207104	-0.058485	-0.082023
C	1.512855	-1.320621	0.450329
C	2.827912	-1.773720	0.506949
C	3.865895	-0.979811	0.016972
C	3.574225	0.264375	-0.539632
H	2.036672	1.677803	-1.063298
H	0.708877	-1.955181	0.827380
H	3.044478	-2.755220	0.934620
H	4.897519	-1.336112	0.058293
H	4.376097	0.883853	-0.947581
C	-0.207924	0.395411	-0.149157
C	-0.657719	1.643762	0.098463
C	-2.092714	2.023352	-0.153581
H	-2.617140	1.286841	-0.775606
H	-2.651841	2.139342	0.791140
H	-2.133744	3.000313	-0.662168
C	0.196008	2.749104	0.660143
H	-0.355752	3.267456	1.461516
H	1.145350	2.392000	1.078602
H	0.422311	3.509714	-0.106347
C	-2.005215	-1.092843	0.392878
H	-1.557886	-1.950639	0.931849
H	-2.250438	-0.326402	1.146118
C	-3.239259	-1.541181	-0.327119
H	-3.072352	-2.233157	-1.160399
C	-4.475101	-1.161470	-0.006685
H	-5.346732	-1.535790	-0.549342
H	-4.662771	-0.463638	0.816013
O	-1.069975	-0.598911	-0.552593

There are no imaginary frequencies

INT1a
Charge: 0

C	-2.197310	-0.779232	0.968325
C	-3.250339	-1.614336	0.606025

C	-3.036897	-2.664319	-0.286447
C	-1.762508	-2.878073	-0.810787
C	-0.705845	-2.051616	-0.436445
C	-0.907169	-0.996003	0.463639
C	0.192022	-0.082688	0.841884
C	0.503548	0.413975	2.048429
C	-0.148120	-0.088455	3.305613
H	-0.703676	-1.022443	3.161571
H	-0.836125	0.669954	3.711988
H	0.625405	-0.255977	4.070846
C	1.534928	1.485866	2.252187
H	1.803875	2.009144	1.327771
H	2.450716	1.067274	2.701196
H	1.149556	2.227902	2.967351
O	0.991087	0.338494	-0.274690
C	0.295010	1.129038	-1.344063
C	-0.613230	2.135571	-0.750566
C	-1.948389	2.175116	-0.913501
C	-2.751631	3.280974	-0.291301
H	-2.123325	4.009092	0.238312
H	-3.480753	2.860711	0.420260
H	-3.334165	3.812701	-1.060298
C	-2.750511	1.190810	-1.716930
H	-3.091143	1.665901	-2.651259
H	-3.653993	0.901891	-1.160035
H	-2.209900	0.271121	-1.973896
H	-0.128868	2.922771	-0.164127
H	1.123573	1.596475	-1.892247
H	-0.205087	0.398440	-1.989883
Si	2.667970	-0.347297	-0.626019
C	3.810165	1.111627	-0.551055
H	4.793180	0.790332	-0.932330
H	3.951186	1.482423	0.473245
H	3.470196	1.943096	-1.186367
C	2.928469	-1.615693	0.696443
H	3.878372	-2.131176	0.479273
H	2.131739	-2.372970	0.717709
H	3.010665	-1.164298	1.694810
C	2.526193	-1.040396	-2.343190
H	2.346260	-0.259638	-3.096449
H	3.490400	-1.514857	-2.588764
H	1.746259	-1.808712	-2.439992
H	0.285717	-2.241937	-0.850042
H	-1.587306	-3.697343	-1.510507
H	-3.864783	-3.313394	-0.577953
H	-4.248133	-1.433013	1.010146
H	-2.380437	0.067742	1.631904

There are no imaginary frequencies

INT2'
Charge: 1

C	2.265318	-0.448122	0.636903
C	3.437267	0.234890	0.938005
C	3.726843	1.446017	0.307009
C	2.835903	1.986985	-0.620305
C	1.642861	1.330847	-0.896631
C	1.347809	0.106145	-0.271073
C	0.069903	-0.533220	-0.602942
O	-0.965175	0.175018	-0.672943
Si	-1.690873	1.668985	0.057451
C	-3.374186	1.029744	0.493266
H	-3.889408	0.630527	-0.393081
H	-3.313554	0.239251	1.255033
H	-3.984781	1.850848	0.901117
C	-1.705846	2.902217	-1.327617
H	-0.715192	3.333208	-1.529679
H	-2.098066	2.454529	-2.253164
H	-2.377525	3.729943	-1.048231
C	-0.652827	2.112333	1.529952
H	0.329514	2.522329	1.257696
H	-0.506770	1.240348	2.185348
H	-1.194872	2.878672	2.108034
C	-0.098964	-2.005404	-0.906992
C	-1.345694	-2.205456	-1.783378
H	-2.262700	-1.850591	-1.297273
H	-1.459161	-3.277050	-1.997248
H	-1.241522	-1.676391	-2.741635
C	1.133230	-2.578475	-1.623698
H	0.952708	-3.639070	-1.848094
H	1.313818	-2.057838	-2.575441
H	2.045828	-2.514032	-1.017807
C	-0.295827	-2.740775	0.466354
C	-1.346403	-2.157805	1.366127
C	-1.095422	-1.606681	2.554721
H	-1.899364	-1.209365	3.179406
H	-0.079913	-1.550958	2.960948
H	-2.381694	-2.205158	1.010301
H	-0.566418	-3.775120	0.197877
H	0.662229	-2.805858	1.000734
H	0.952717	1.747244	-1.632145
H	3.070325	2.923912	-1.127828
H	4.657355	1.968756	0.536214
H	4.133241	-0.183647	1.666418
H	2.052673	-1.389523	1.140990

There are no imaginary frequencies

INT2
Charge: 0

C	1.277141	-0.461600	-1.120321
C	0.823039	-0.521064	0.203564
C	1.753300	-0.347298	1.238747
C	3.092439	-0.093663	0.961967
C	3.531263	-0.030370	-0.361115
C	2.622108	-0.223778	-1.399602
H	0.593350	-0.611744	-1.954054
H	1.399774	-0.406899	2.269144
H	3.798631	0.053756	1.781793
H	4.582628	0.165867	-0.582629
H	2.959349	-0.189629	-2.437582
C	-0.604402	-0.772636	0.629372
C	-1.809004	-0.400284	-0.262845
C	-3.071274	-0.422913	0.610023
H	-2.989020	0.257094	1.468945
H	-3.942918	-0.123745	0.008086
H	-3.252762	-1.429213	1.009994
C	-1.979830	-1.461073	-1.368358
H	-2.903985	-1.255405	-1.930126
H	-1.153069	-1.476640	-2.090224
H	-2.067791	-2.466912	-0.931123
C	-0.226799	2.763609	0.224473
H	-0.098995	3.549949	0.972759
H	0.634252	2.534949	-0.412400
C	-1.379164	2.104445	0.097586
H	-2.209863	2.361822	0.764887
C	-1.629348	1.004983	-0.896371
H	-2.554203	1.222506	-1.458112
H	-0.816133	0.993019	-1.635007
O	-0.797007	-1.251116	1.726616

There are no imaginary frequencies

INT2a'
Charge: 1

C	-1.953150	-1.378392	-0.644723
C	-3.118770	-1.325382	-1.398350
C	-3.959181	-0.213901	-1.314598
C	-3.629688	0.855278	-0.481031
C	-2.446207	0.825726	0.245564
C	-1.593432	-0.290934	0.169080
C	-0.354650	-0.255135	0.953427
O	0.289468	0.822862	1.021291
Si	0.692176	2.283671	0.033743

C	0.212178	1.872309	-1.711184
H	-0.876109	1.854678	-1.862975
H	0.627244	0.897207	-2.006648
H	0.639454	2.637128	-2.379713
C	2.519620	2.378232	0.316691
H	3.025759	1.481569	-0.066530
H	2.930946	3.254885	-0.208131
H	2.747639	2.484077	1.387844
C	-0.235635	3.683923	0.822367
H	0.212562	4.630259	0.478874
H	-1.300676	3.704517	0.552270
H	-0.143342	3.653707	1.918640
C	0.209669	-1.422500	1.728045
C	1.092379	-0.896047	2.871178
H	0.503323	-0.292114	3.576545
H	1.925223	-0.280833	2.510122
H	1.506606	-1.752681	3.420454
C	-0.900571	-2.311168	2.310067
H	-1.551079	-1.738420	2.987132
H	-1.529451	-2.771945	1.538251
H	-0.437805	-3.120577	2.891835
C	1.087425	-2.263125	0.728476
C	2.132445	-1.498058	-0.029323
C	2.260008	-1.382035	-1.362456
C	1.342334	-2.004490	-2.380262
H	0.559196	-2.638053	-1.947867
H	0.854428	-1.227521	-2.992109
H	1.924185	-2.626870	-3.078116
C	3.404843	-0.606445	-1.958891
H	4.085772	-0.211553	-1.192256
H	3.992101	-1.245953	-2.637074
H	3.035176	0.236245	-2.567189
H	2.896980	-1.013566	0.588327
H	1.577753	-3.017118	1.367122
H	0.435459	-2.829185	0.052030
H	-2.200468	1.653832	0.911649
H	-4.296933	1.714427	-0.397484
H	-4.881642	-0.185378	-1.897629
H	-3.376889	-2.160328	-2.051277
H	-1.309324	-2.251858	-0.723293

There are no imaginary frequencies

INT2a
Charge: 1

C	-0.090717	2.409356	1.214447
C	-0.110632	1.417415	0.222438
C	-0.440921	1.796938	-1.088721

C	-0.688307	3.126961	-1.410645
C	-0.635261	4.106926	-0.418975
C	-0.348422	3.741243	0.894849
H	0.090926	2.141363	2.254974
H	-0.525087	1.034616	-1.864169
H	-0.935362	3.399272	-2.438606
H	-0.835307	5.151044	-0.667301
H	-0.336320	4.496171	1.683358
C	0.123391	-0.018223	0.532727
C	1.018300	-0.518585	1.439683
C	1.000242	-1.974797	1.810306
H	0.388427	-2.580145	1.132478
H	2.017804	-2.394281	1.844147
H	0.594195	-2.083280	2.830538
C	1.949465	0.332000	2.257128
H	2.875875	-0.222545	2.468506
H	2.212667	1.281475	1.771650
H	1.507706	0.569945	3.239168
C	0.792244	-1.745523	-2.018504
H	0.166152	-2.619106	-2.207425
H	0.516450	-0.826005	-2.534131
C	1.922410	-1.856571	-1.262794
H	2.152526	-2.822263	-0.807085
C	2.746856	-0.768078	-0.917023
O	-0.609425	-0.879927	-0.203387
C	2.662389	0.578986	-1.531787
H	1.841779	0.711782	-2.241278
H	2.582691	1.347266	-0.746841
H	3.616542	0.770075	-2.051316
C	3.931769	-1.017041	-0.058084
H	4.761456	-1.287386	-0.737836
H	4.247507	-0.117373	0.485727
H	3.791584	-1.859775	0.630553
Si	-2.297425	-1.241465	-0.002957
C	-2.599002	-1.310595	1.835582
H	-2.347409	-0.349372	2.310873
H	-3.662943	-1.513879	2.035090
H	-2.005766	-2.102580	2.316199
C	-3.319097	0.084204	-0.822469
H	-3.177267	1.063068	-0.340726
H	-3.072692	0.185197	-1.890403
H	-4.386070	-0.181325	-0.746329
C	-2.485168	-2.892430	-0.845263
H	-2.317458	-2.816982	-1.930838
H	-1.790801	-3.638955	-0.429912
H	-3.509681	-3.269236	-0.697948

There are no imaginary frequencies

INT3a
Charge: 1

C	0.554249	2.211376	-1.256303
C	0.395925	1.232737	-0.265438
C	0.908984	1.449777	1.024191
C	1.546772	2.647129	1.324694
C	1.716167	3.614207	0.332688
C	1.231288	3.389878	-0.955355
H	0.196004	2.042250	-2.270761
H	0.766107	0.693703	1.797553
H	1.919332	2.823620	2.334993
H	2.230977	4.548294	0.565056
H	1.377215	4.139814	-1.734342
C	-0.230537	-0.074975	-0.540025
C	-1.662705	-0.287327	-0.971901
C	-1.772563	-1.671982	-1.634260
H	-1.036413	-1.762624	-2.446139
H	-1.600189	-2.498361	-0.933801
H	-2.767972	-1.793711	-2.077711
C	-2.125399	0.770868	-1.985373
H	-3.189616	0.613259	-2.205379
H	-2.007992	1.800643	-1.631476
H	-1.580490	0.659255	-2.934256
C	-1.490144	-0.610348	2.686471
H	-1.048559	-1.308491	3.401667
H	-1.612599	0.422433	3.022085
C	-1.870328	-1.027574	1.478359
H	-1.721933	-2.085629	1.232724
C	-2.542441	-0.205590	0.391630
O	0.444191	-1.110959	-0.327344
C	-2.773650	1.245155	0.830570
H	-1.844527	1.812214	0.979142
H	-3.391048	1.784310	0.100609
H	-3.325652	1.254617	1.780815
C	-3.920305	-0.848478	0.136137
H	-4.547934	-0.691346	1.025006
H	-4.435456	-0.389717	-0.720384
H	-3.855662	-1.931167	-0.033442
Si	2.157258	-1.637288	-0.041933
C	2.278960	-1.830778	1.798650
H	3.159886	-2.451279	2.029002
H	2.399730	-0.870575	2.319108
H	1.392548	-2.345502	2.198706
C	2.130541	-3.244976	-0.961930
H	1.376813	-3.928507	-0.543596
H	1.911475	-3.090217	-2.029045
H	3.114588	-3.733701	-0.885151
C	3.262972	-0.356474	-0.804212
H	2.975993	-0.149418	-1.846432

H	3.274254	0.588759	-0.243878
H	4.289257	-0.758963	-0.816424

There are no imaginary frequencies

TS1'
Charge: 1

C	2.268228	0.110811	-1.069299
C	1.359676	0.231517	-0.008651
C	1.772869	-0.146026	1.276532
C	3.066927	-0.607971	1.500290
C	3.964712	-0.715435	0.438354
C	3.560973	-0.358783	-0.847422
H	1.955429	0.371097	-2.082637
H	1.083073	-0.091499	2.119864
H	3.374011	-0.890684	2.508895
H	4.977525	-1.083547	0.612665
H	4.253900	-0.452120	-1.685711
C	-0.035915	0.673391	-0.278625
C	-0.393466	1.799695	-0.968600
C	-1.811000	2.036418	-1.399909
H	-1.862635	1.950487	-2.498242
H	-2.516370	1.318960	-0.966441
H	-2.137098	3.057421	-1.151836
C	0.616115	2.782873	-1.488562
H	0.228643	3.807236	-1.384244
H	1.584145	2.716329	-0.977209
H	0.784464	2.621683	-2.565897
C	-1.772478	0.548323	2.015033
H	-2.679497	-0.057993	2.009665
H	-0.917910	0.127250	2.544797
C	-1.839614	1.921081	1.750774
H	-2.790621	2.359234	1.440968
C	-0.679657	2.626881	1.607445
O	-1.014763	-0.117757	0.228577
Si	-1.319927	-1.778986	-0.326397
C	-3.083078	-2.086608	0.180327
H	-3.424699	-3.020323	-0.294704
H	-3.196833	-2.218537	1.266945
H	-3.752021	-1.280145	-0.156960
C	-1.087774	-1.696887	-2.169052
H	-1.750398	-0.945579	-2.624413
H	-0.046616	-1.454327	-2.429610
H	-1.326345	-2.675648	-2.614450
C	-0.113155	-2.907141	0.524814
H	0.920403	-2.716542	0.200840
H	-0.161276	-2.798014	1.619006
H	-0.363127	-3.952169	0.279803

H	0.281567	2.220975	1.932628
H	-0.680824	3.661469	1.256538

1 imaginary frequency: –250.94 cm^{-1}

TS1
Charge: 0

C	1.780136	1.187138	-0.195180
C	0.988675	0.030507	-0.287615
C	1.631445	-1.211407	-0.152797
C	2.990644	-1.294860	0.136517
C	3.753418	-0.134424	0.264294
C	3.144039	1.105508	0.079560
H	1.343071	2.167891	-0.374532
H	1.057515	-2.124909	-0.306328
H	3.460238	-2.274744	0.248447
H	4.821324	-0.196842	0.484656
H	3.736514	2.021254	0.139298
C	-0.471588	0.056299	-0.622886
C	-1.369975	1.052228	-0.191088
C	-2.687152	1.178657	-0.919304
H	-2.942668	0.256273	-1.451692
H	-3.509062	1.440874	-0.235657
H	-2.608683	1.992557	-1.661032
C	-0.973821	2.299607	0.561308
H	-1.848515	2.690635	1.103272
H	-0.174067	2.124434	1.293031
H	-0.646567	3.100431	-0.123271
C	-1.713442	-2.143297	0.051998
H	-2.137321	-2.874471	-0.638858
H	-0.772604	-2.452264	0.512995
C	-2.567104	-1.306729	0.787513
H	-3.618030	-1.235065	0.493695
C	-1.999109	-0.268559	1.500678
H	-2.625209	0.486692	1.983202
H	-0.993705	-0.374592	1.919038
O	-0.947404	-1.006520	-1.183031

1 imaginary frequency: –581.29 cm^{-1}

TS1a'
Charge: 1

C	2.804536	-1.175724	0.808804
C	1.780851	-0.794546	-0.069323
C	2.096291	-0.615193	-1.424573
C	3.385147	-0.847370	-1.895424

C	4.390602	-1.245957	-1.013651
C	4.097723	-1.402661	0.340133
H	2.591578	-1.274302	1.875003
H	1.327566	-0.265309	-2.116691
H	3.609805	-0.708280	-2.954965
H	4.883427	-1.693693	1.040311
C	0.407571	-0.498549	0.425379
C	-0.298947	-1.248906	1.298327
C	-1.609256	-0.767871	1.856084
H	-1.536770	-0.652051	2.950744
H	-1.910137	0.198203	1.432796
H	-2.411351	-1.506892	1.684477
C	0.148991	-2.595339	1.799695
H	-0.693090	-3.305905	1.762680
H	0.975147	-3.019785	1.215964
H	0.467411	-2.545283	2.854485
C	-1.608261	-0.793016	-1.862468
H	-0.729384	-1.358172	-2.180974
C	-2.625096	-1.426506	-1.216049
H	-2.546573	-2.499131	-1.025022
C	-3.773670	-0.755050	-0.744246
O	-0.142040	0.645346	-0.102389
Si	0.364522	2.234953	0.302729
C	1.923941	2.682071	-0.621364
H	2.181970	3.735808	-0.427332
H	2.773219	2.058525	-0.303893
H	1.797223	2.558015	-1.707851
C	0.630769	2.276316	2.151002
H	-0.284549	2.003495	2.698073
H	1.427488	1.574575	2.444115
H	0.935652	3.284022	2.474568
C	-1.073917	3.295646	-0.244036
H	-0.850168	4.360364	-0.072850
H	-1.275757	3.170928	-1.319907
H	-1.989159	3.052870	0.317521
H	5.403838	-1.422367	-1.380236
H	-1.625802	0.267668	-2.109530
C	-4.858226	-1.543760	-0.128693
H	-5.379293	-0.983891	0.660013
H	-5.603301	-1.712021	-0.931760
H	-4.528354	-2.526468	0.228639
C	-3.964272	0.705337	-0.820681
H	-3.260720	1.236933	-1.467239
H	-5.001791	0.938225	-1.103236
H	-3.843777	1.088265	0.209503

1 imaginary frequency: –95.87 cm^{-1}

TS1a
Charge: 1

C	1.160096	-2.137560	1.206521
C	0.690383	-1.263577	0.214084
C	1.084072	-1.497054	-1.112587
C	1.874645	-2.593042	-1.445292
C	2.306634	-3.471219	-0.451691
C	1.954958	-3.232979	0.875802
H	0.929738	-1.945376	2.254917
H	0.784873	-0.804137	-1.899474
H	2.161364	-2.757625	-2.485805
H	2.930143	-4.329294	-0.709931
H	2.312754	-3.896936	1.665093
C	-0.127673	-0.071414	0.561801
C	-1.096678	0.001057	1.512275
C	-1.688433	1.316238	1.933513
H	-1.340862	2.159025	1.326325
H	-2.788685	1.287844	1.903849
H	-1.415349	1.511461	2.984336
C	-1.593989	-1.188558	2.284597
H	-2.679739	-1.095627	2.443360
H	-1.394900	-2.145078	1.784159
H	-1.140832	-1.230411	3.288832
C	-1.255898	1.336897	-1.751682
H	-0.974468	2.384558	-1.859294
H	-0.717271	0.628933	-2.380508
C	-2.459367	1.009547	-1.142466
H	-3.012504	1.809220	-0.645881
C	-2.919043	-0.288295	-0.987040
O	0.135347	1.038988	-0.192385
C	-2.316380	-1.488189	-1.629661
H	-1.515101	-1.270001	-2.341580
H	-1.916250	-2.158163	-0.851902
H	-3.108942	-2.050886	-2.146967
C	-4.158191	-0.535011	-0.199951
H	-4.979279	-0.752012	-0.905304
H	-4.048885	-1.431405	0.428570
H	-4.452393	0.323788	0.415595
Si	1.579676	2.029344	-0.033755
C	2.197609	1.723787	1.694314
H	2.492538	0.672270	1.830926
H	3.080968	2.352042	1.889395
H	1.429971	1.971722	2.442948
C	2.782445	1.503622	-1.352940
H	3.145033	0.479199	-1.183277
H	2.327778	1.552124	-2.354619
H	3.652024	2.180313	-1.347981
C	0.972800	3.772237	-0.294071
H	0.659523	3.961939	-1.332142

H	0.137563	4.018864	0.378923
H	1.795009	4.472002	-0.074072

1 imaginary frequency: –104.84 cm^{-1}

TS2a
Charge: 1

C	-0.526738	-2.395617	-1.130565
C	-0.425349	-1.349850	-0.201659
C	-1.038241	-1.507378	1.053537
C	-1.692650	-2.687220	1.386915
C	-1.770822	-3.726797	0.458983
C	-1.196436	-3.572328	-0.801118
H	-0.124353	-2.284031	-2.135875
H	-1.003565	-0.691241	1.776649
H	-2.151204	-2.794179	2.371695
H	-2.291554	-4.651635	0.714462
H	-1.277993	-4.370159	-1.541534
C	0.210487	-0.048273	-0.526540
C	1.347451	0.150535	-1.299180
C	1.650341	1.540145	-1.791785
H	1.226433	1.657905	-2.803795
H	1.218848	2.318158	-1.153091
H	2.732587	1.708721	-1.889988
C	2.048189	-0.953491	-2.040881
H	3.079166	-0.656630	-2.276623
H	2.072807	-1.905953	-1.494511
H	1.556296	-1.135583	-3.010650
C	1.190198	1.628496	2.107672
H	0.891157	2.646250	2.367360
H	0.632042	0.818315	2.578745
C	2.242070	1.411697	1.288123
H	2.764372	2.273702	0.864809
C	2.693698	0.121094	0.864279
O	-0.357810	1.009264	0.029089
C	2.281712	-1.138576	1.544138
H	1.336277	-1.075928	2.088989
H	2.229426	-1.976305	0.834355
H	3.077954	-1.387540	2.267072
C	4.006884	0.058633	0.160150
H	4.777484	0.180737	0.943027
H	4.187282	-0.911169	-0.318318
H	4.152501	0.876932	-0.556515
Si	-1.931829	1.736361	-0.123828
C	-2.605904	1.871289	1.606805
H	-3.549989	2.438866	1.589018
H	-2.819056	0.883962	2.042748
H	-1.910396	2.407440	2.270439

C	-1.593830	3.407827	-0.871942
H	-0.888471	3.982393	-0.251711
H	-1.170429	3.314626	-1.883525
H	-2.526877	3.988292	-0.947081
C	-2.952441	0.639948	-1.230999
H	-2.453055	0.474383	-2.198481
H	-3.150341	-0.339230	-0.770180
H	-3.920974	1.124943	-1.432208

1 imaginary frequency: –130.42 cm^{-1}

8.4 3-D Figures of Calculated Structures

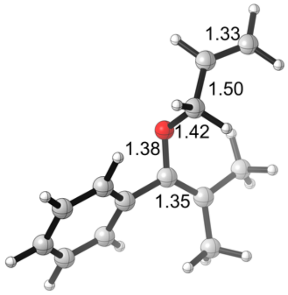

INT1

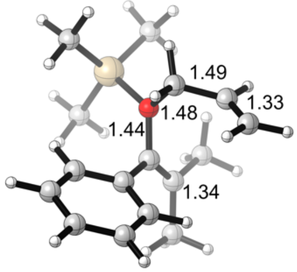

INT1'

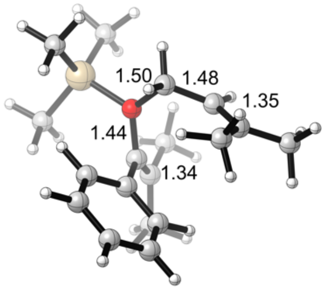

INT1a

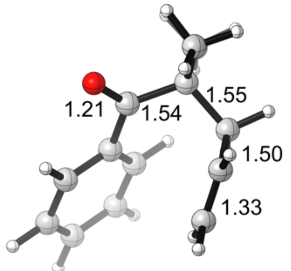

INT2

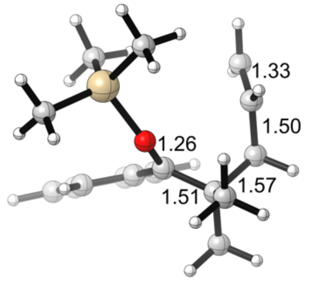

INT2’

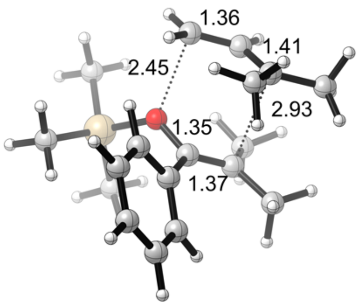

INT2a

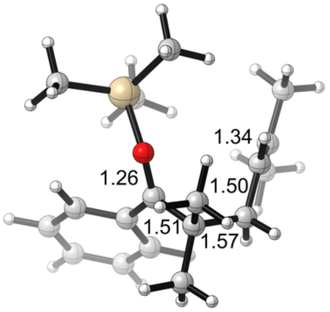

INT2a’

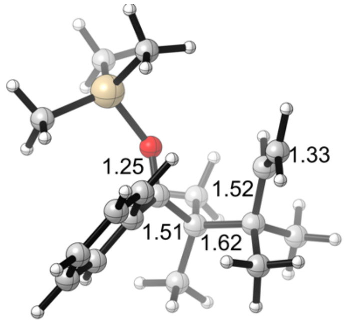

INT3a

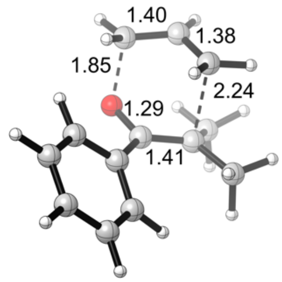

TS1

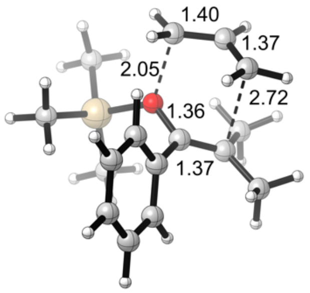

TS1’

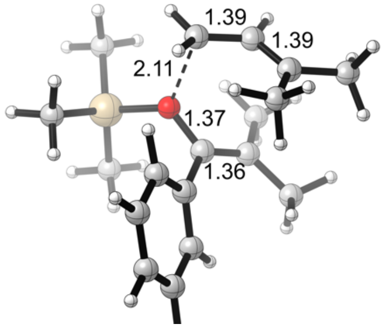

TS1a

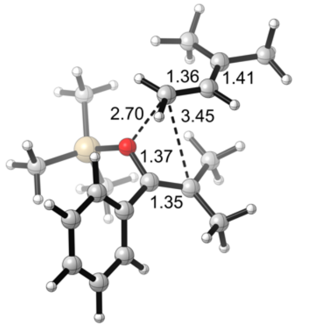

TS1a’

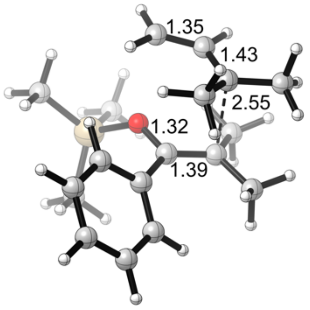

TS2a

8.5 CM5 Charge Calculations of neutral TS1 and cationic TS1'

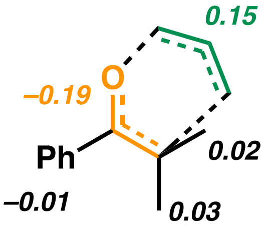

TS1

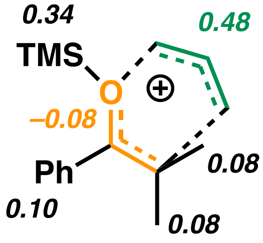

TS1'

9- References

(1) Williams, C. G.; Nistanaki, S. K.; Wells, C. W.; Nelson, H. M. α-Vinylation of Ester Equivalents via Main Group Catalysis for the Construction of Quaternary Centers. *Org. Lett.* **2023**, *25*, 3591–3595.

(2) Zell, D.; Kingston, C.; Jermaks, J.; Smith, S. R.; Seeger, N.; Wassmer, J.; Sirois, L. E.; Han, C.; Zhang, H.; Sigman, M. S.; Gosselin, F. Stereoconvergent and -Divergent Synthesis of Tetrasubstituted Alkenes by Nickel-Catalyzed Cross-Couplings. *J. Am. Chem. Soc.* **2021**, *143*, 19078–19090.

(3) Li, B. X.; Le, D. N.; Mack, K. A.; McClory, A.; Lim, N.-K.; Cravillion, T.; Savage, S.; Han, C.; Collum, D. B.; Zhang, H.; Gosselin, F. Highly Stereoselective Synthesis of Tetrasubstituted Acyclic All-Carbon Olefins via Enol Tosylation and Suzuki–Miyaura Coupling. *J. Am. Chem. Soc.* **2017**, *139*, 10777–10783.

(4) Malmedy, F.; Wirth, T. Stereoselective Ketone Rearrangements with Hypervalent Iodine Reagents. *Chem. - Eur. J.* **2016**, *22*, 16072–16077.

(5) Liu, W.; Pu, M.; He, J.; Zhang, T.; Dong, S.; Liu, X.; Wu, Y.-D.; Feng, X. Iron-Catalyzed Enantioselective Radical Carboazidation and Diazidation of α,β-Unsaturated Carbonyl Compounds. *J. Am. Chem. Soc.* **2021**, *143*, 11856–11863.

(6) Su, F.; Zou, J.; Lv, X.; Lu, F.; Long, Y.; Tang, K.; Li, B.; Chai, H.; Wu, X.; Chi, Y. R. Carbene-Catalyzed Intermolecular Dehydrogenative Coupling of Aldehydes with C(Sp ^{3})−H Bonds. *Angew. Chem. Int. Ed.* **2023**, *62*, e202303388.

(7) Lou, S.; Fu, G. C. Nickel/Bis(Oxazoline)-Catalyzed Asymmetric Kumada Reactions of Alkyl Electrophiles: Cross-Couplings of Racemic α-Bromoketones. *J. Am. Chem. Soc.* **2010**, *132*, 1264–1266.

(8) Goeppel, D.; Münster, I.; Brückner, R. The Geometry of the Carbanionic Moiety Influences the Non-Induced Diastereoselectivity of the [2,3]-Wittig Rearrangement of Lithiated Diallyl Ethers. *Tetrahedron* **1994**, *50*, 3687–3708.

(9) Hayashi, T.; Konishi, M.; Yokota, K.-I.; Kumada, M. Regio- and Stereo-Chemistry in Allylation of Aryl Grignard Reagents Catalyzed by Phosphine-Nickel and -Palladium Complexes. *J. Organomet. Chem.* **1985**, *285* (1–3), 359–373.

(10) Onishi, Y.; Nishimoto, Y.; Yasuda, M.; Baba, A. $InCl_3$ /Me_3 SiBr-Catalyzed Direct Coupling between Silyl Ethers and Enol Acetates. *Org. Lett.* **2011**, *13*, 2762–2765.

(11) Steele, K. P.; Weber, W. P. Mass Spectrometry of Allyloxy Di- and Trimethylsilanes. *Org. Mass Spectrom.* **1982**, *17*, 222–228.

(12) Mello, R.; Martínez-Ferrer, J.; Alcalde-Aragonés, A.; Varea, T.; Acerete, R.; González-Núñez, M. E.; Asensio, G. Reactions at Interfaces: Oxygenation of *n* -Butyl Ligands Anchored on Silica Surfaces with Methyl(Trifluoromethyl)Dioxirane. *J. Org. Chem.* **2011**, *76*, 10129–10139.

(13) Nuñez, S. A.; Yeung, K.; Fox, N. S.; Phillips, S. T. A Structurally Simple Self-Immolative Reagent That Provides Three Distinct, Simultaneous Responses per Detection Event. *J. Org. Chem.* **2011**, *76*, 10099–10113.

(14) Kleinmans, R.; Apolinar, O.; Derosa, J.; Karunananda, M. K.; Li, Z.-Q.; Tran, V. T.; Wisniewski, S. R.; Engle, K. M. Ni-Catalyzed 1,2-Diarylation of Alkenyl Ketones: A Comparative Study of Carbonyl-Directed Reaction Systems. *Org. Lett.* **2021**, *23*, 5311–5316.

(15) Faulkner, A.; Scott, J. S.; Bower, J. F. An Umpolung Approach to Alkene Carboamination: Palladium Catalyzed 1,2-Amino-Acylation, -Carboxylation, -Arylation, -Vinylation, and -Alkynylation. *J. Am. Chem. Soc.* **2015**, *137*, 7224–7230.

(16) Alexy, E. J.; Zhang, H.; Stoltz, B. M.; Catalytic Enantioselective Synthesis of Acyclic Quaternary Centers: Palladium-Catalyzed Decarboxylative allylic Alkylation of Fully Substituted Acyclic Enol Carbonates. *J. Am. Chem. Soc.* 2018, **140**, *32*, 10109–10112.
(17) Faulkner, A.; Bower, J. F. Highly Efficient Narasaka-Heck Cyclizations Mediated by P(3,5-(CF3)2C6H3)3: Facile Access to N-Heterobicyclic Scaffolds. *Angew. Chem. Int. Ed.* **2012**, *51*, 1675–1679.
(18) Banerjee, A.; Hattori, T.; Yamamoto, H. Regio- and Stereoselective (SN2) N-, O-, C- and S-Alkylation Using Trialkyl Phosphates. *Synthesis* **2023**, *55*, 315–332.
(19) Uyeda, C.; Jacobsen, E. N. Enantioselective Claisen Rearrangements with a Hydrogen-Bond Donor Catalyst. *J. Am. Chem. Soc.* **2008**, *130*, 9228–9229.
(20) Schuetz, R. D.; Millard, F. W. Reaction of N-Bromobenzamide with Allyl-1-d $_2$ Acetate [1]. *J. Org. Chem.* **1959**, *24*, 297–300.
(20) Frisch, M. J.; Trucks, G. W.; Schlegel, H. B.; Scuseria, G. E.; Robb, M. A.; Cheeseman, J. R.; Scalmani, G.; Barone, V.; Petersson, G. A.; Nakatsuji, H.; Li, X.; Caricato, M.; Marenich, A. V.; Bloino, J.; Janesko, B. G.; Gomperts, R.; Mennucci, B.; Hratchian, H. P.; Ortiz, J. V.; Izmaylov, A. F.; Sonnenberg, J. L.; Williams-Young, D.; Ding, F.; Lipparini, F.; Egidi, F.; Goings, J.; Peng, B.; Petrone, A.; Henderson, T.; Ranasinghe, D.; Zakrzewski, V. G.; Gao, J.; Rega, N.; Zheng, G.; Liang, W.; Hada, M.; Ehara, M.; Toyota, K.; Fukuda, R.; Hasegawa, J.; Ishida, M.; Nakajima, T.; Honda, Y.; Kitao, O.; Nakai, H.; Vreven, T.; Throssell, K.; Montgomery, J. A., Jr.; Peralta, J. E.; Ogliaro, F.; Bearpark, M. J.; Heyd, J. J.; Brothers, E. N.; Kudin, K. N.; Staroverov, V. N.; Keith, T. A.; Kobfayashi, R.; Normand, J.; Raghavachari, K.; Rendell, A. P.; Burant, J. C.; Iyengar, S. S.; Tomasi, J.; Cossi, M.; Millam, J. M.; Klene, M.; Adamo, C.; Cammi, R.; Ochterski, J. W.; Martin, R. L.; Morokuma, K.; Farkas, O.; Foresman, J. B.; and Fox, D. J. *Gaussian 16*; Gaussian, Inc.: Wallingford, CT, **2016**.
(21) Chai, J.-D.; Head-Gordon, M. Long-range corrected hybrid density functionals with damped atom–atom dispersion corrections†. *Phys. Chem. Chem. Phys.* **2008**, *10*, 6615−6620.
(22) Weigend, F.; Ahlrichs, R. Balanced Basis Sets of Split Valence, Triple Zeta Valence and Quadruple Zeta Valence Quality for H to Rn: Design and Assessment of Accuracy. *Phys. Chem. Chem. Phys.* **2005**, *7*, 3297−3305.
(23) (a) Barone, V.; Cossi, M. Quantum Calculation of Molecular Energies and Energy Gradients in Solution by a Conductor Solvent Model. *J. Phys. Chem. A* **1998**, *102*, 1995–2001. (b) Cossi, M.; Rega, N.; Scalmani, G.; Barone, V. Energies, Structures, and Electronic Properties of Molecules in Solution with the C-PCM Solvation Model. *J. Comput. Chem.* **2003**, *24*, 669–681.
(24) (a) Grimme, S.; Bannwarth, C.; Shushkov, P. A Robust and Accurate Tight-Binding Quantum Chemical Method for Structures, Vibrational Frequencies, and Noncovalent Interactions of Large Molecular Systems Parametrized for All spd-Block Elements (z = 1-86). *J. Chem. Theory Comput.* **2017**, *13*, 1989–2009. (b) Bannwarth, C.; Ehlert, S.; Grimme, S. GFN2-xTB–An Accurate and Broadly Parametrized Self-Consistent Tight-Binding Quantum Chemical Method with Multipole Electrostatics and Density-Dependent Dispersion Contributions. *J. Chem. Theory Comput.* **2019**, *15*, 1652–1671. (c) Pracht, P.; Caldeweyher, E.; Ehlert, S.; Grimme, S. A Robust Non-Self-Consistent Tight-Binding Quantum Chemistry Method for Large Molecules. *ChemRxiv* **2019,** https://doi.org/10.26434/chemrxiv.8326202.v1
(25) Legault, C. Y. *CYLview*, 1.0b; Universite′ de Sherbrooke, **2009**; http://www.cylview.org.

10-Spectra

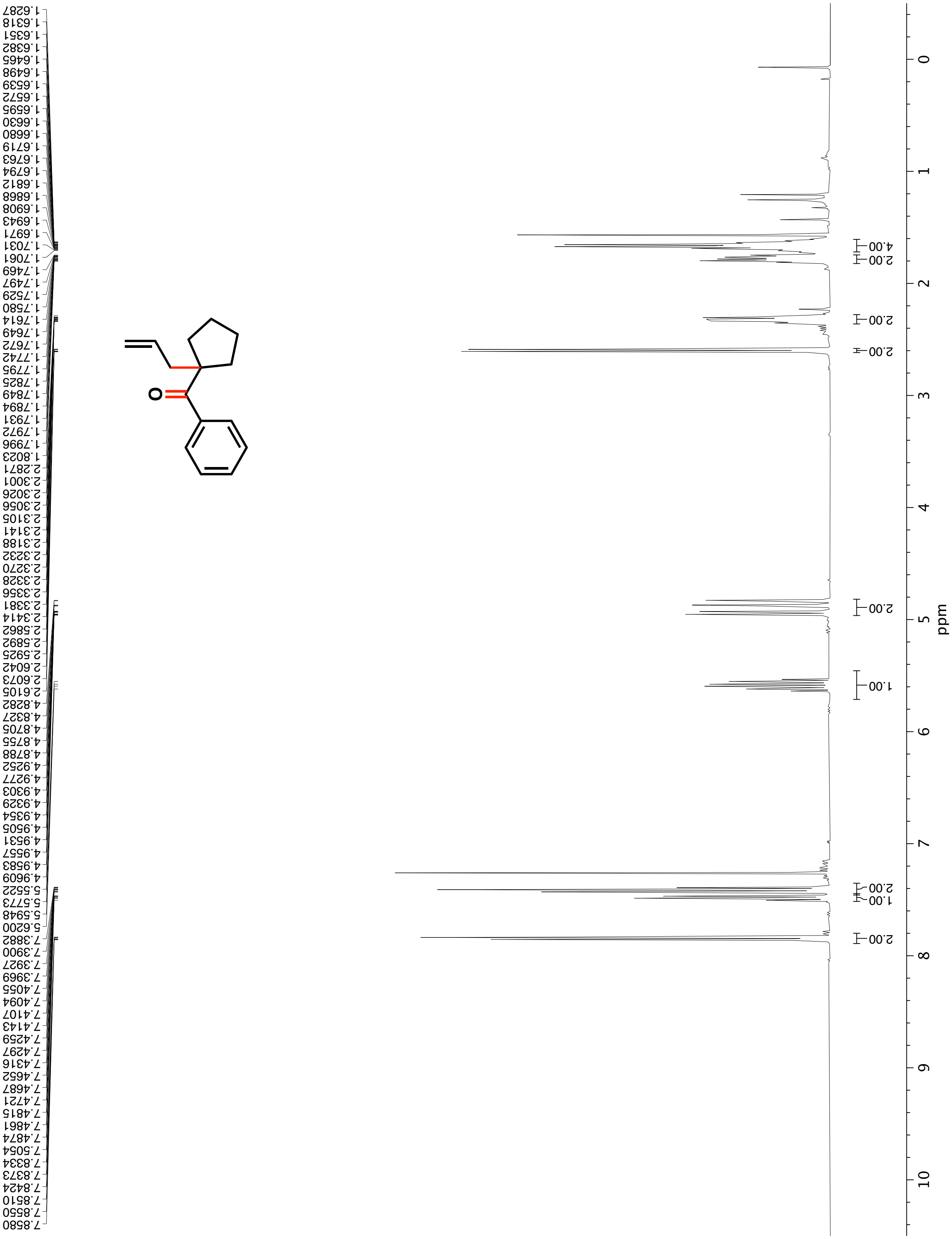

^{1}H NMR (400 MHz, $CDCl_3$) of compound **2c**.

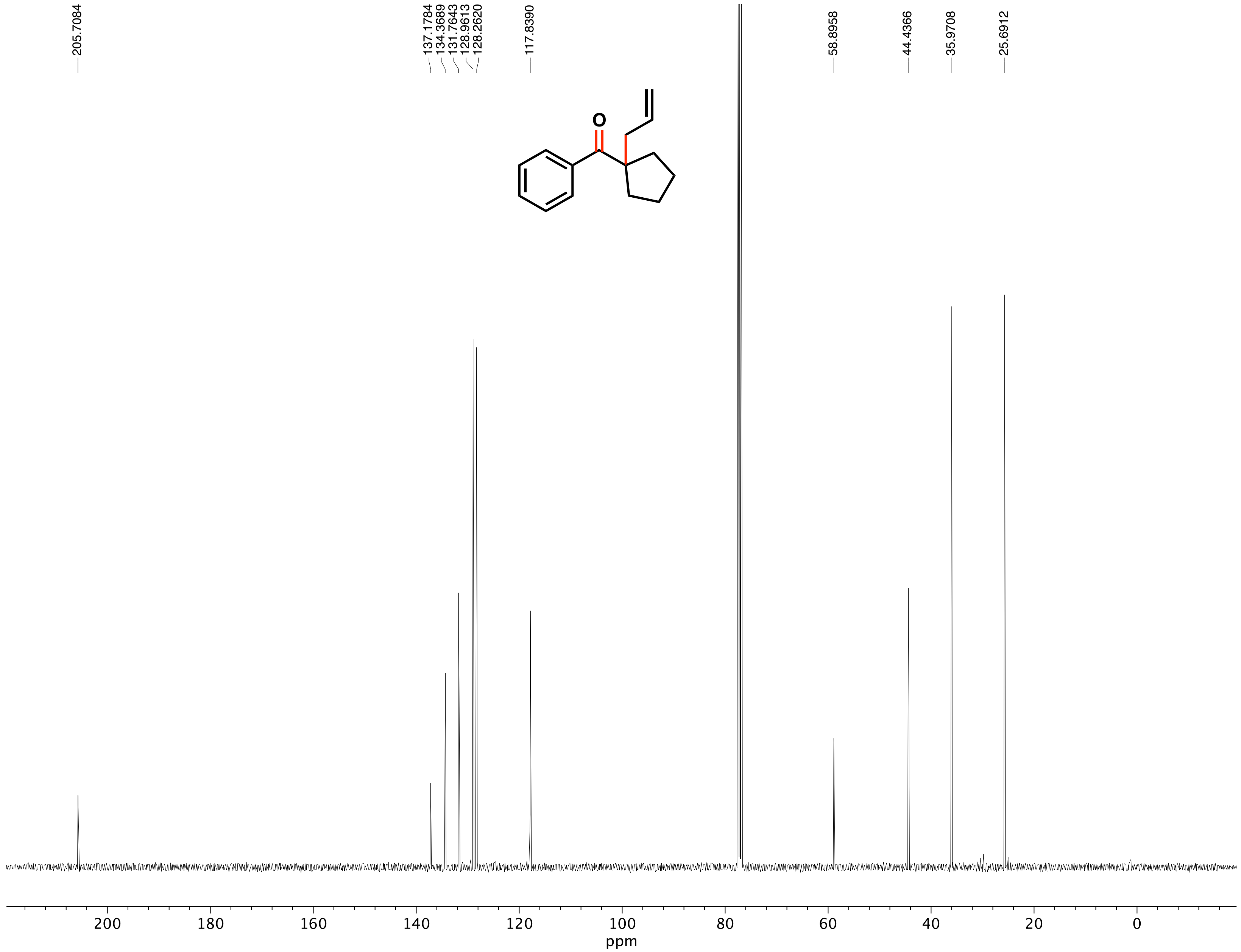

^{13}C NMR (101 MHz, $CDCl_3$) of compound **2c**.

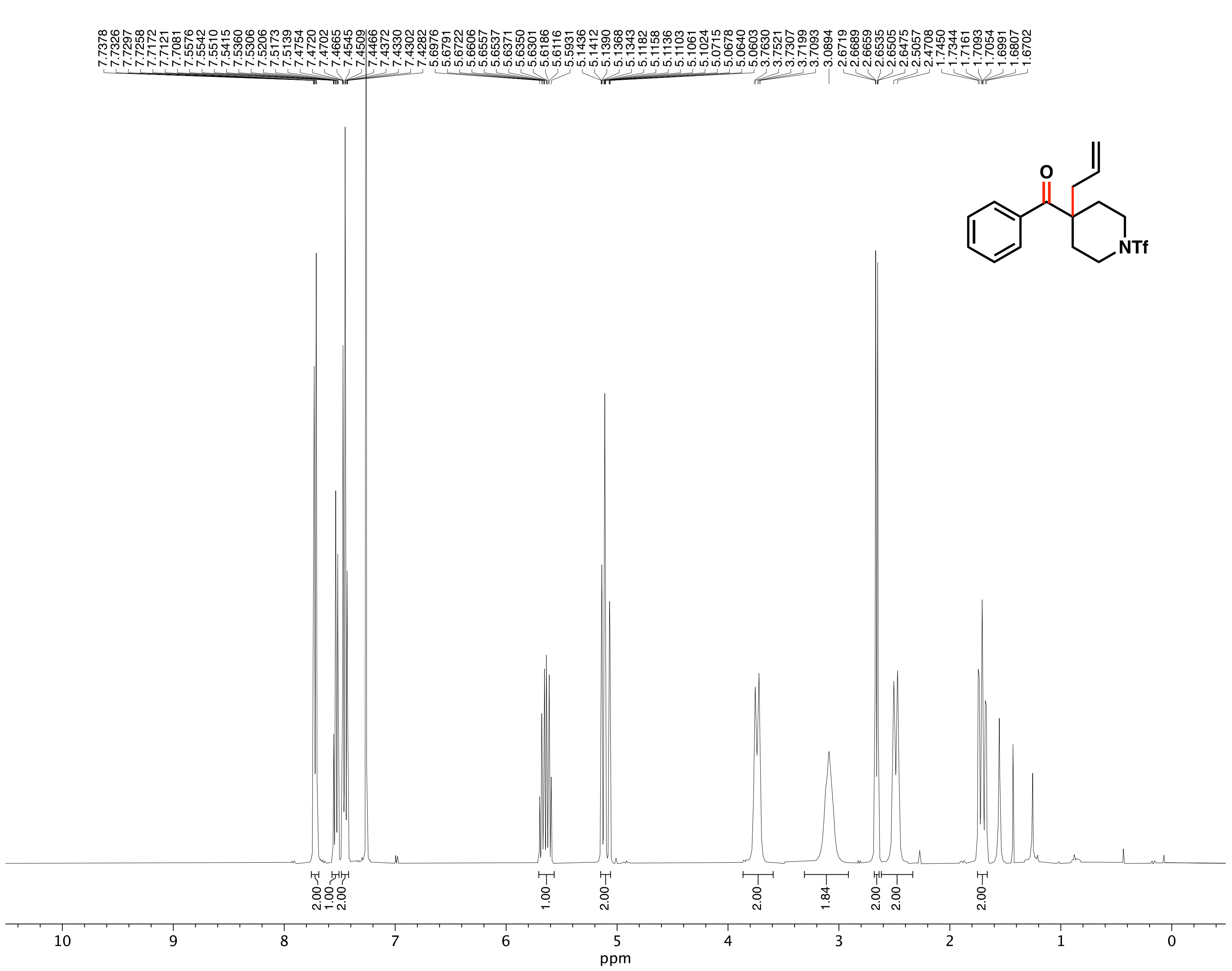

^{1}H NMR (400 MHz, $CDCl_3$) of compound **2d**.

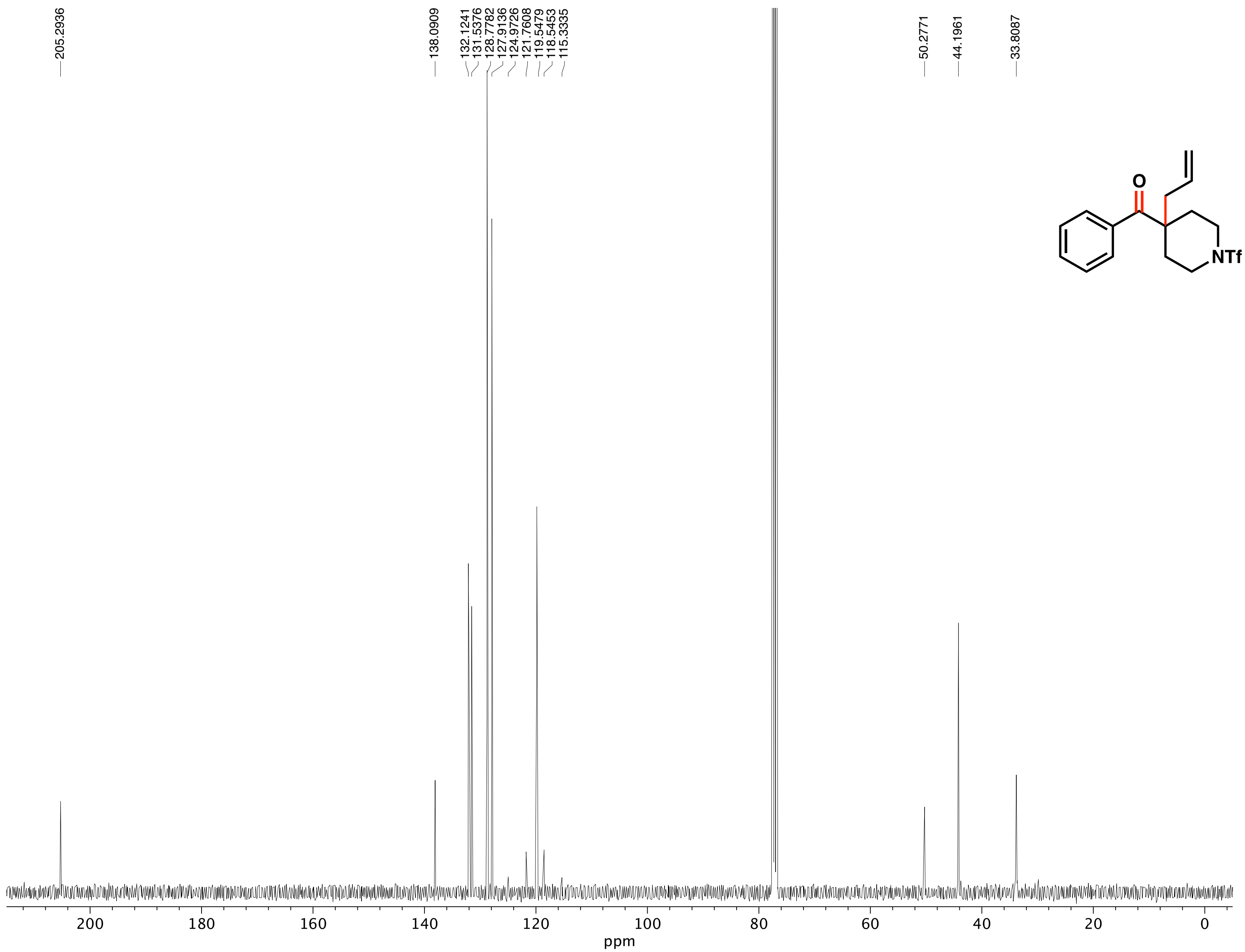

^{13}C NMR (101 MHz, $CDCl_3$) of compound **2d**.

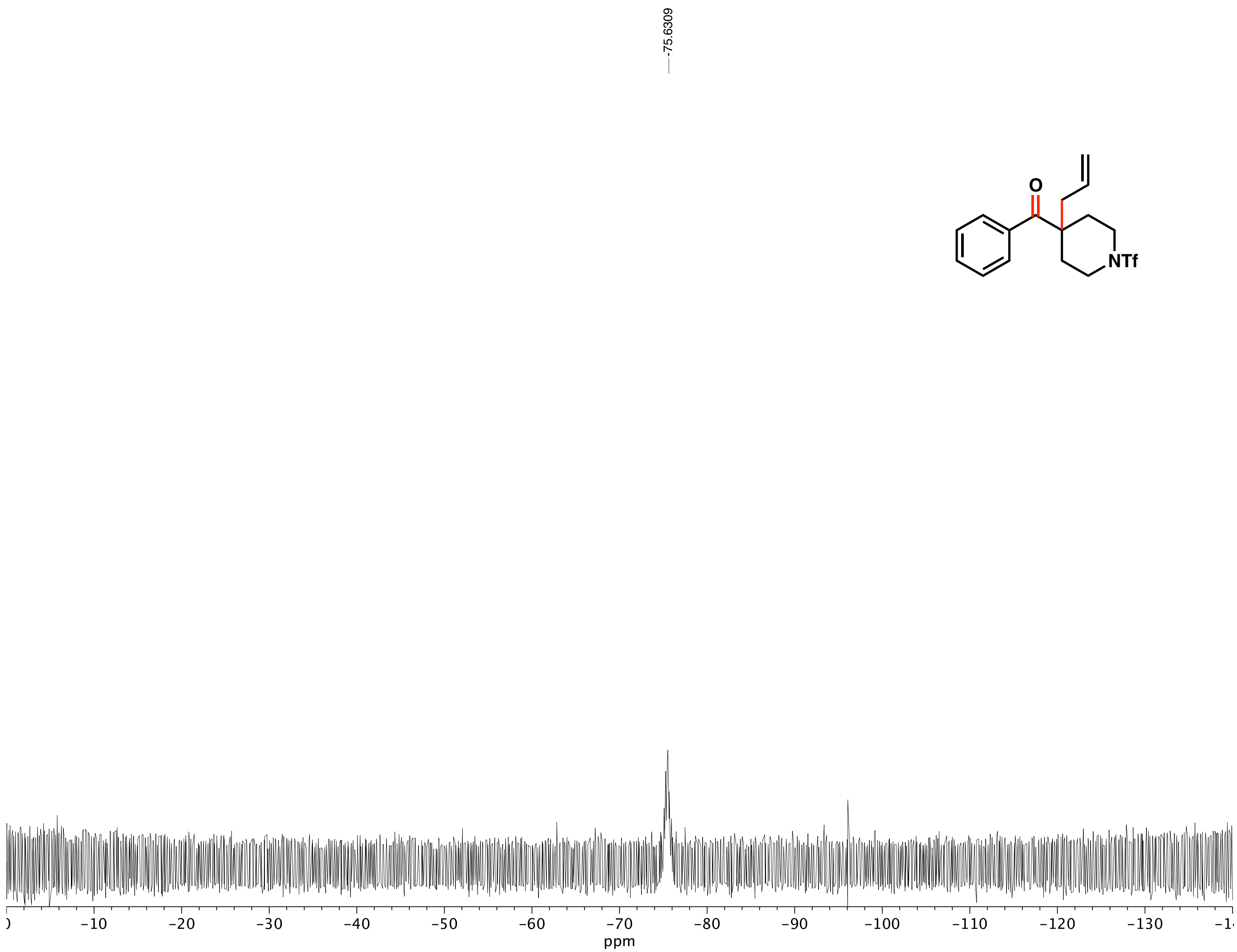

^{19}F NMR (282 MHz, $CDCl_3$) of compound **2d**.

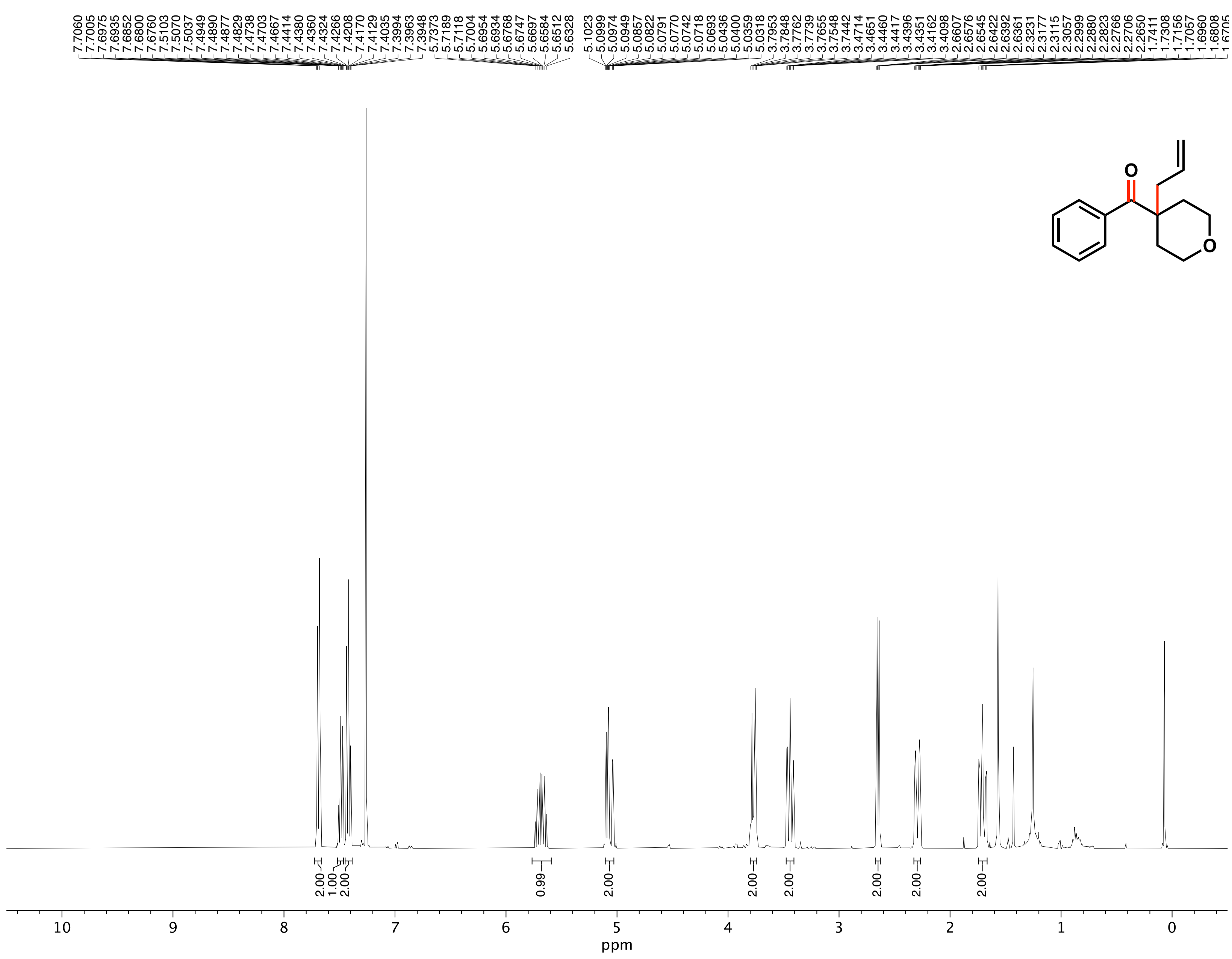

^{1}H NMR (400 MHz, $CDCl_3$) of compound **2e**.

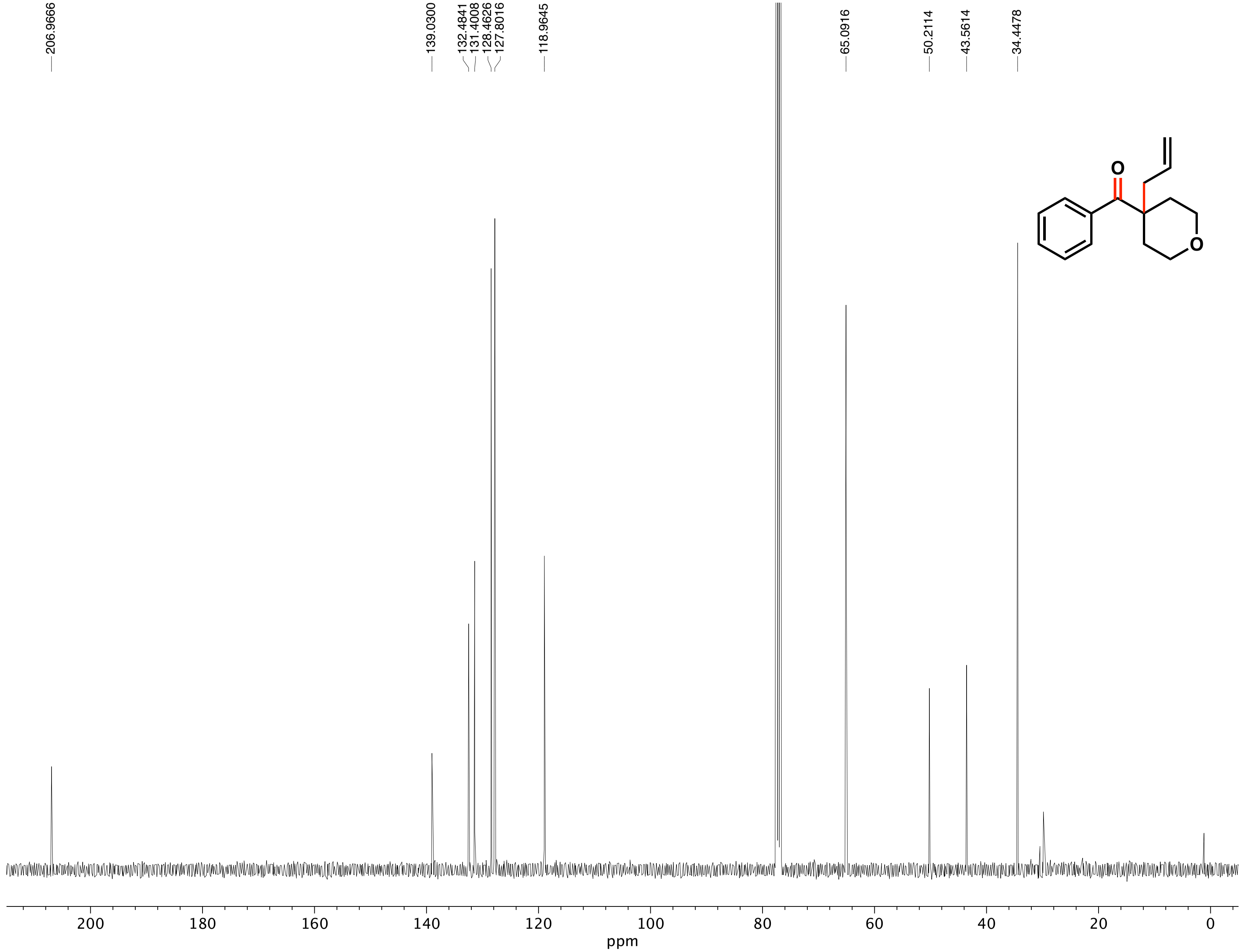

^{13}C NMR (101 MHz, $CDCl_3$) of compound **2e**.

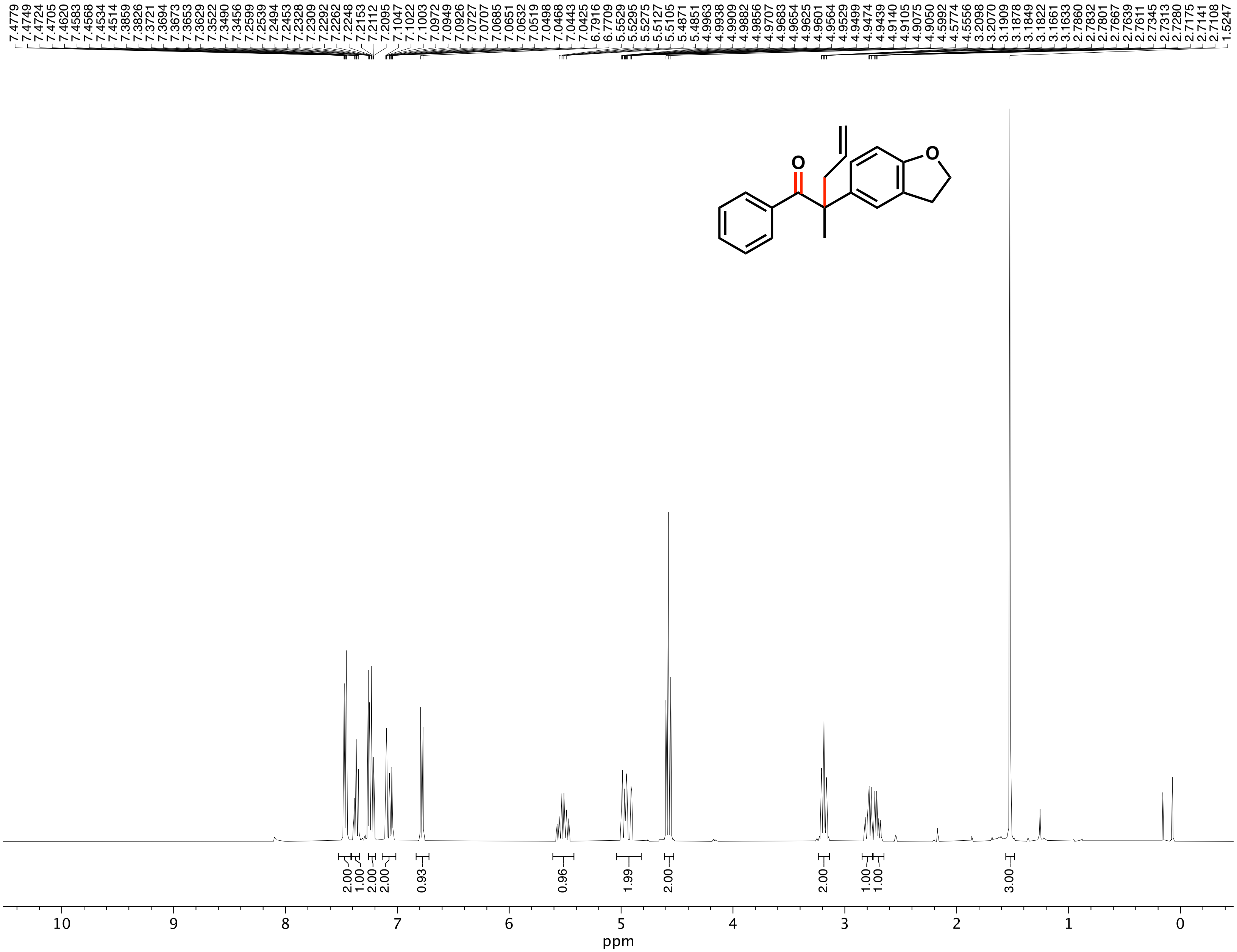

^{1}H NMR (400 MHz, $CDCl_3$) of compound **2f**.

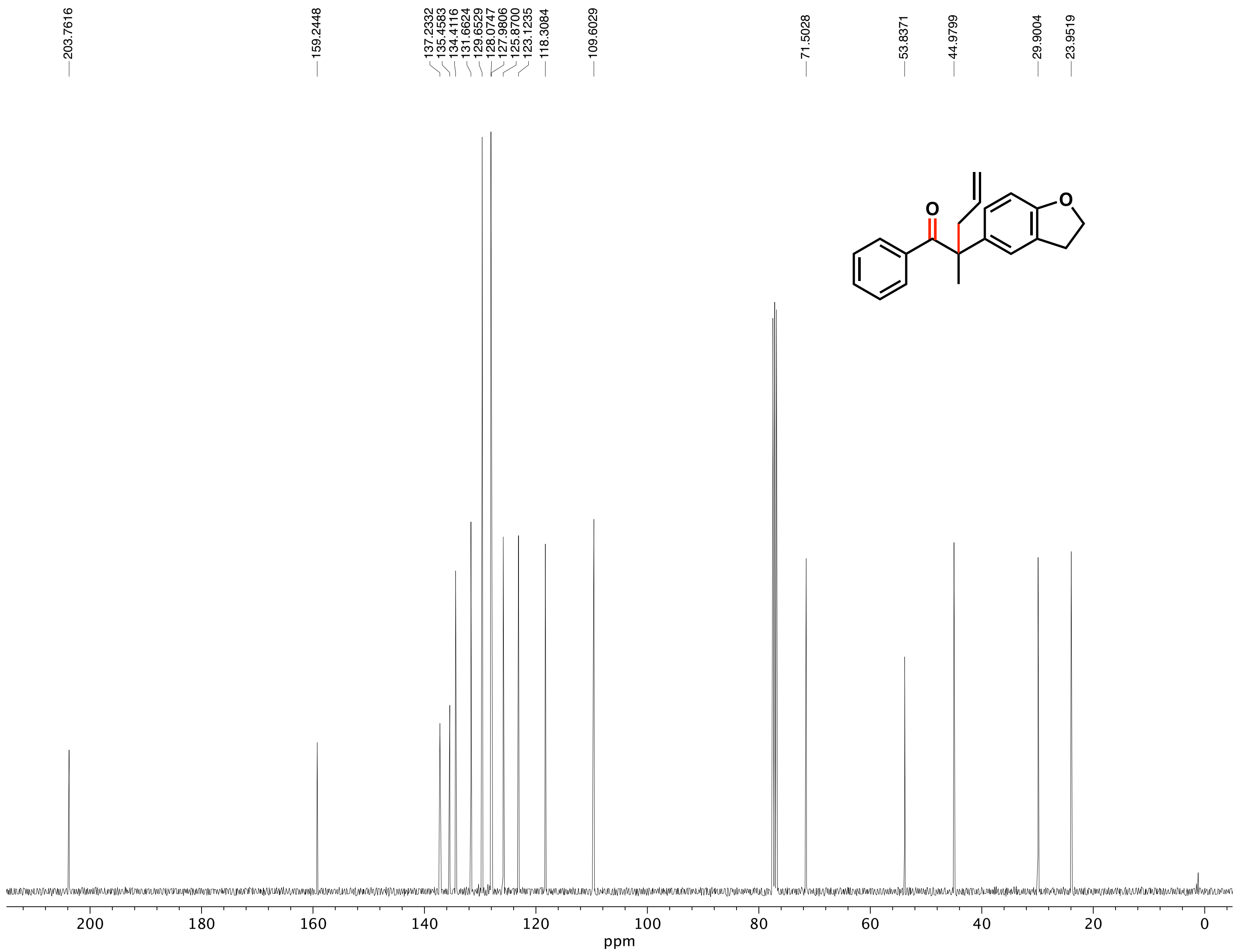

^{13}C NMR (101 MHz, $CDCl_3$) of compound **2f**.

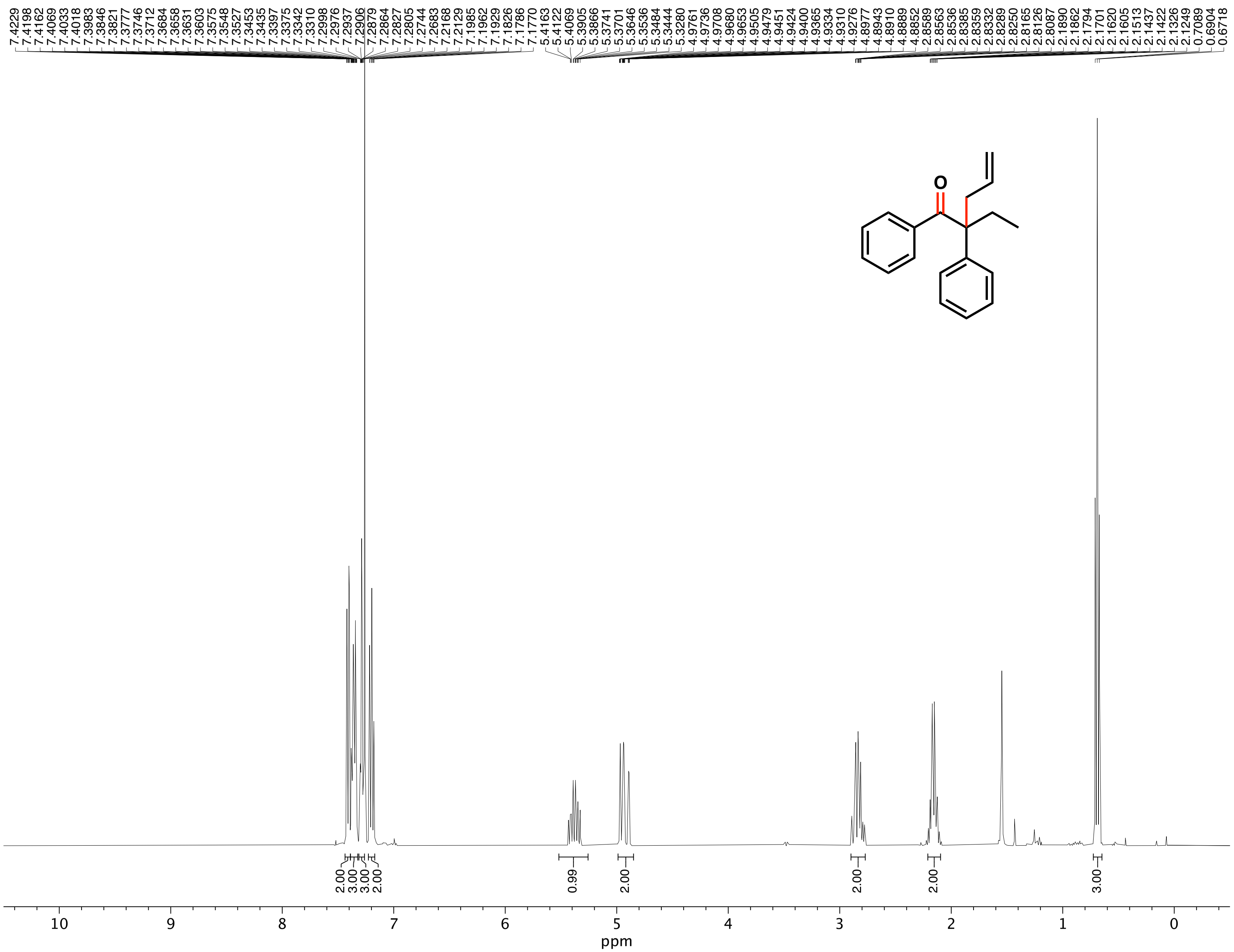

^{1}H NMR (400 MHz, $CDCl_3$) of compound **2g**.

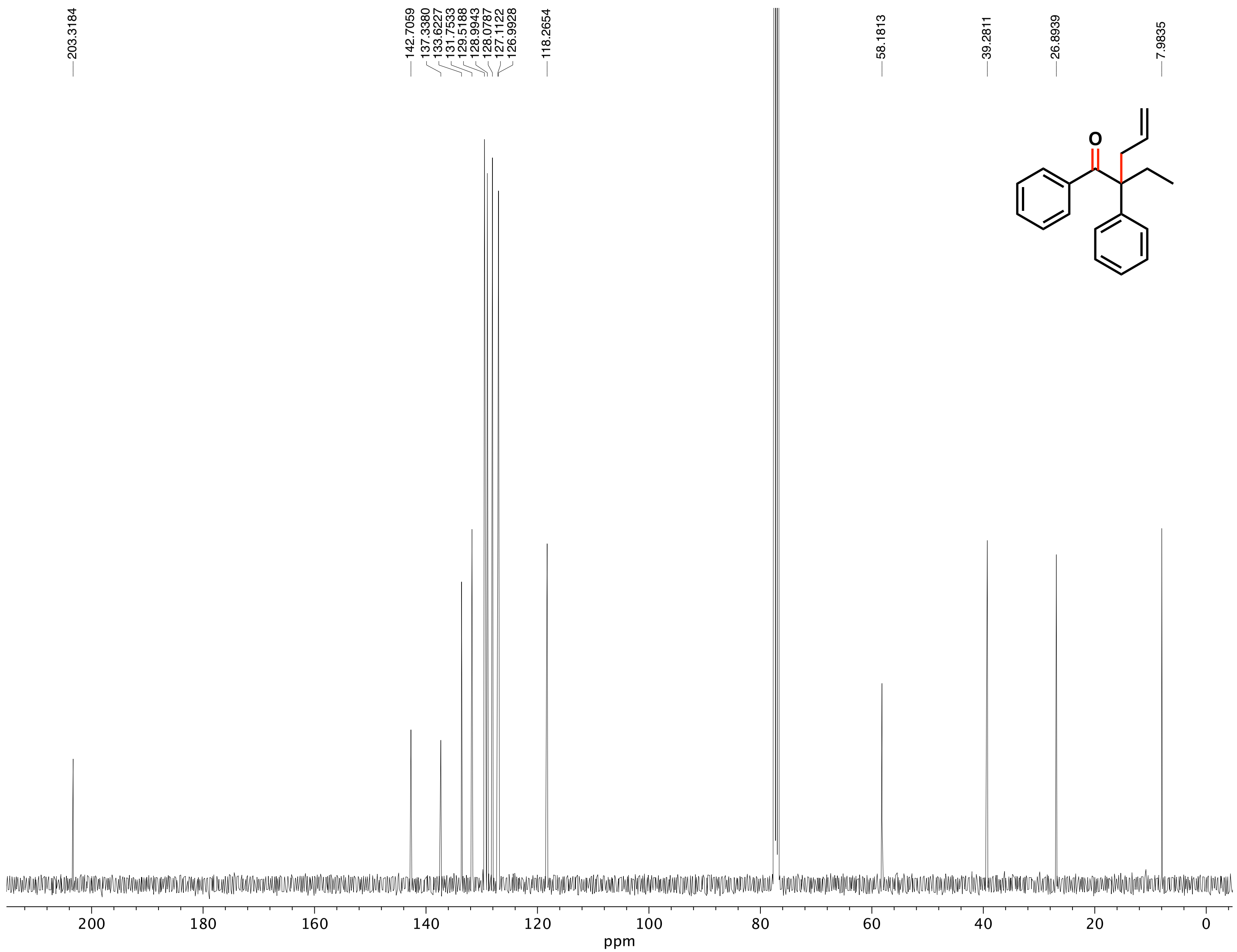

^{13}C NMR (101 MHz, $CDCl_3$) of compound **2g**.

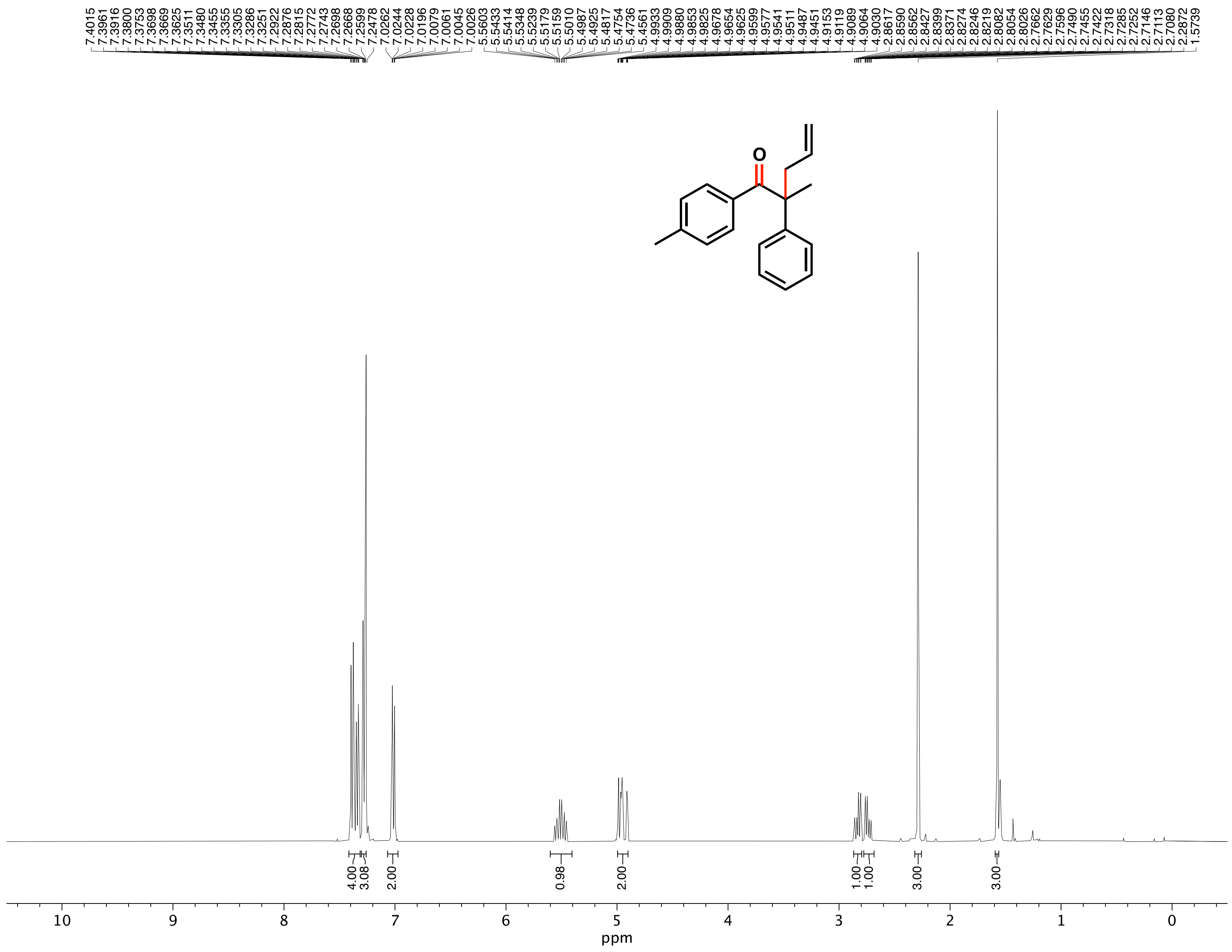

^{1}H NMR (400 MHz, $CDCl_3$) of compound **2h**.

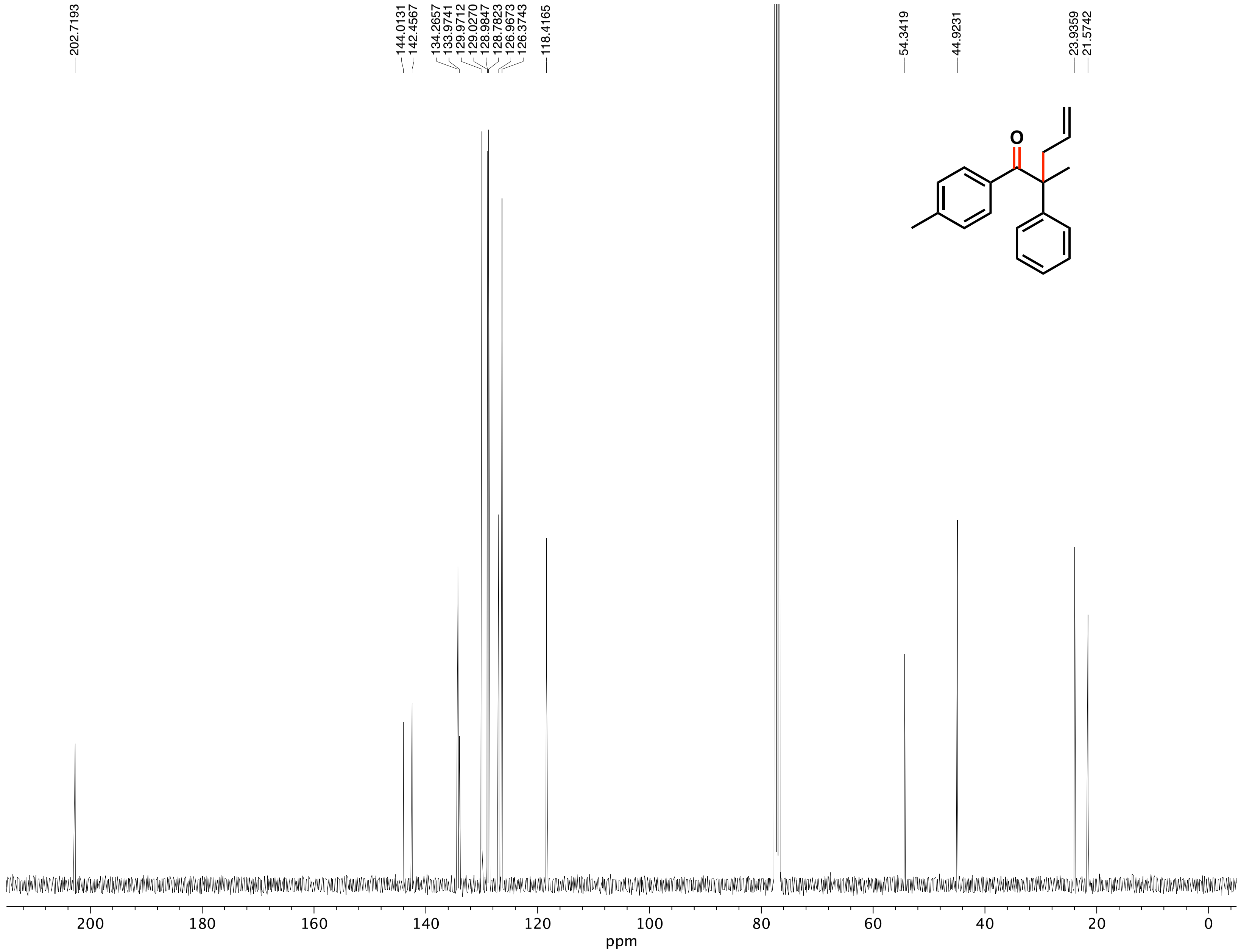

^{13}C NMR (101 MHz, $CDCl_3$) of compound **2h**.

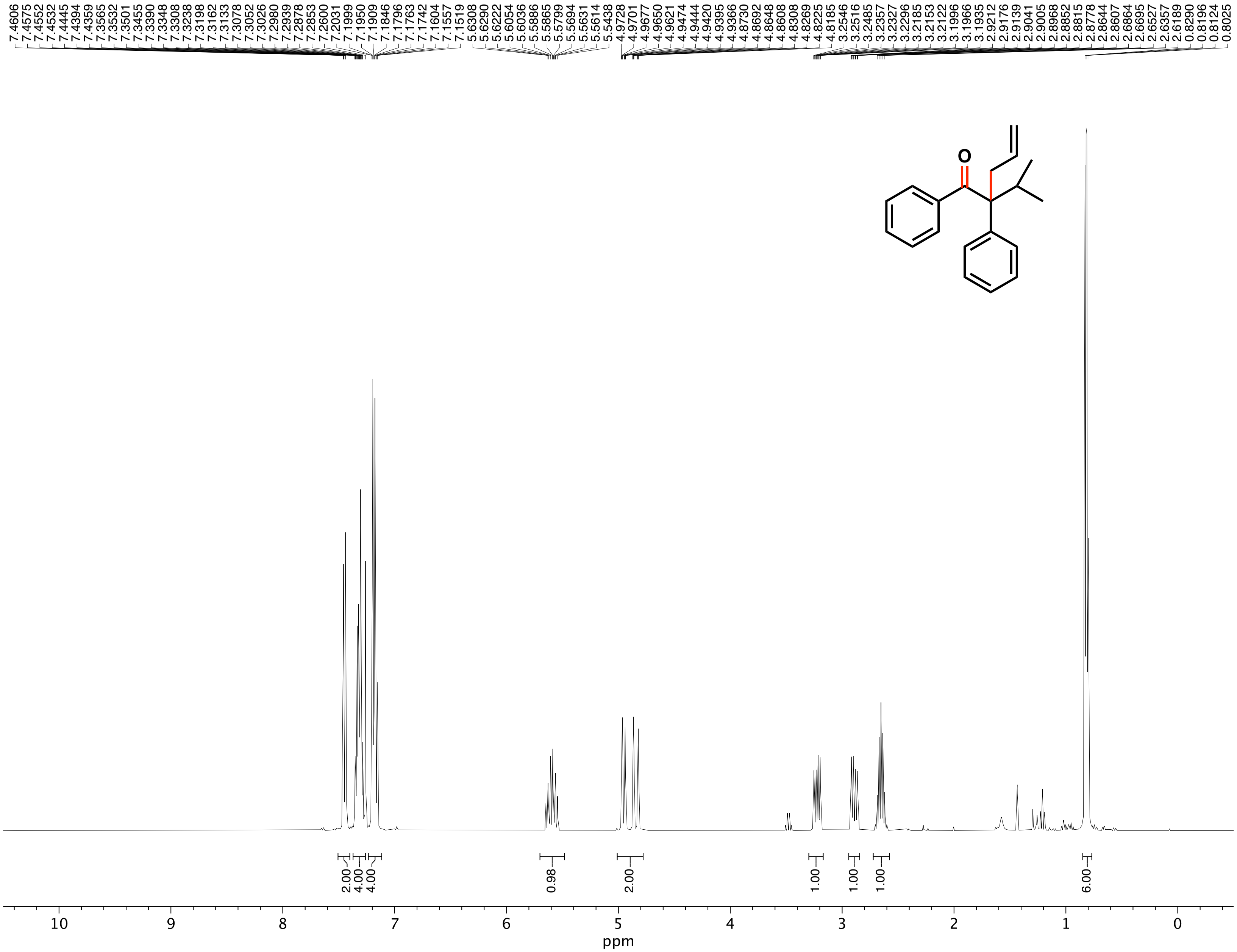

^{1}H NMR (400 MHz, $CDCl_3$) of compound **2i**.

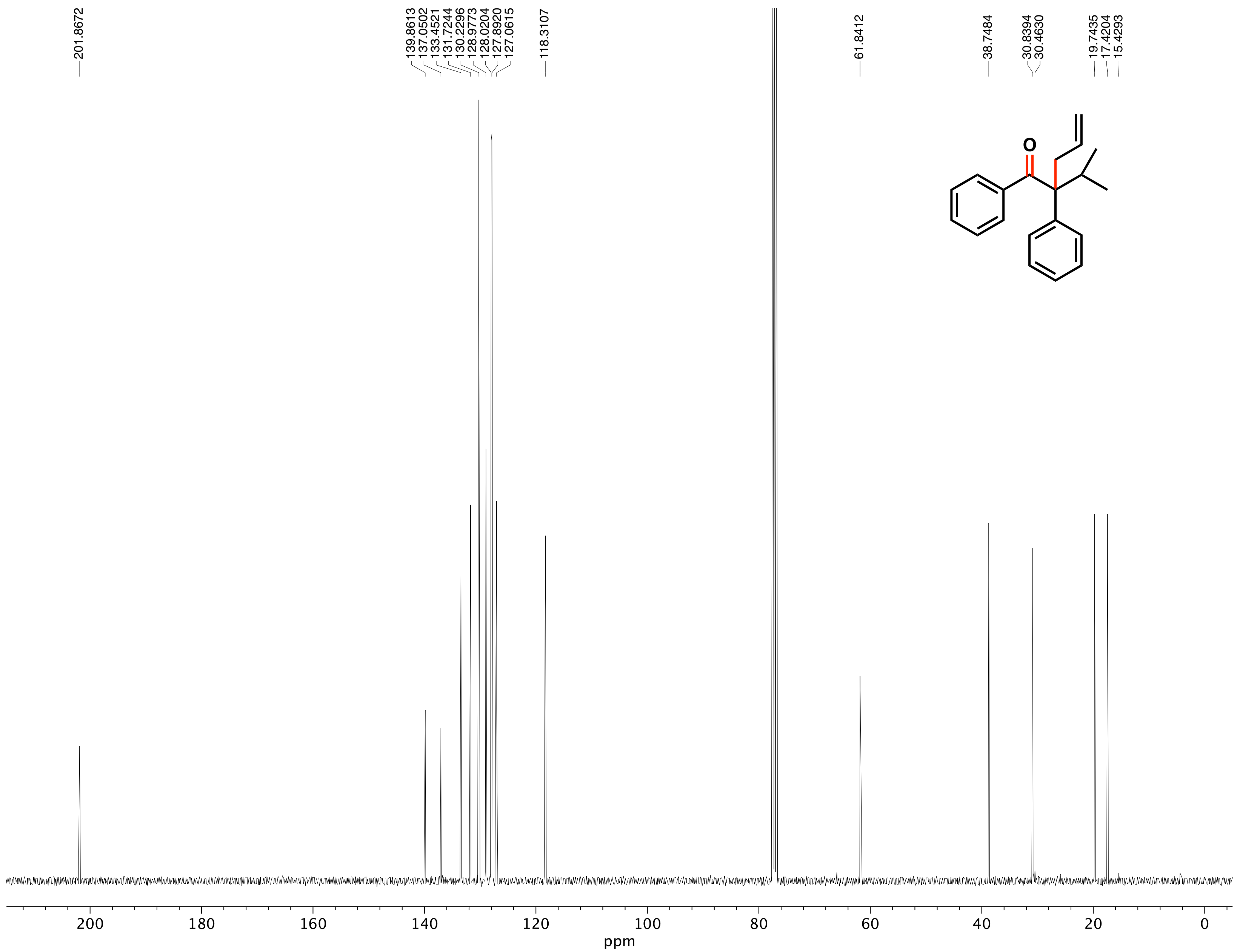

^{13}C NMR (101 MHz, $CDCl_3$) of compound **2i**.

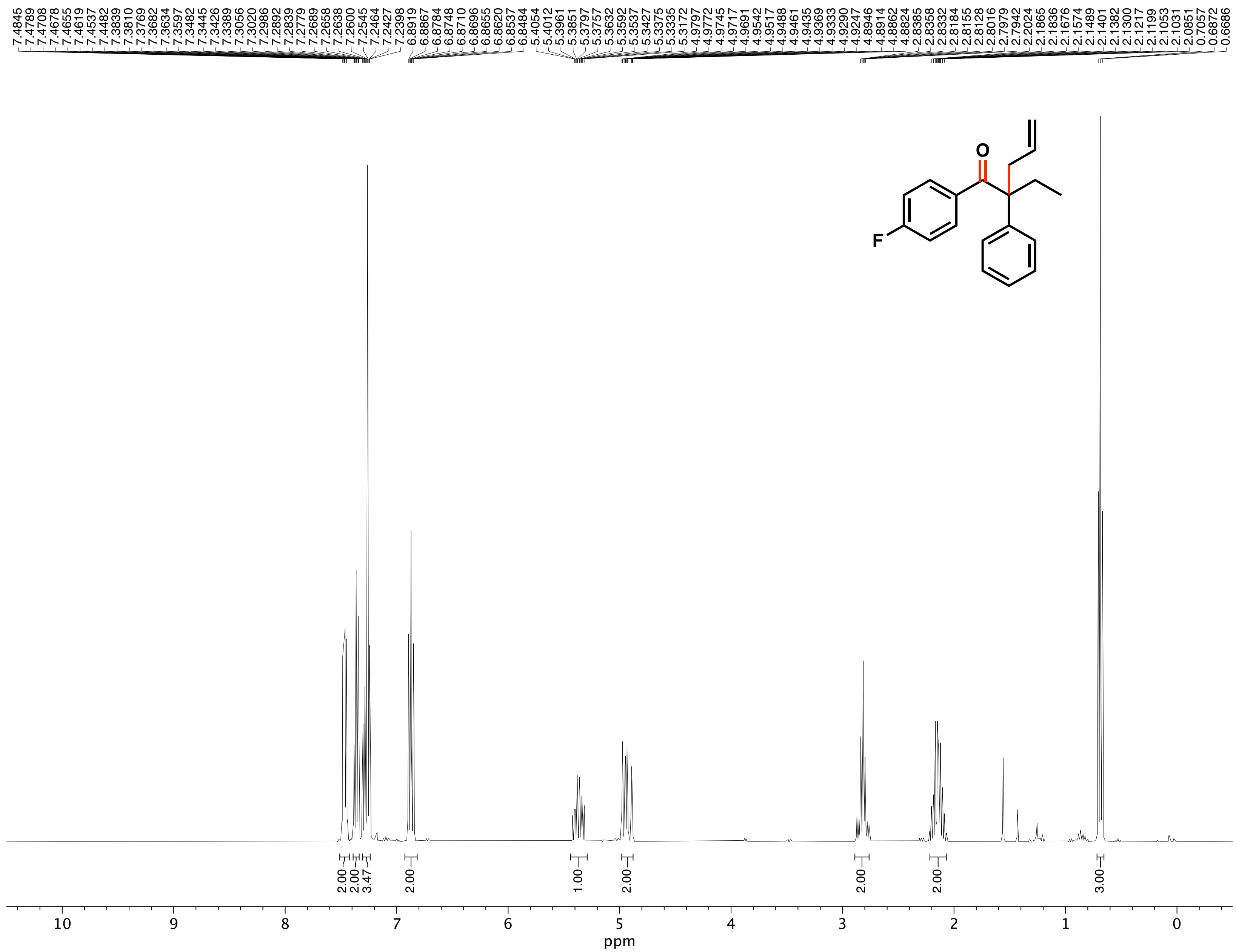

^{1}H NMR (400 MHz, $CDCl_3$) of compound **2j**.

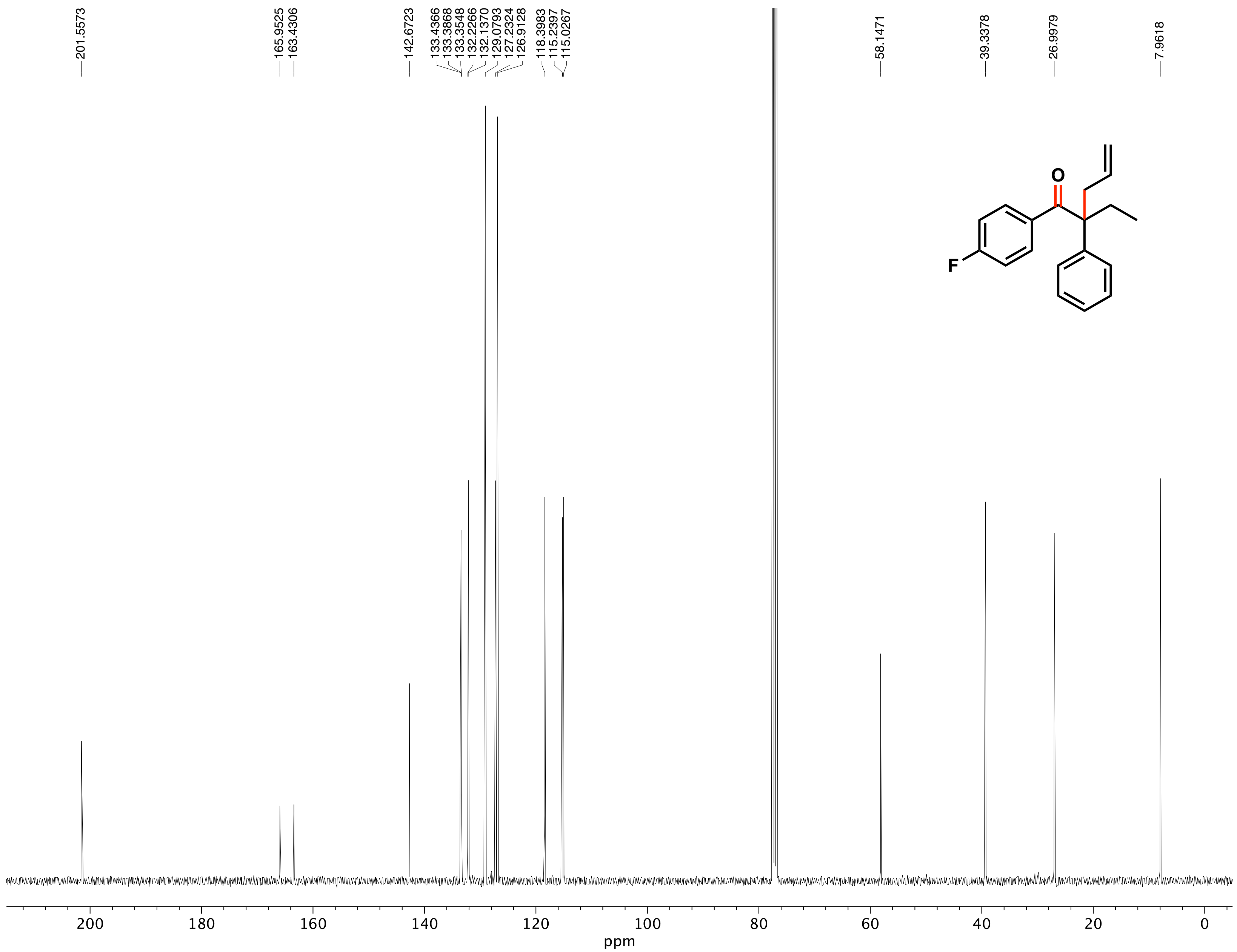

^{13}C NMR (101 MHz, $CDCl_3$) of compound **2j**.

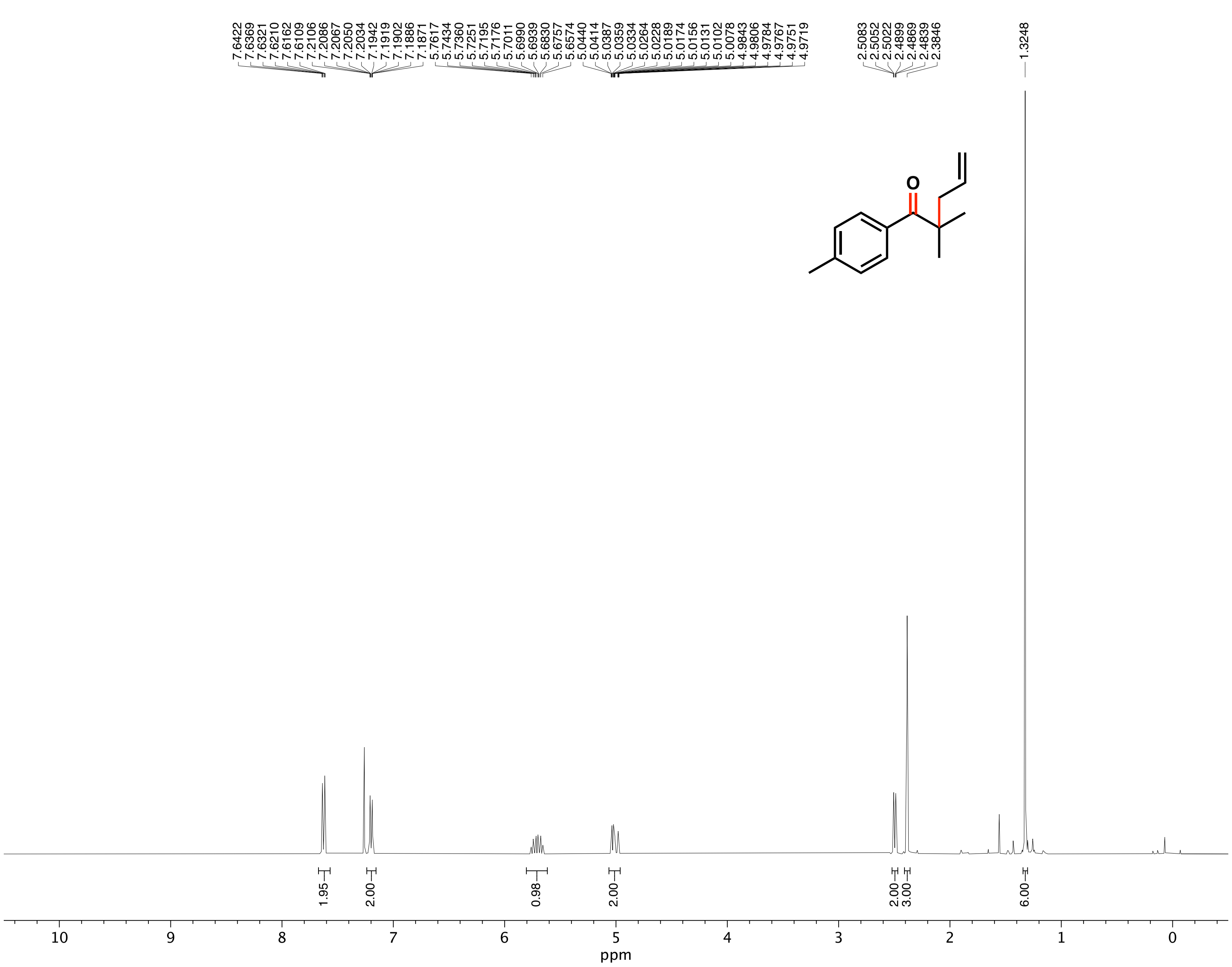

^{1}H NMR (400 MHz, $CDCl_3$) of compound **2k**.

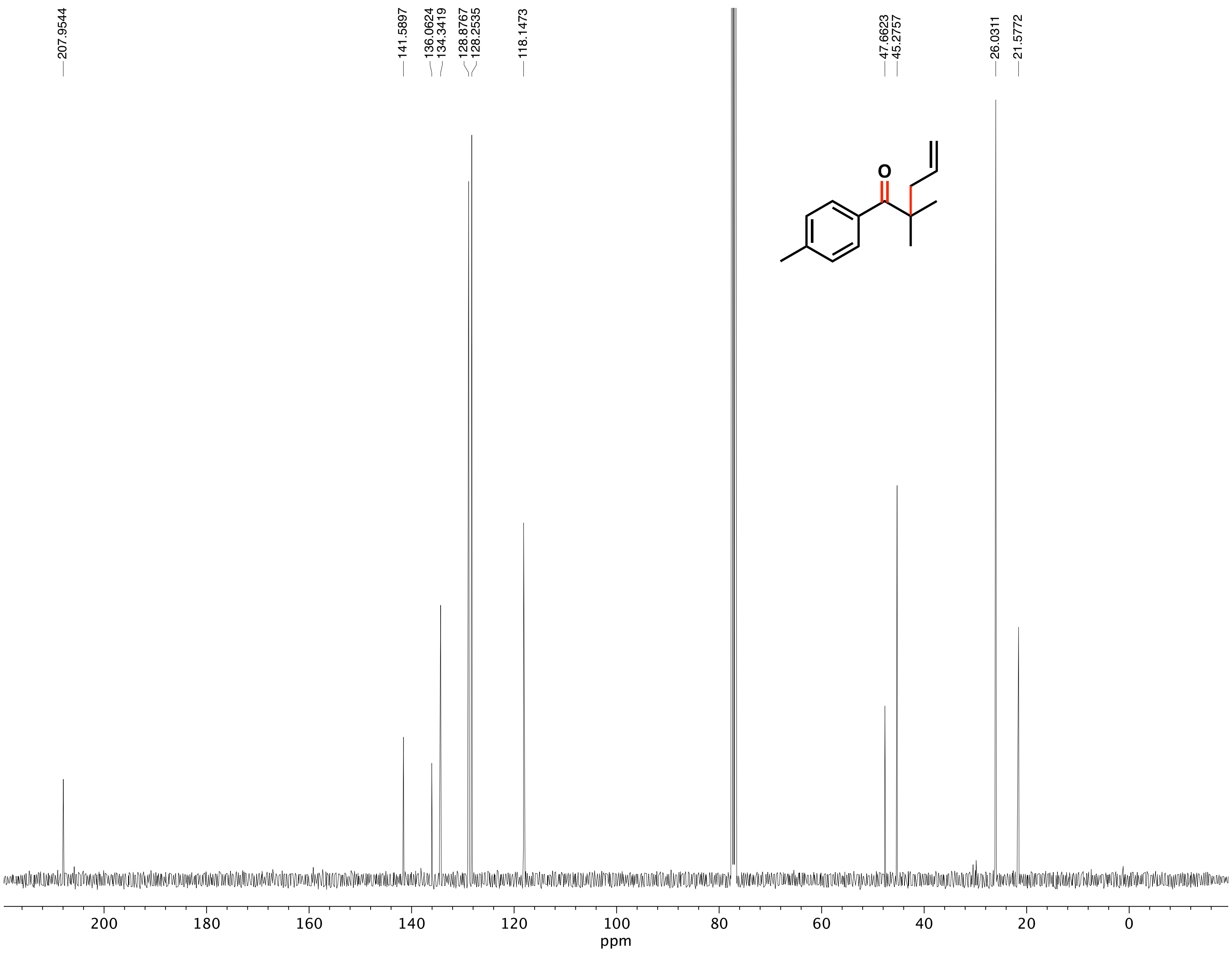

^{13}C NMR (101 MHz, $CDCl_3$) of compound **2k**.

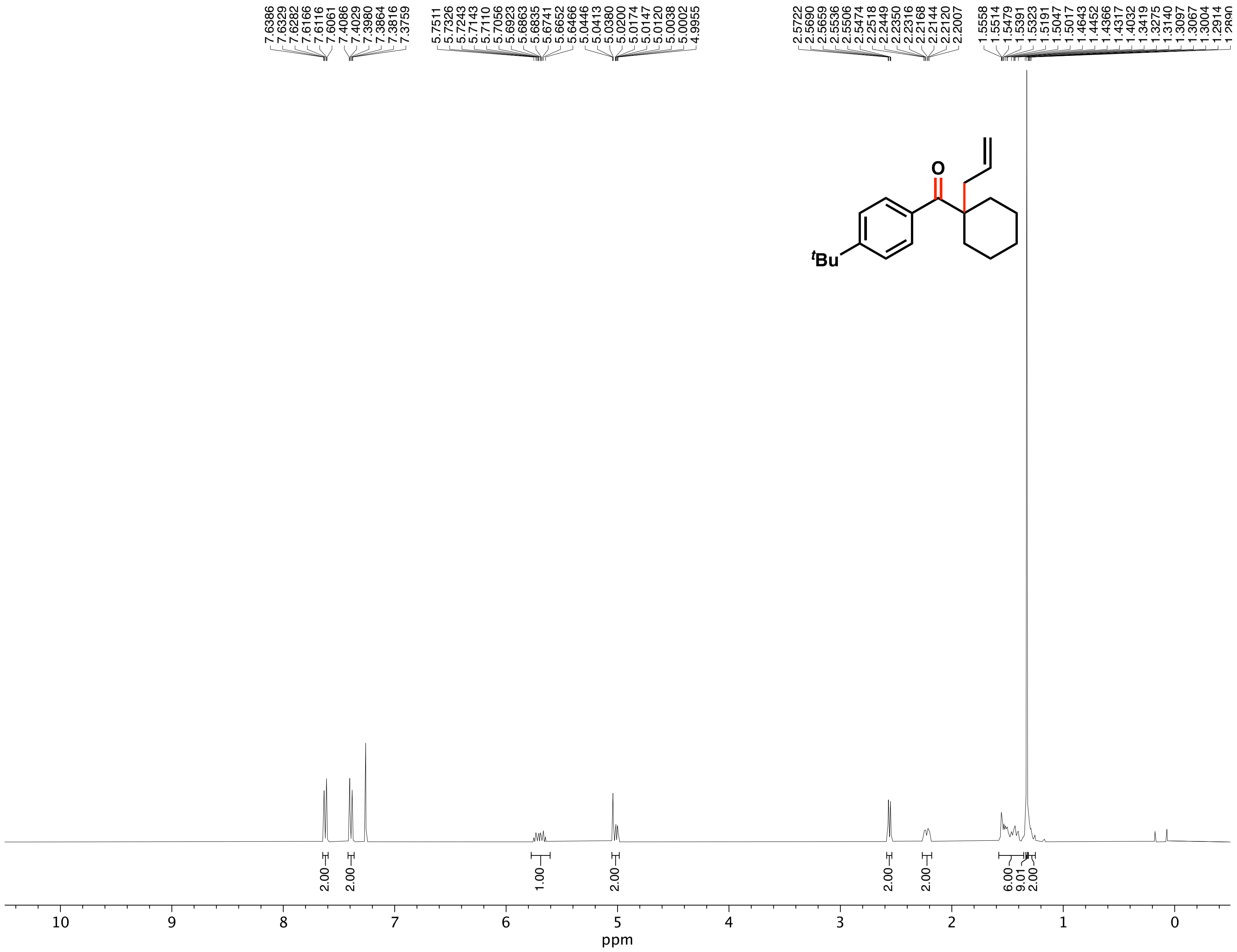

^{1}H NMR (400 MHz, $CDCl_3$) of compound **2l**.

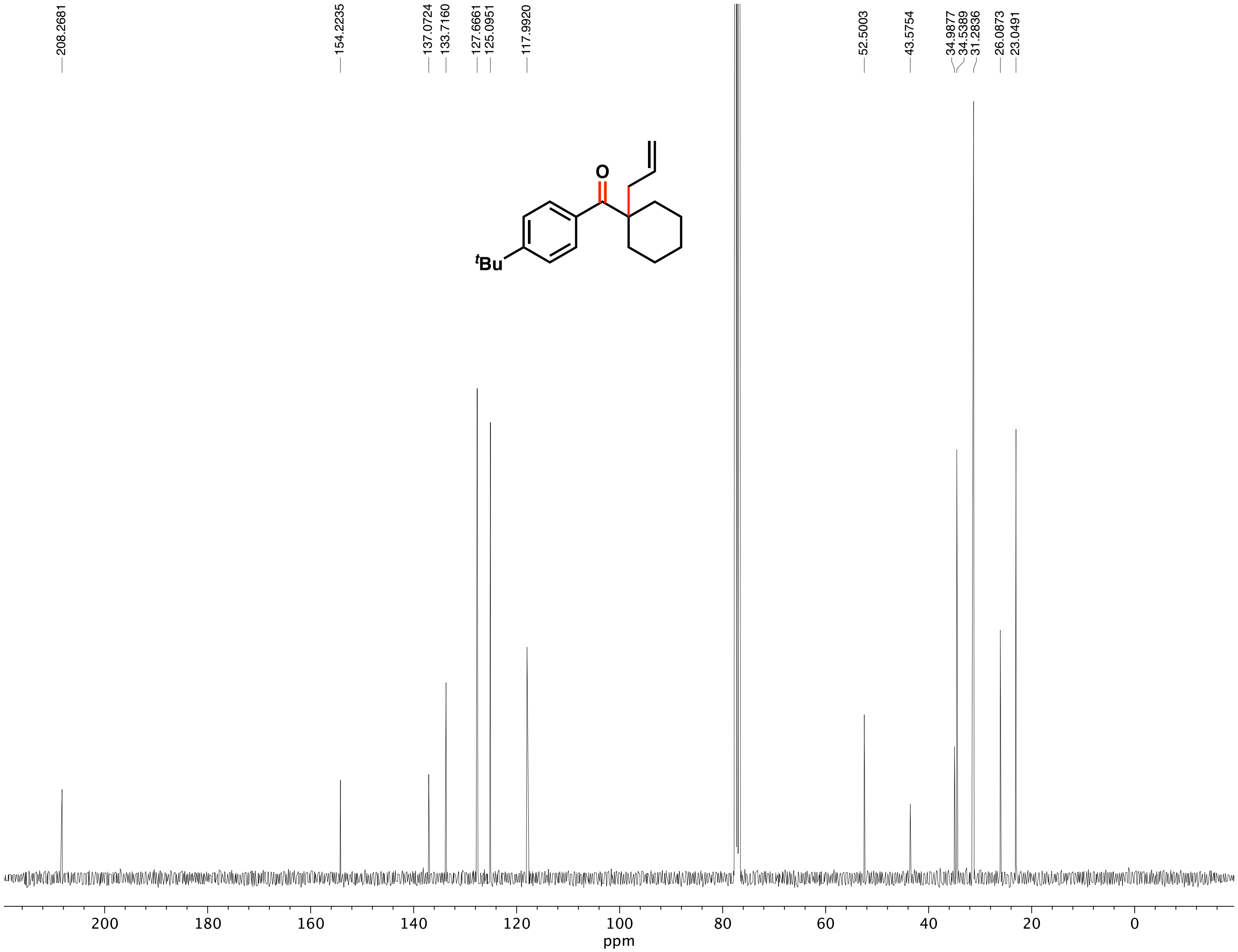

^{13}C NMR (101 MHz, $CDCl_3$) of compound **2l**.

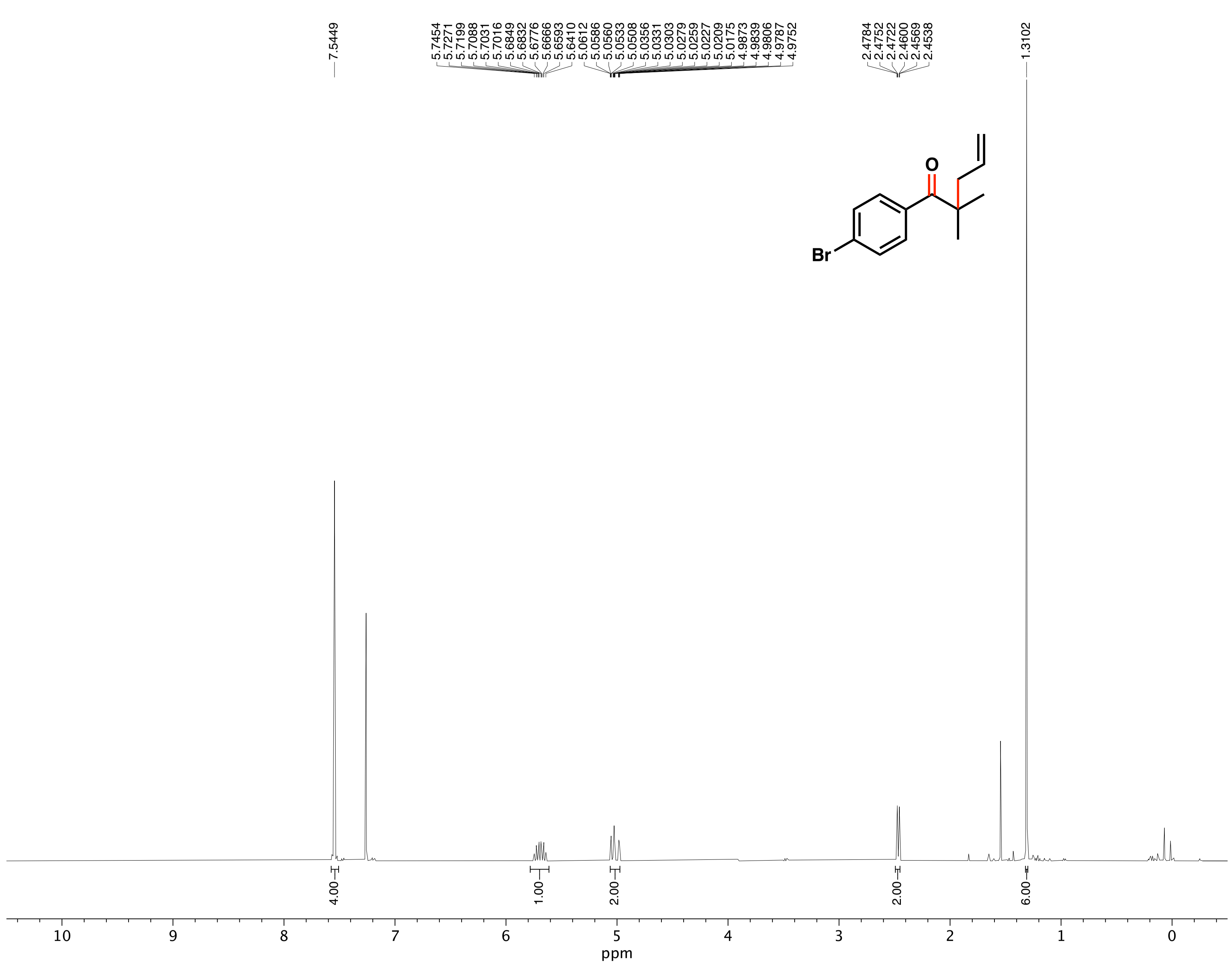

^{1}H NMR (400 MHz, $CDCl_3$) of compound **2m**.

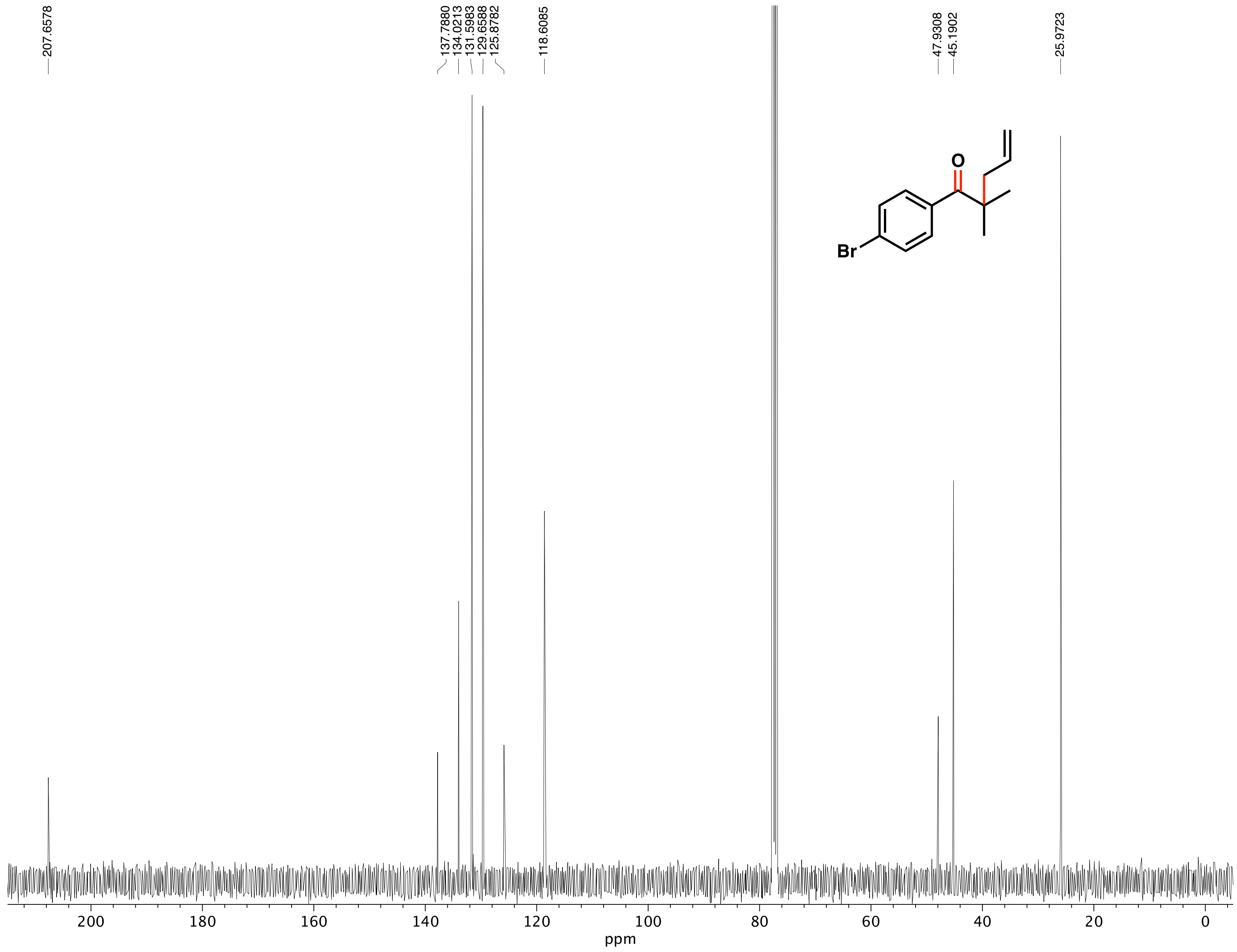

^{13}C NMR (101 MHz, $CDCl_3$) of compound **2m**.

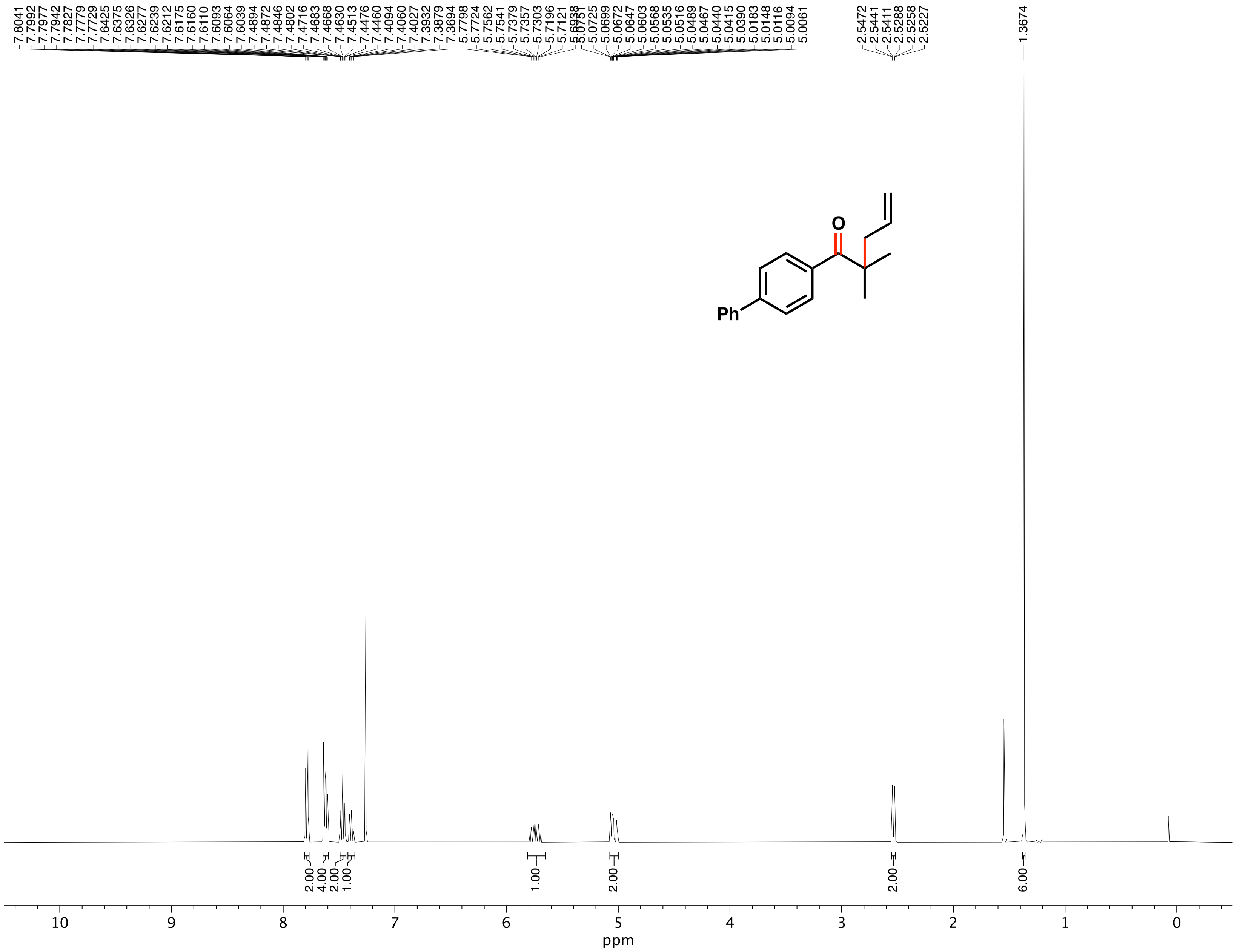

^{1}H NMR (400 MHz, $CDCl_3$) of compound **2n.**

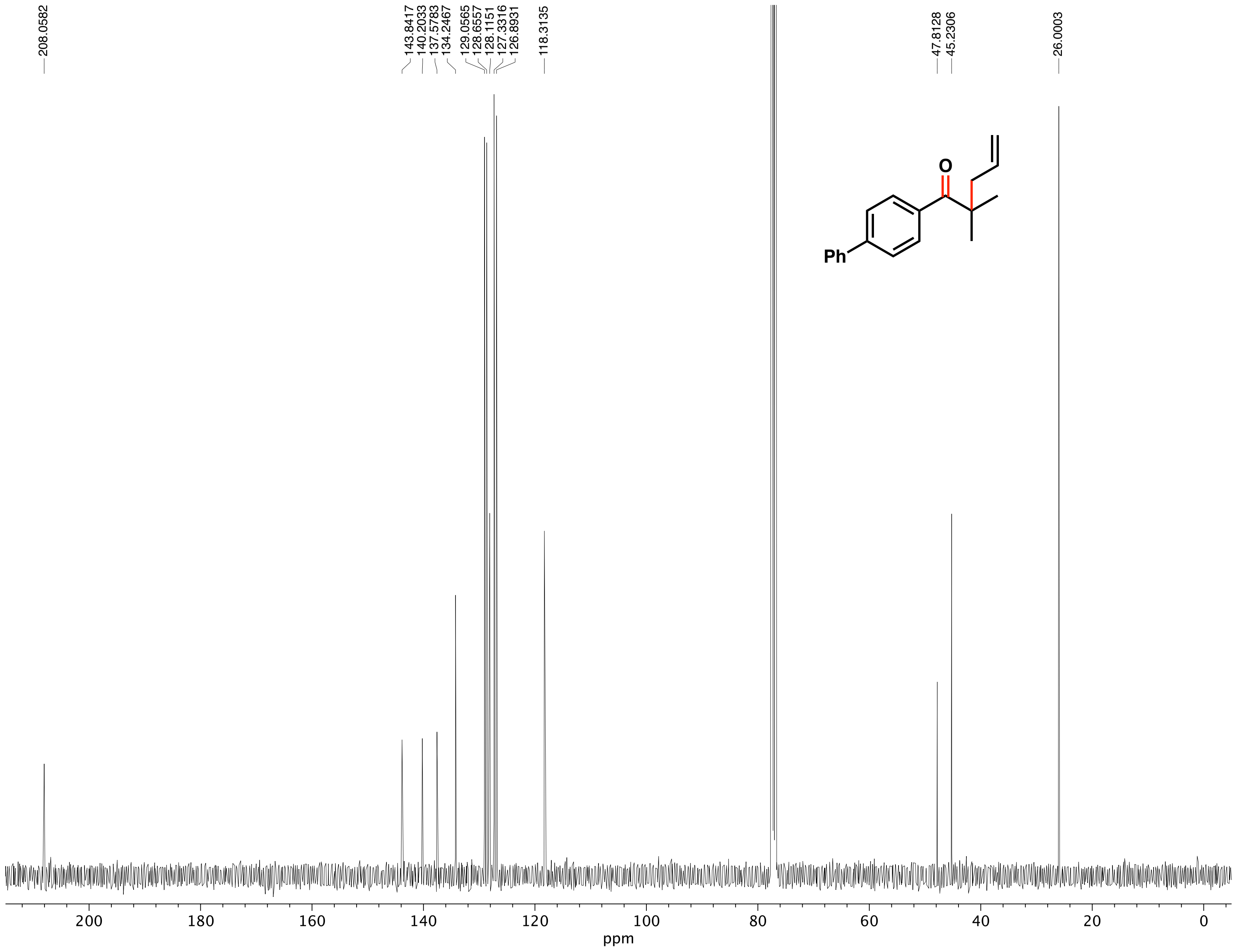

^{13}C NMR (101 MHz, $CDCl_3$) of compound **2n**.

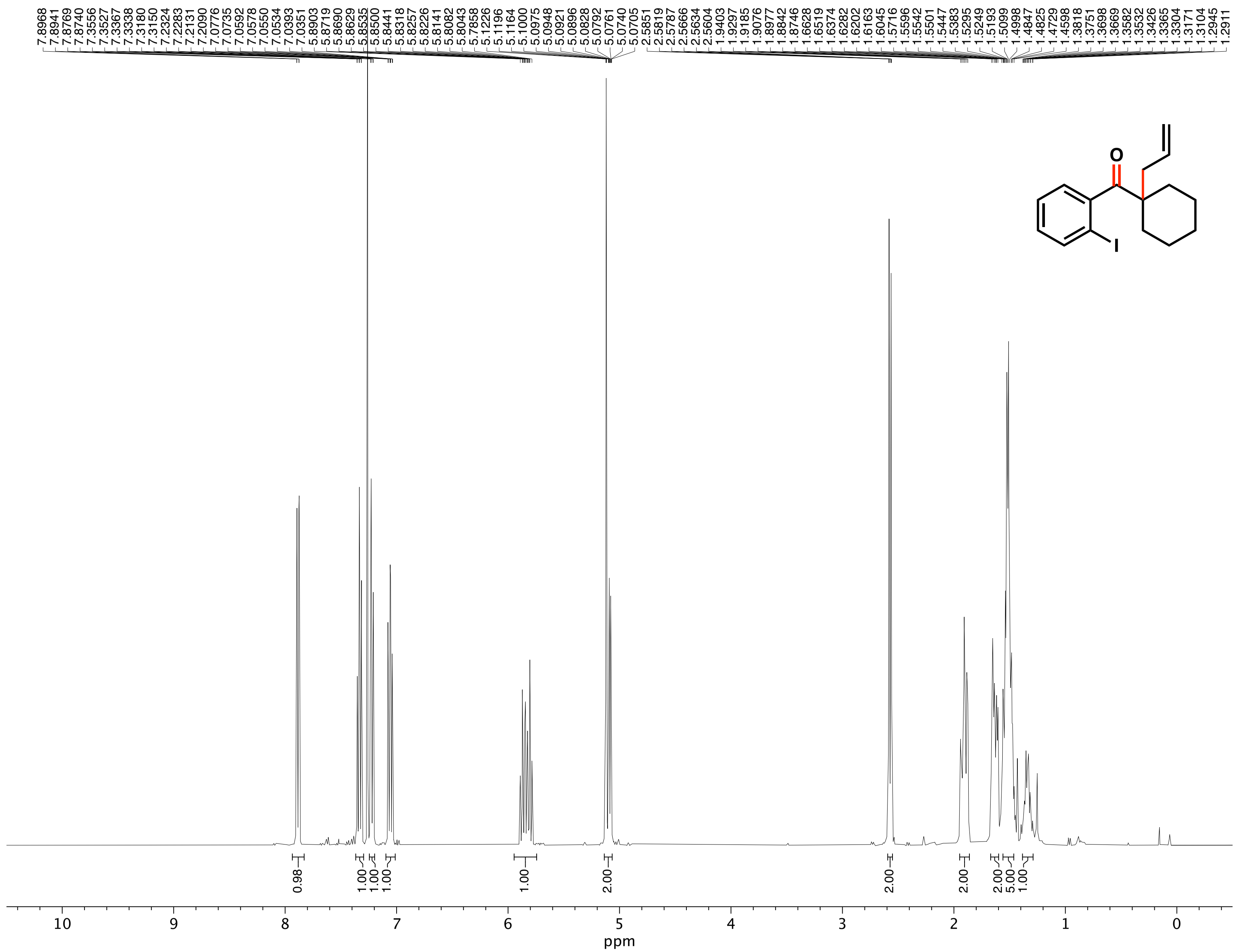

^{1}H NMR (400 MHz, $CDCl_3$) of compound **2o**.

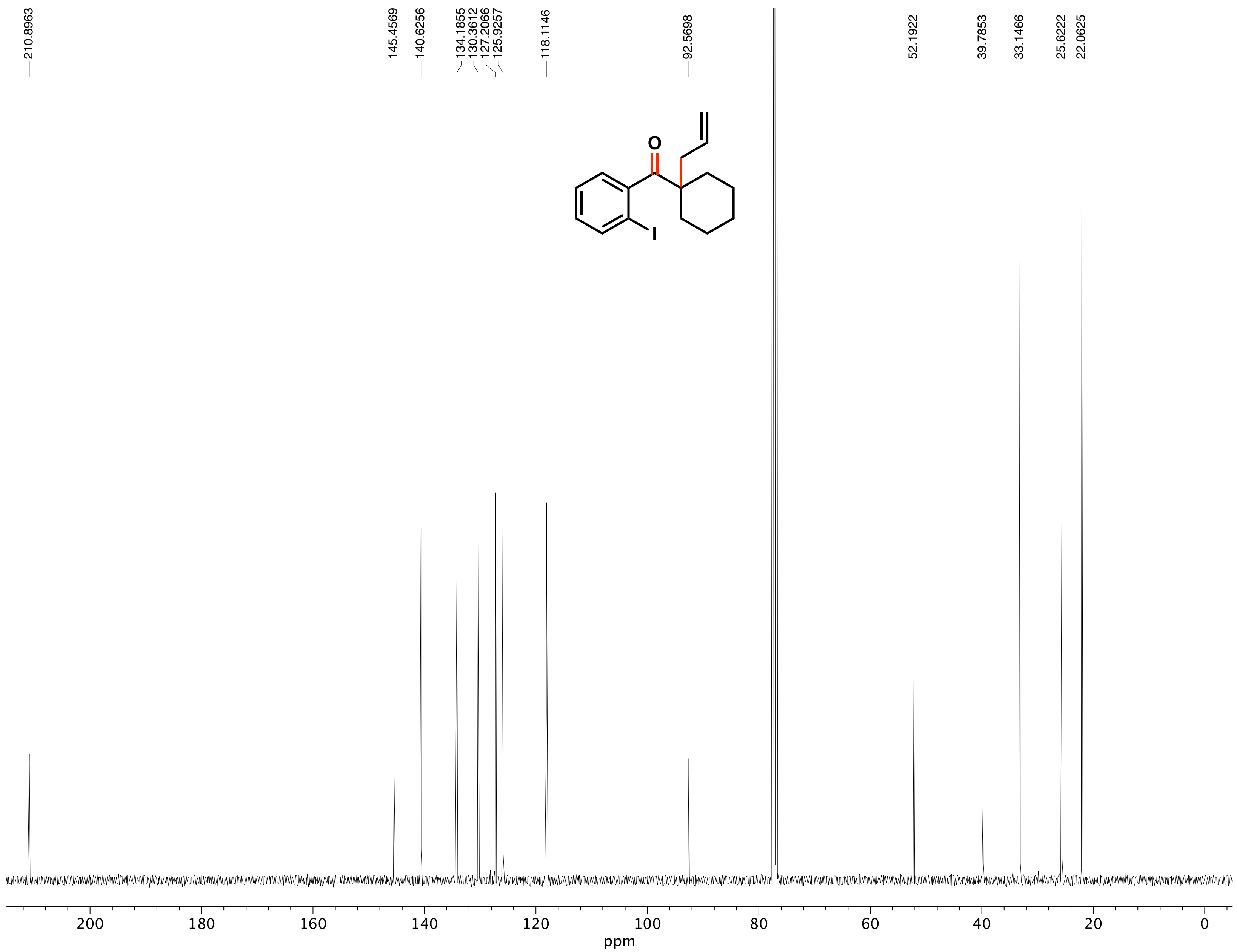

^{13}C NMR (101 MHz, $CDCl_3$) of compound **2o**.

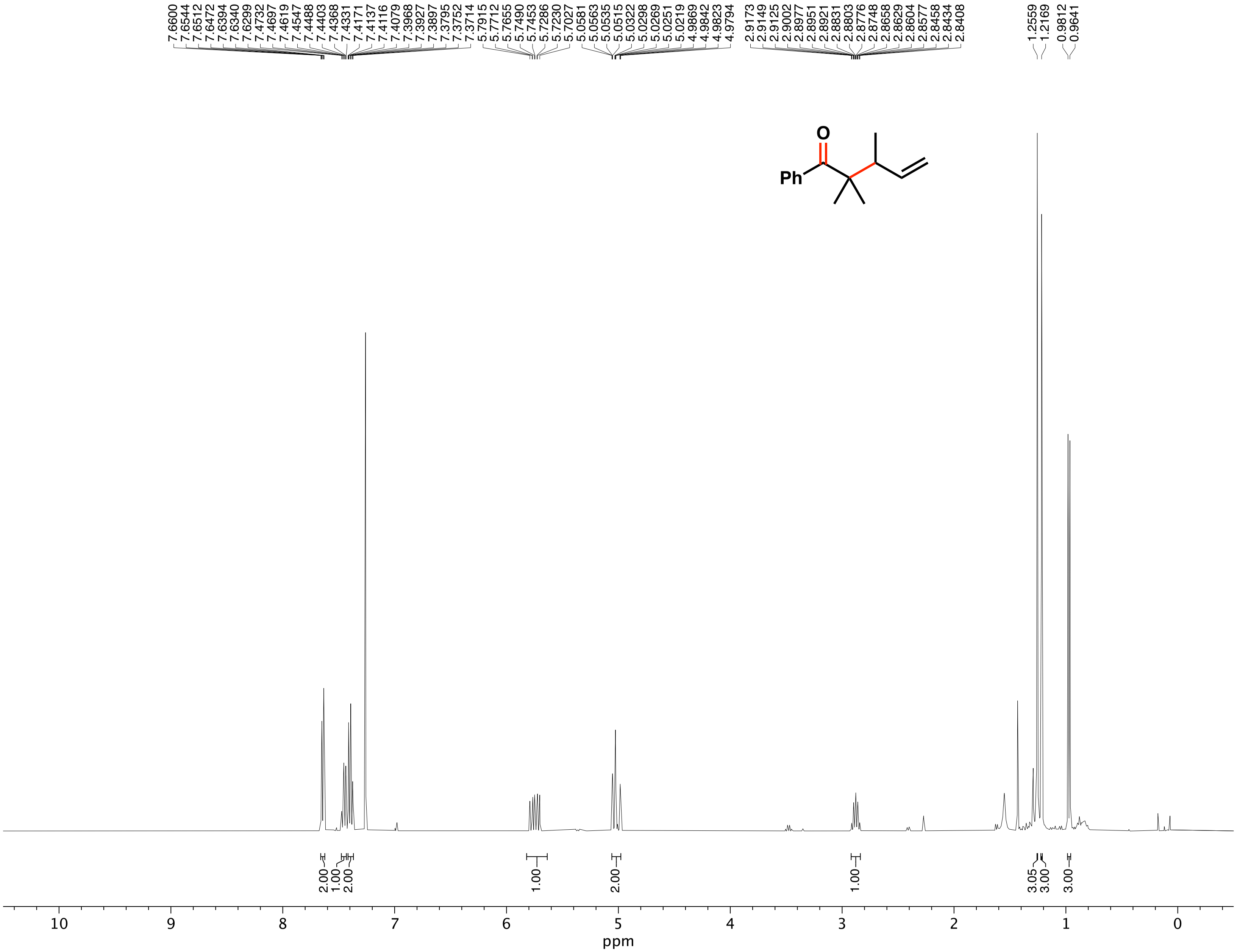

^{1}H NMR (400 MHz, $CDCl_3$) of compound **2p**.

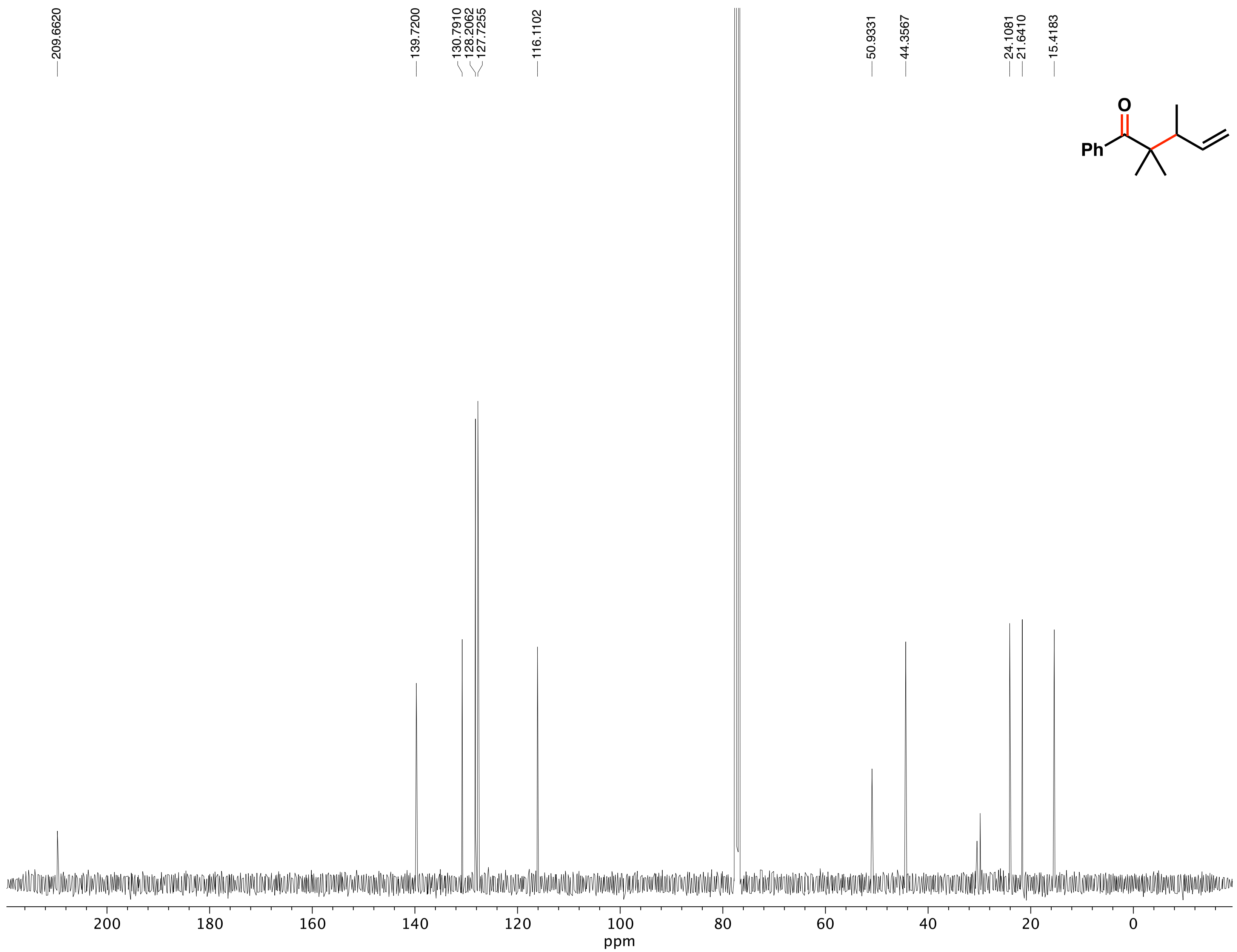

^{13}C NMR (101 MHz, $CDCl_3$) of compound **2p.**

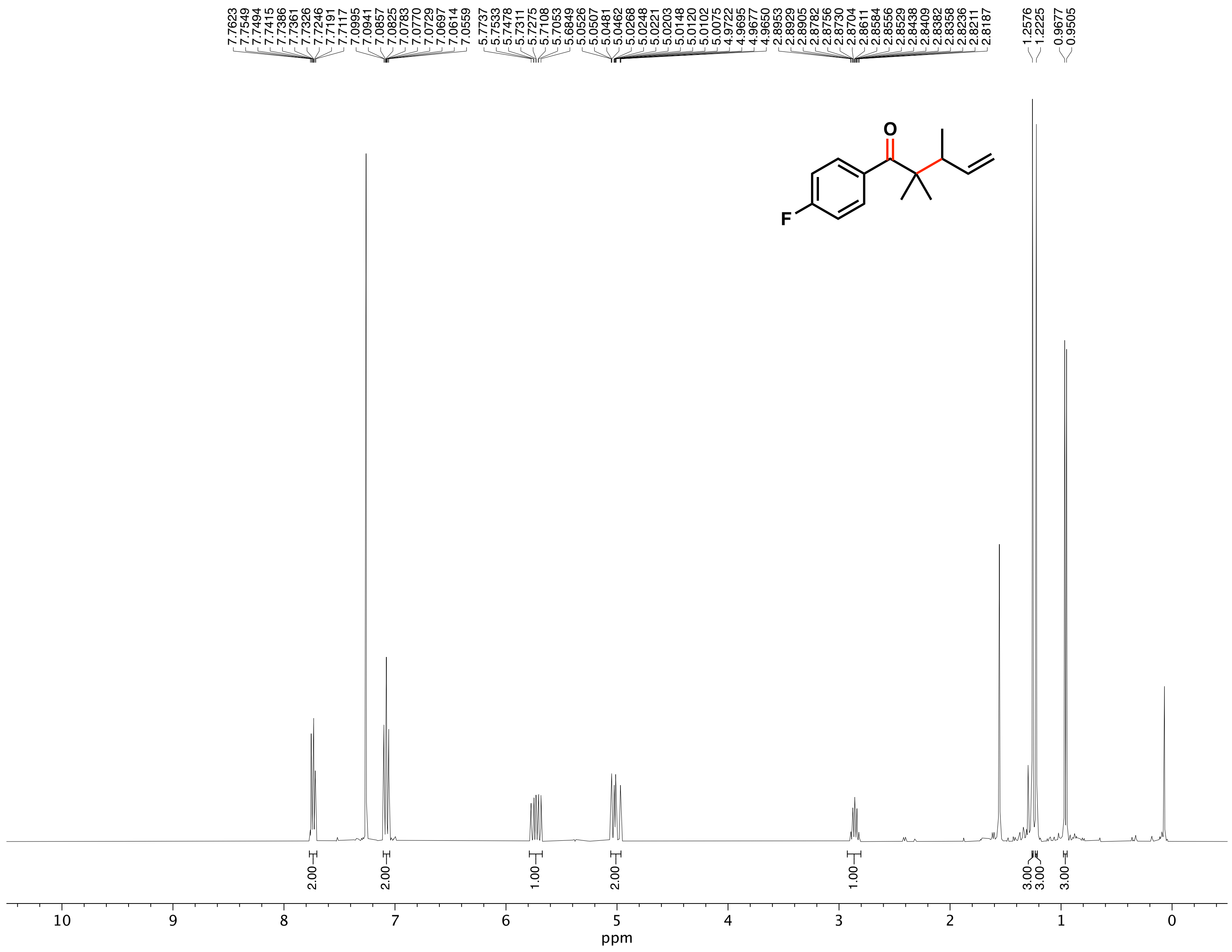

^{1}H NMR (400 MHz, $CDCl_3$) of compound **2q.**

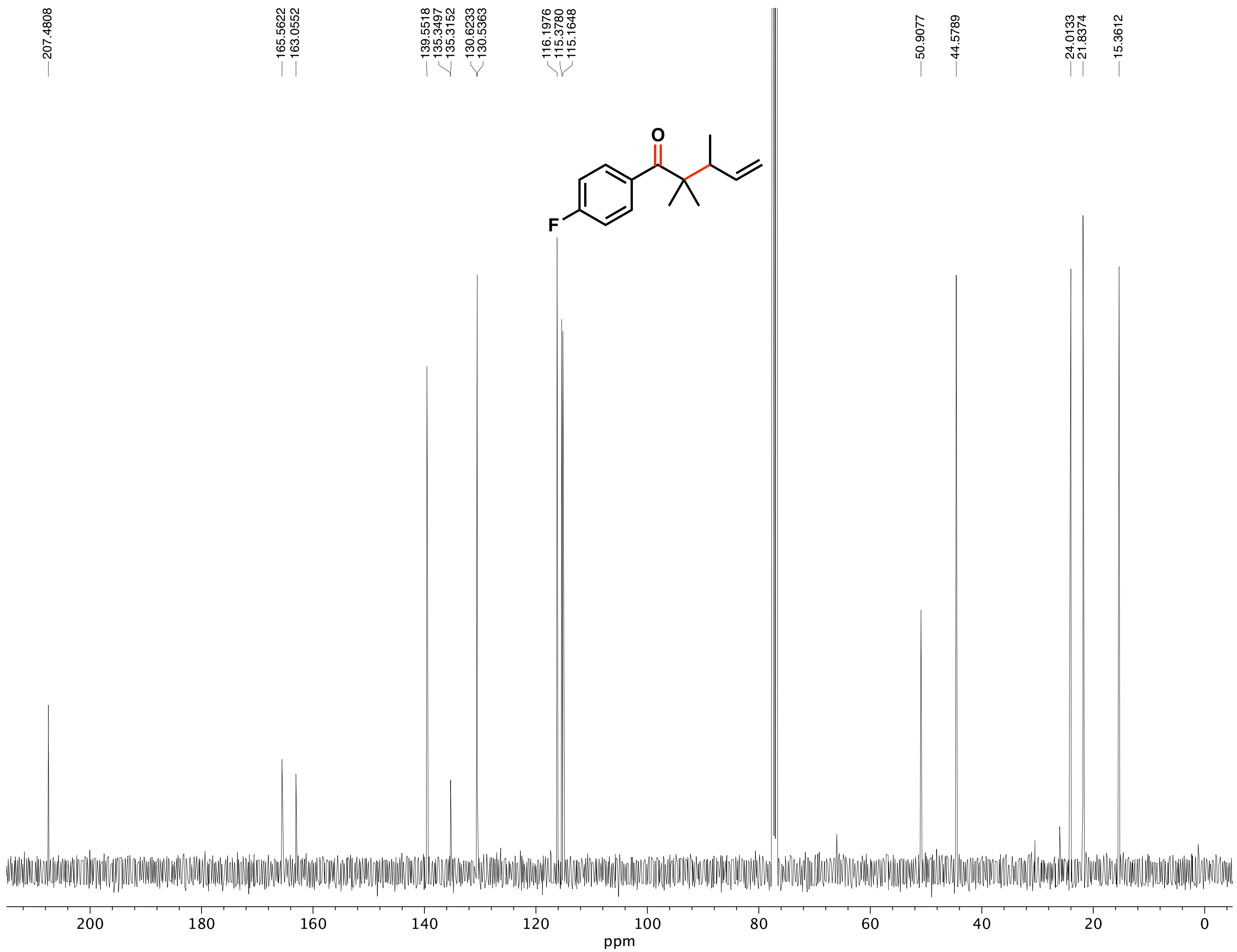

^{13}C NMR (101 MHz, $CDCl_3$) of compound **2q**.

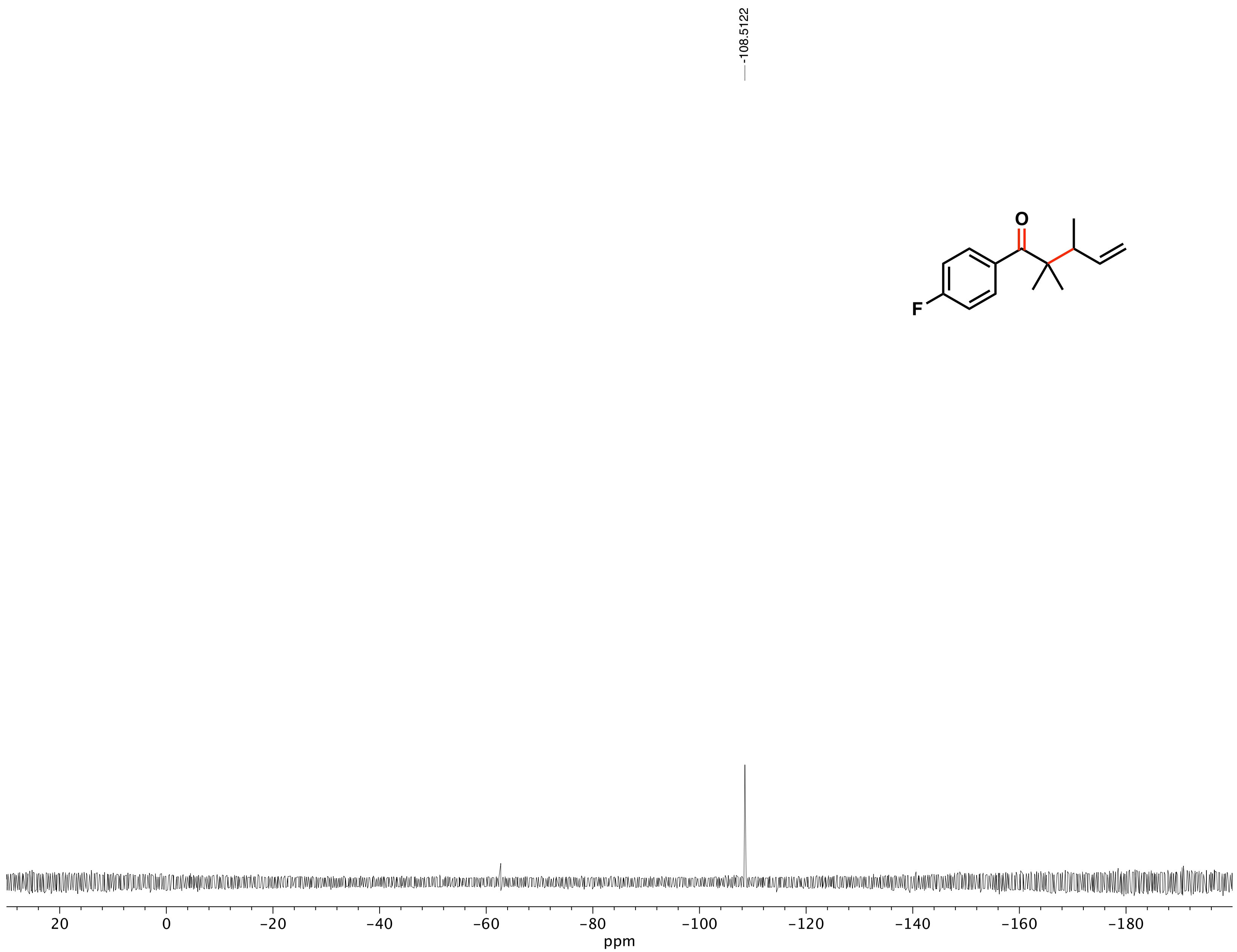

^{19}F NMR (282 MHz, $CDCl_3$) of compound **2q**.

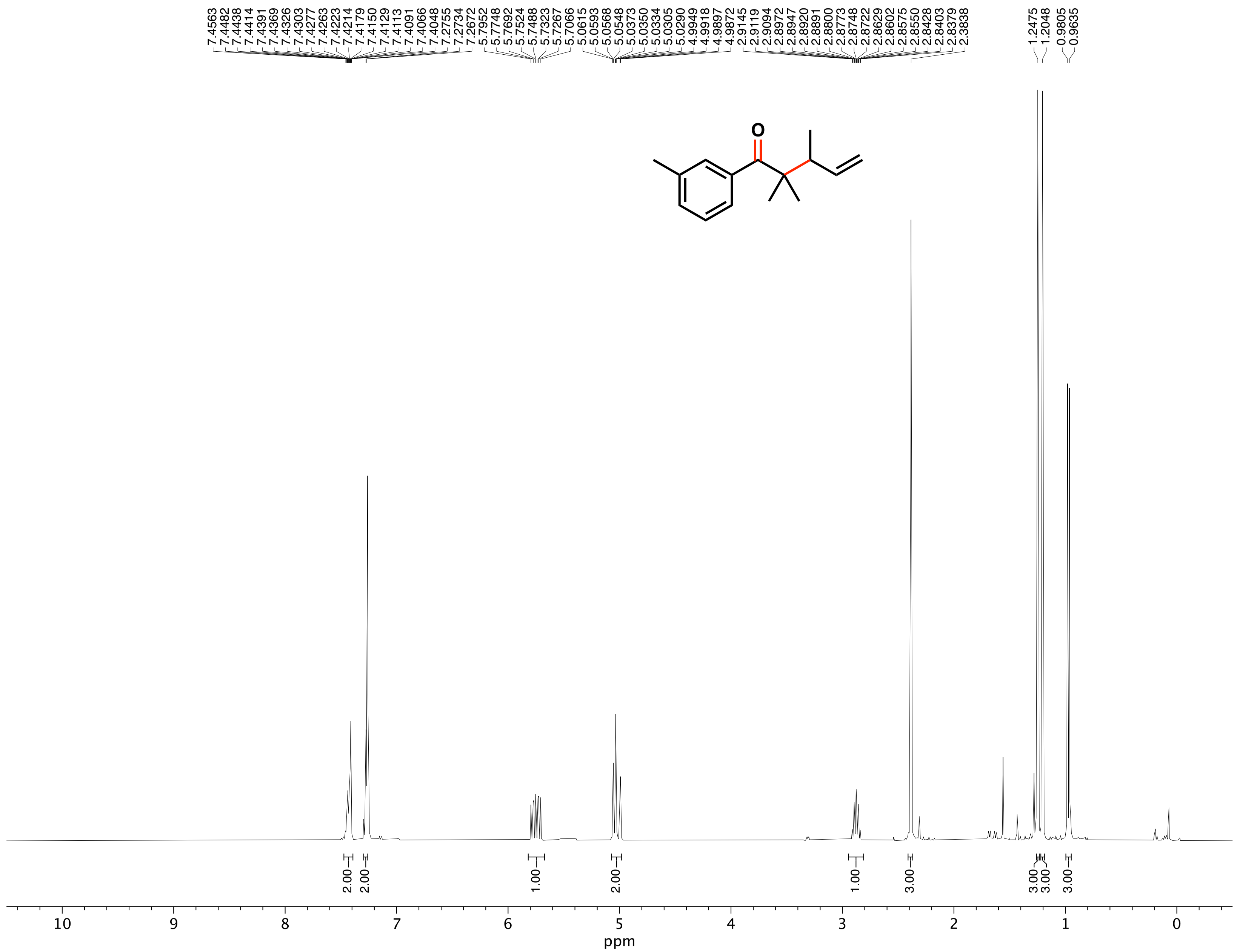

^{1}H NMR (400 MHz, $CDCl_3$) of compound **2r.**

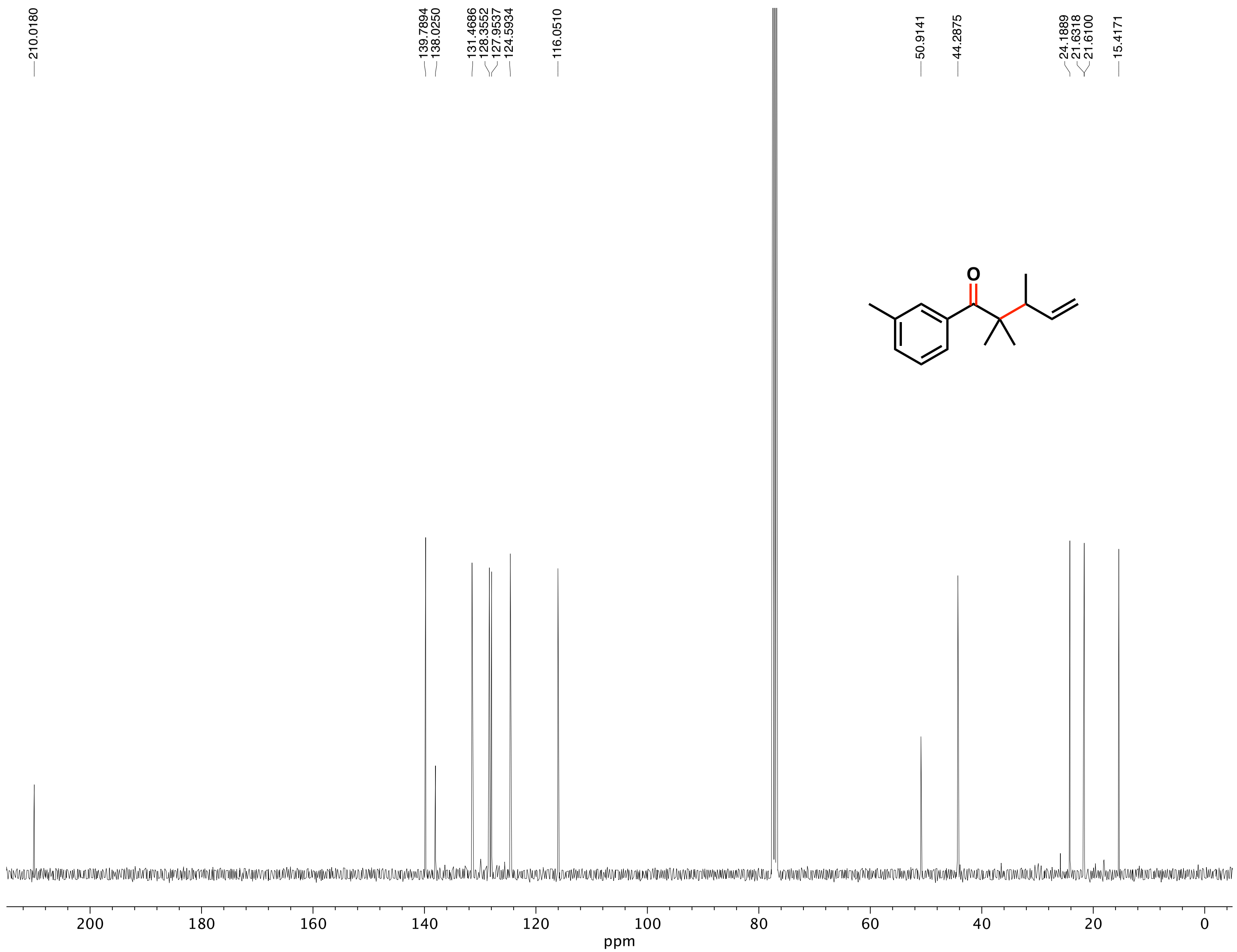

^{13}C NMR (101 MHz, $CDCl_3$) of compound **2r**.

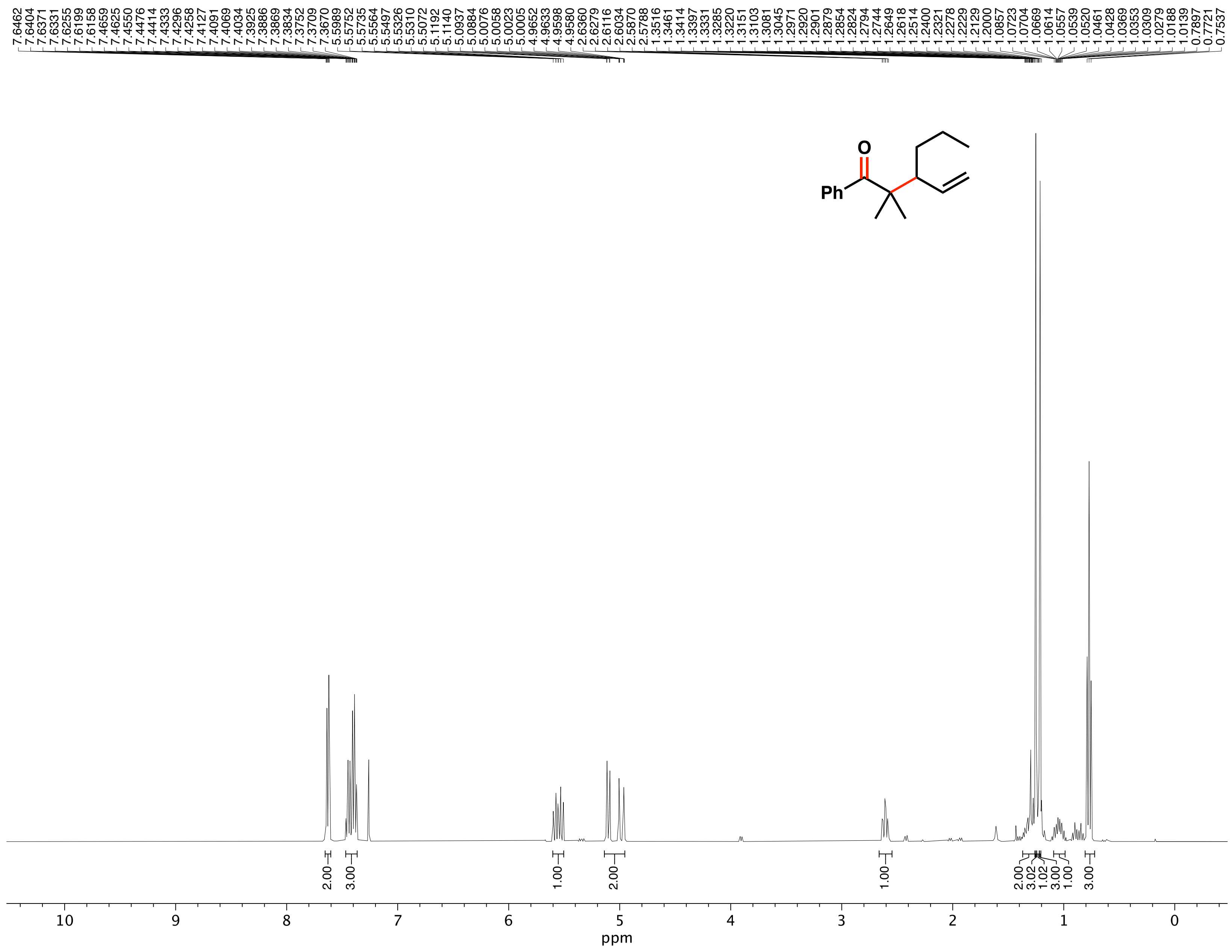

^{1}H NMR (400 MHz, $CDCl_3$) of compound **2s**.

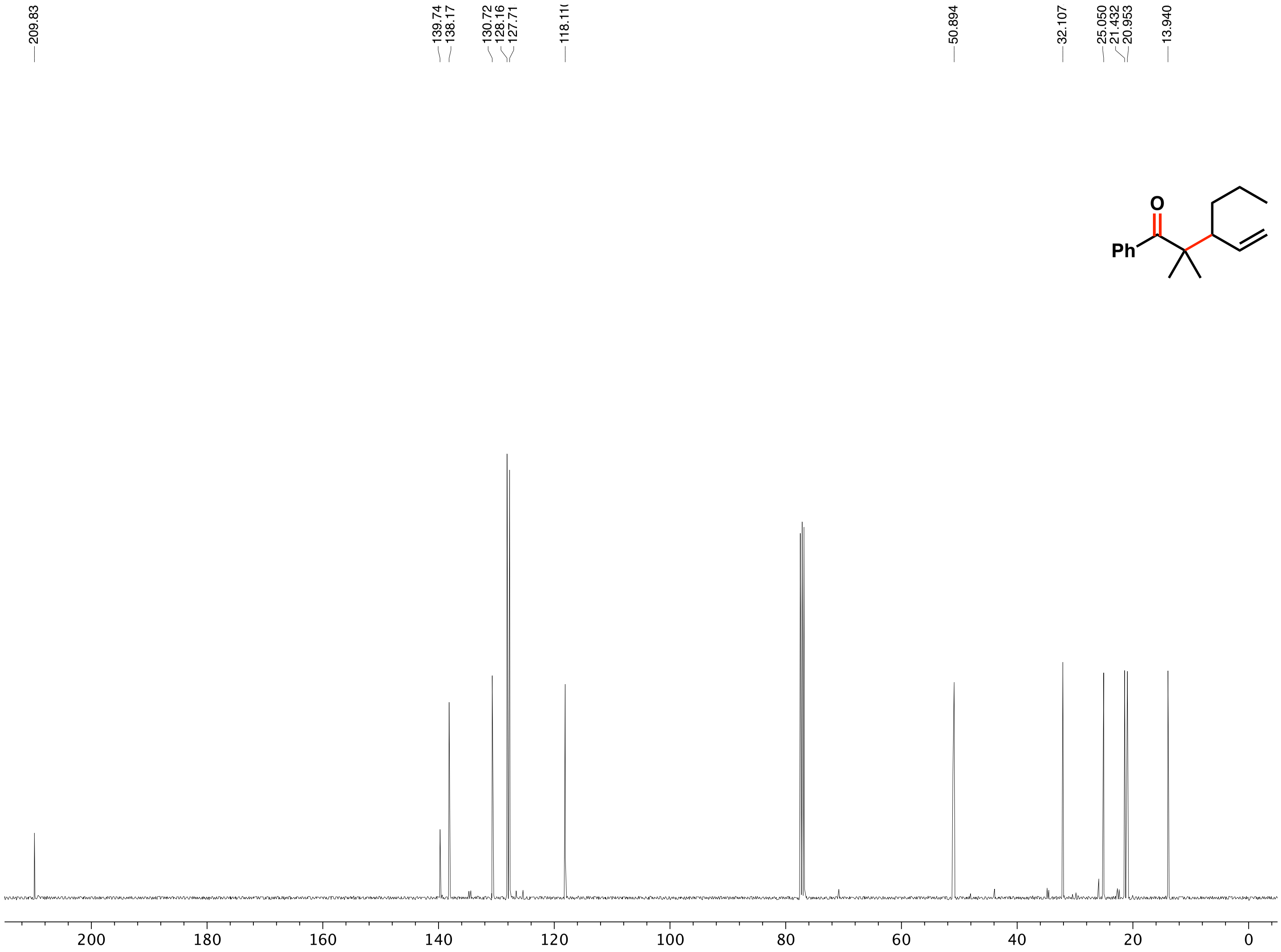

^{13}C NMR (101 MHz, $CDCl_3$) of compound **2s**.

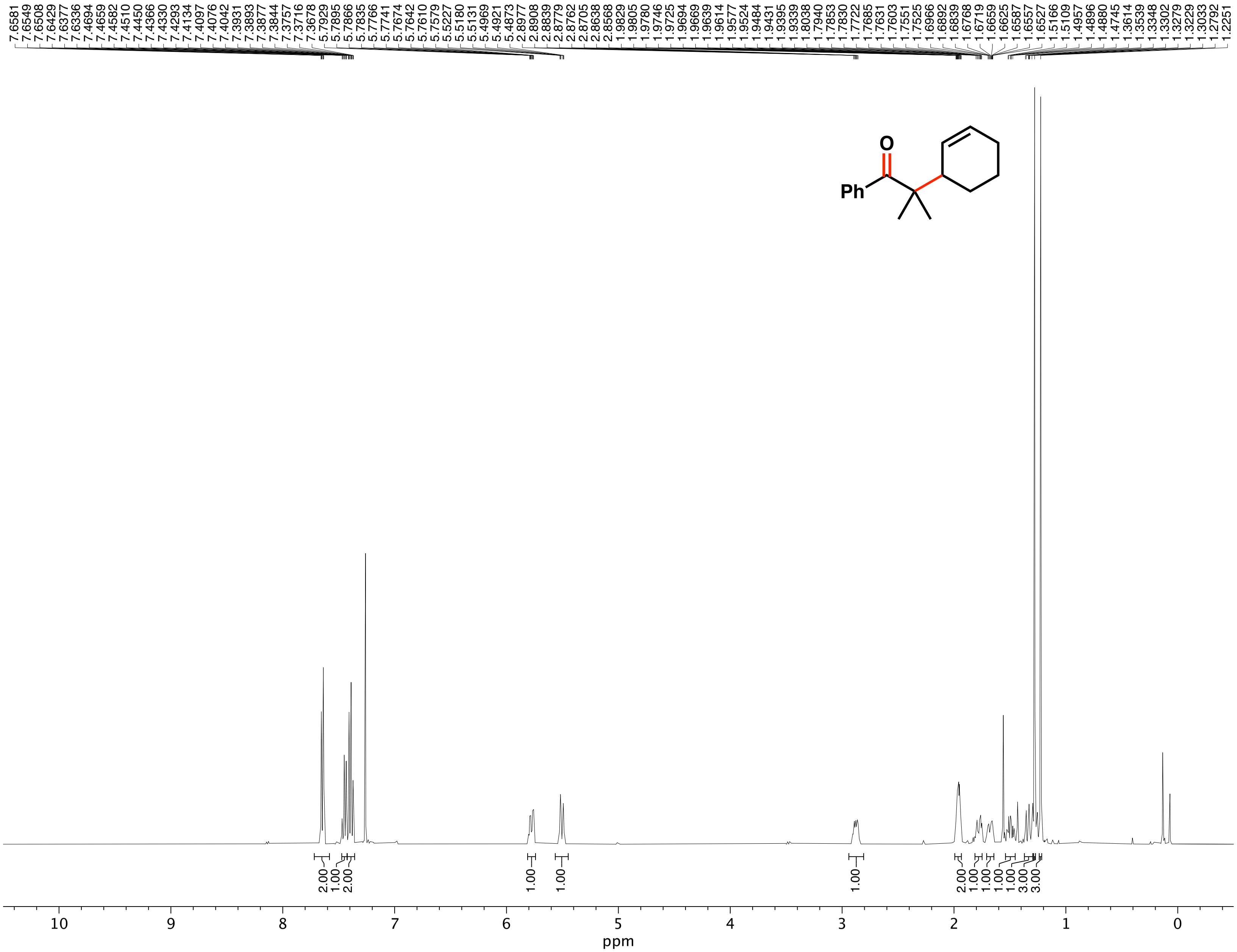

^{1}H NMR (400 MHz, $CDCl_3$) of compound **2t**.

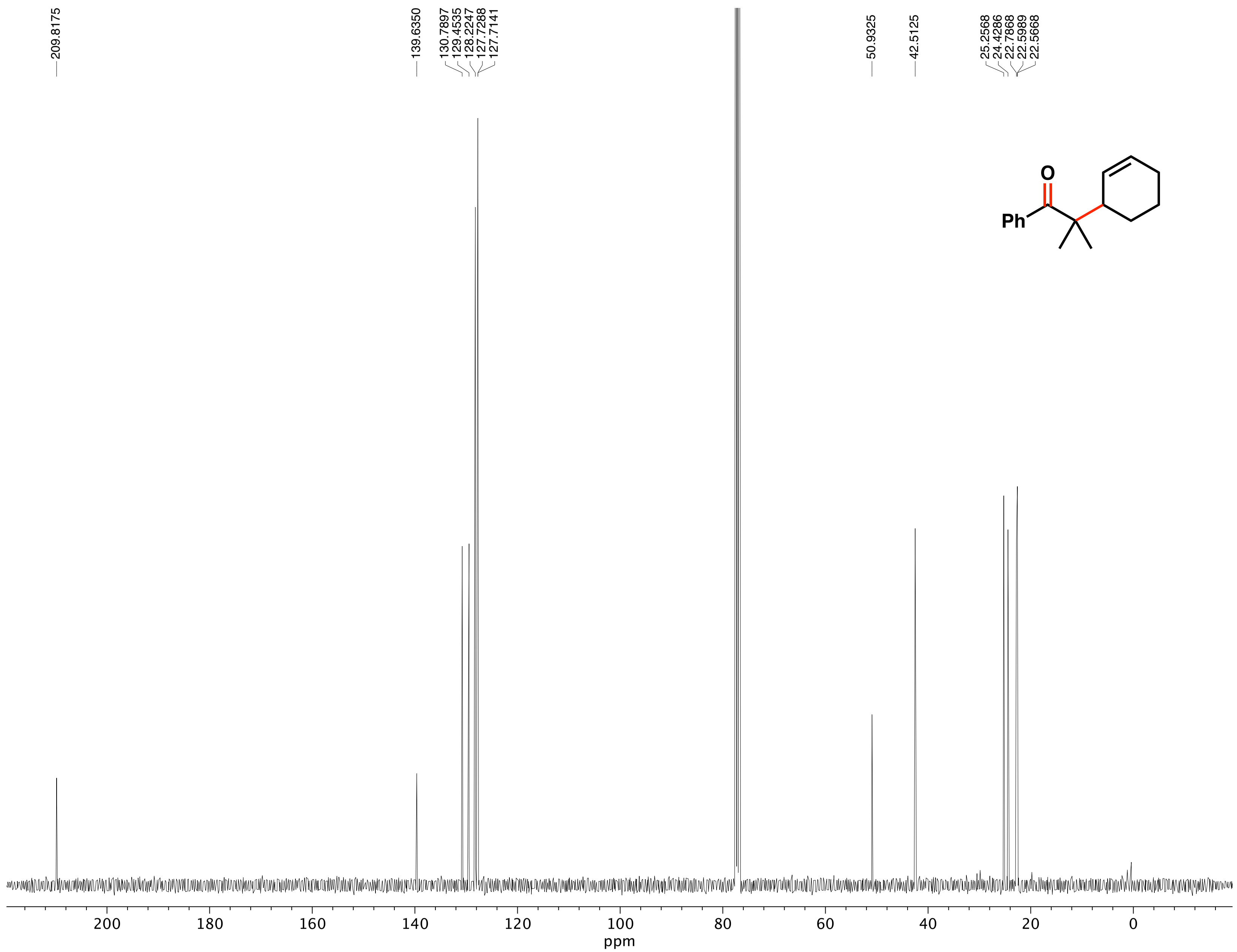

^{13}C NMR (101 MHz, $CDCl_3$) of compound **2t**.

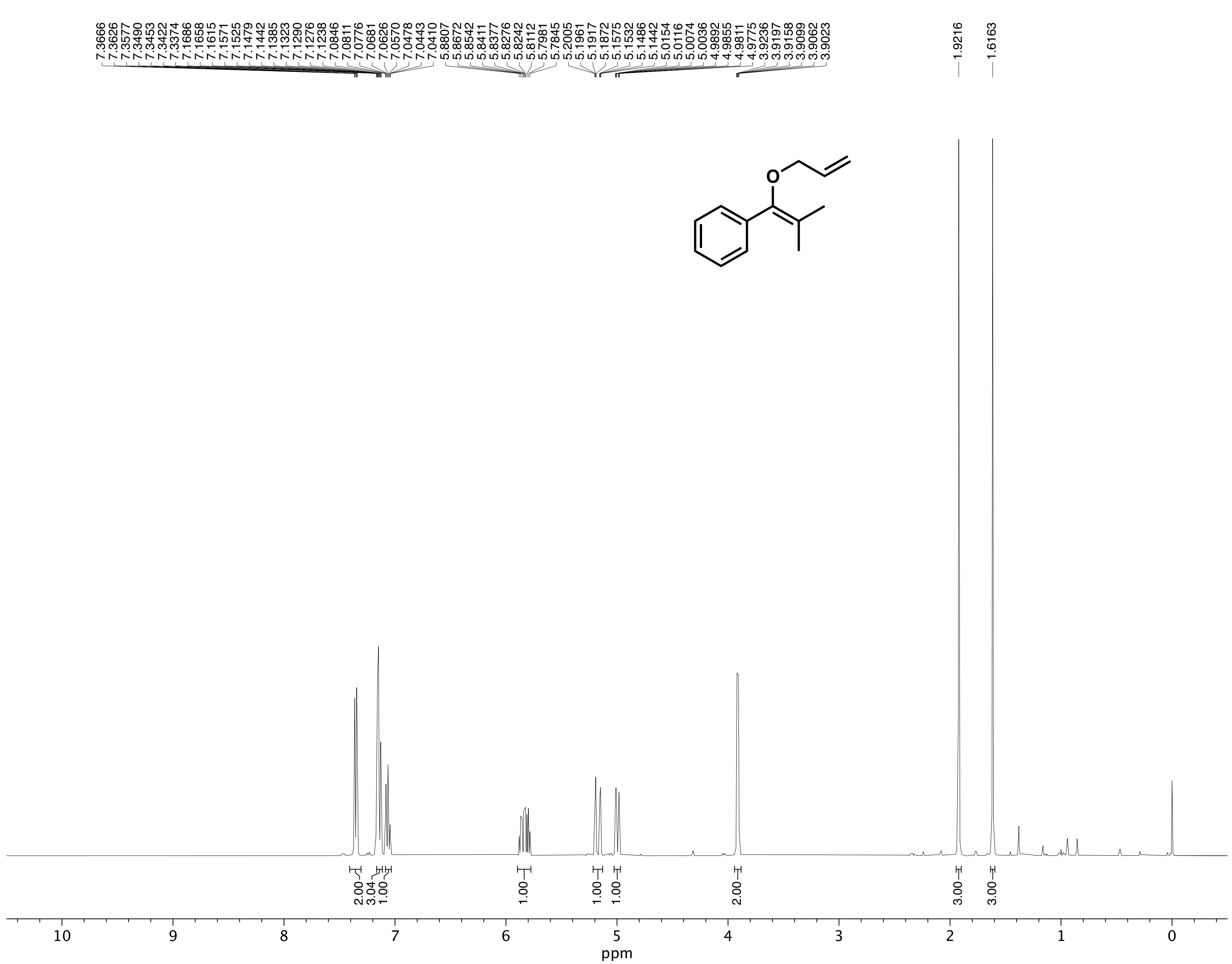

^{1}H NMR (400 MHz, $CDCl_3$) of compound **5**.

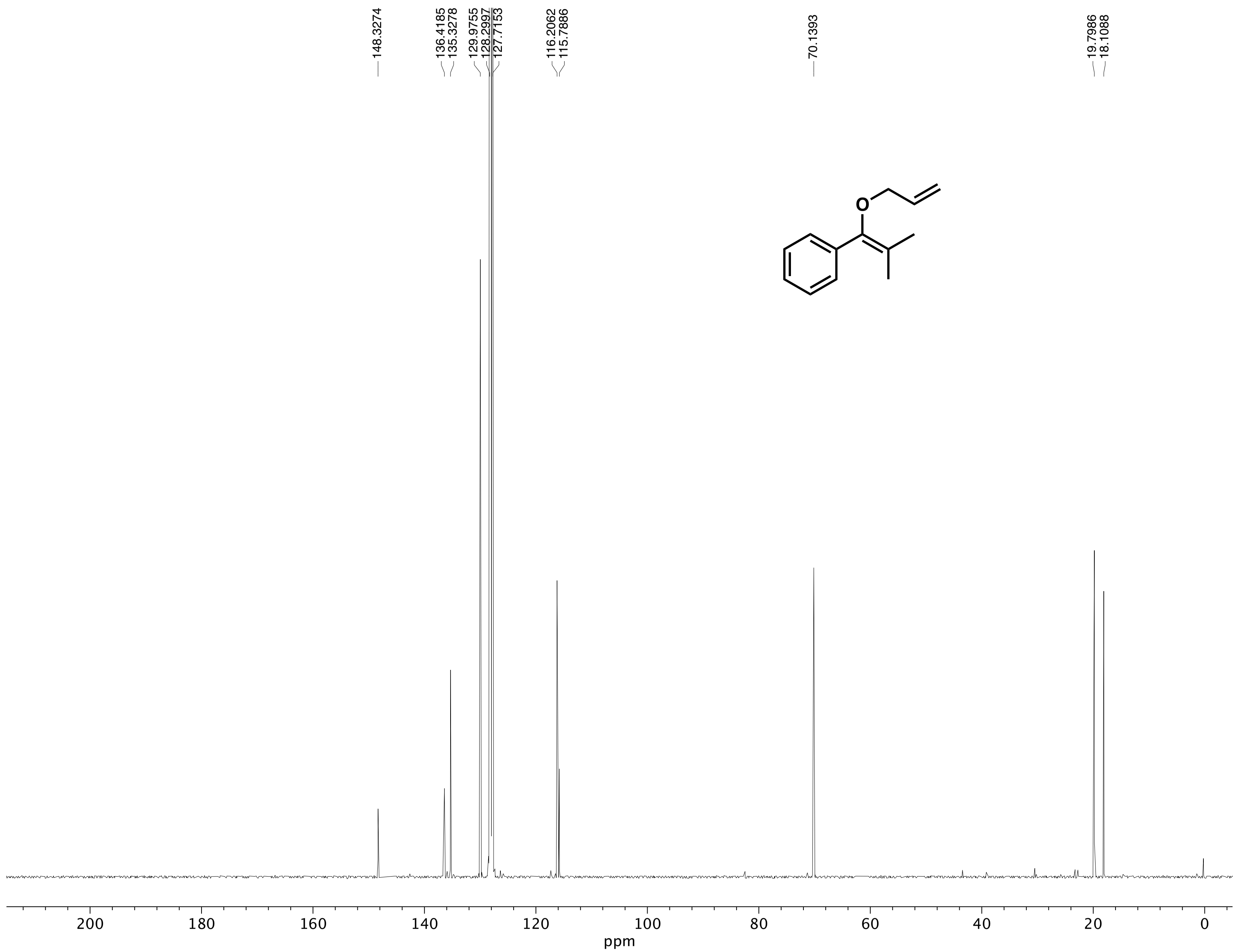

^{13}C NMR (101 MHz, $CDCl_3$) of compound **5**.

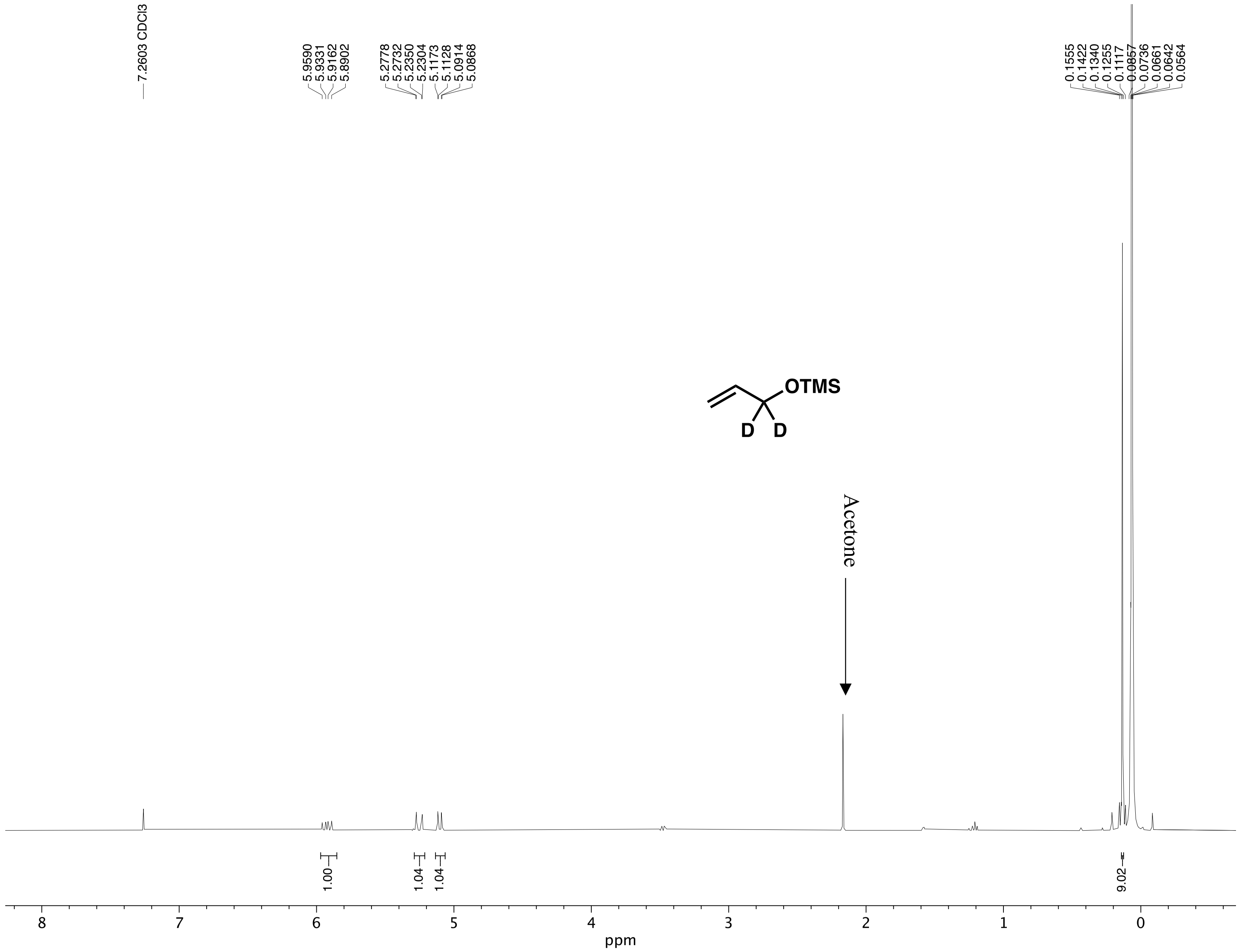

^{1}H NMR (400 MHz, $CDCl_3$) of compound **6**.

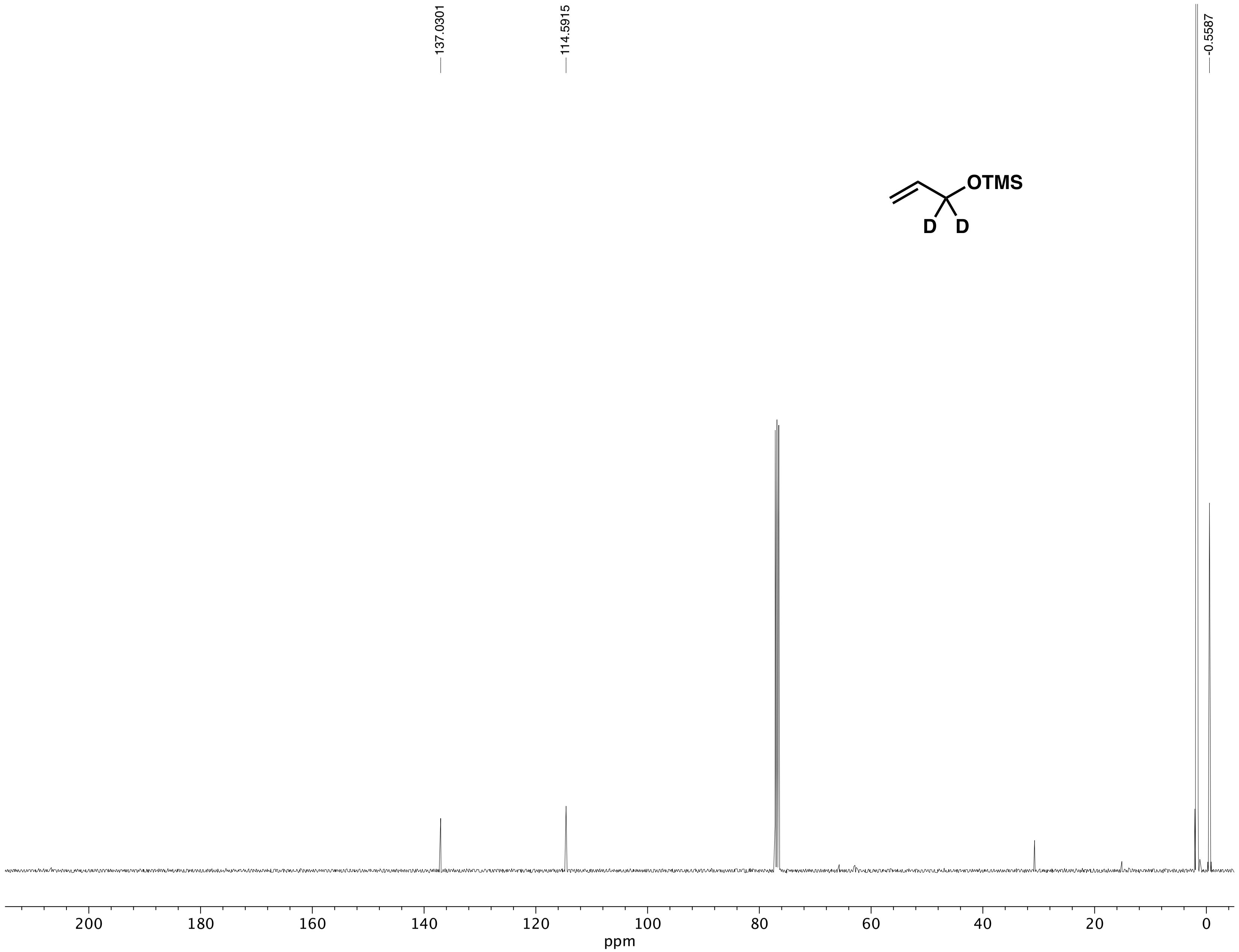

^{13}C NMR (101 MHz, $CDCl_3$) of compound **6**.

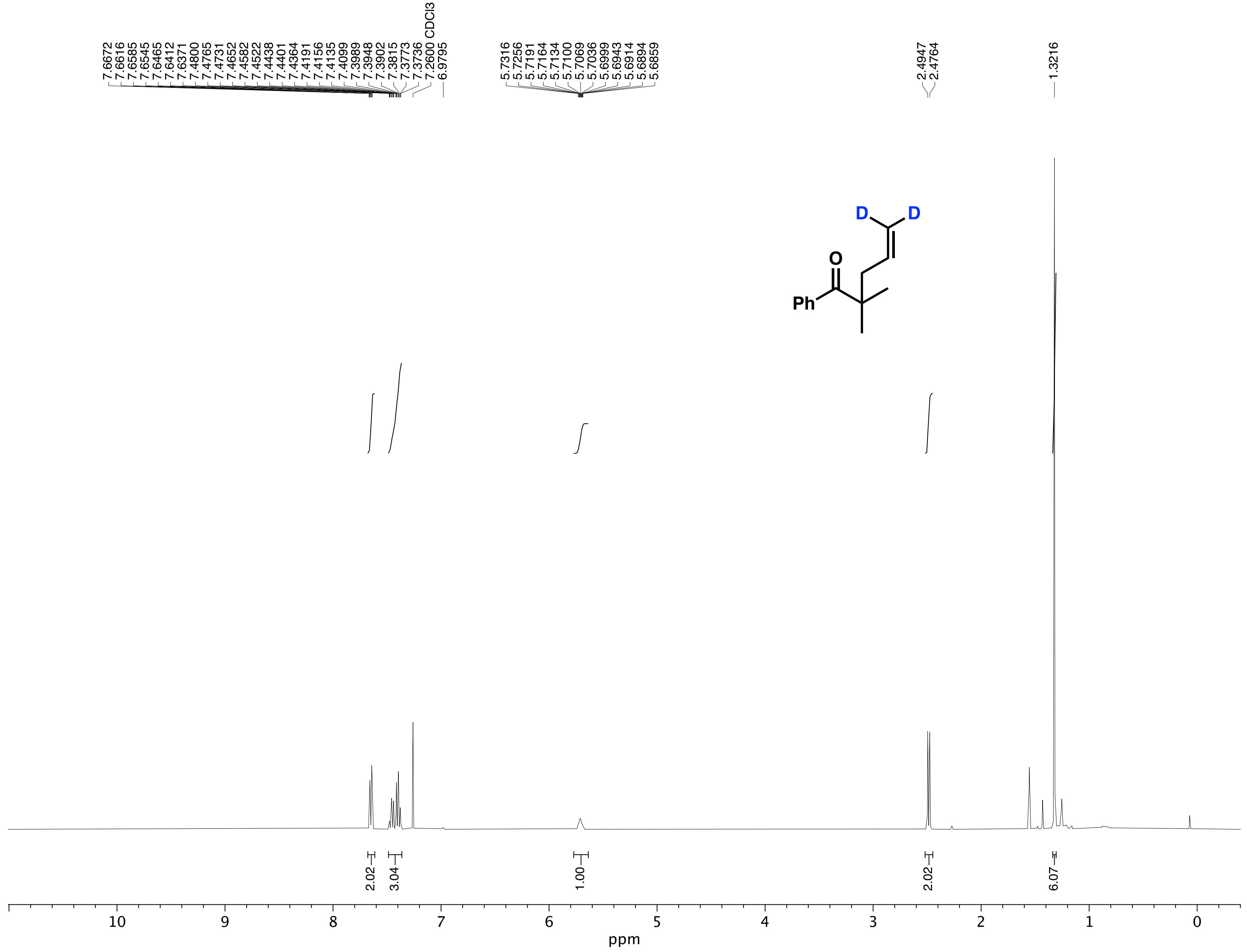

^{1}H NMR (400 MHz, $CDCl_3$) of compound **2a-D$_2$**.

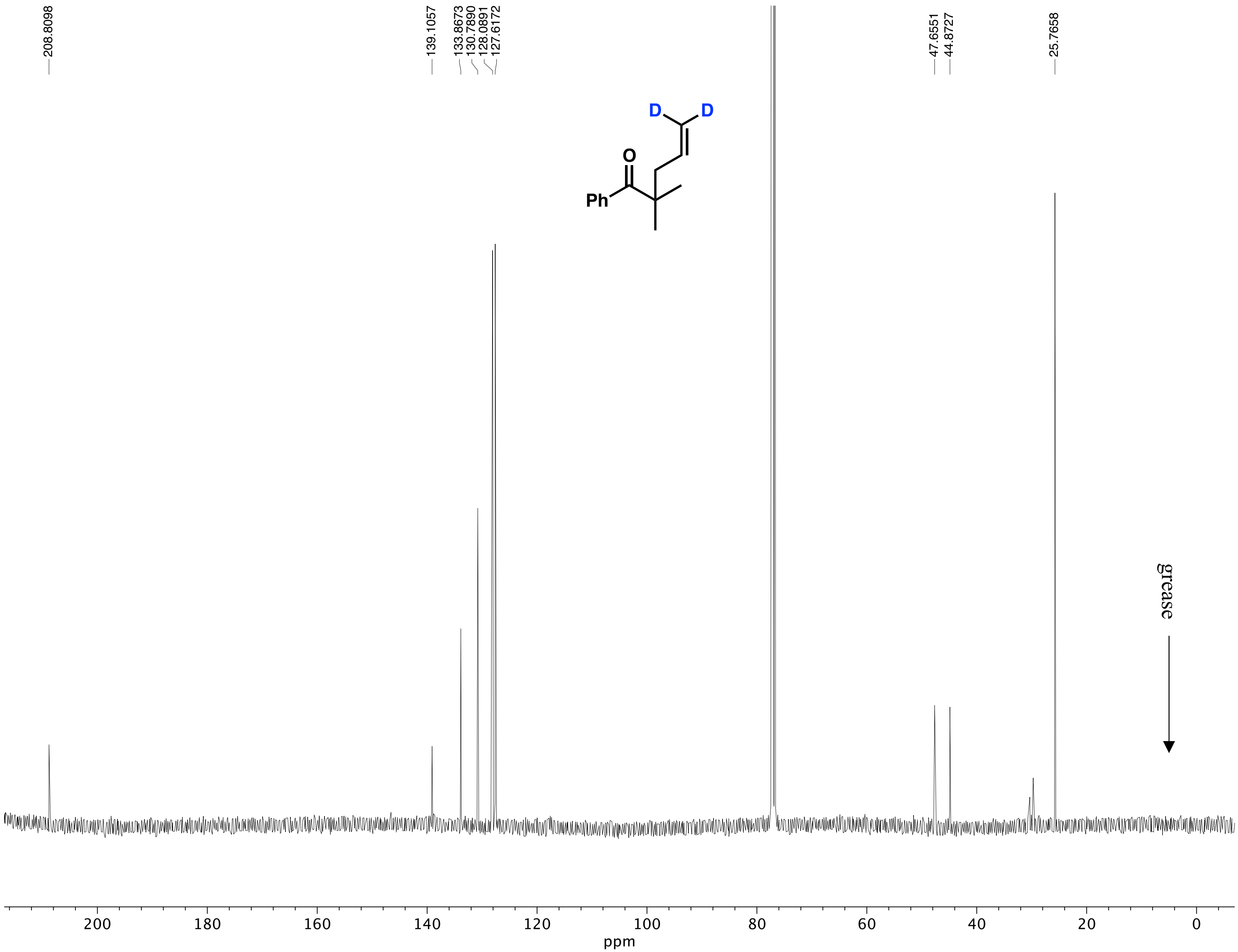

^{13}C NMR (101 MHz, $CDCl_3$) of compound **2a-D₂**.

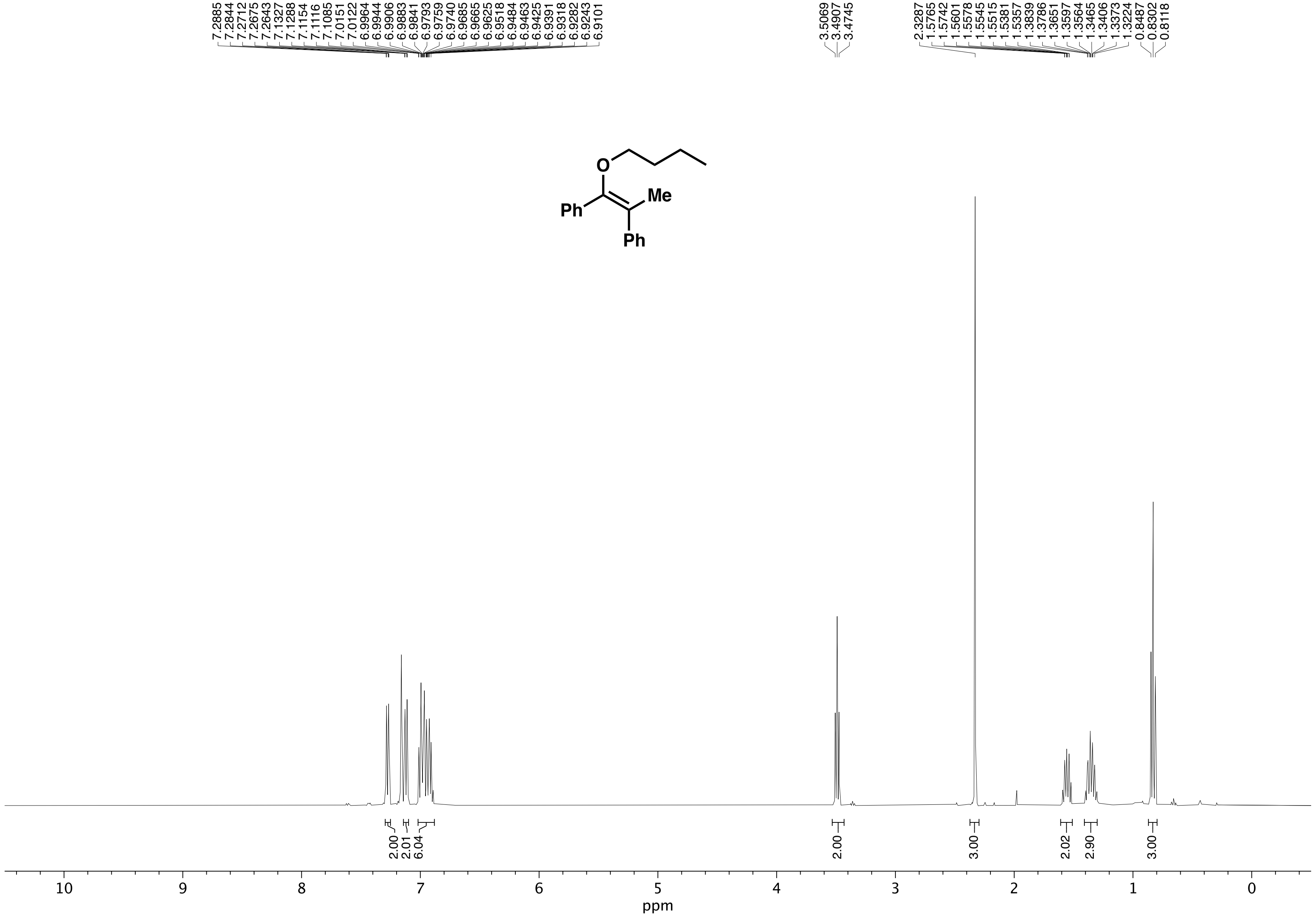

^{1}H NMR (400 MHz, C_6D_6) of **8a**.

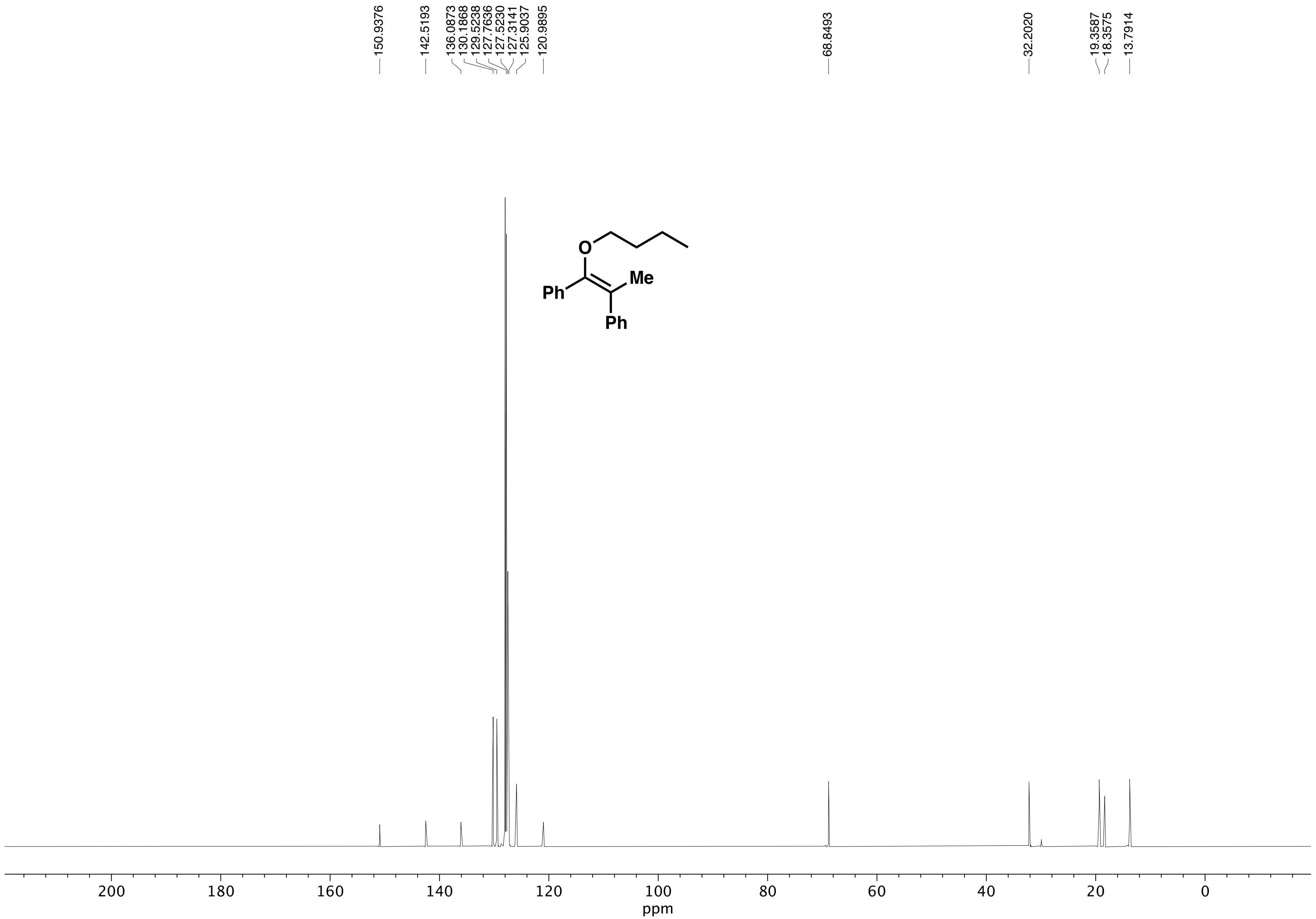

^{13}C NMR (101 MHz, C_6D_6) of **8a**.

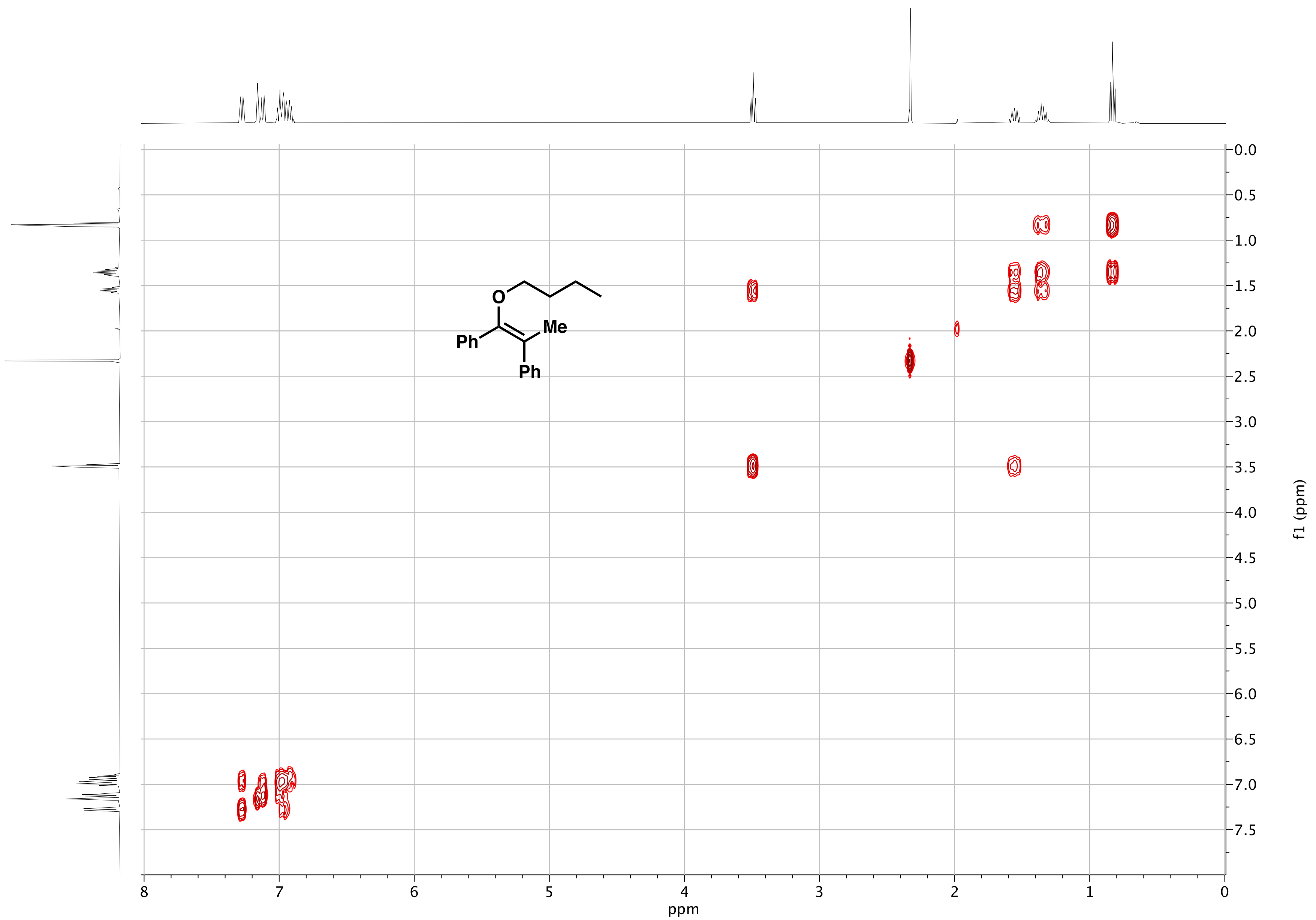

COSY NMR (400 MHz, C_6D_6) of **8a**.

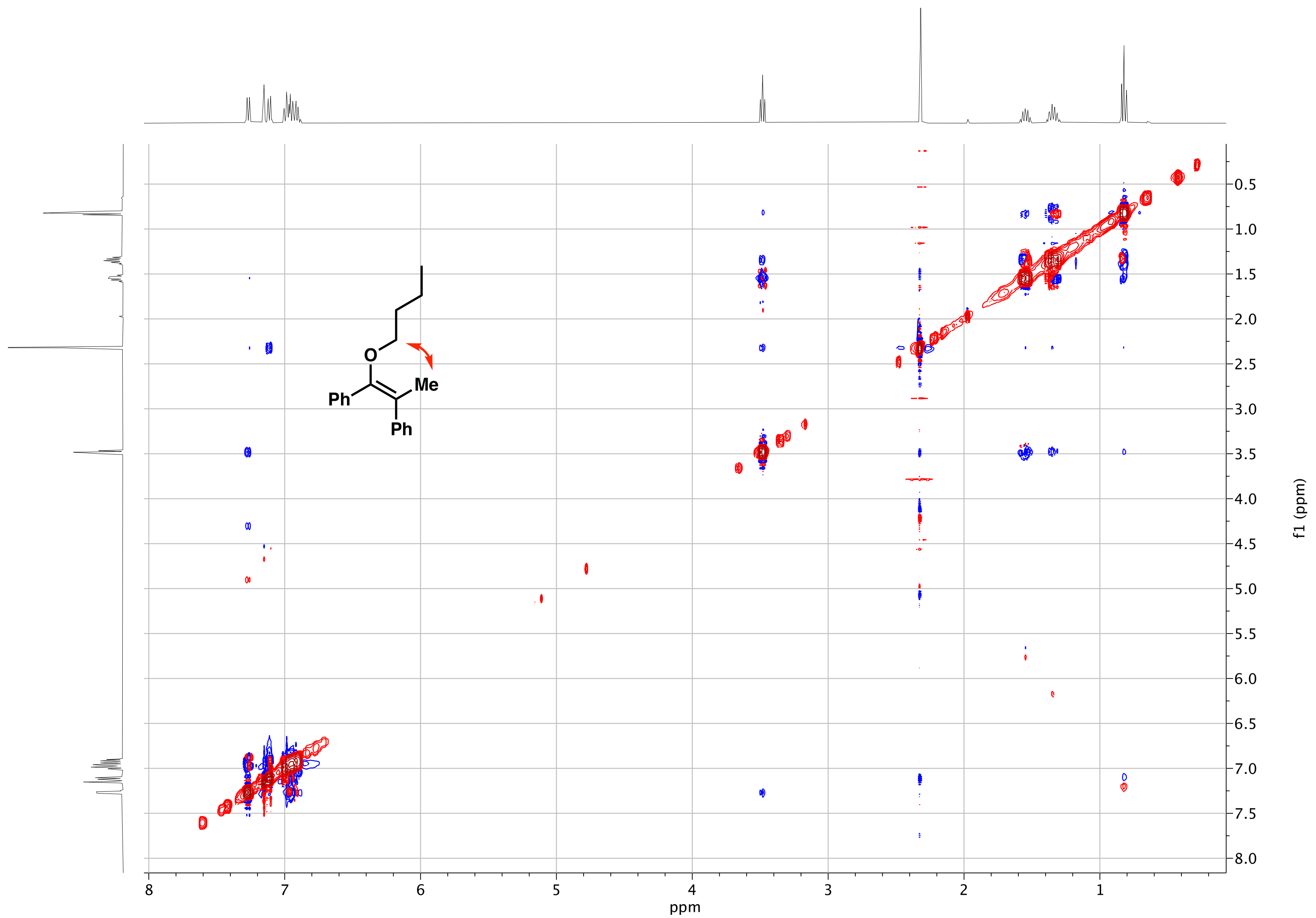

NOESY NMR (400 MHz, C_6D_6) of **8a**.

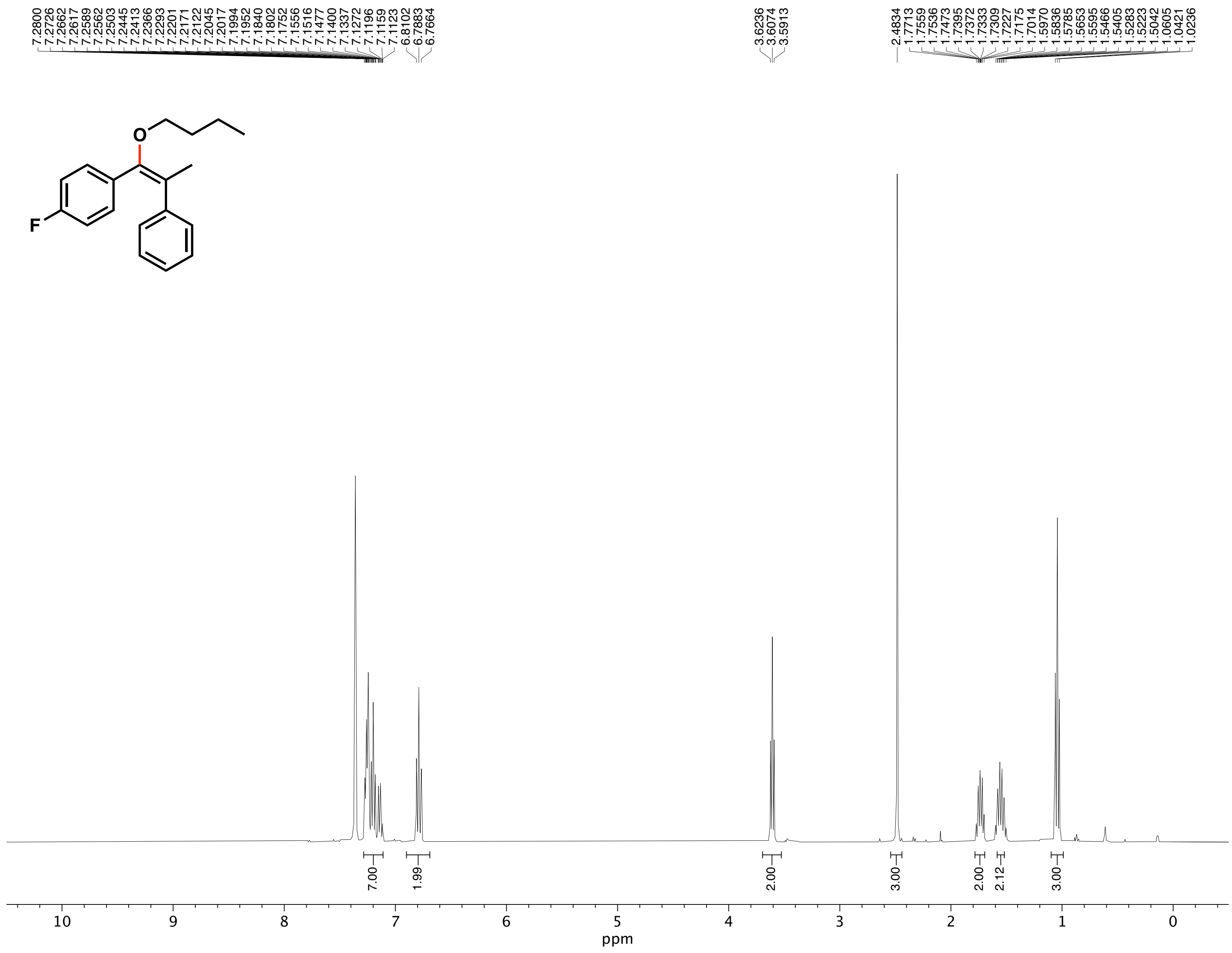

^{1}H NMR (400 MHz, C_6D_6) of compound **8b**.

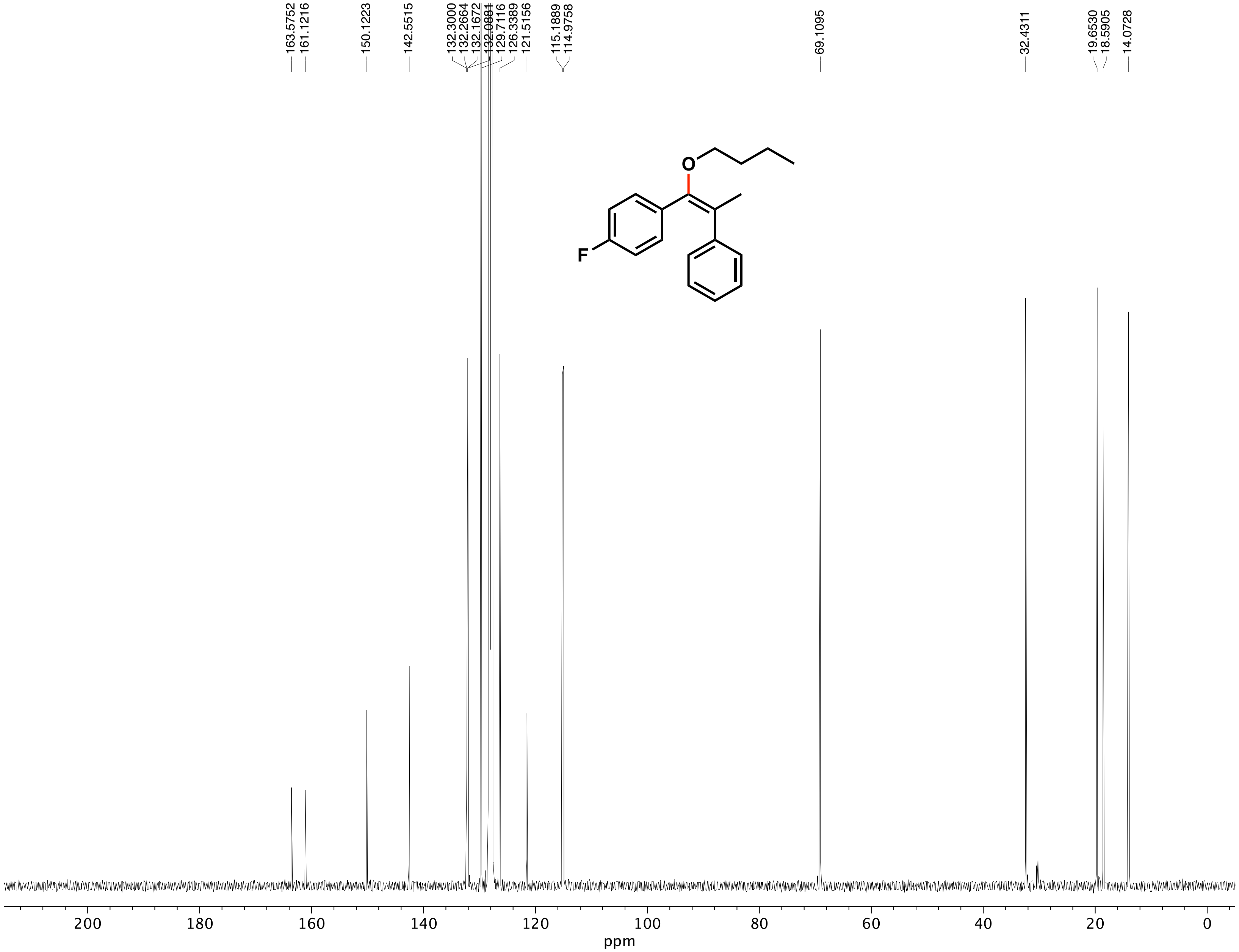

^{13}C NMR (101 MHz, C_6D_6) of compound **8b**.

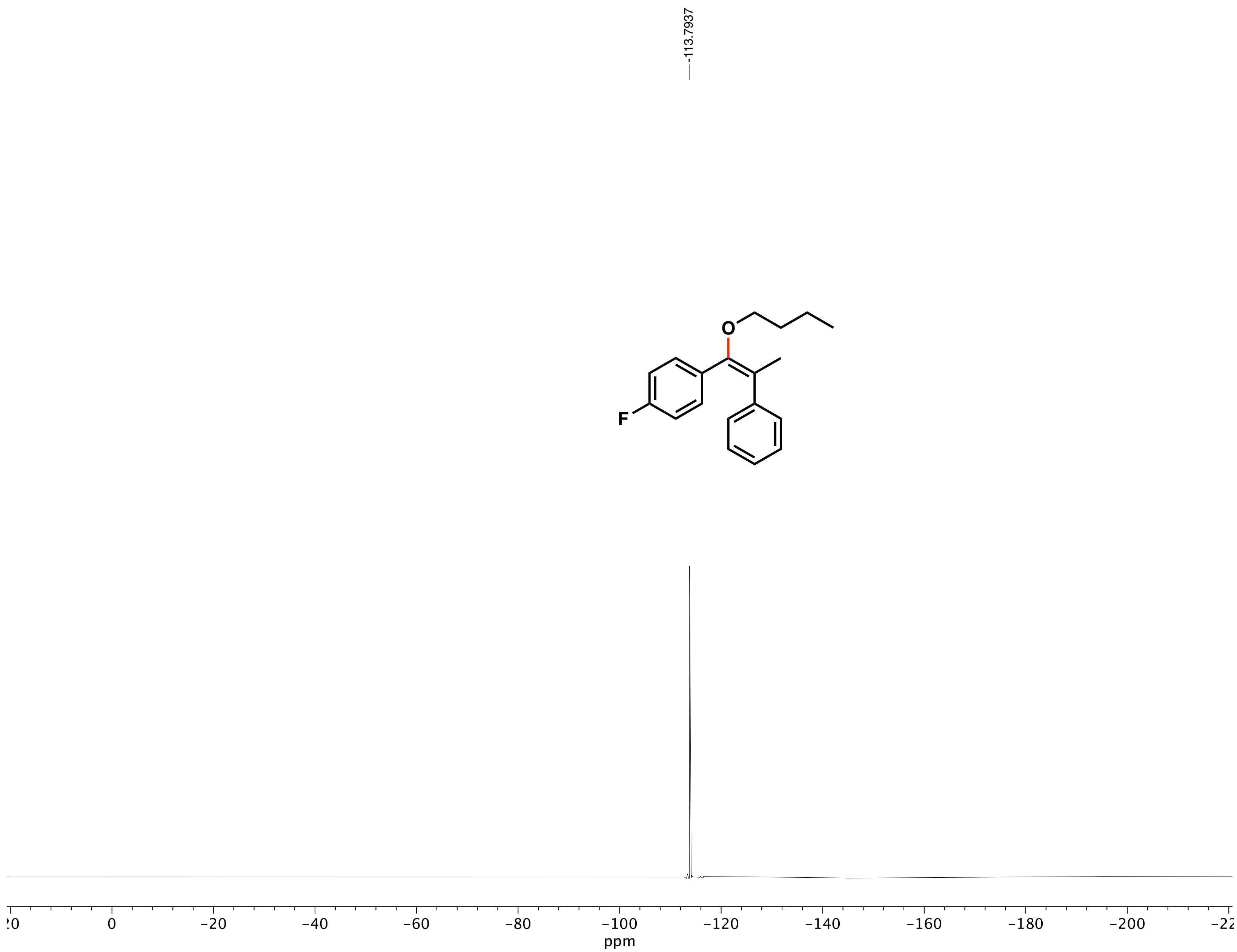

^{19}F NMR (282 MHz, C_6D_6) of compound **8b**.

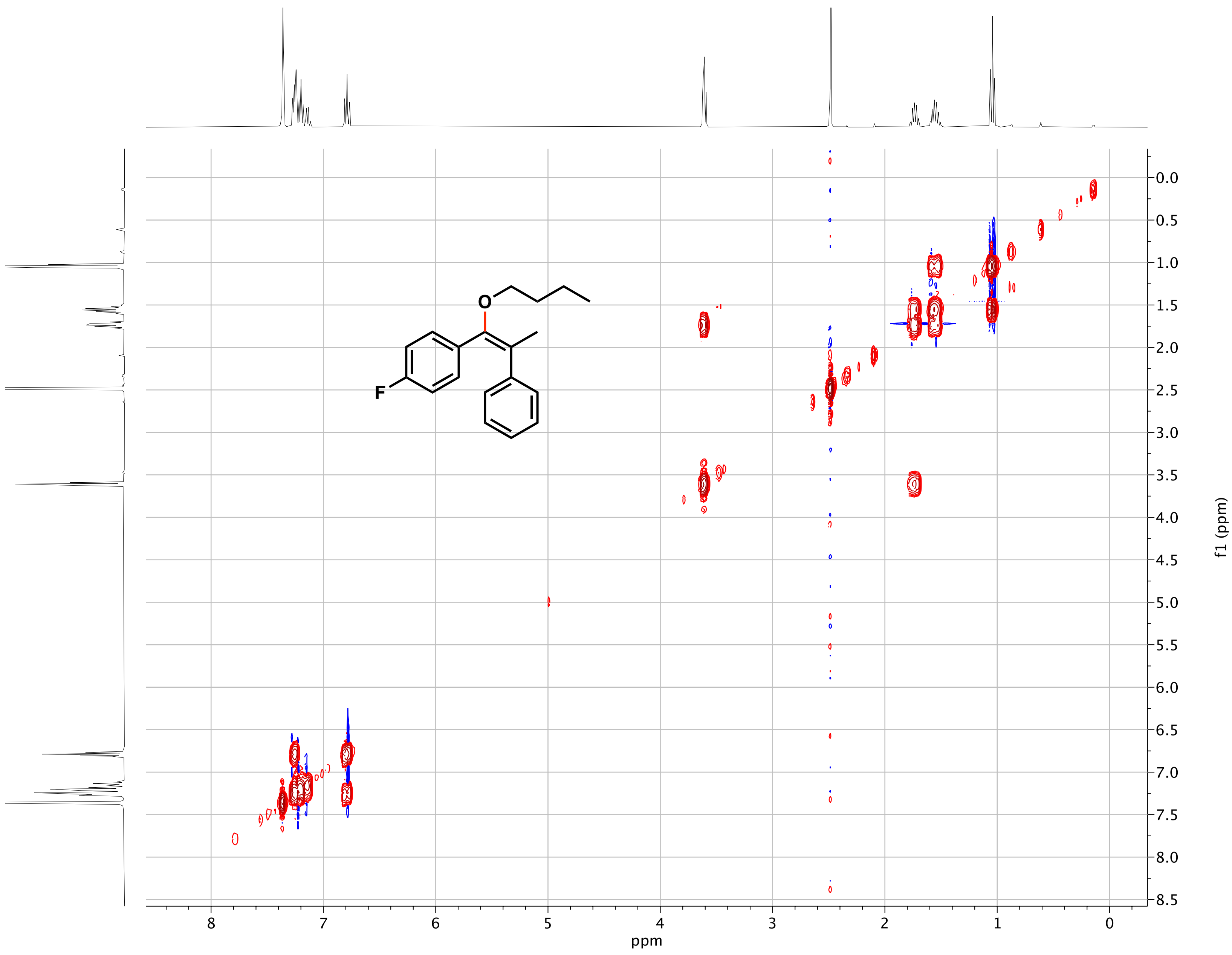

COSY (400 MHz, C_6D_6) of compound **8b**.

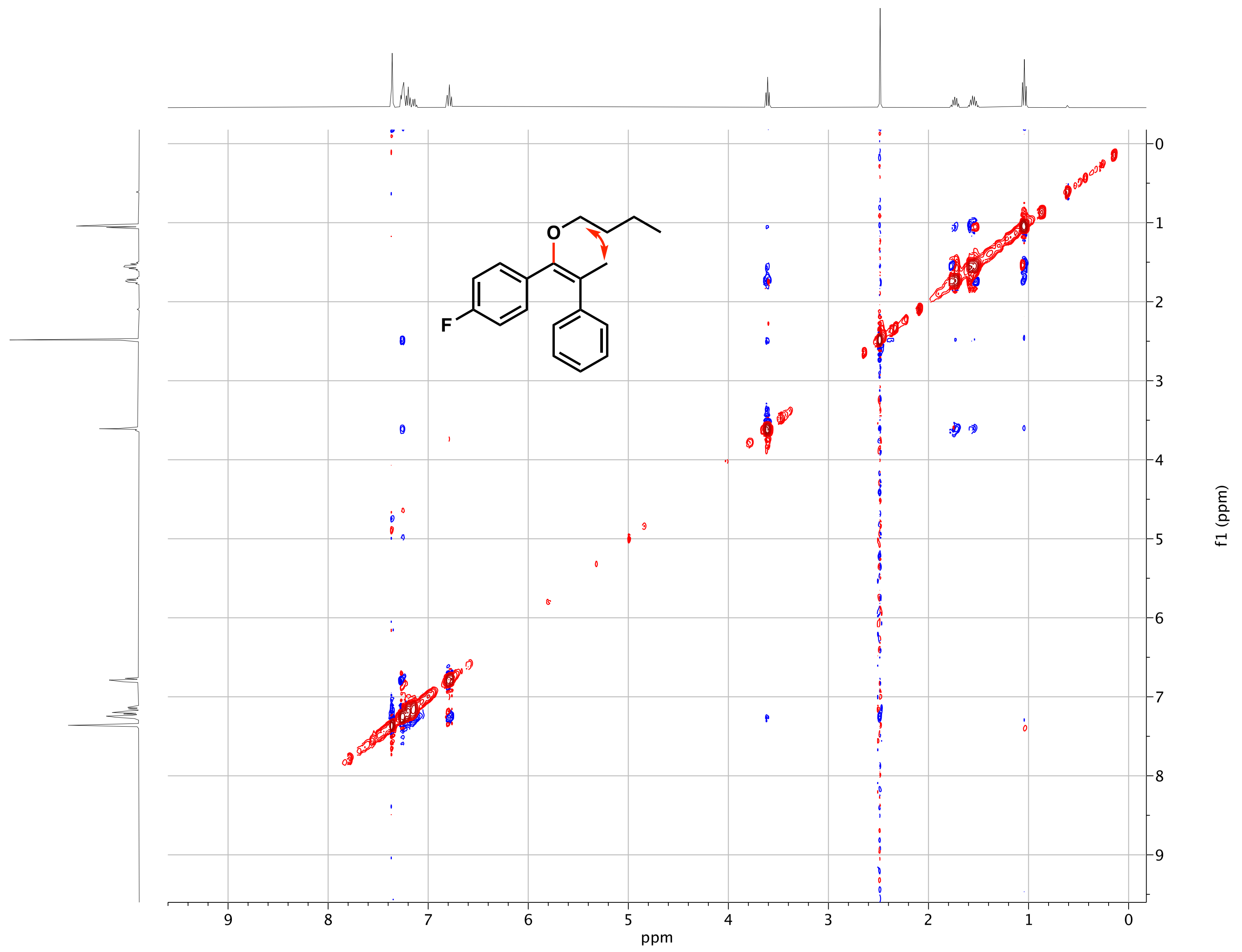

NOESY (400 MHz, C_6D_6) of compound **8b**.

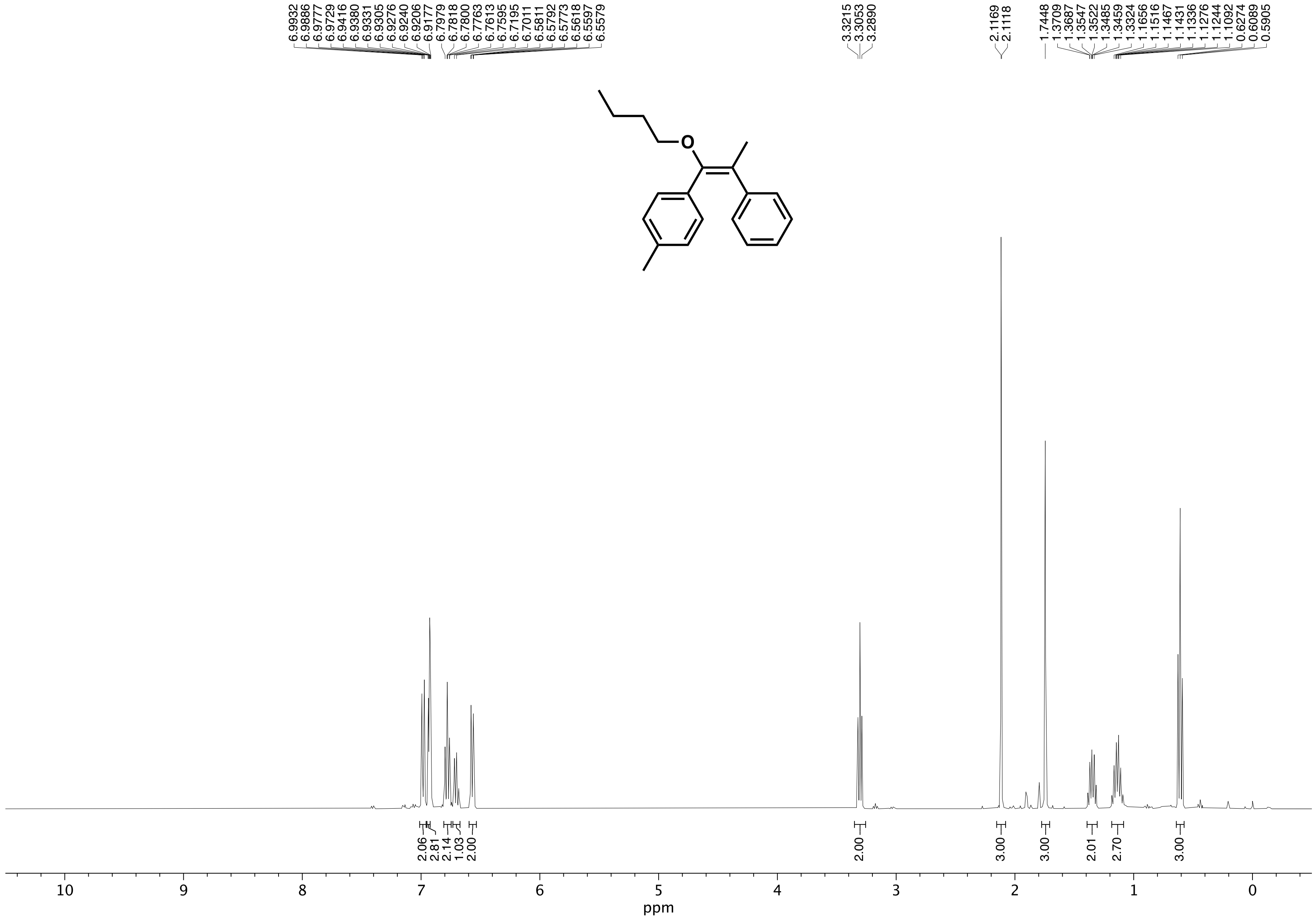

^{1}H NMR (400 MHz, C_6D_6) of **8c**.

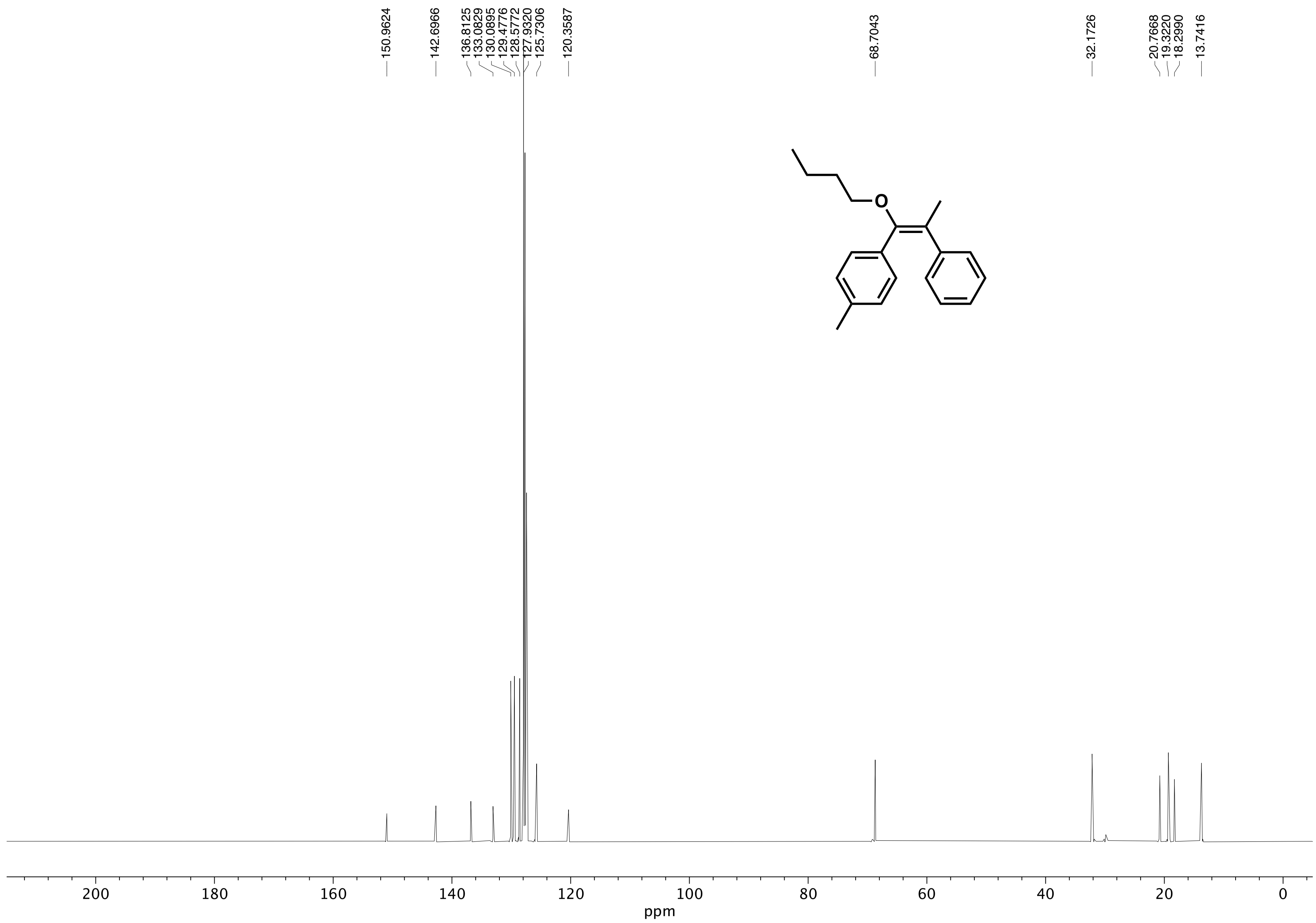

^{13}C NMR (101 MHz, C_6D_6) of **8c**.

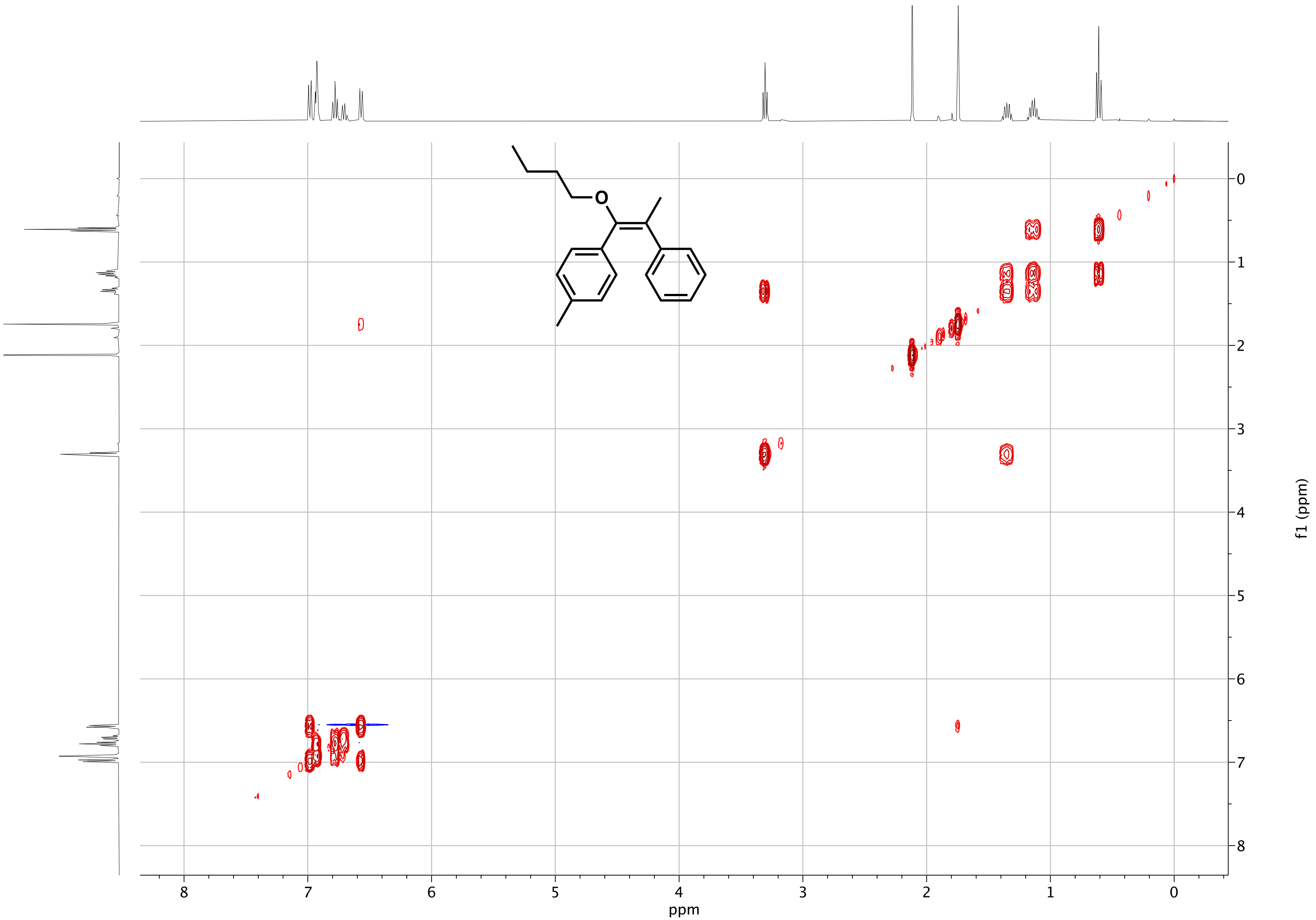

COSY NMR (400 MHz, C_6D_6) of **8c**.

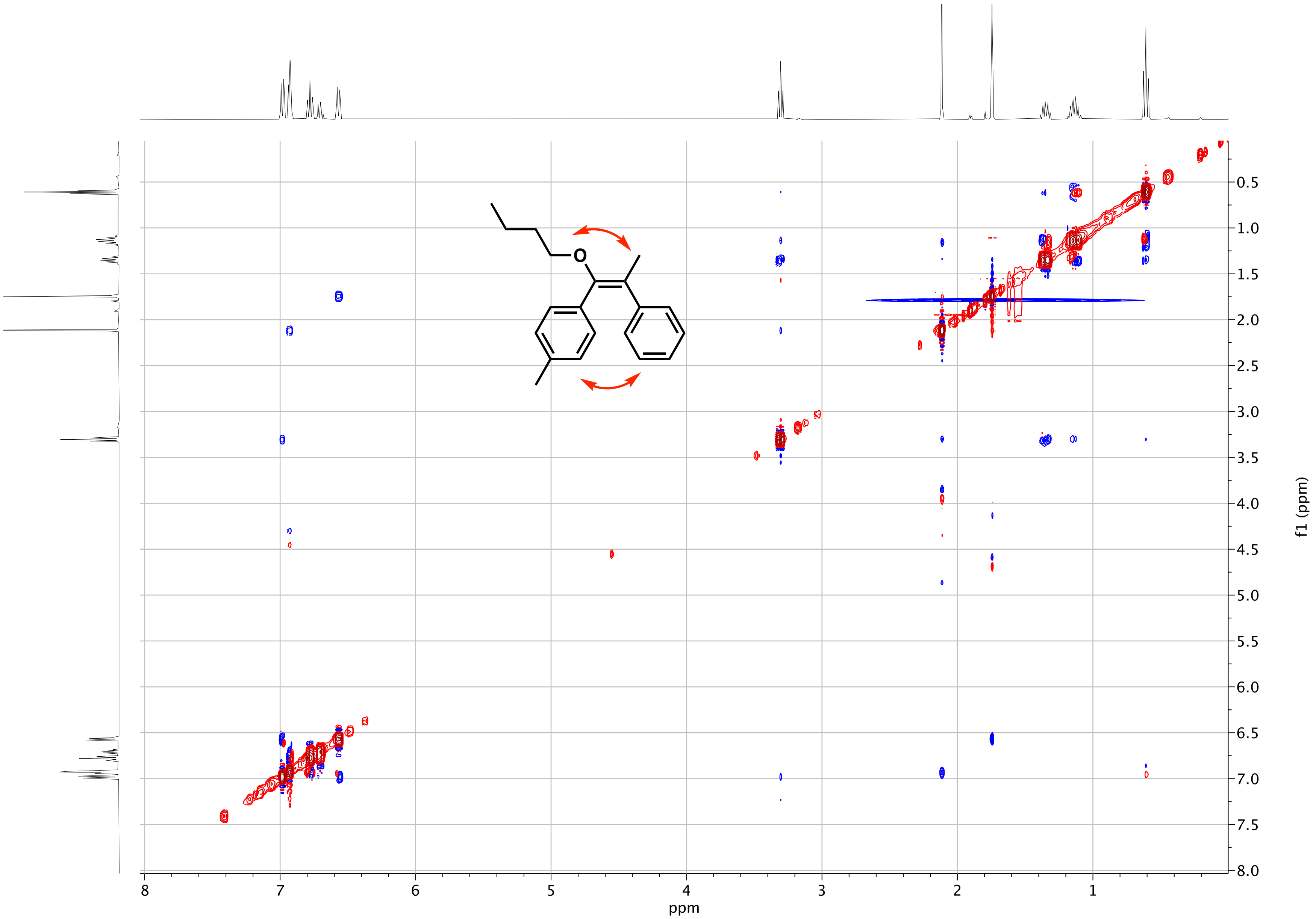

NOESY NMR (400 MHz, C_6D_6) of **8c**.

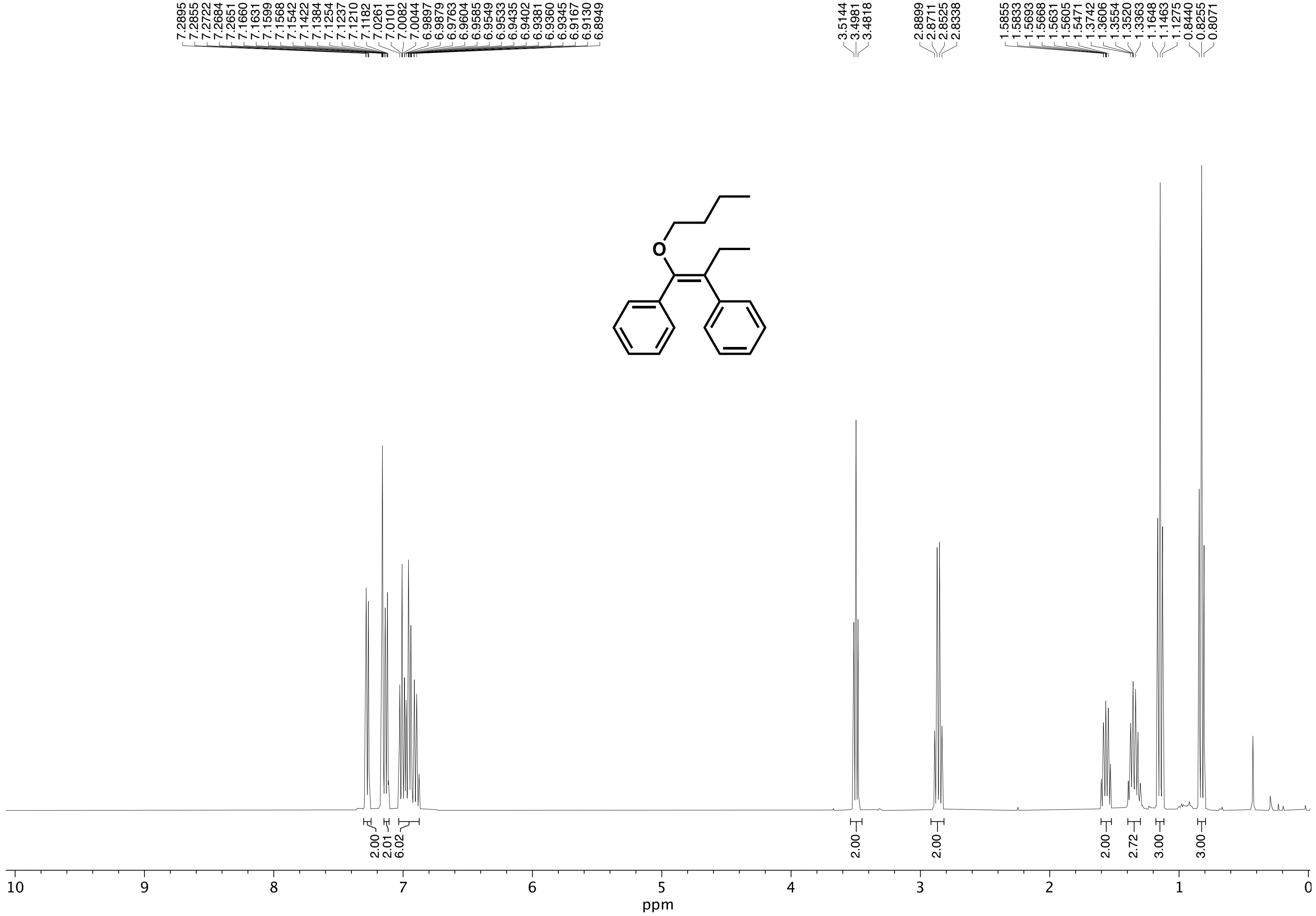

^{1}H NMR (400 MHz, C_6D_6) of **8d**.

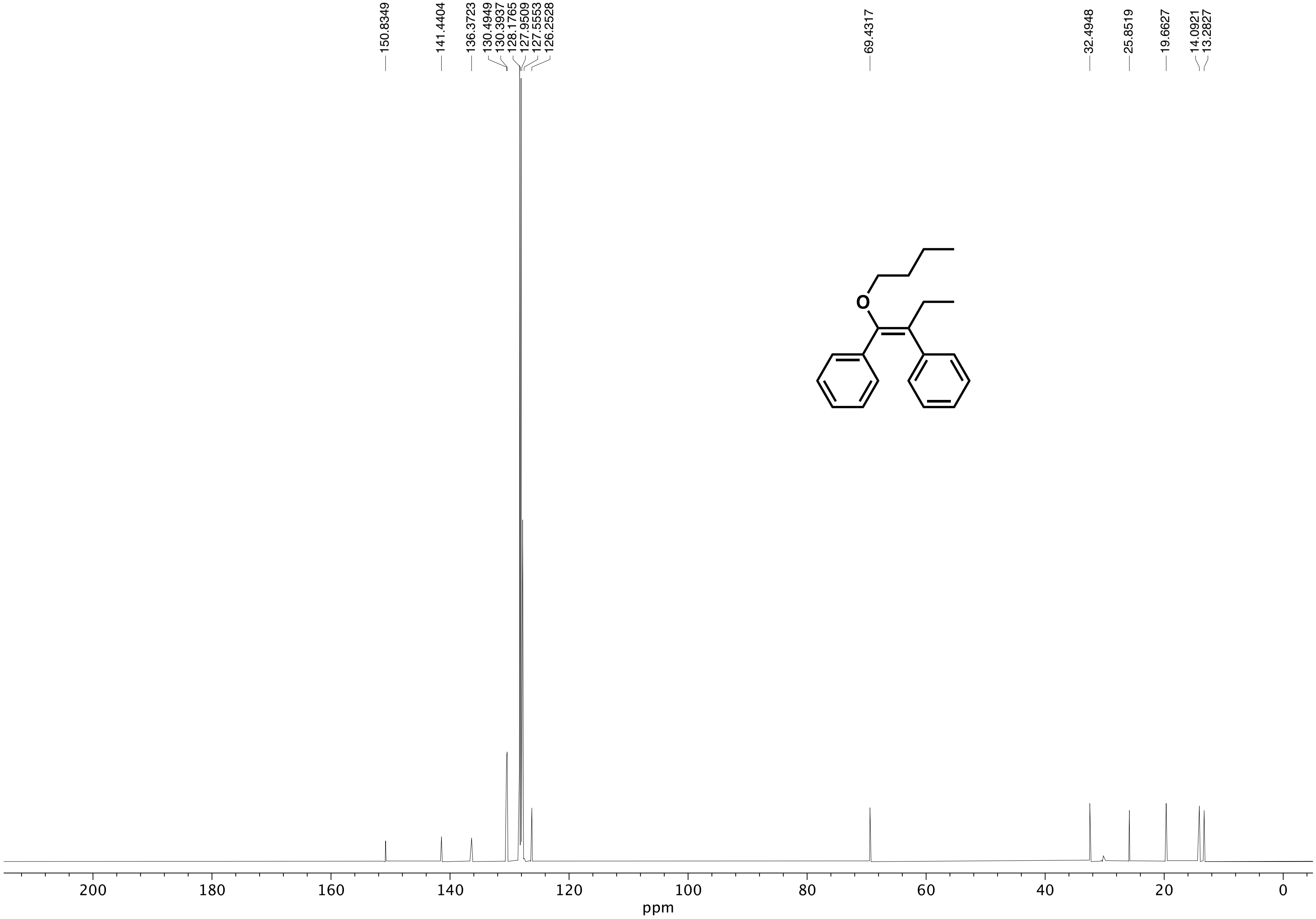

^{13}C NMR (101 MHz, C_6D_6) of **8d**.

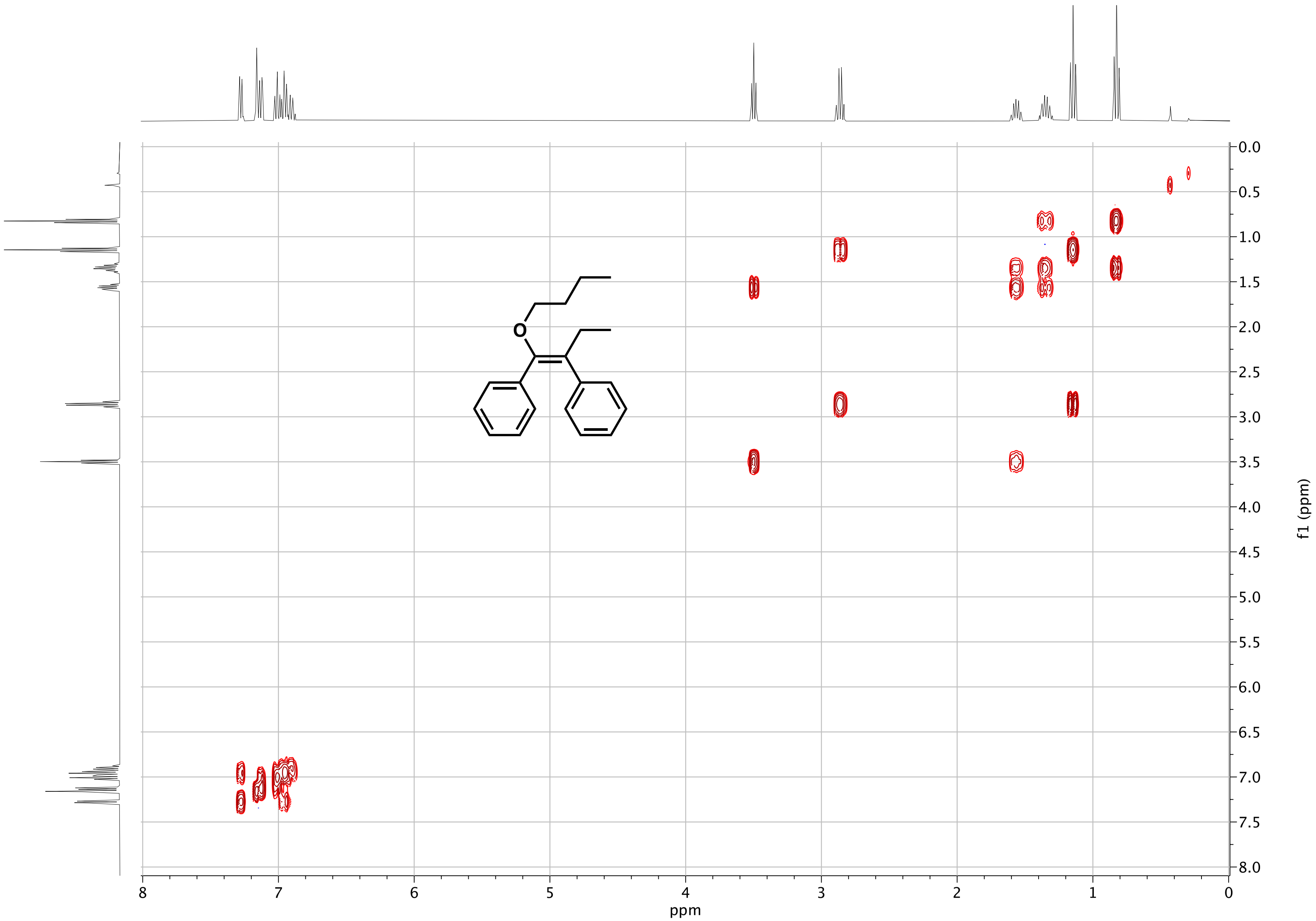

COSY NMR (400 MHz, C_6D_6) of **8d**.

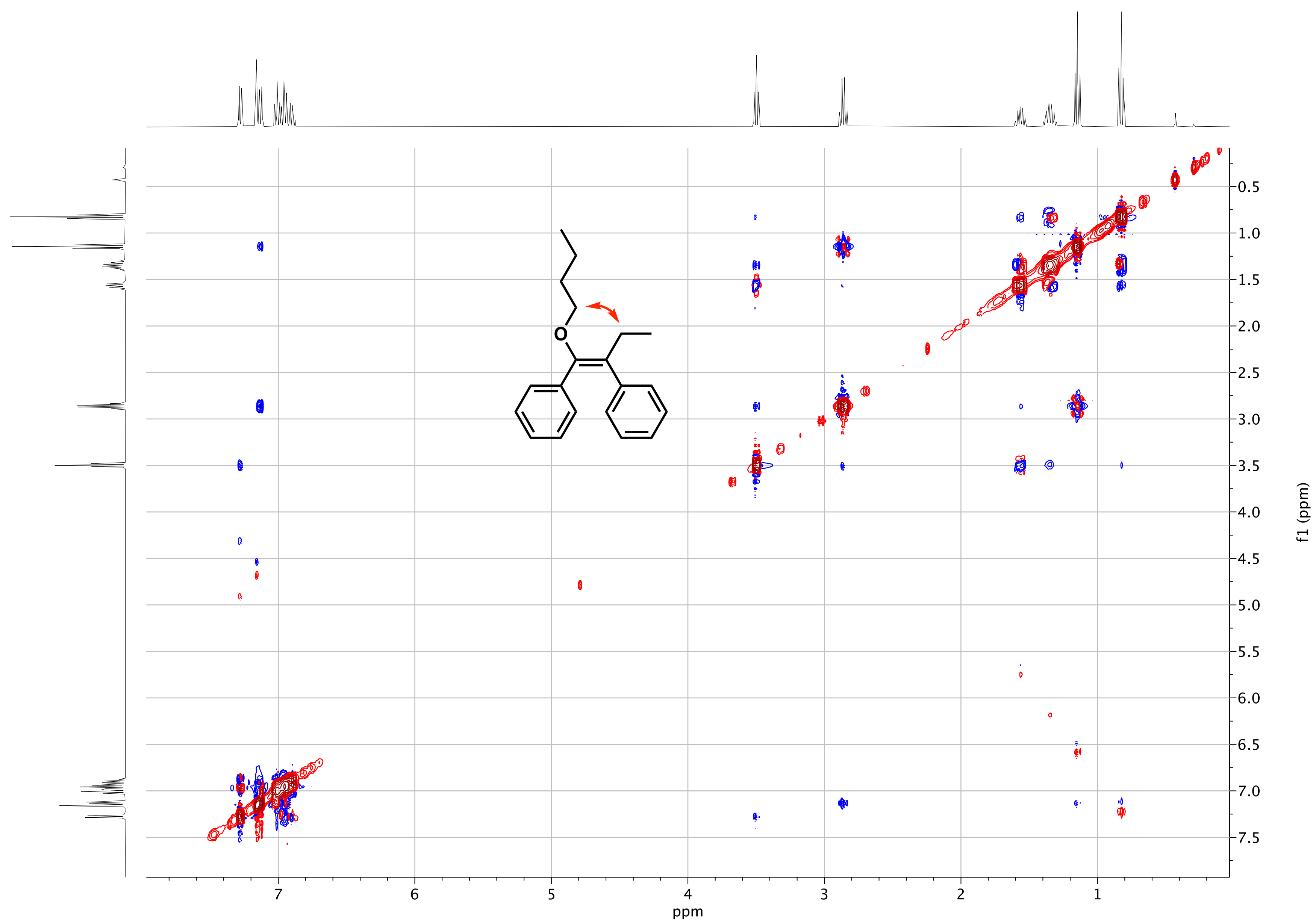

NOESY NMR (400 MHz, C_6D_6) of **8d**.

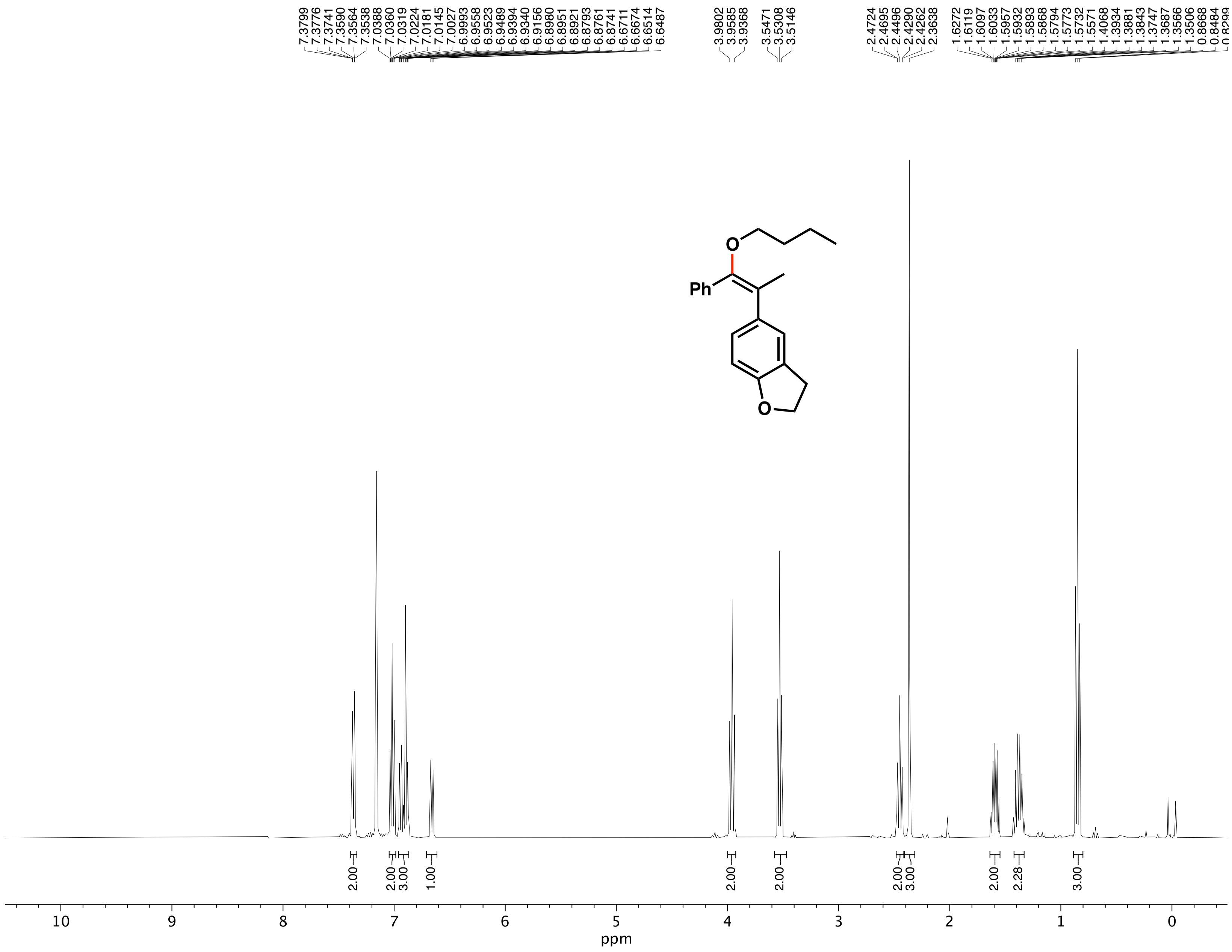

^{1}H NMR (400 MHz, C_6D_6) of compound **8e**.

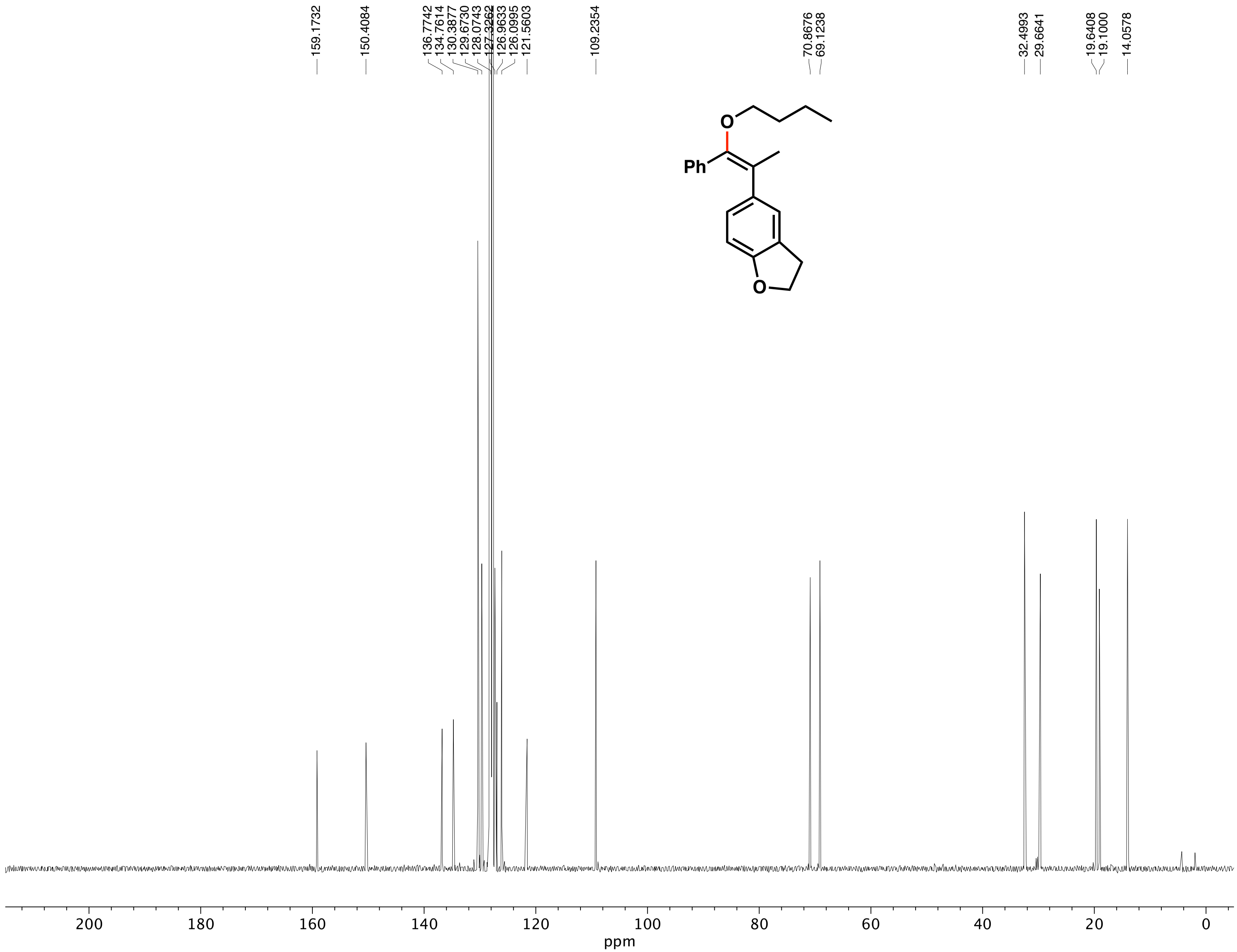

^{13}C NMR (101 MHz, C_6D_6) of compound **8e**.

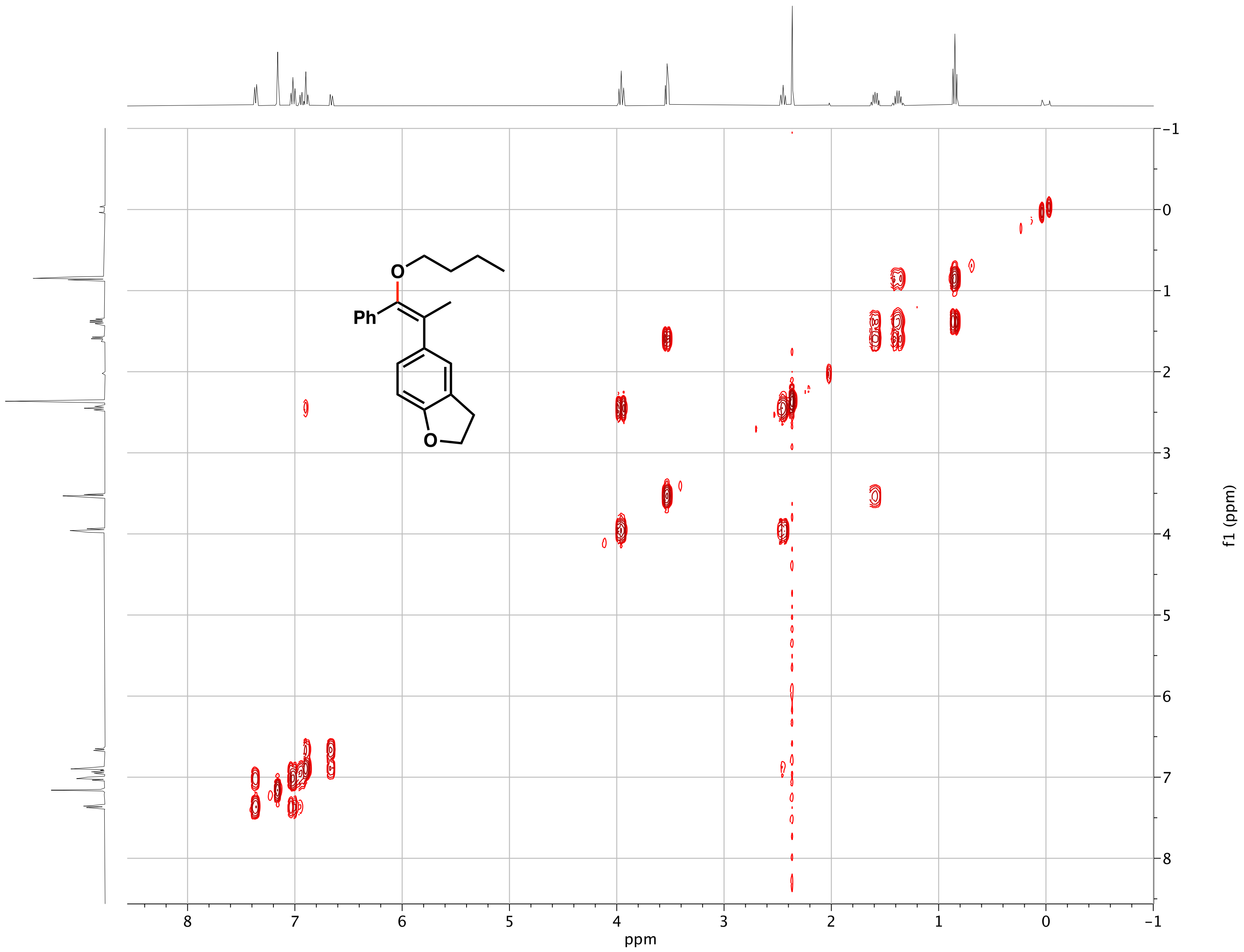

COSY (400 MHz, C_6D_6) of compound **8e**.

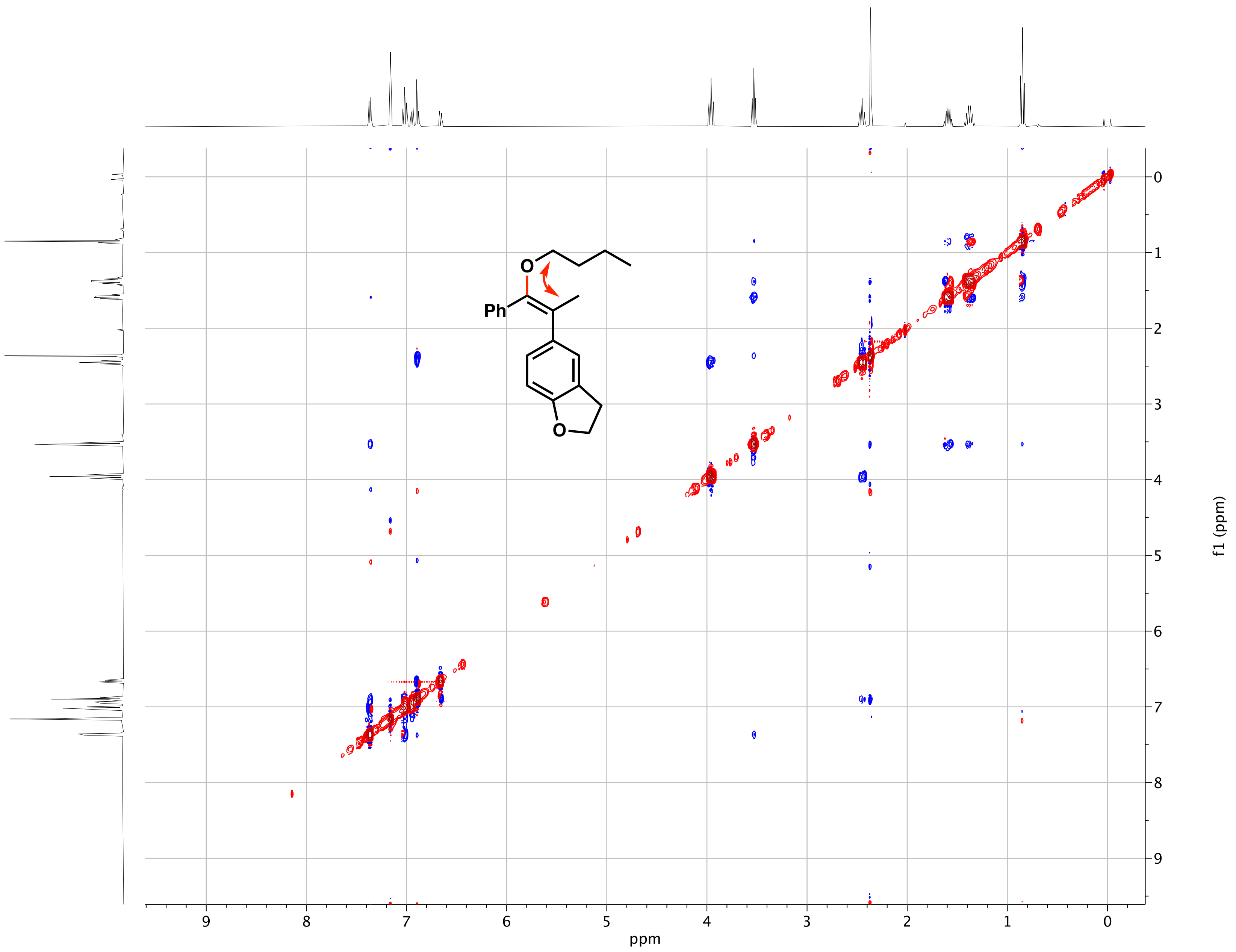

NOESY (400 MHz, C_6D_6) of compound **8e**.

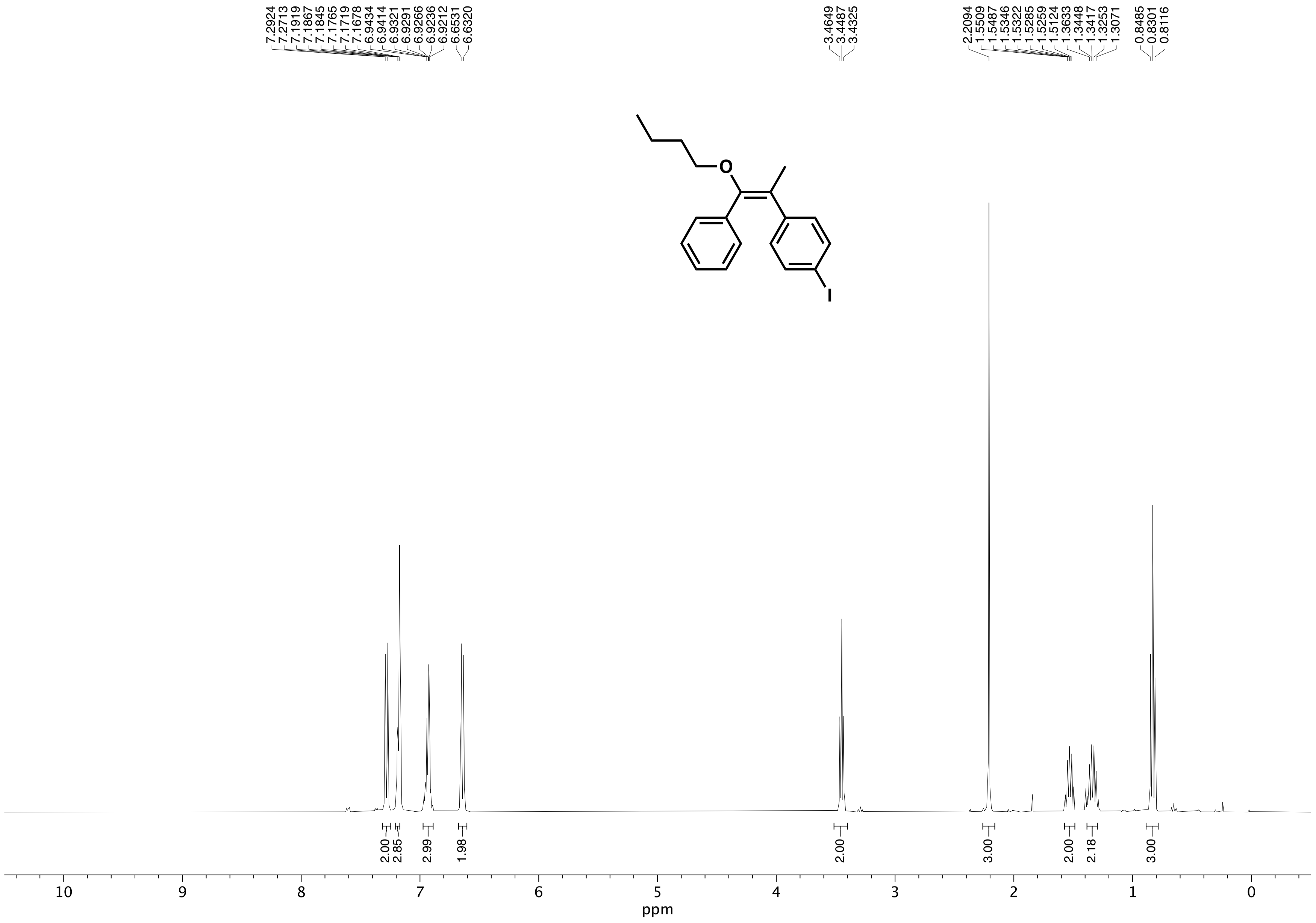

^{1}H NMR (400 MHz, C_6D_6) of **8f**.

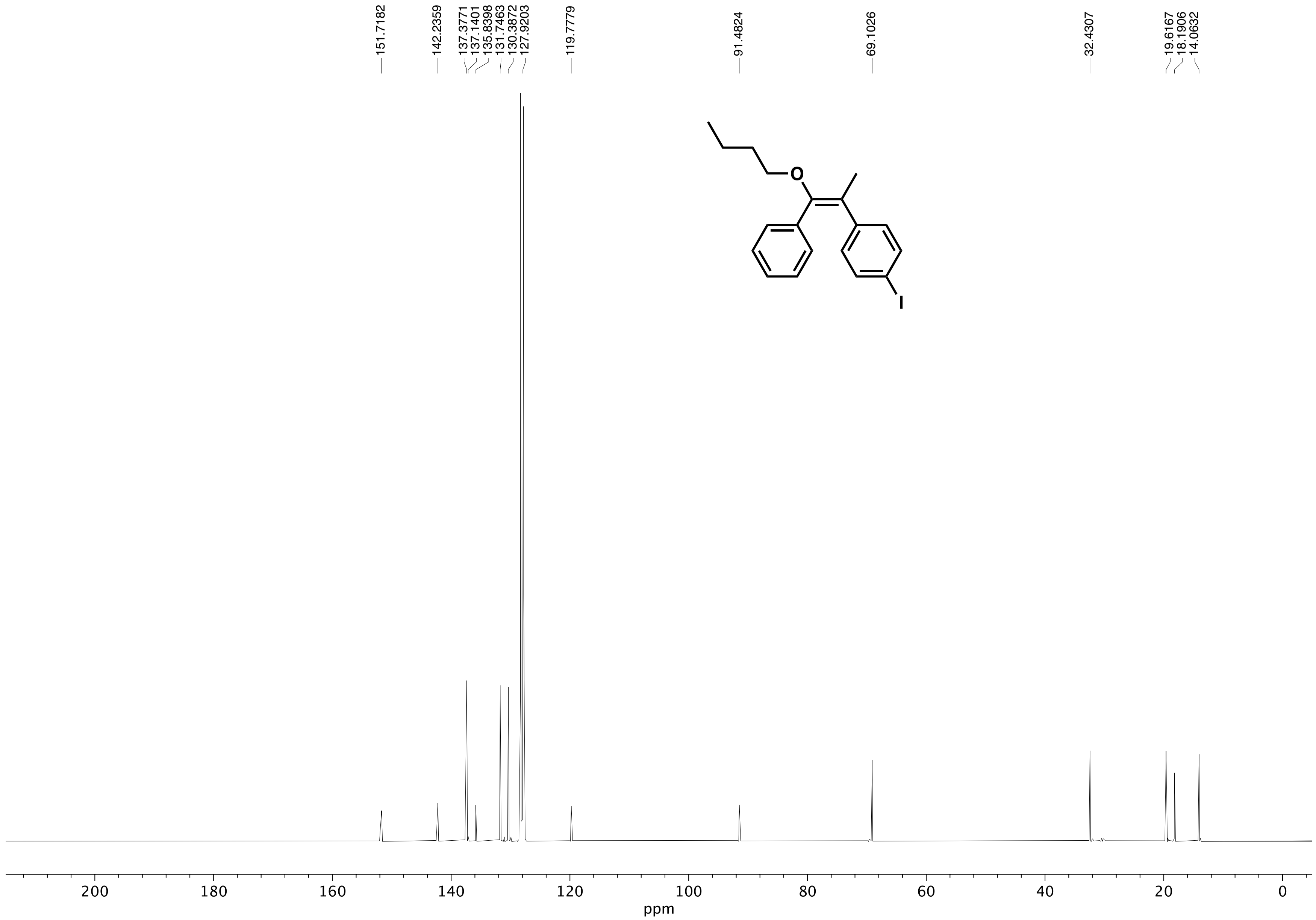

^{13}C NMR (101 MHz, C_6D_6) of **8f**.

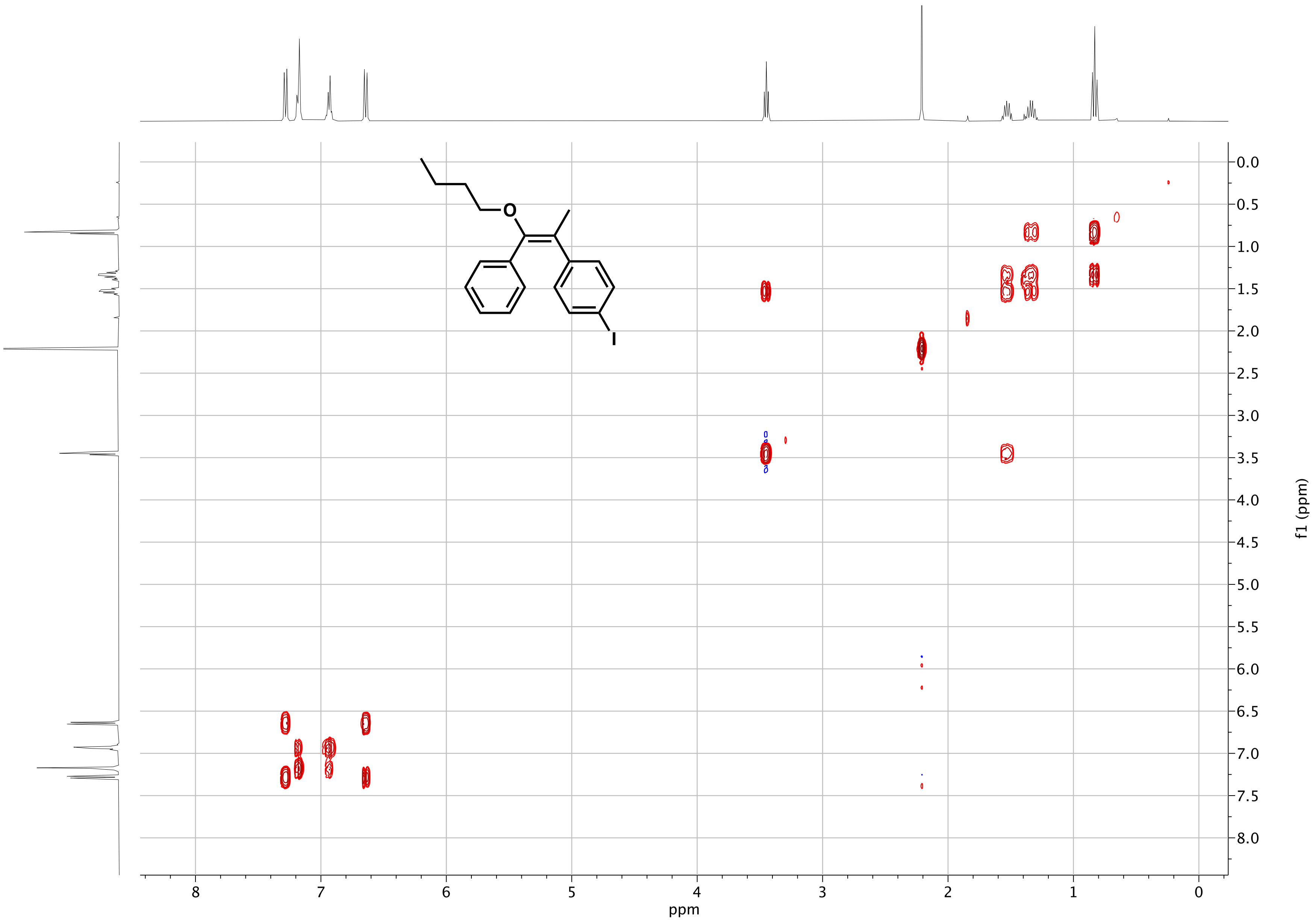

COSY NMR (400 MHz, C_6D_6) of **8f**.

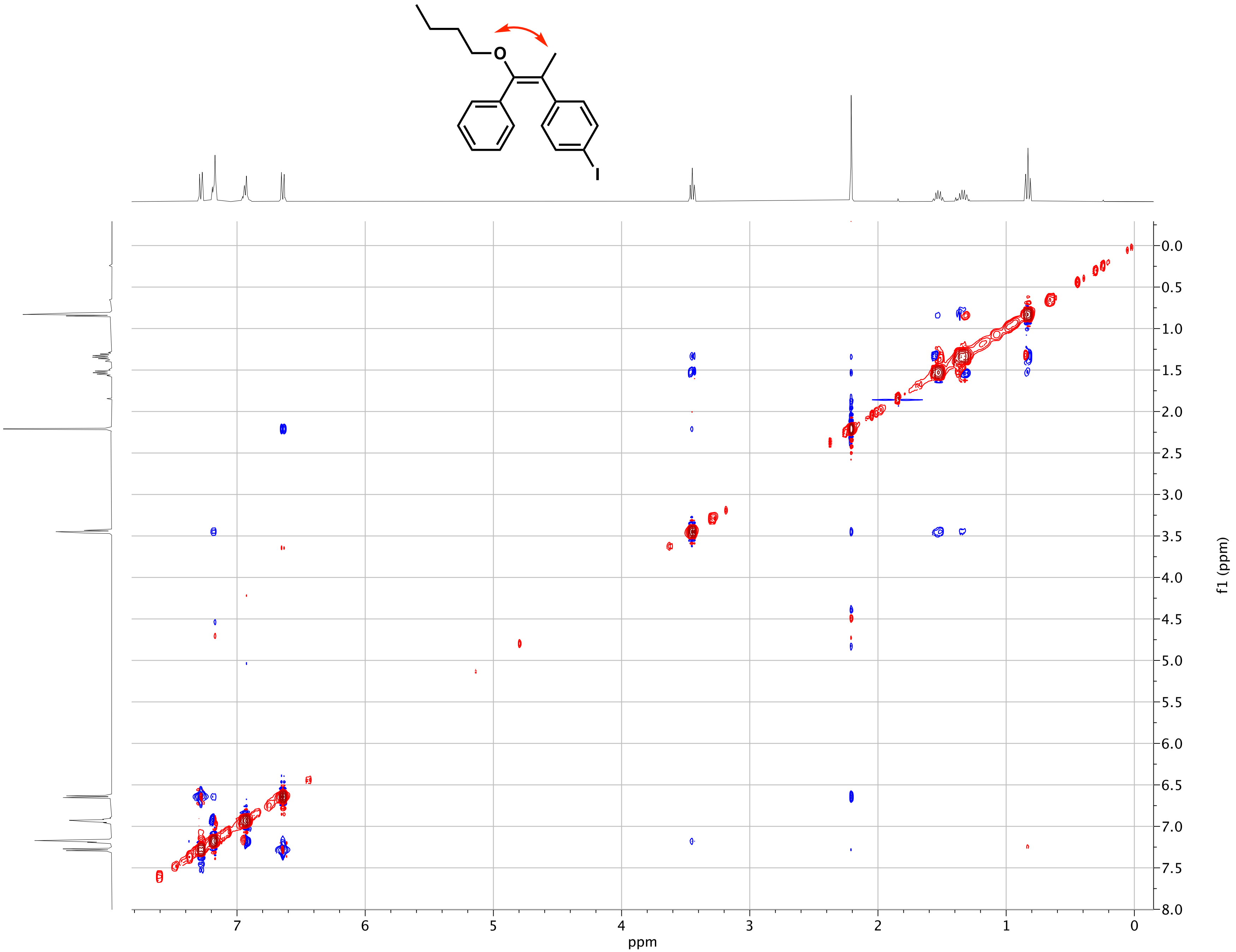

NOESY NMR (400 MHz, C_6D_6) of **8f**.

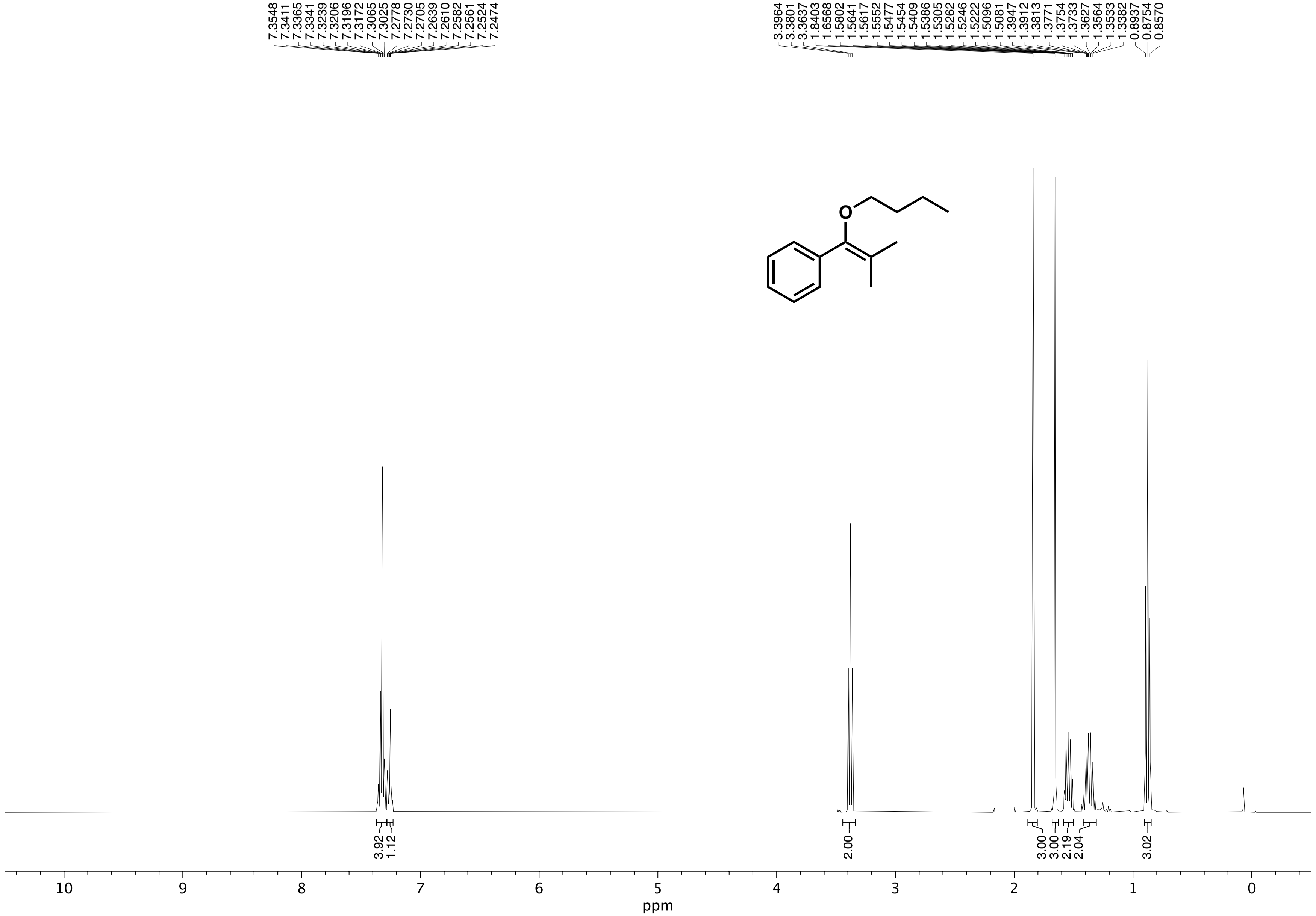

^1H NMR (400 MHz, $CDCl_3$) of **8g**.

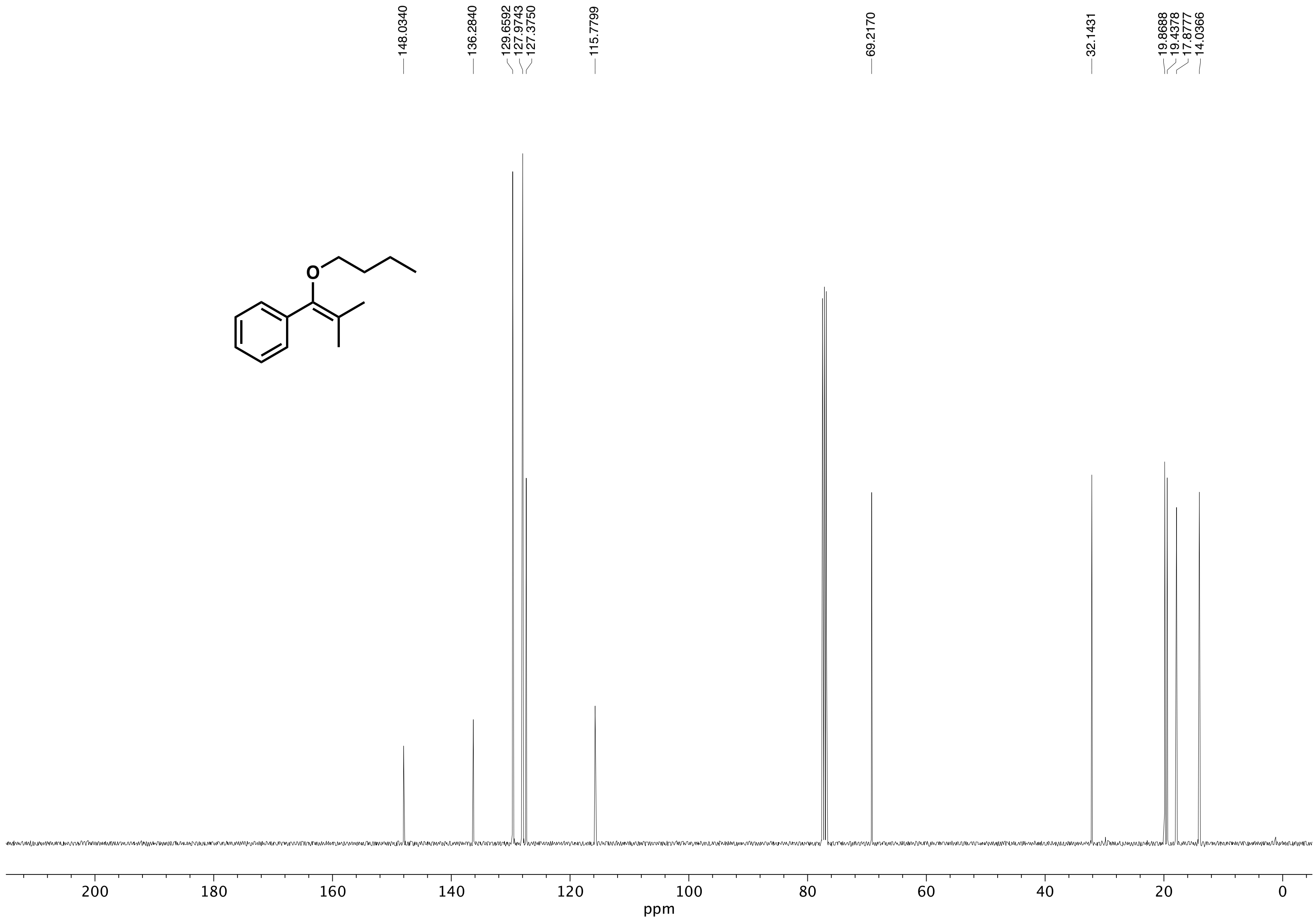

^{13}C NMR (101 MHz, $CDCl_3$) of **8g**.

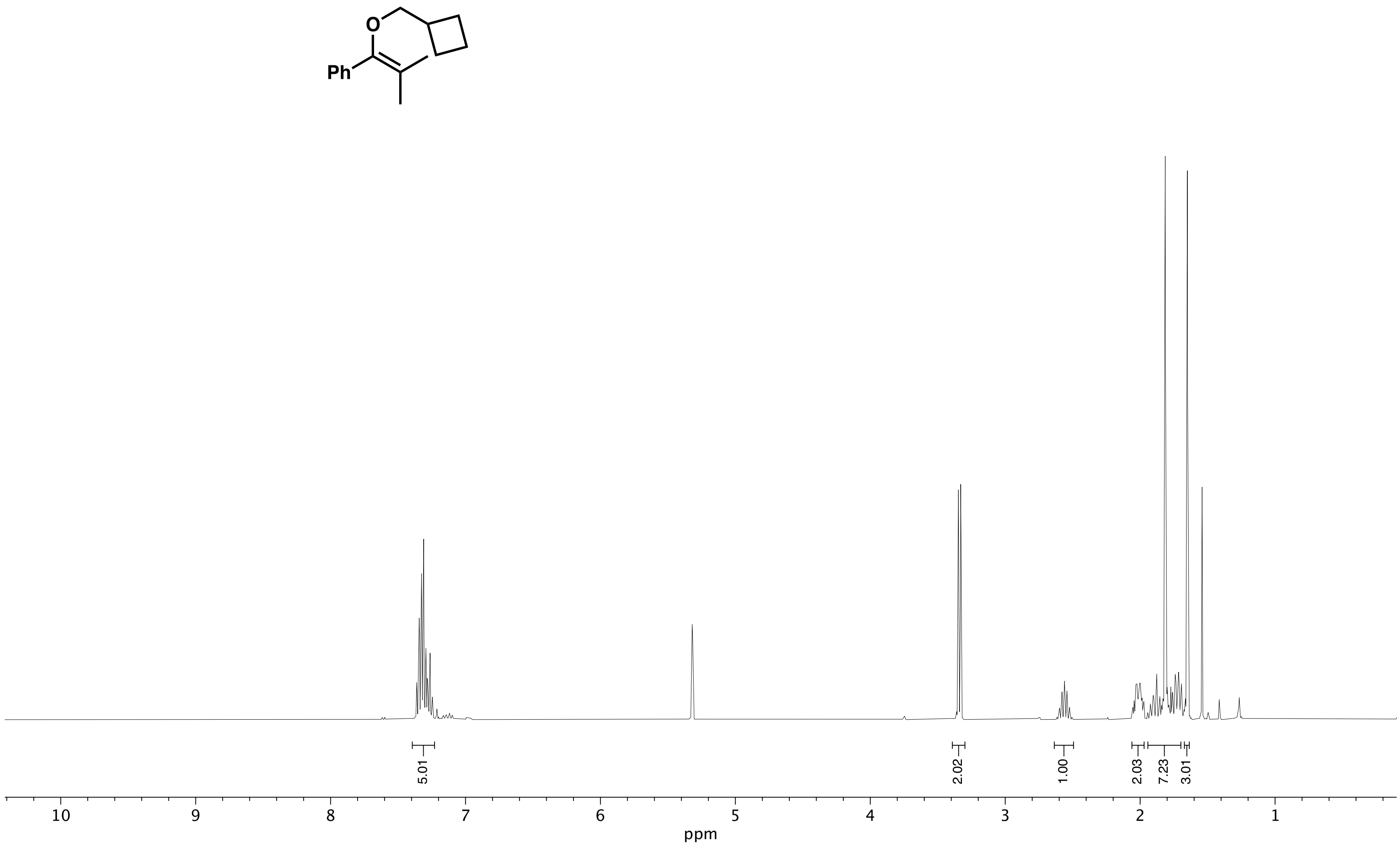

^{1}H NMR (400 MHz, CD_2Cl_2) of **8h**.

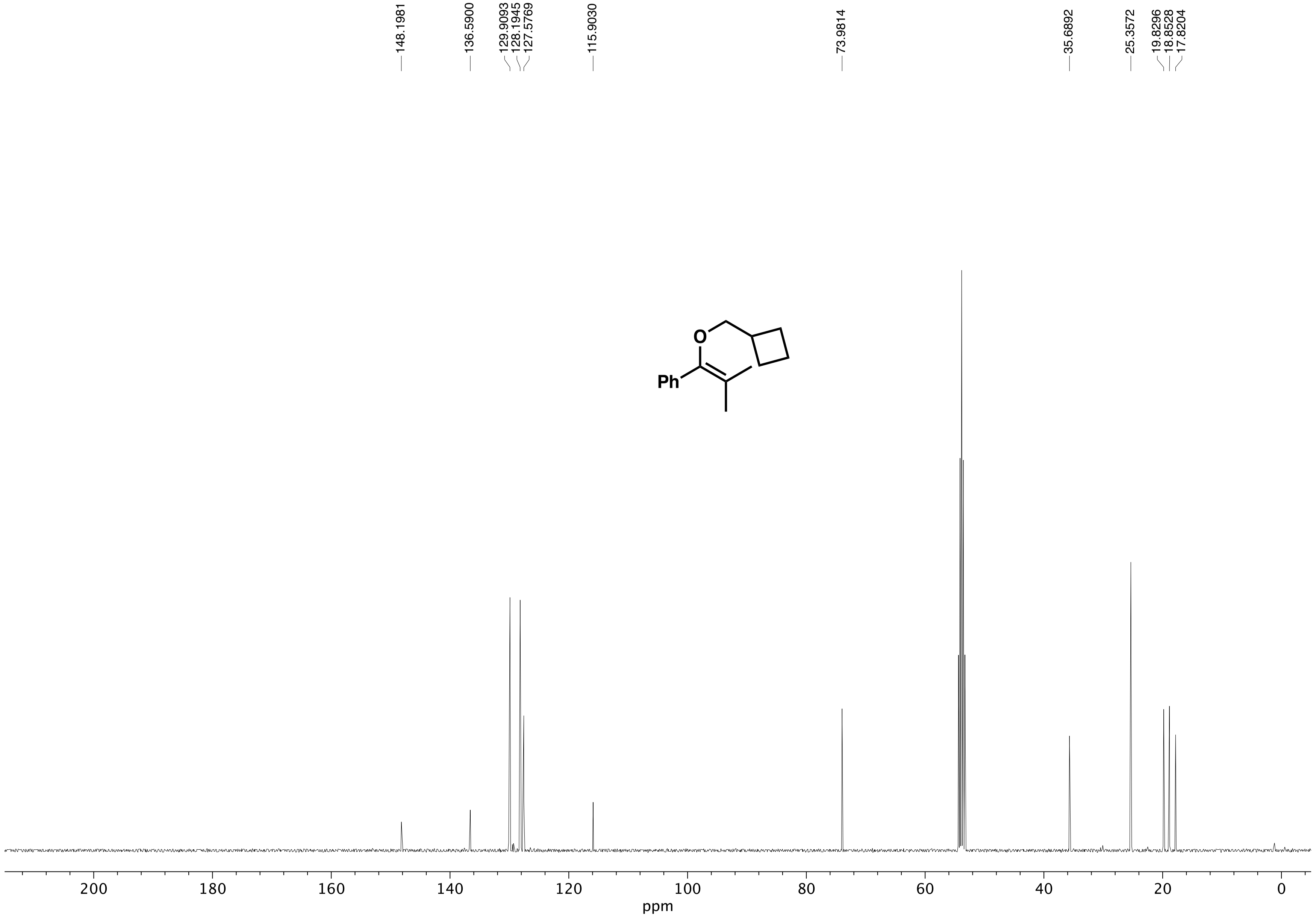

^{13}C NMR (101 MHz, CD_2Cl_2) of **8h**.

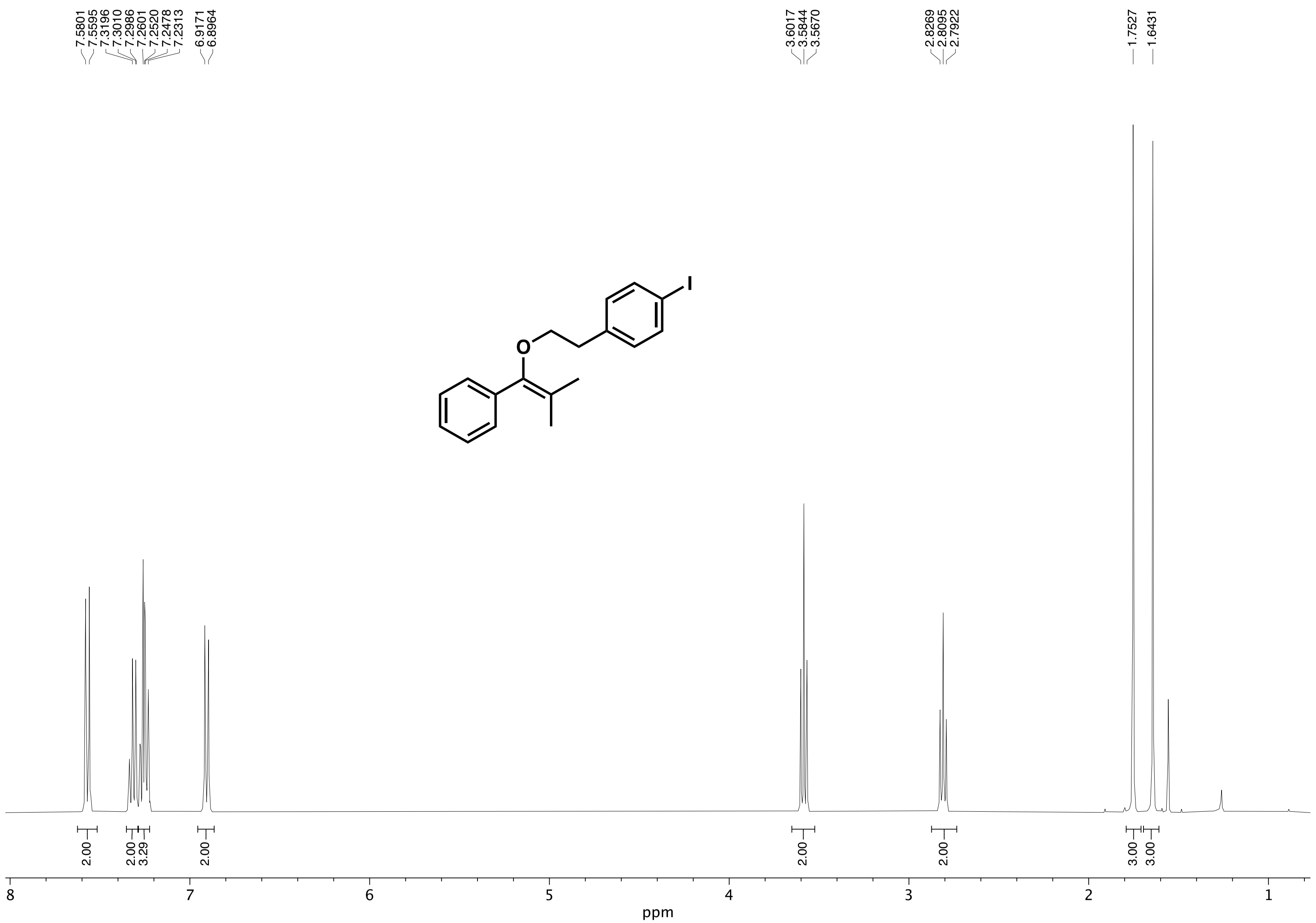

^1H NMR (400 MHz, $CDCl_3$) of **8i.**

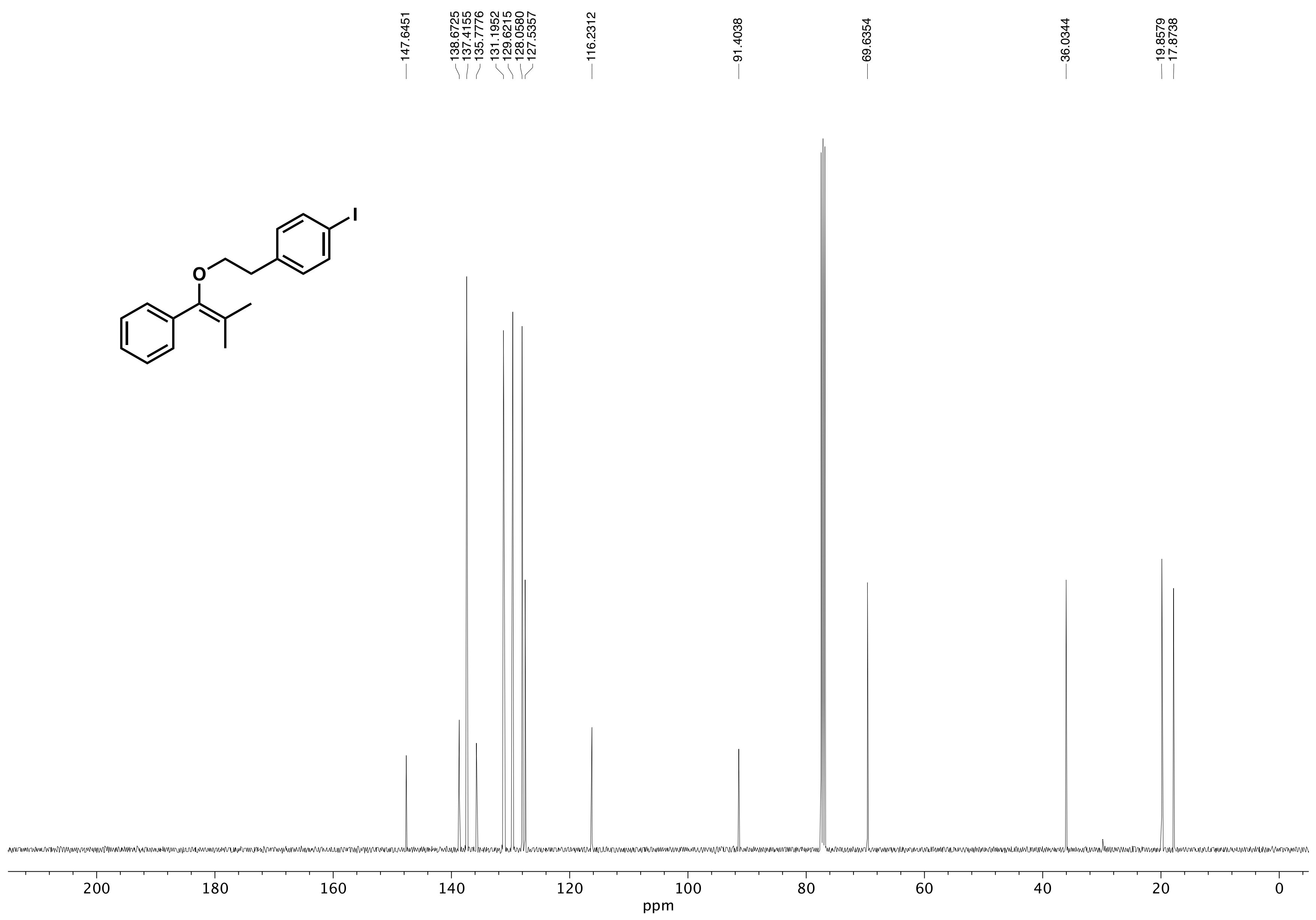

^{13}C NMR (101 MHz, $CDCl_3$) of **8i**.

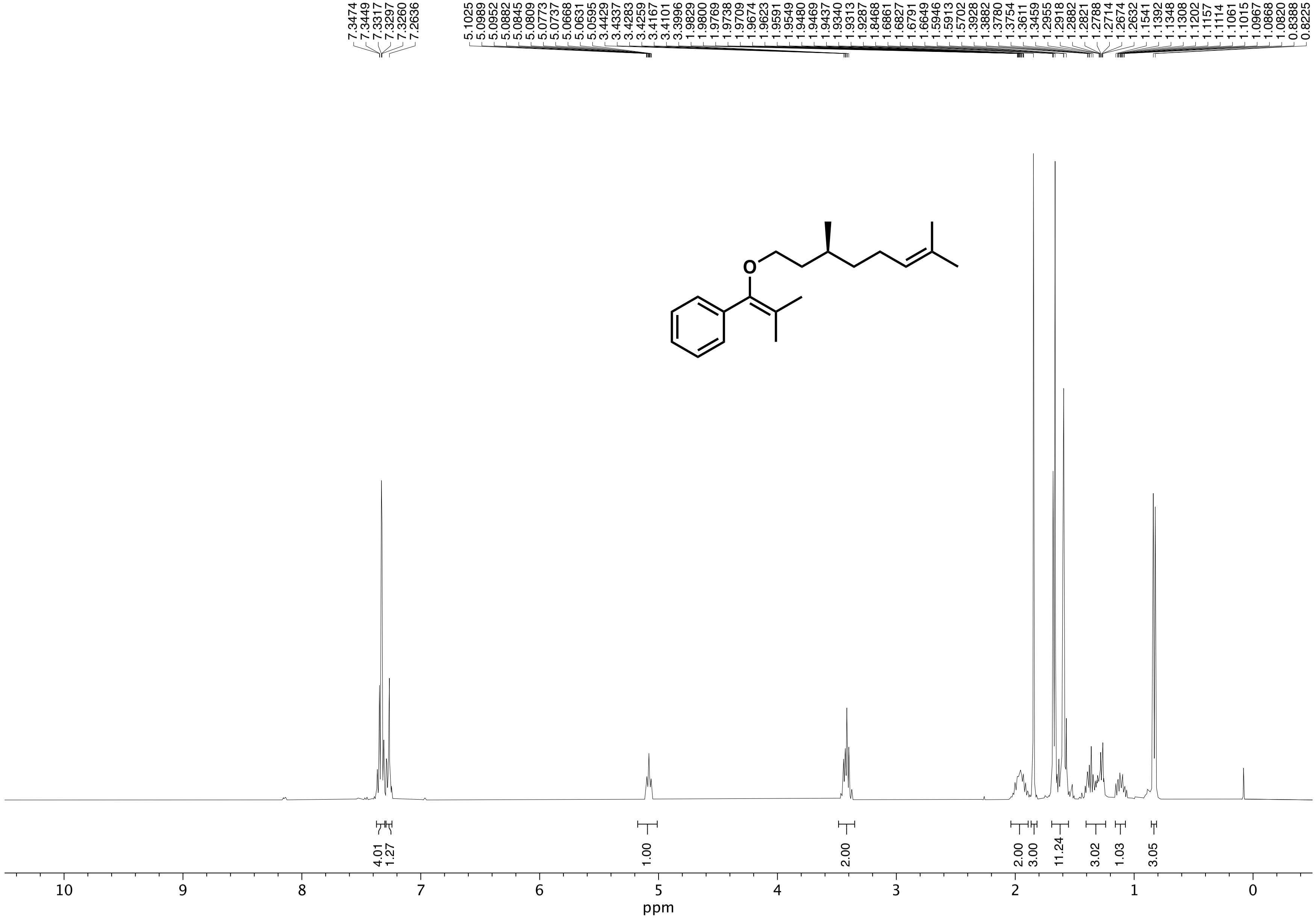

^{1}H NMR (400 MHz, $CDCl_3$) of **8j**.

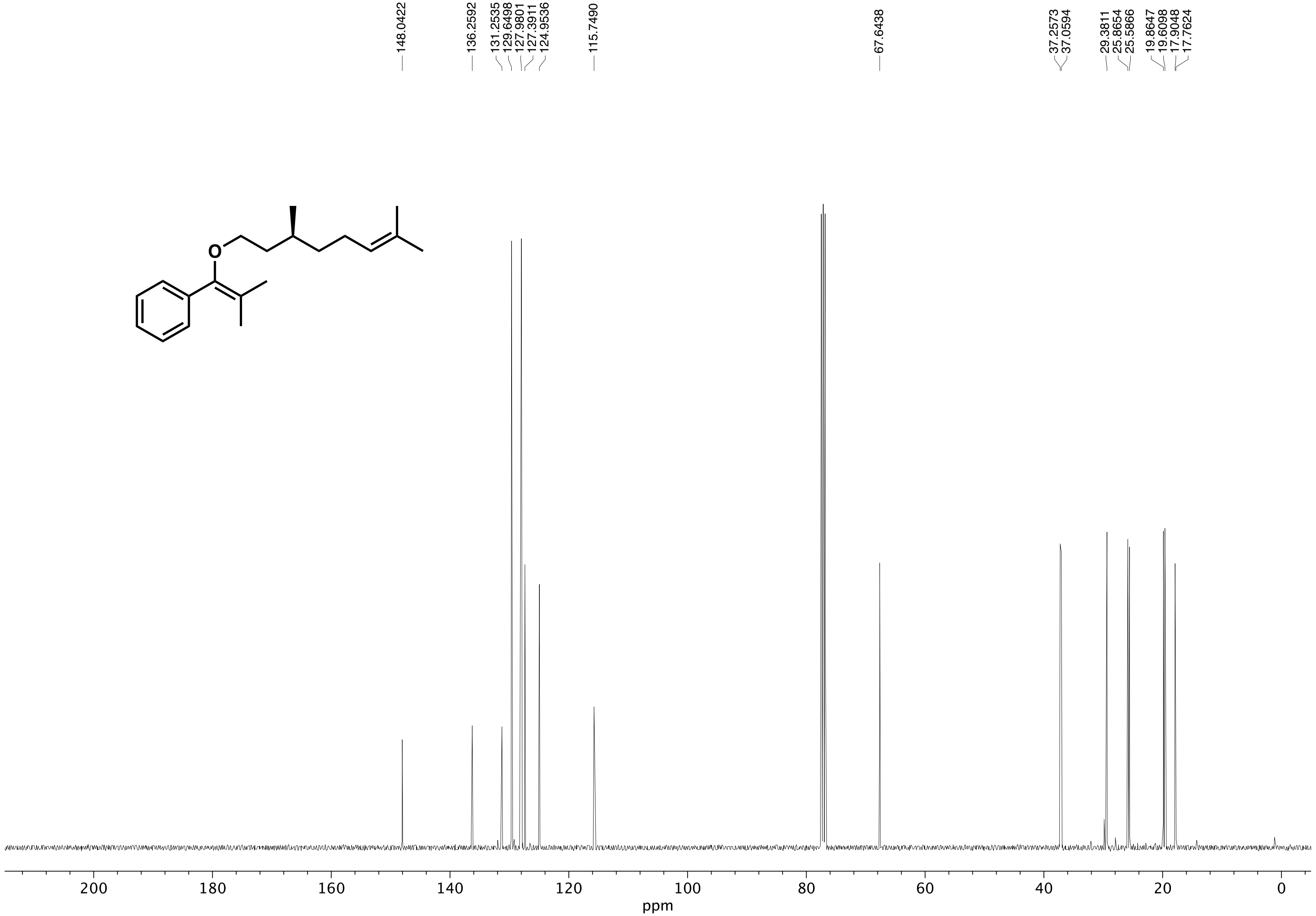

^{13}C NMR (101 MHz, $CDCl_3$) of **8j**.

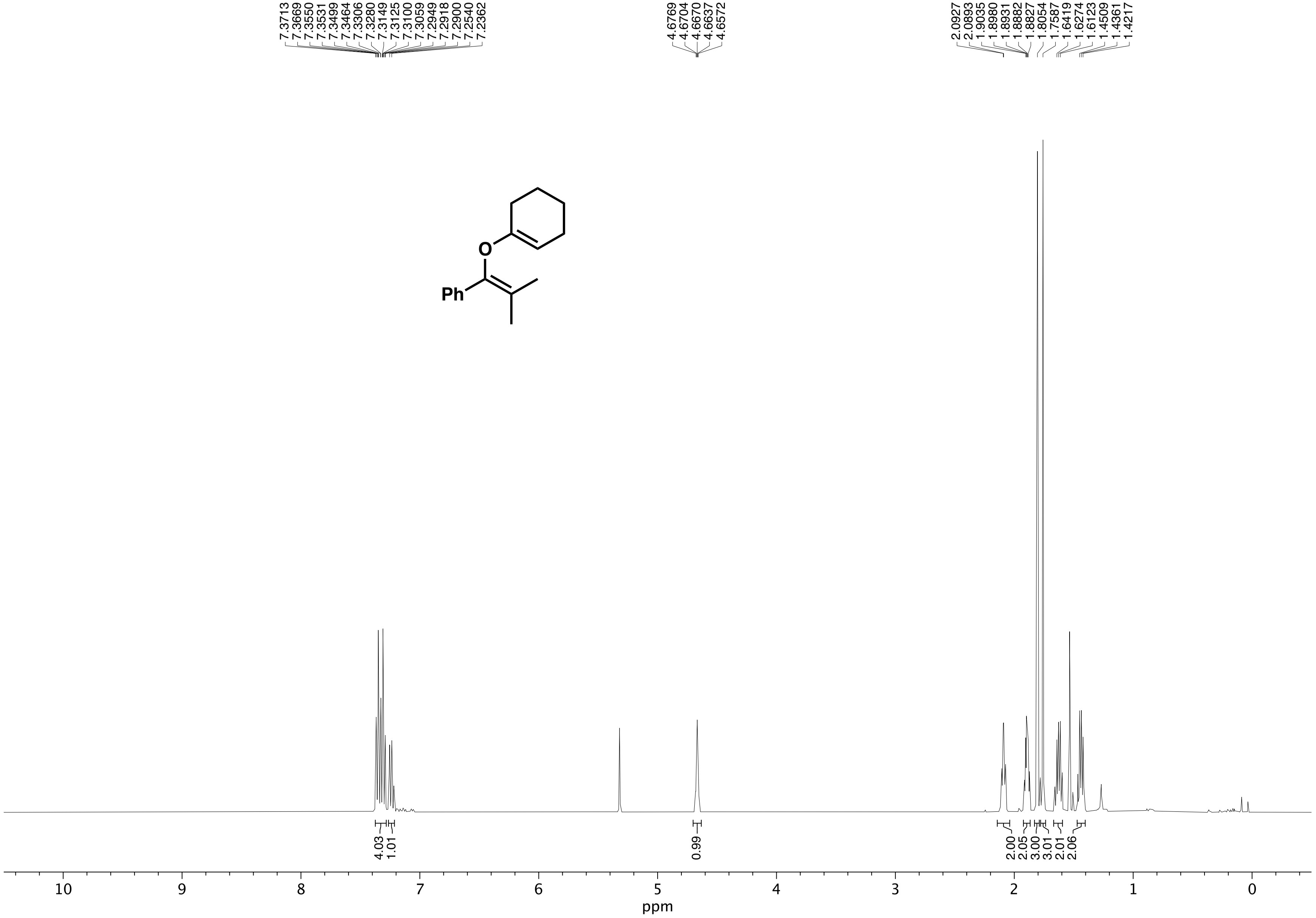

^{1}H NMR (400 MHz, CD_2Cl_2) of **8k**.

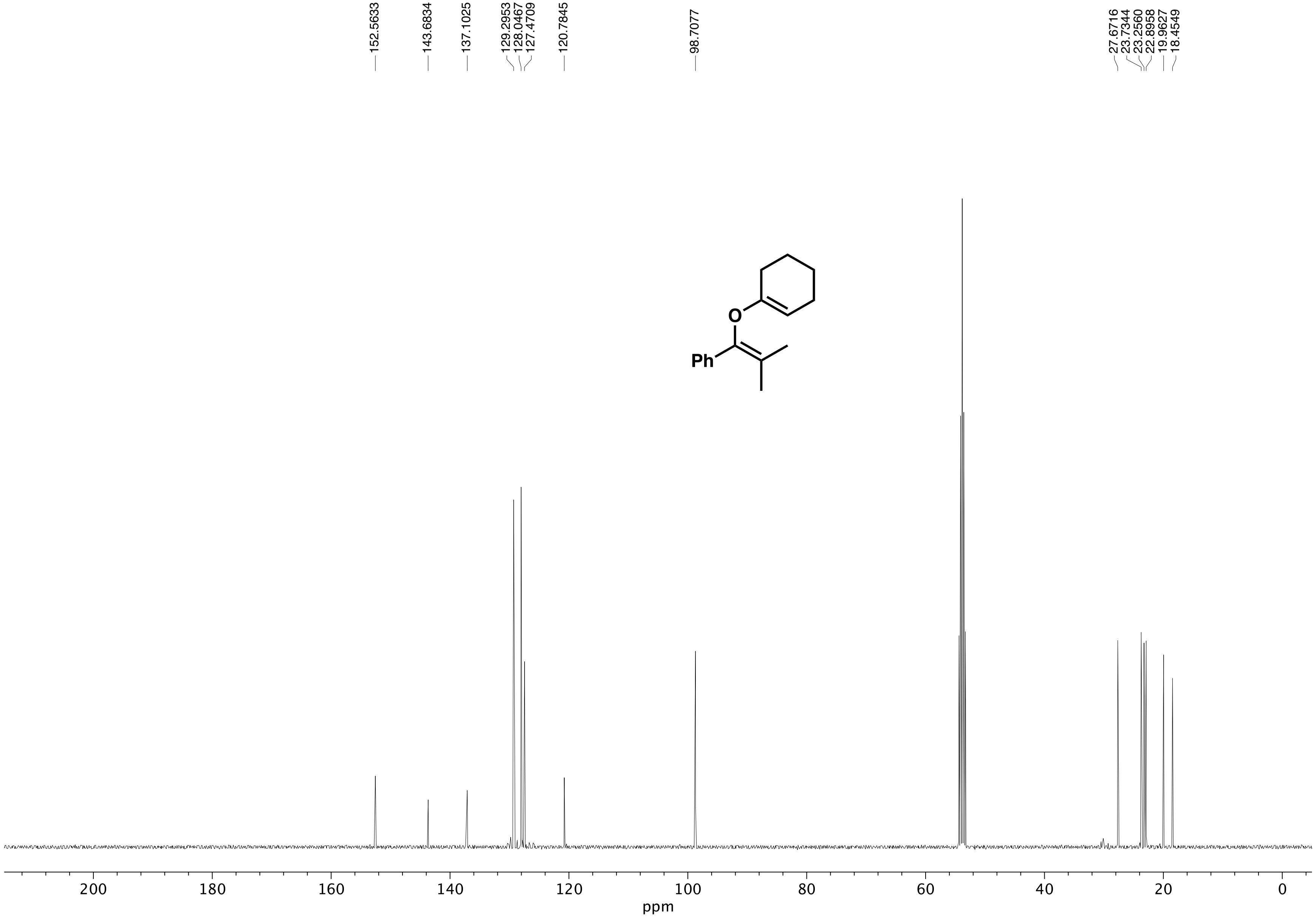

^{13}C NMR (101 MHz, CD_2Cl_2) of **8k.**

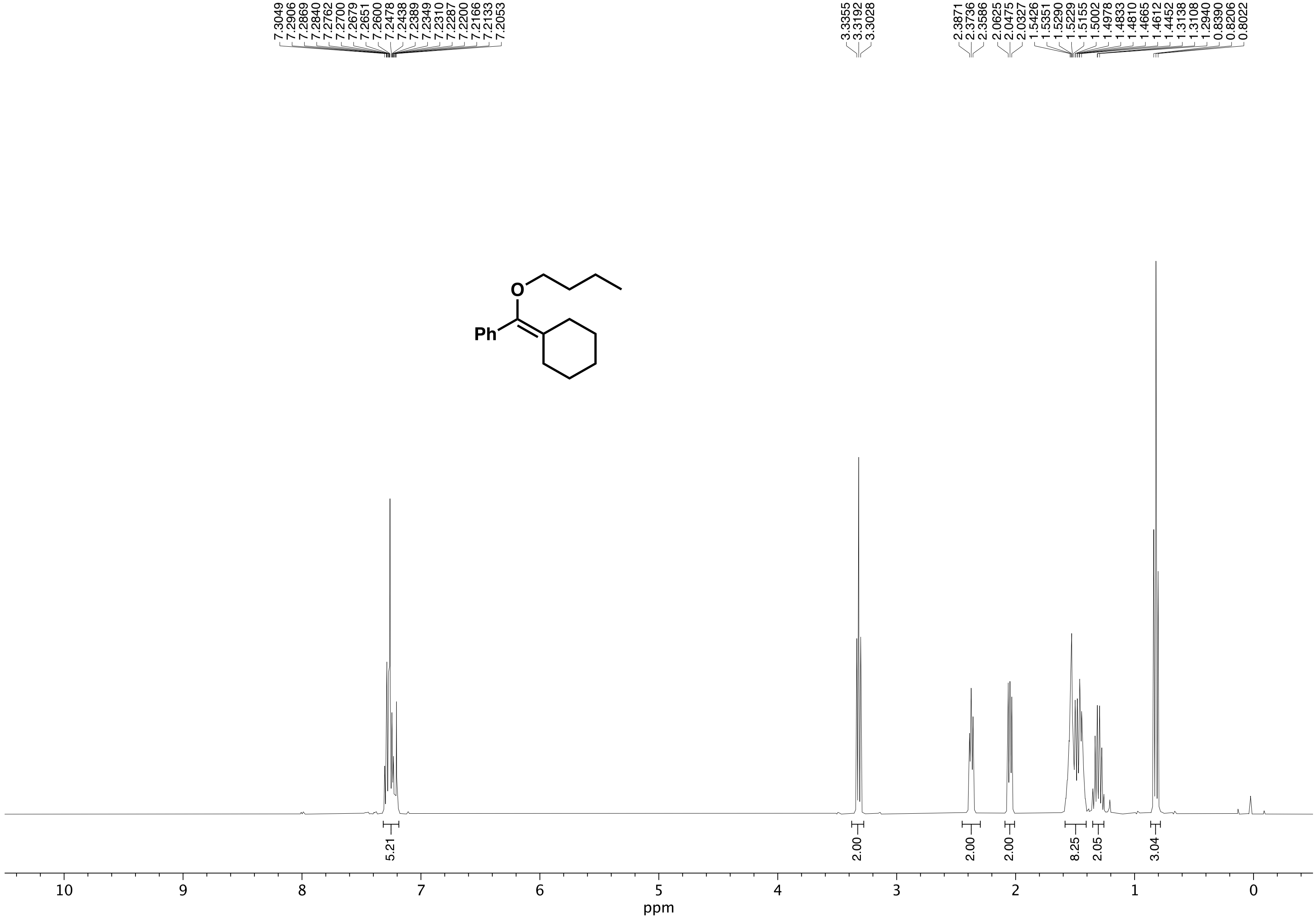

^{1}H NMR (400 MHz, $CDCl_3$) of **8l.**

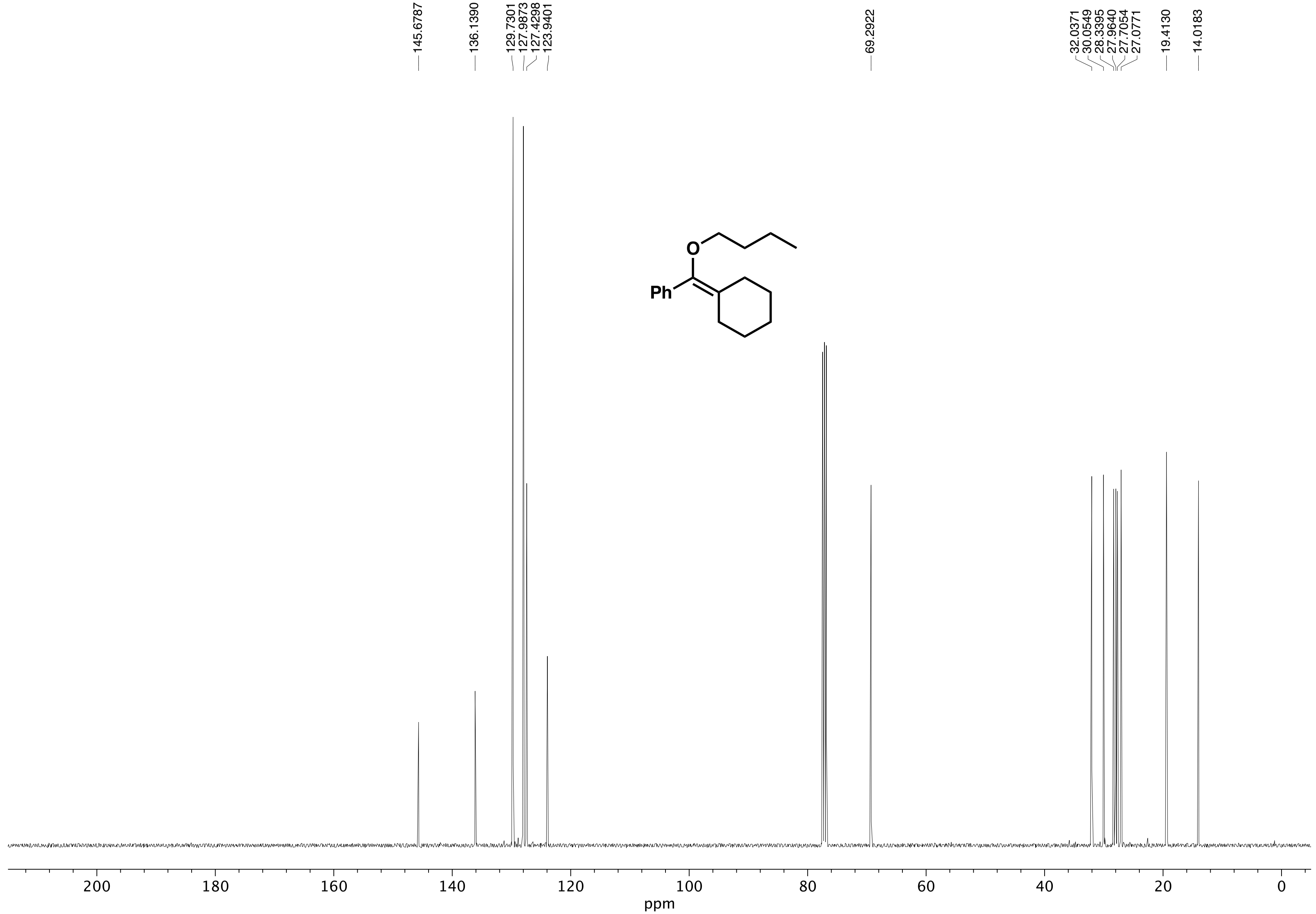

^{13}C NMR (101 MHz, $CDCl_3$) of **8l**.

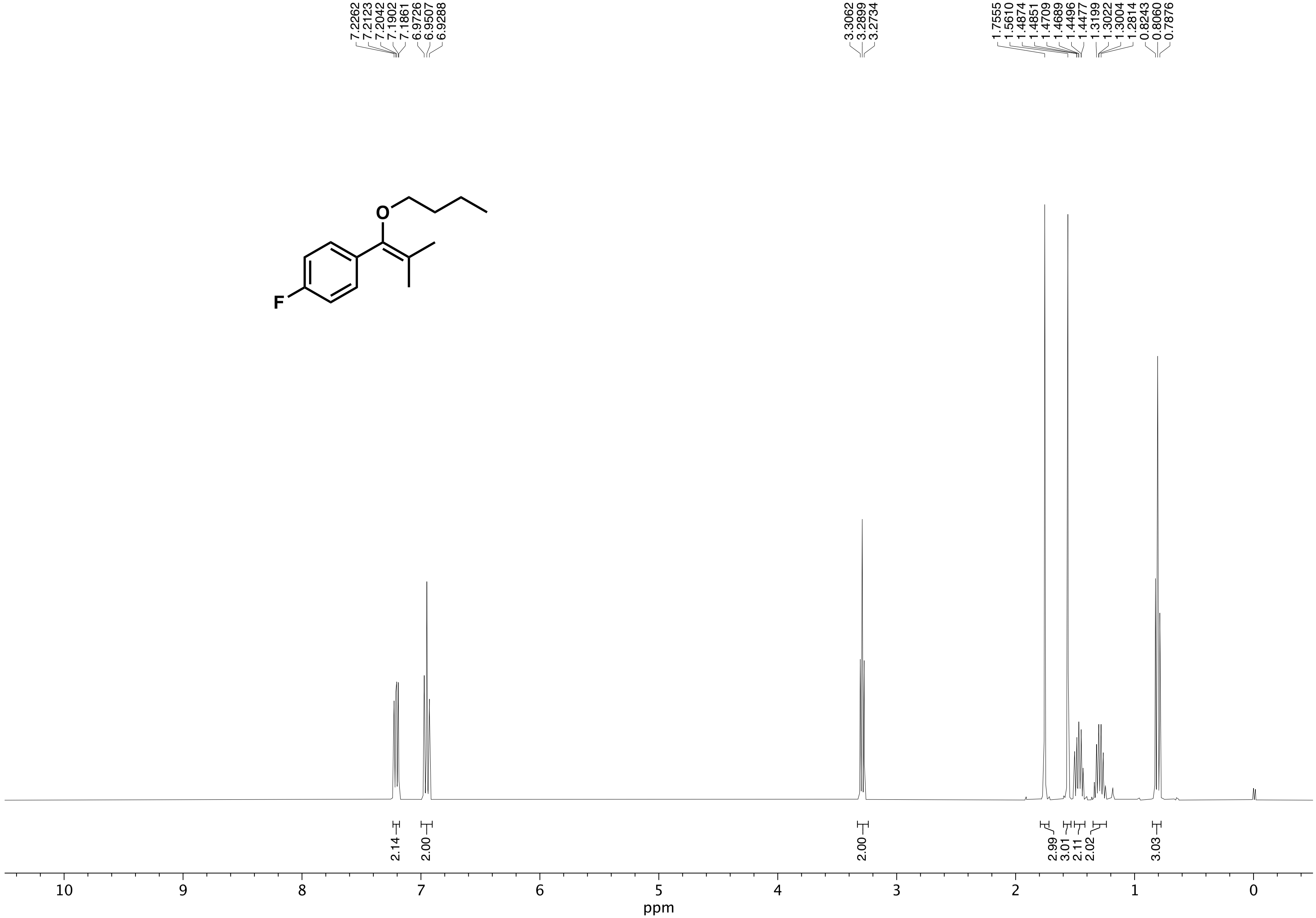

^{1}H NMR (400 MHz, $CDCl_3$) of **8m**.

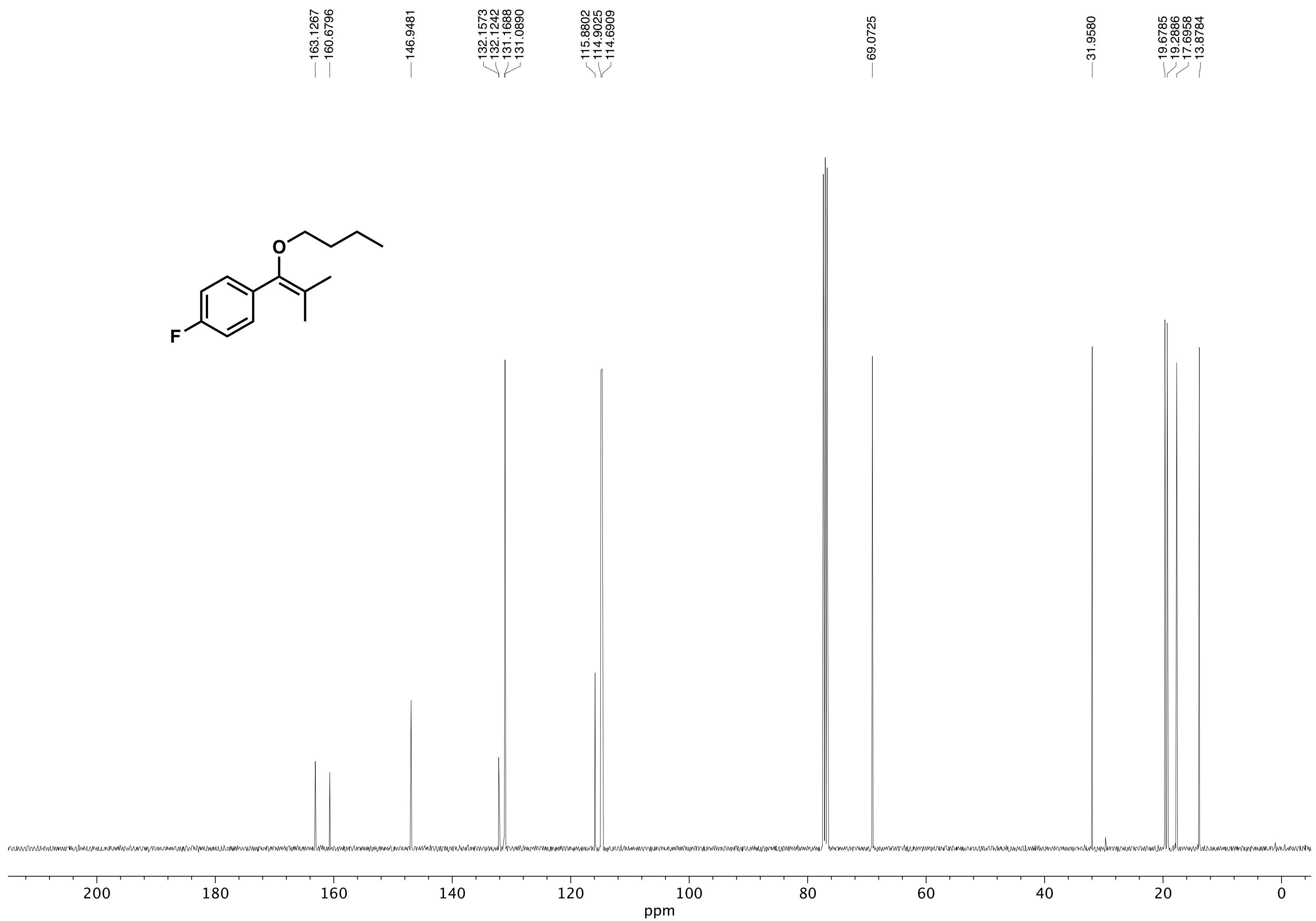

^{13}C NMR (101 MHz, $CDCl_3$) of **8m**.

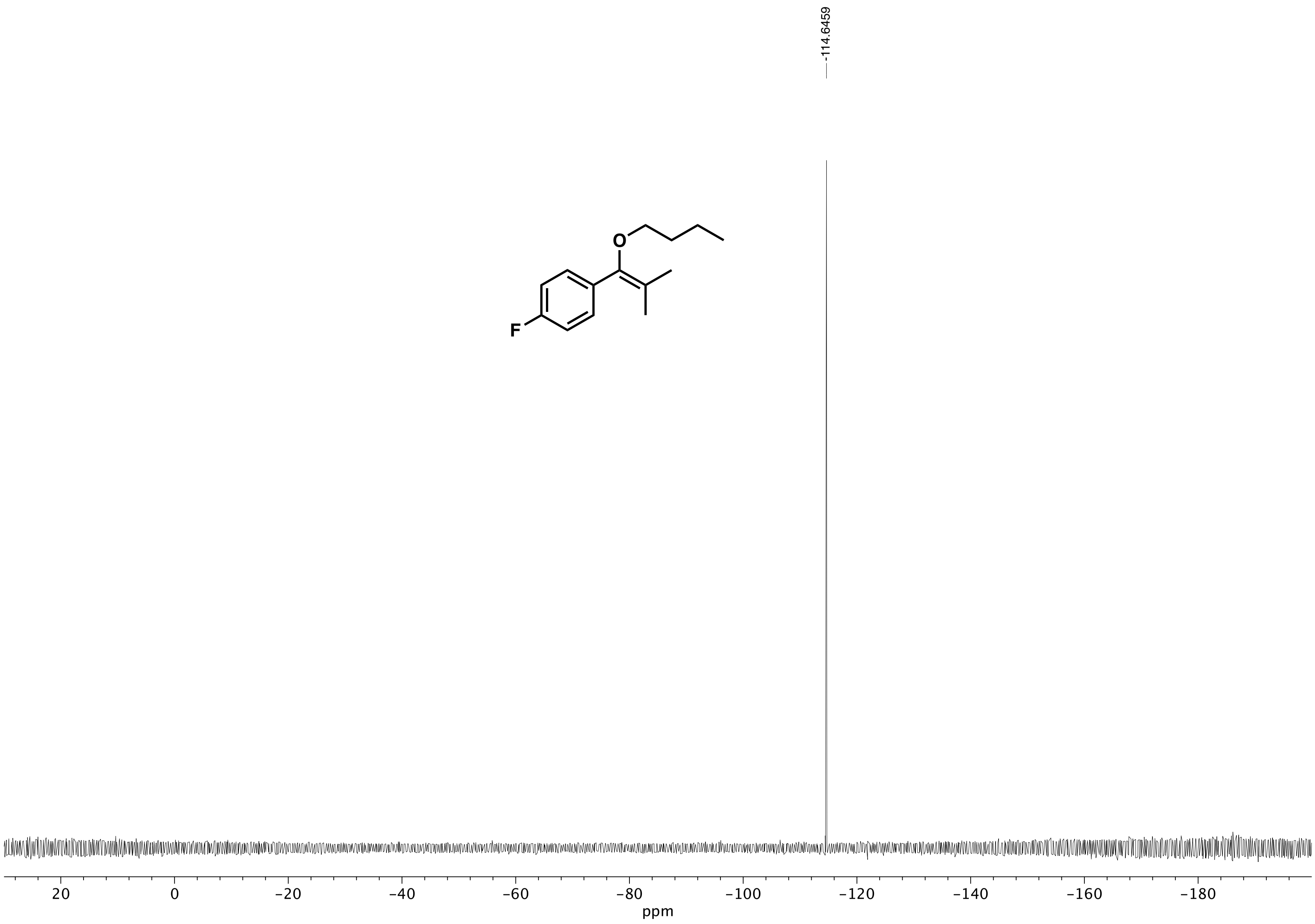

^{19}F NMR (282 MHz, $CDCl_3$) of **8m**.

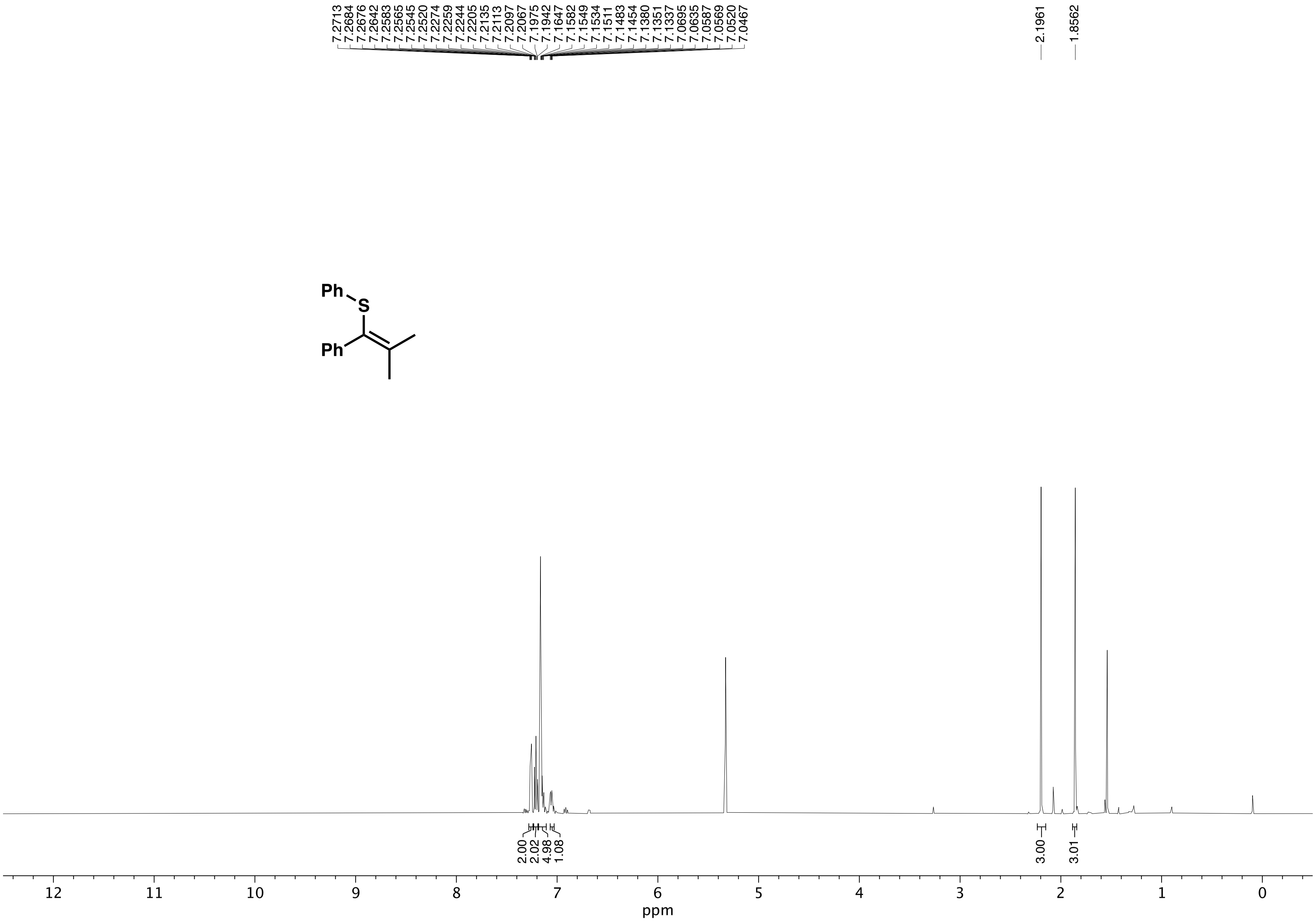

^1H NMR (400 MHz, d_2-DCM) of **8n**.

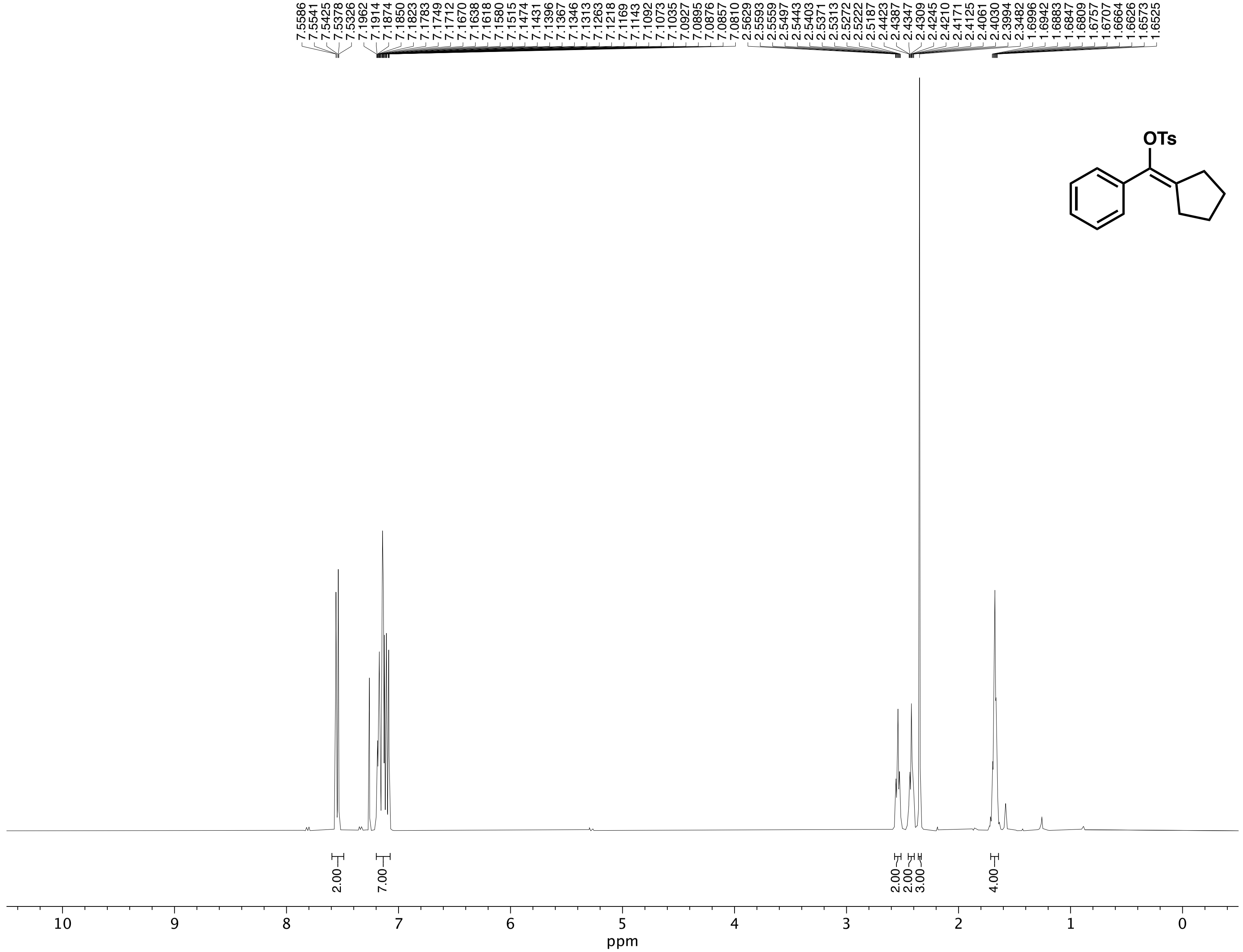

^{1}H NMR (400 MHz, $CDCl_3$) of compound **SI-10**.

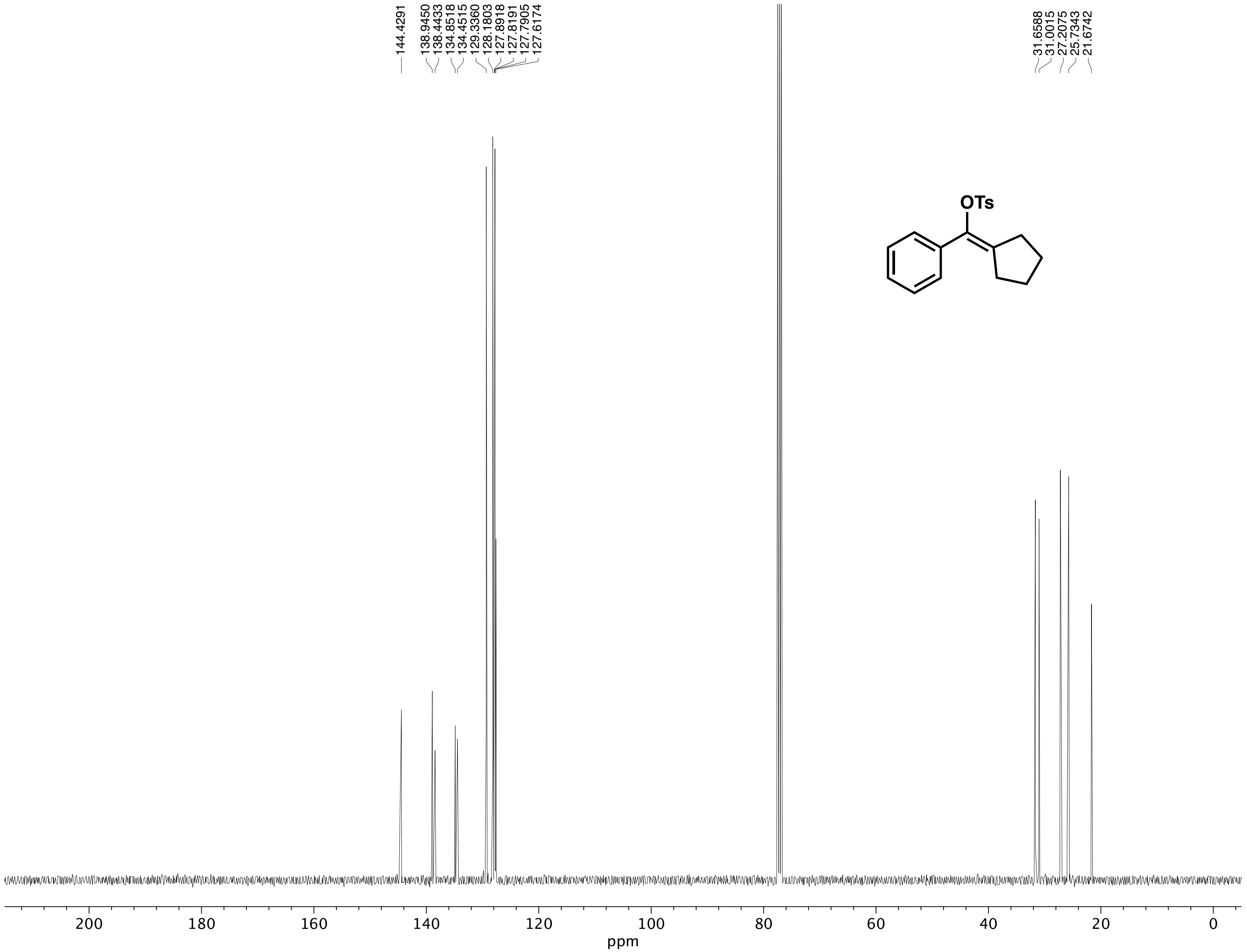

^{13}C NMR (101 MHz, $CDCl_3$) of compound **SI-10**.

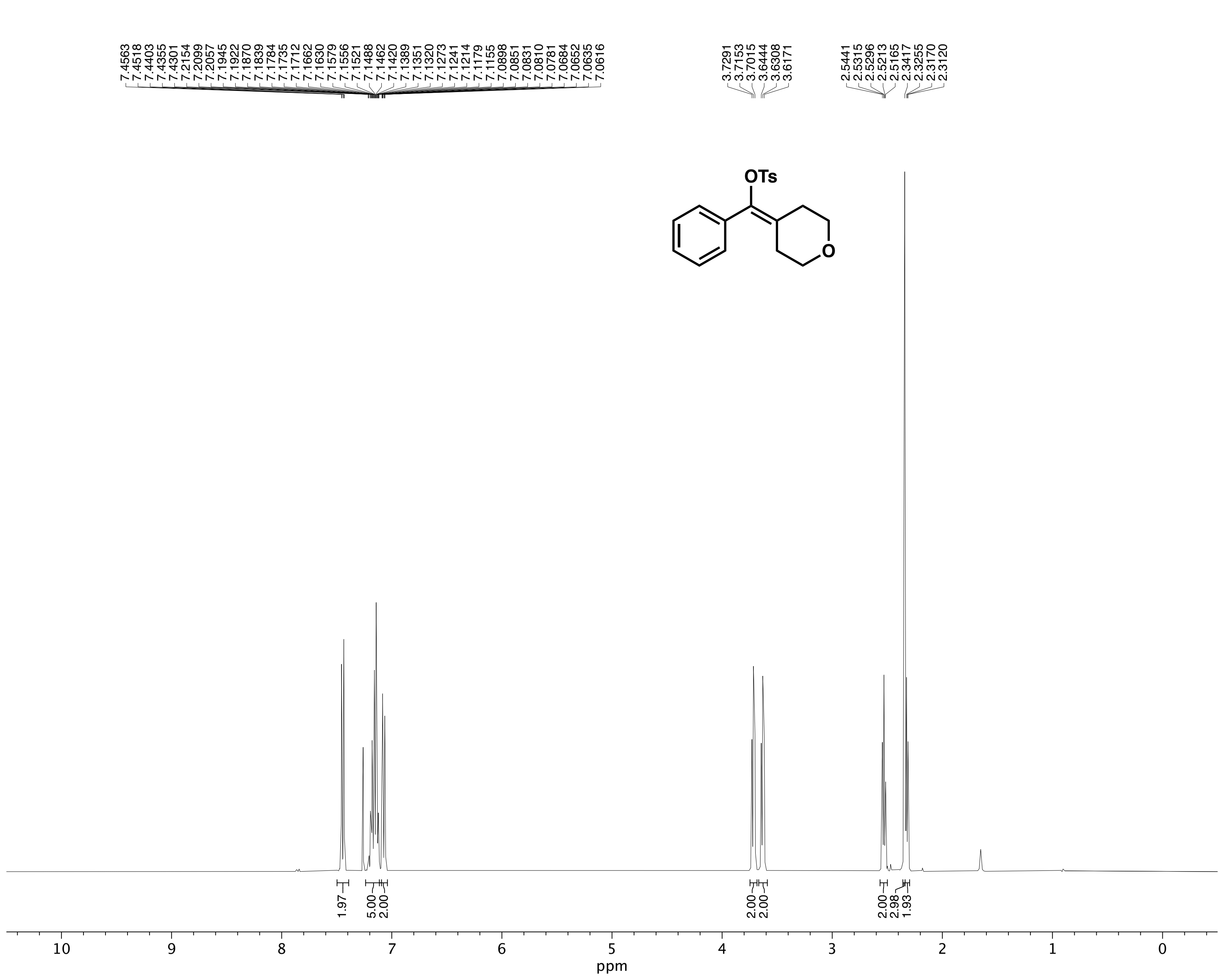

^{1}H NMR (400 MHz, $CDCl_3$) of compound **SI-11**.

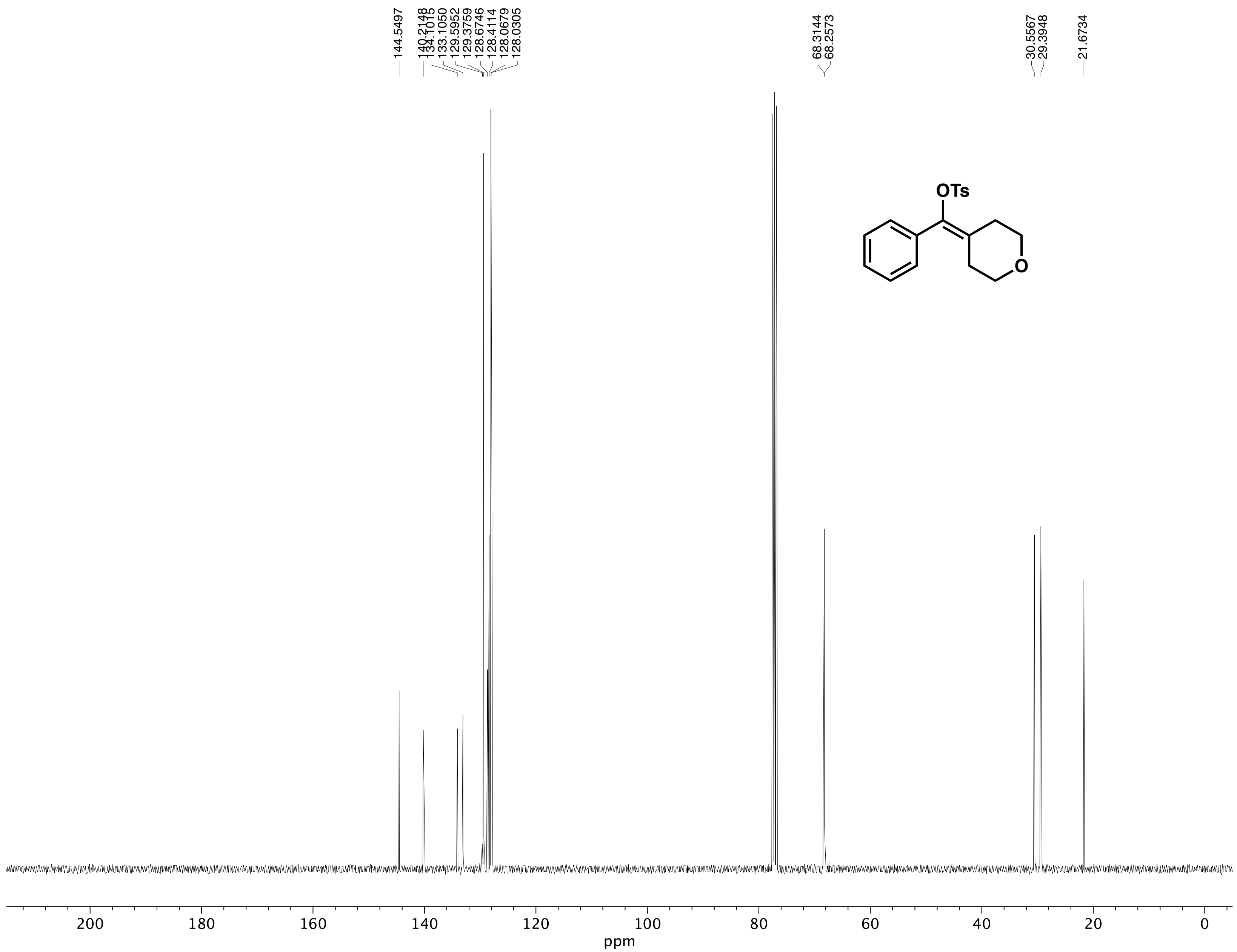

^{13}C NMR (101 MHz, $CDCl_3$) of compound **SI-11**.

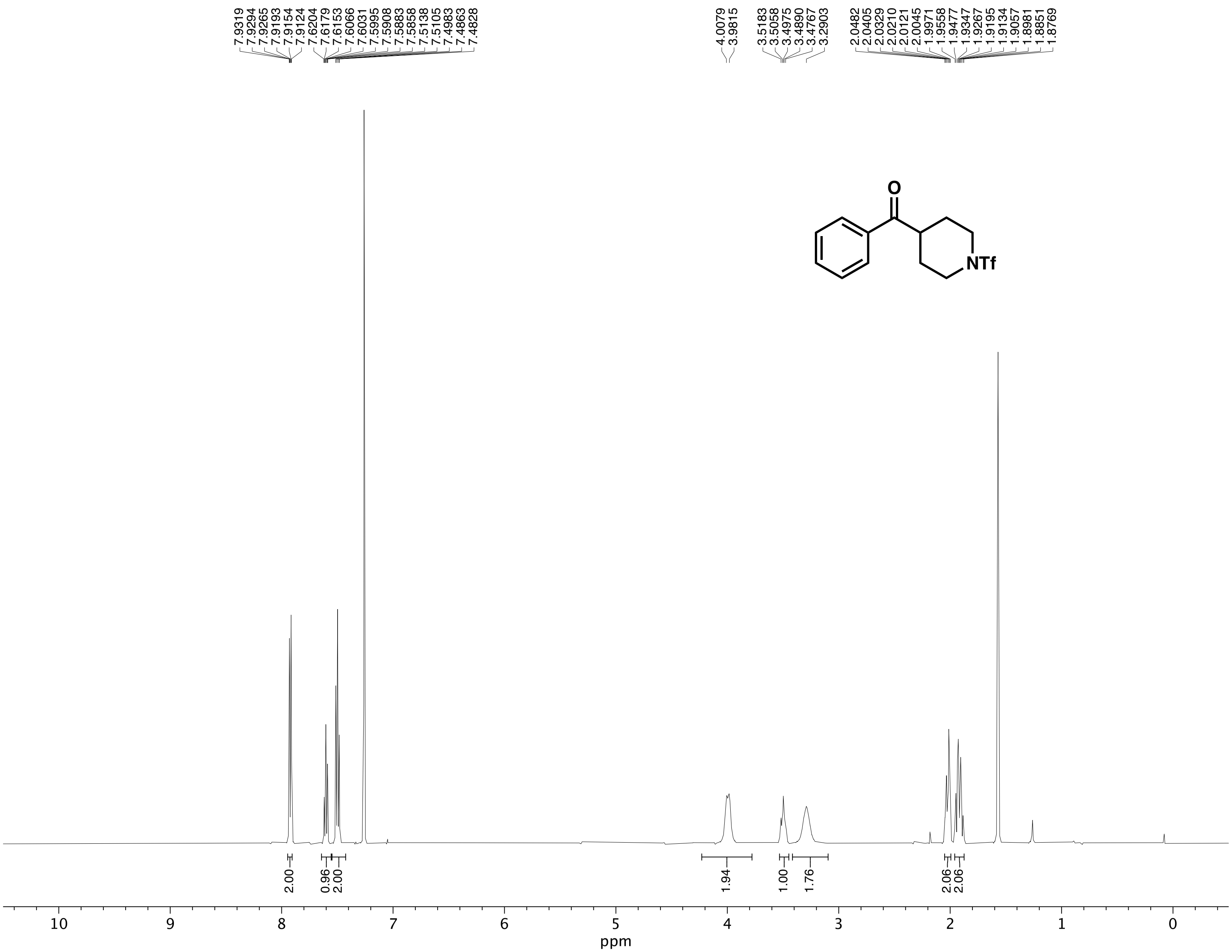

^{1}H NMR (500 MHz, $CDCl_3$) of compound **SI-12**.

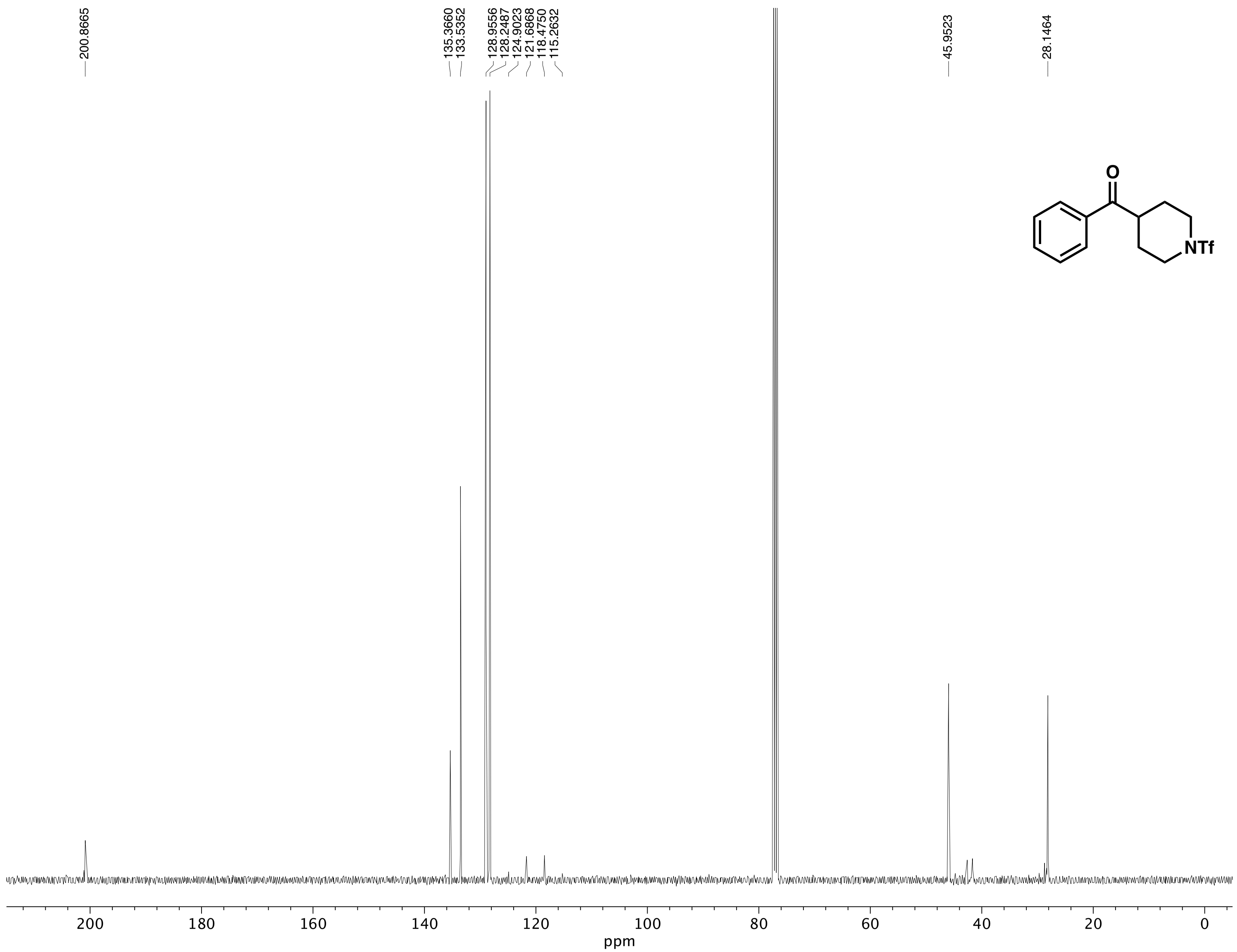

^{13}C NMR (101 MHz, $CDCl_3$) of compound **SI-12**.

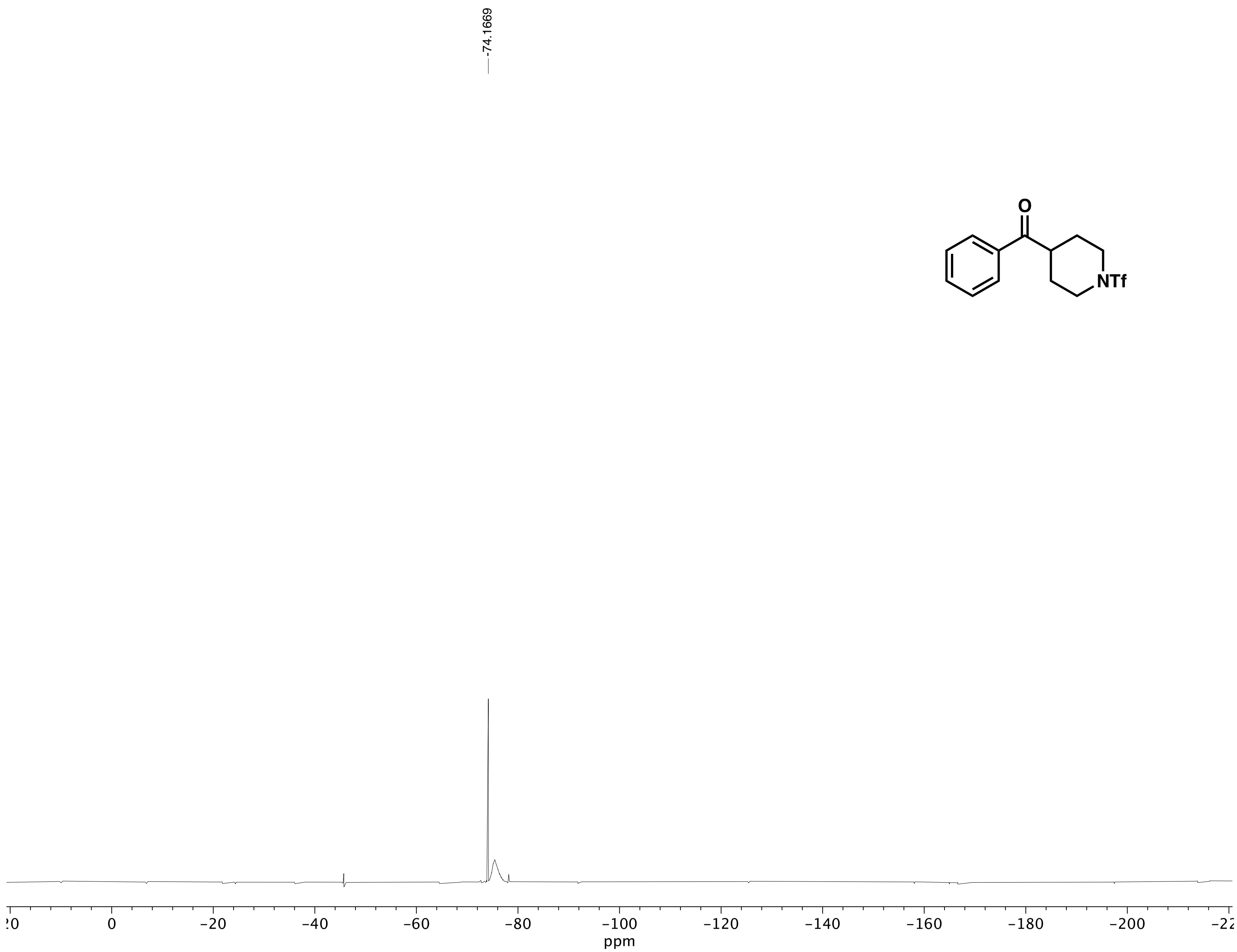

^{19}F NMR (376 MHz, $CDCl_3$) of compound **SI-12**.

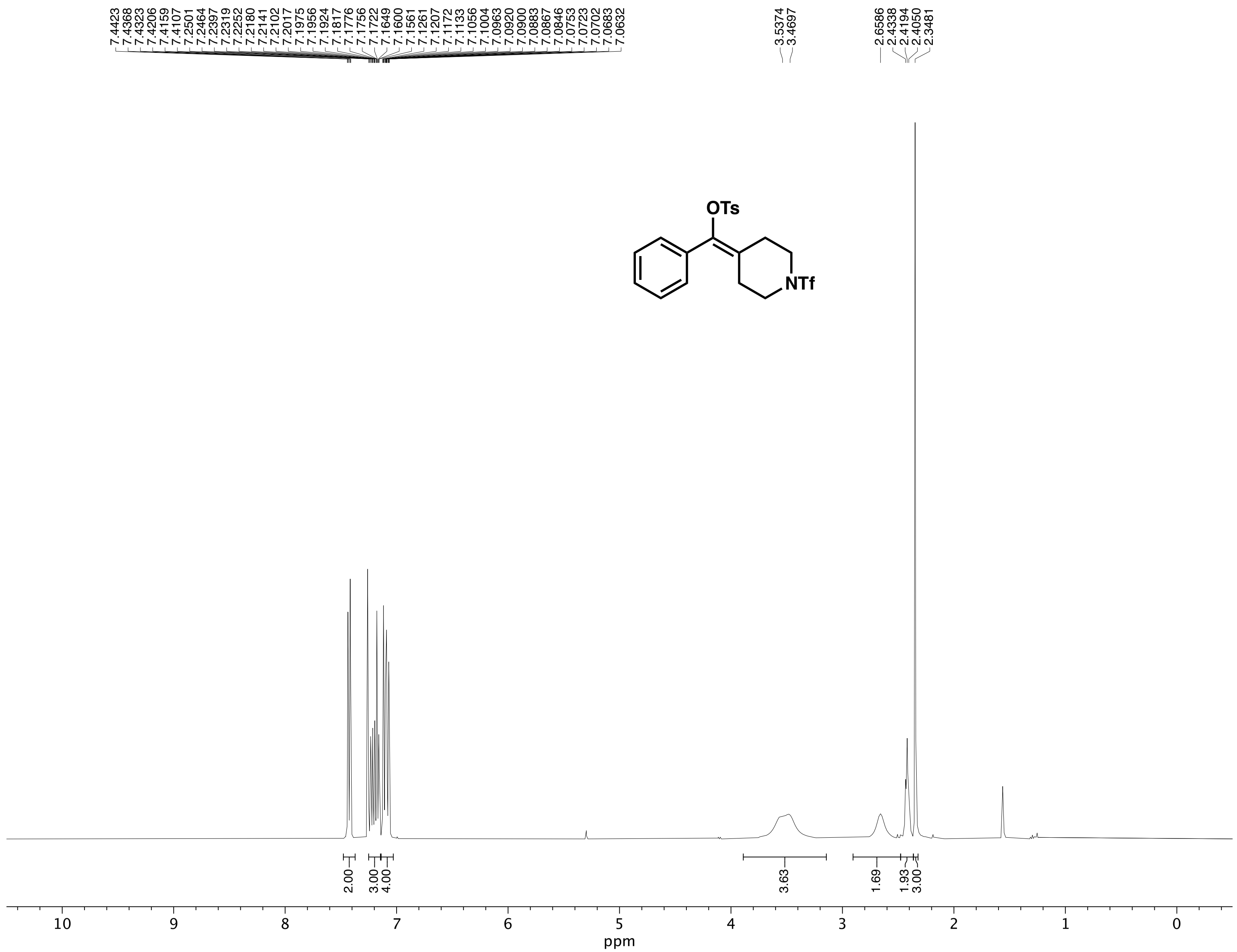

^{1}H NMR (400 MHz, $CDCl_3$) of compound **SI-13**

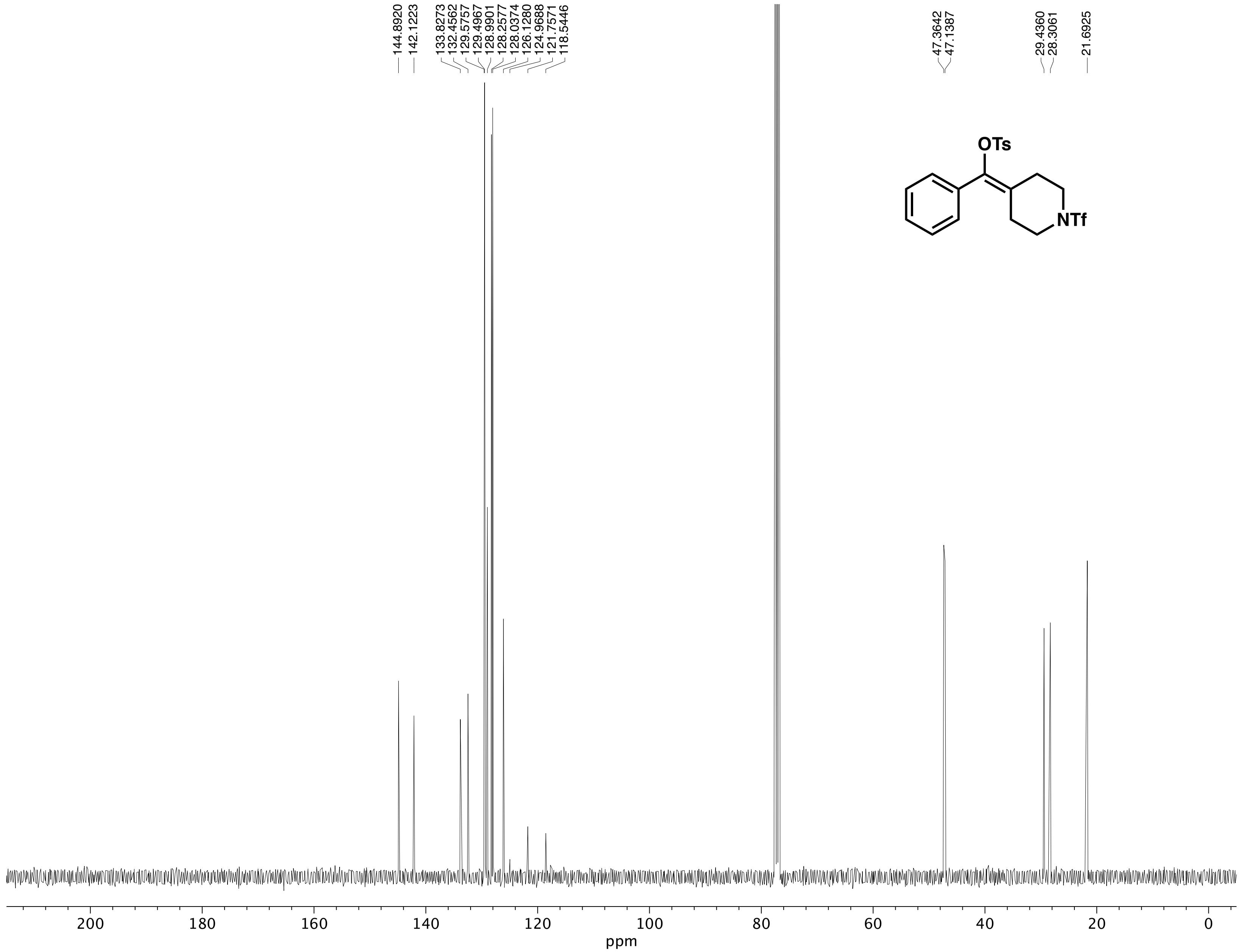

^{13}C NMR (101 MHz, $CDCl_3$) of compound **SI-13**.

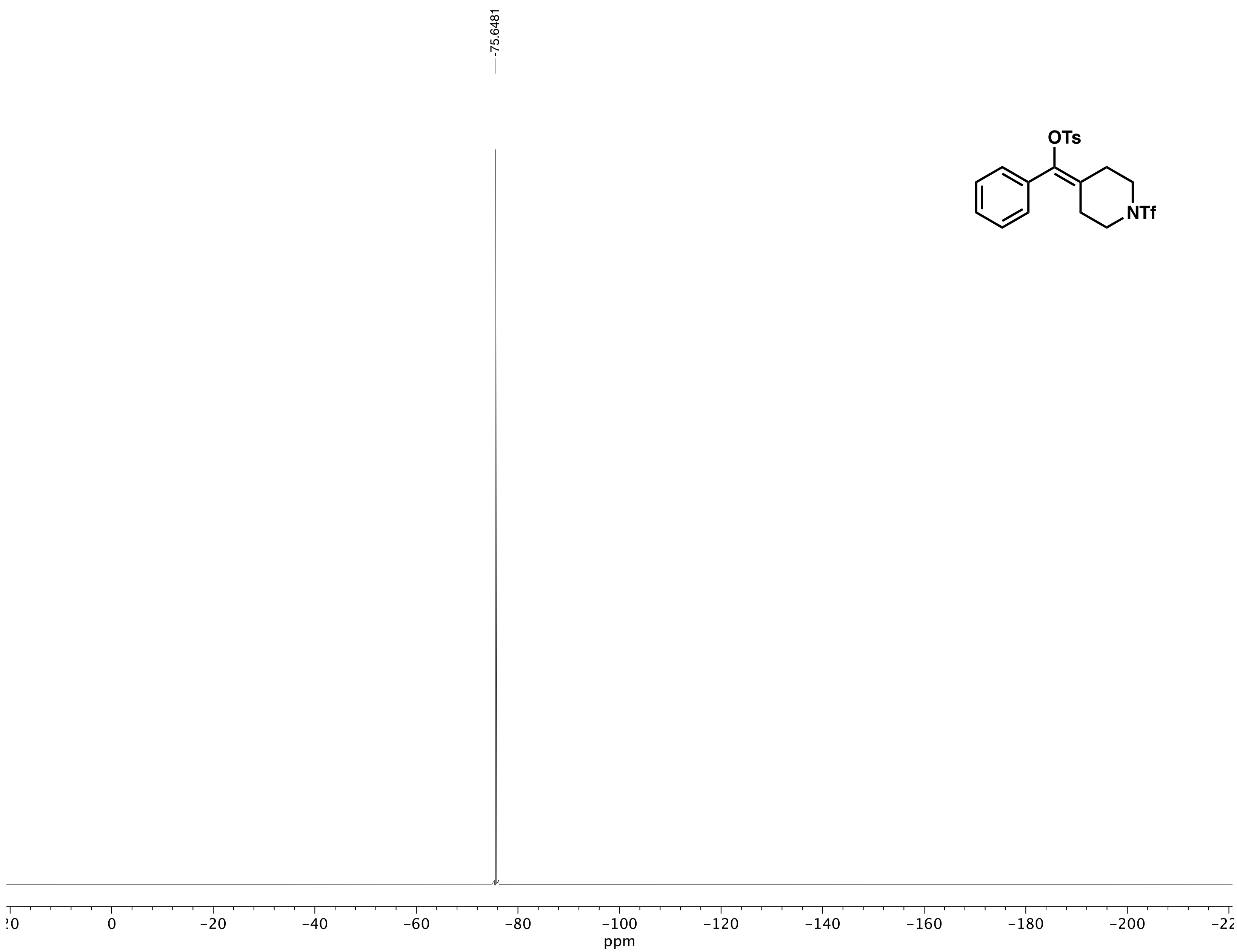

^{19}F NMR (376 MHz, $CDCl_3$) of compound **SI-13**.

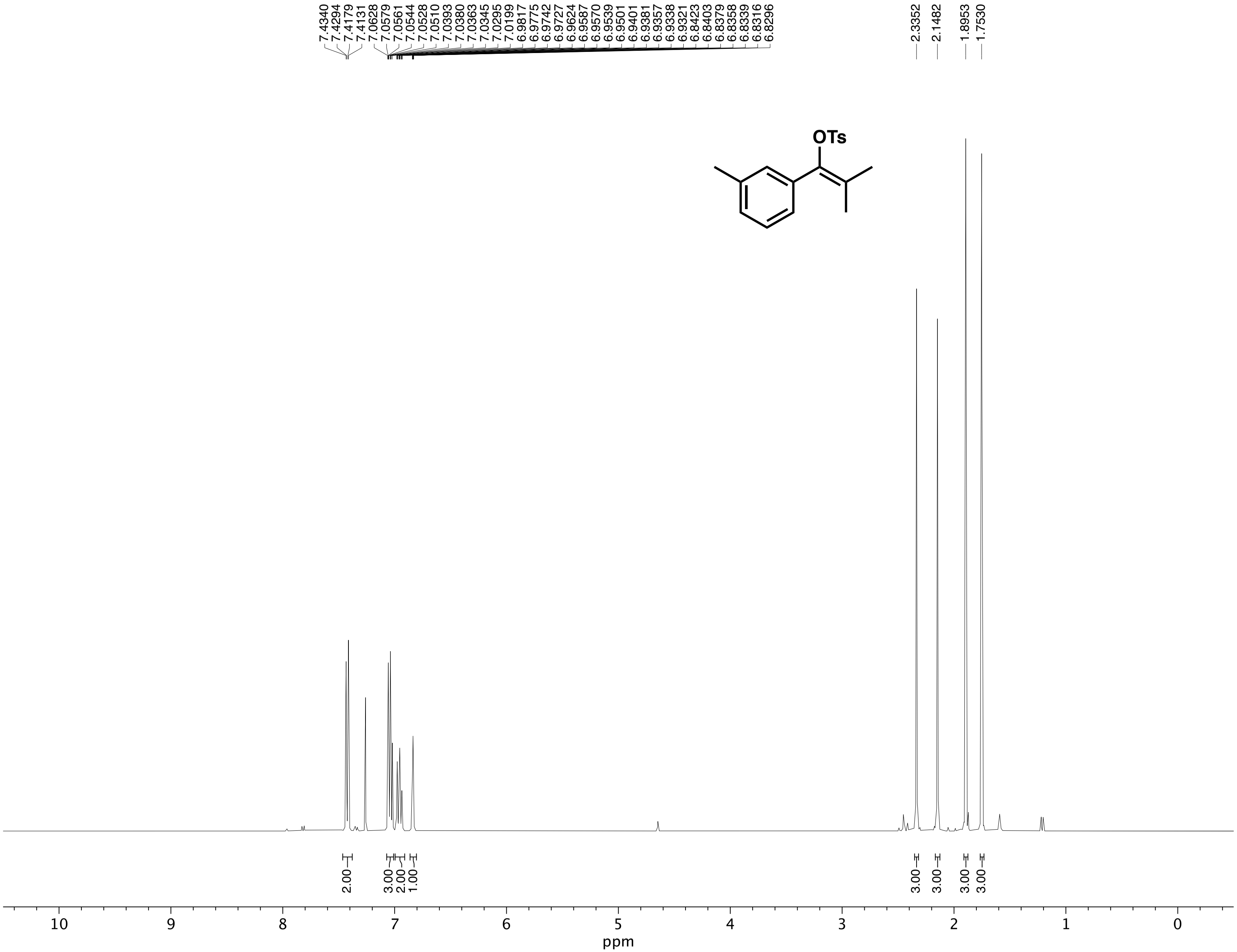

^{1}H NMR (400 MHz, $CDCl_3$) of compound **SI-15**.

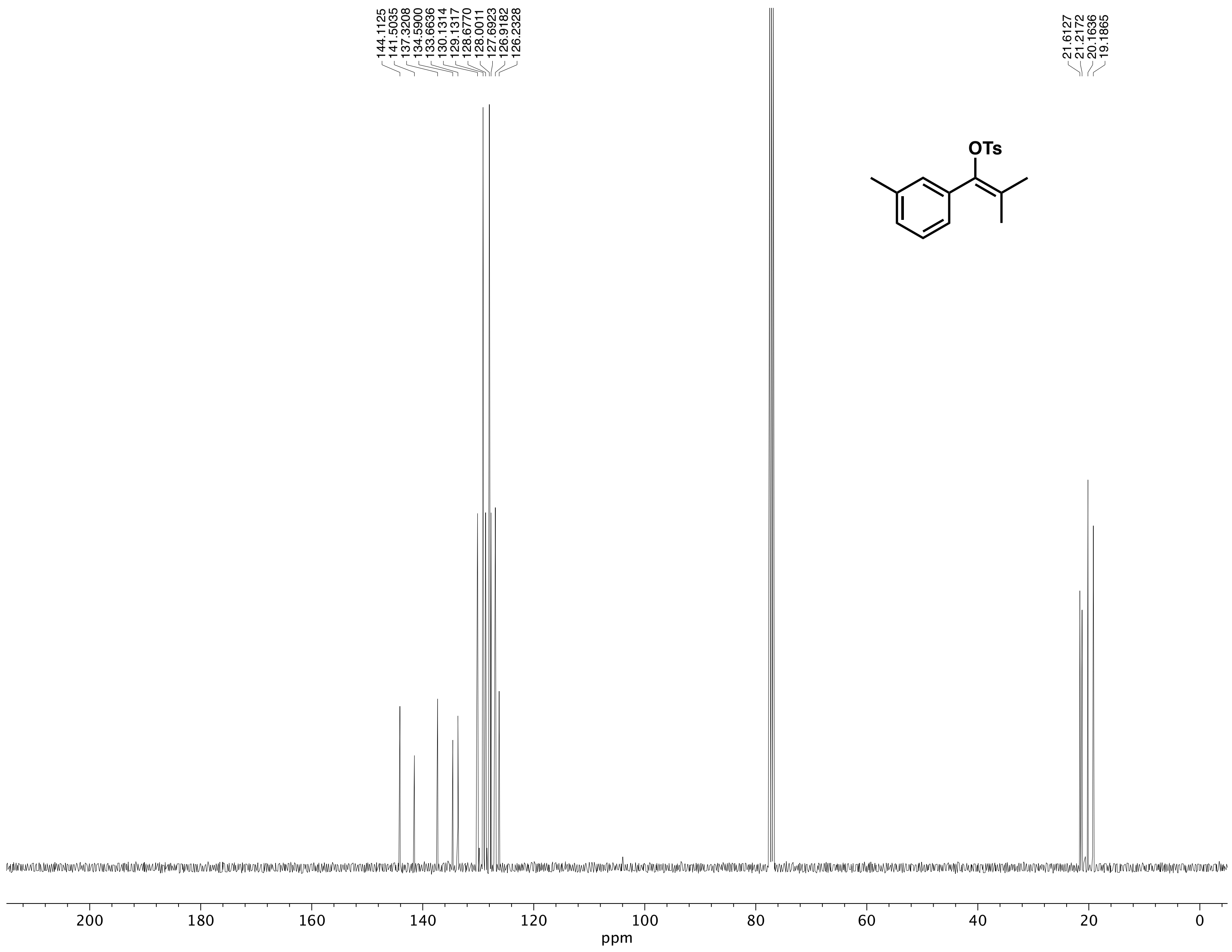

^{13}C NMR (101 MHz, $CDCl_3$) of compound **SI-15**.

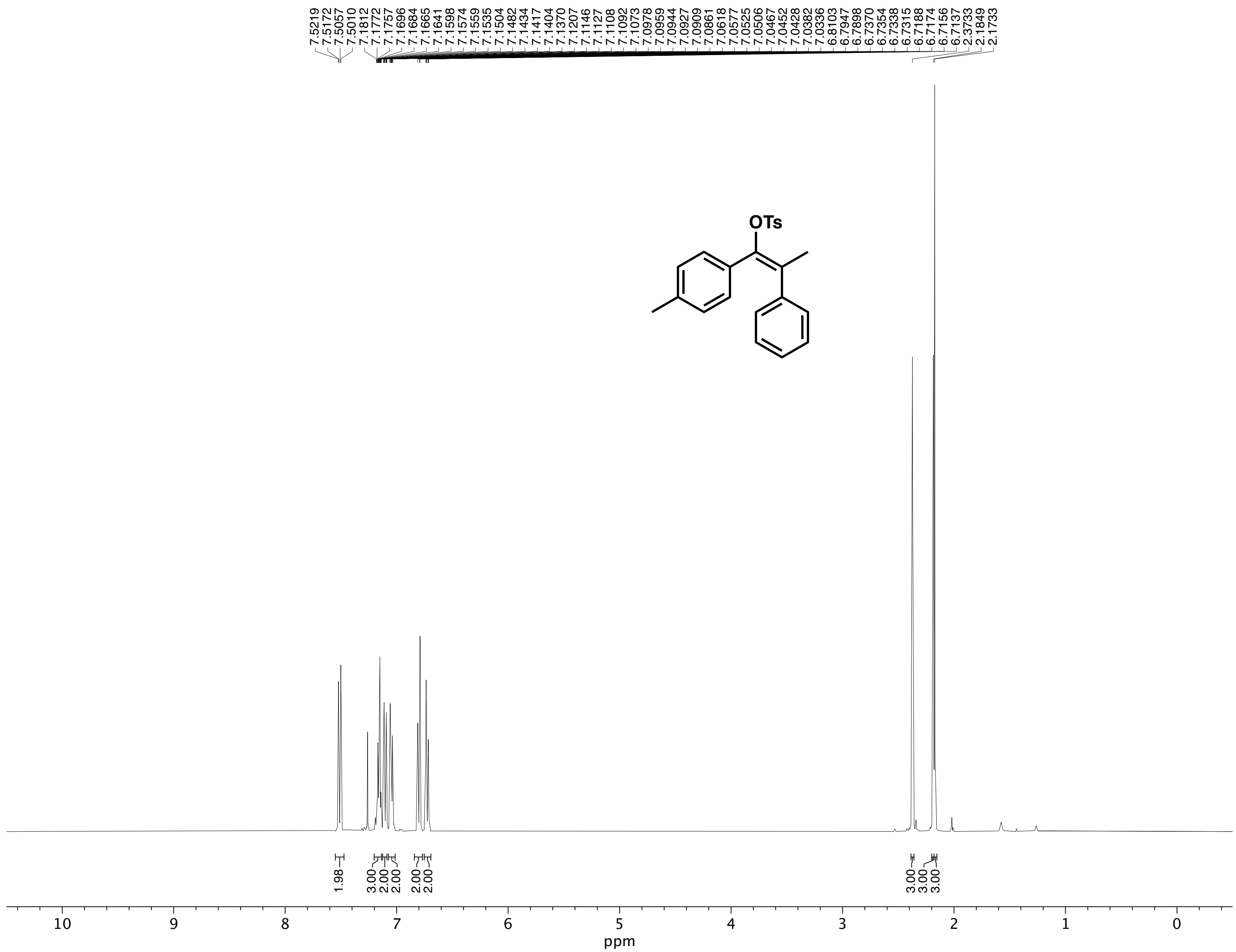

^{1}H NMR (400 MHz, $CDCl_3$) of compound **SI-20**.

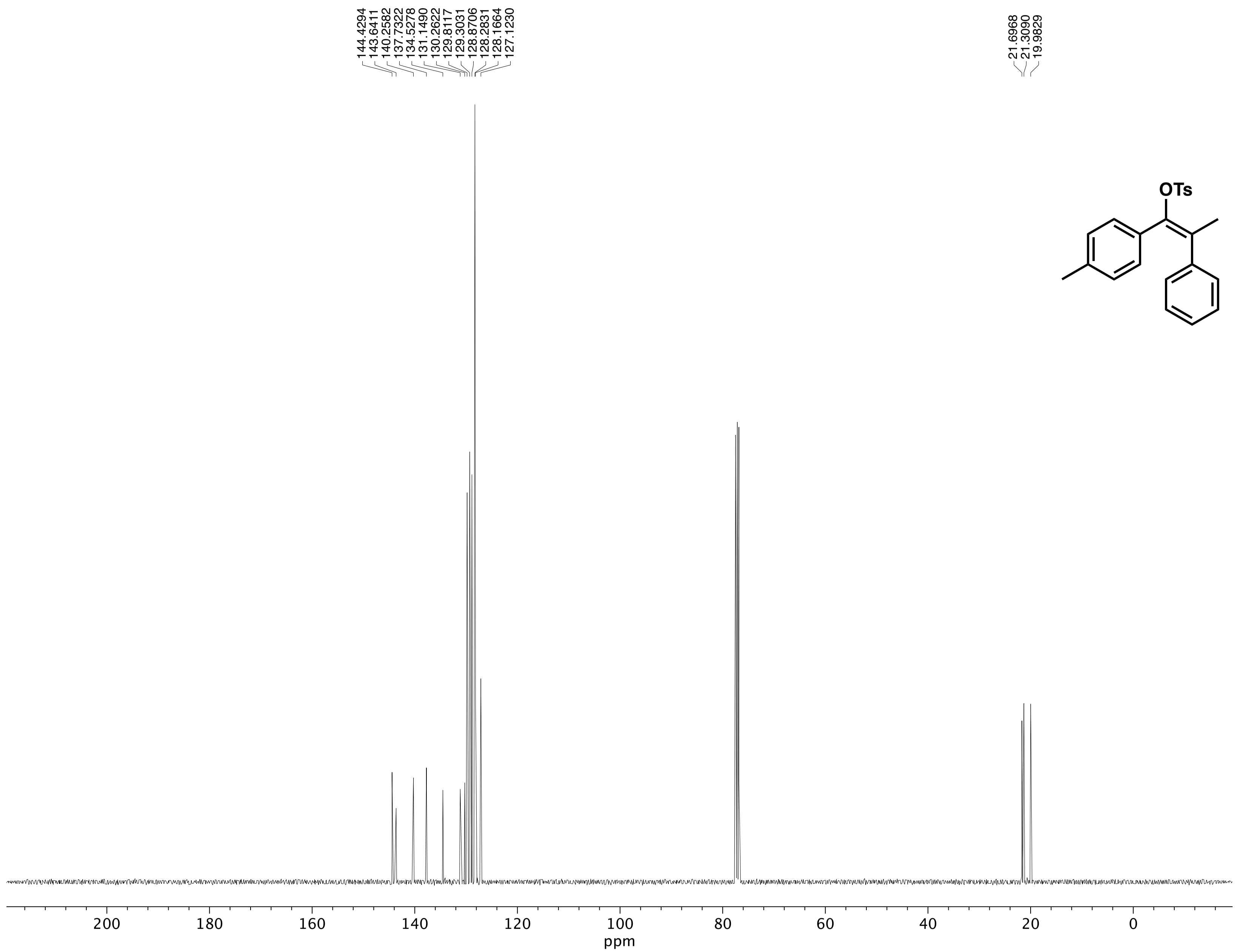

^{13}C NMR (101 MHz, $CDCl_3$) of compound **SI-20**.

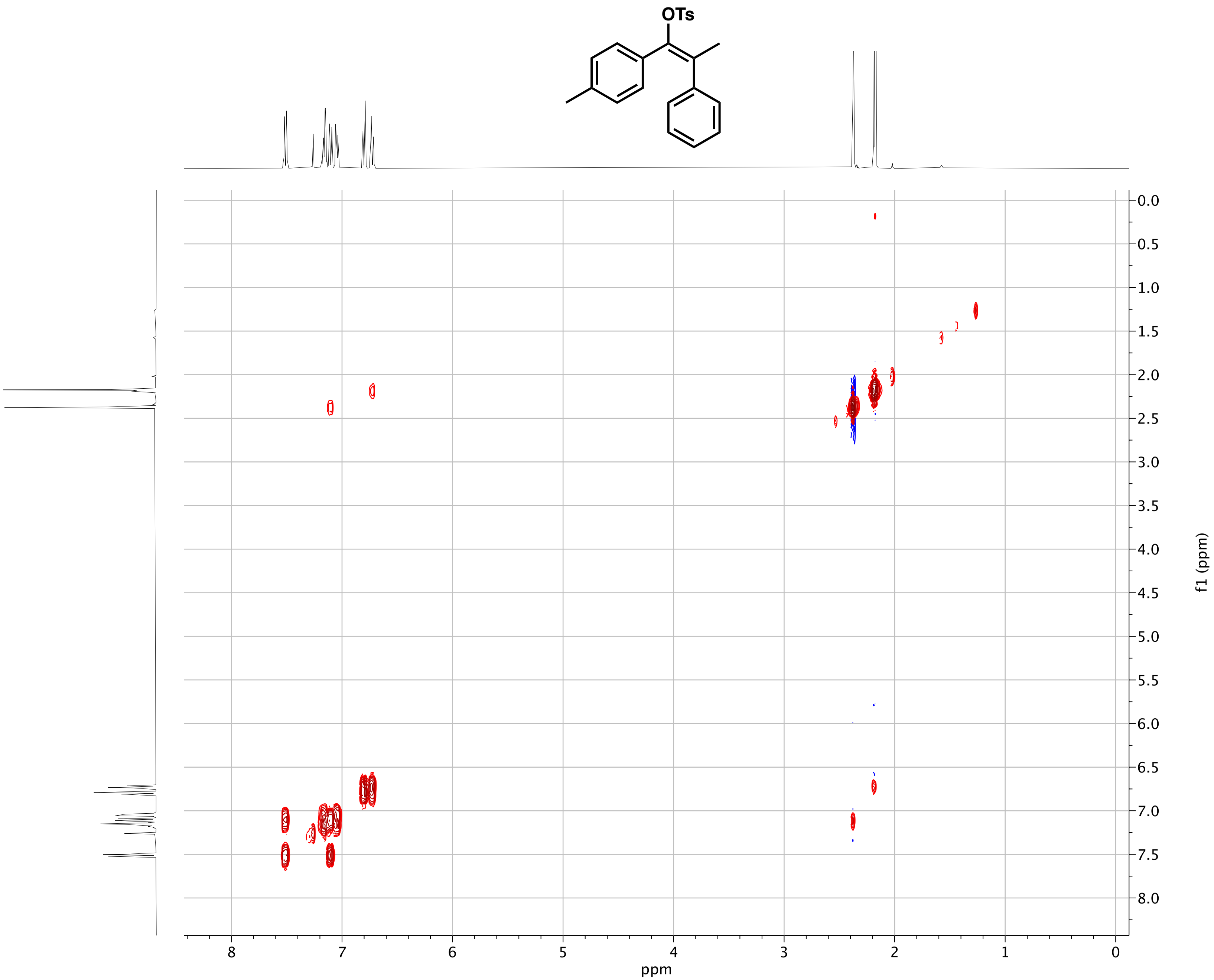

COSEY (101 MHz, $CDCl_3$) of compound **SI-20**.

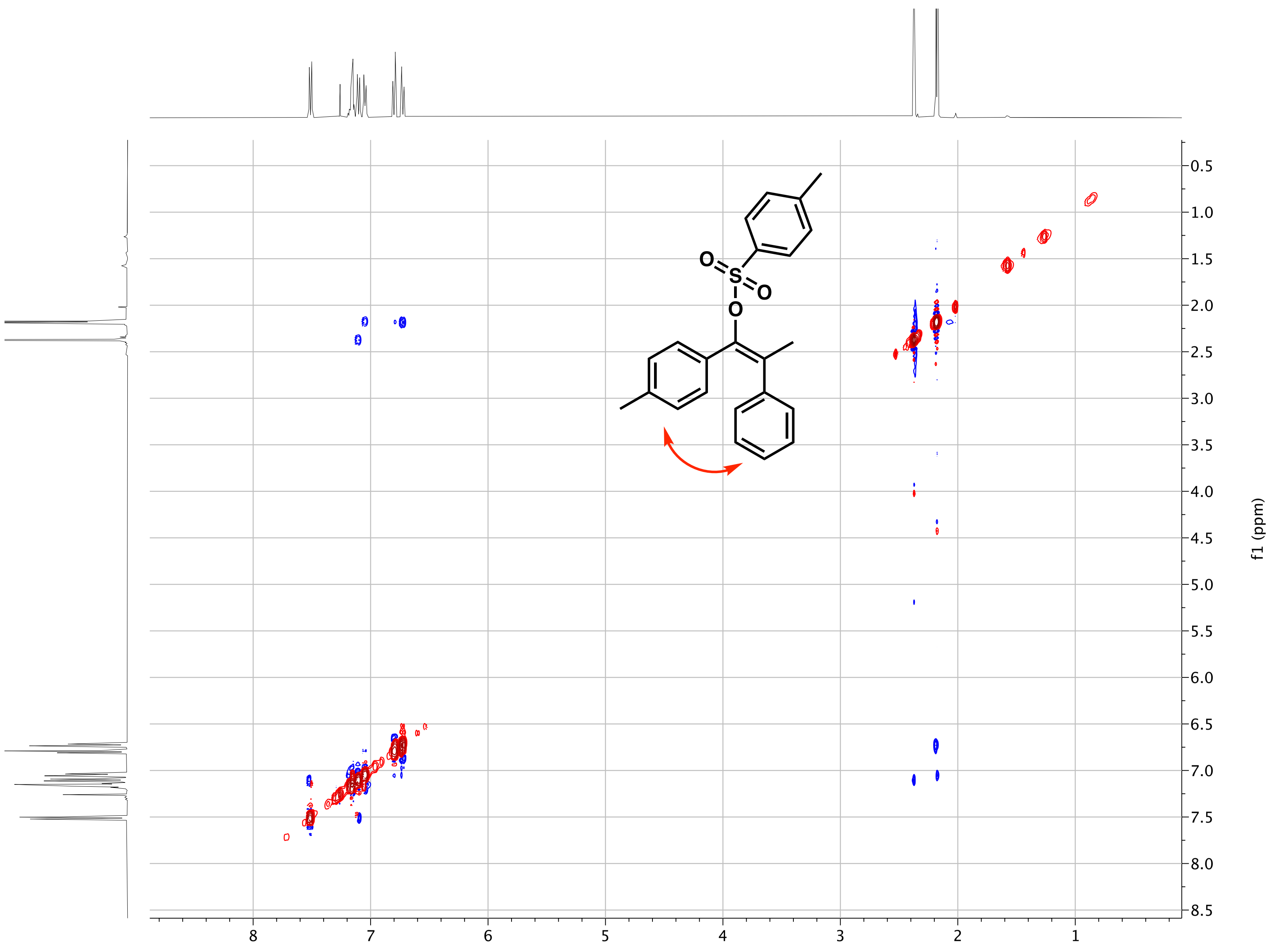

NOESY (400 MHz, $CDCl_3$) of compound **SI-20**.

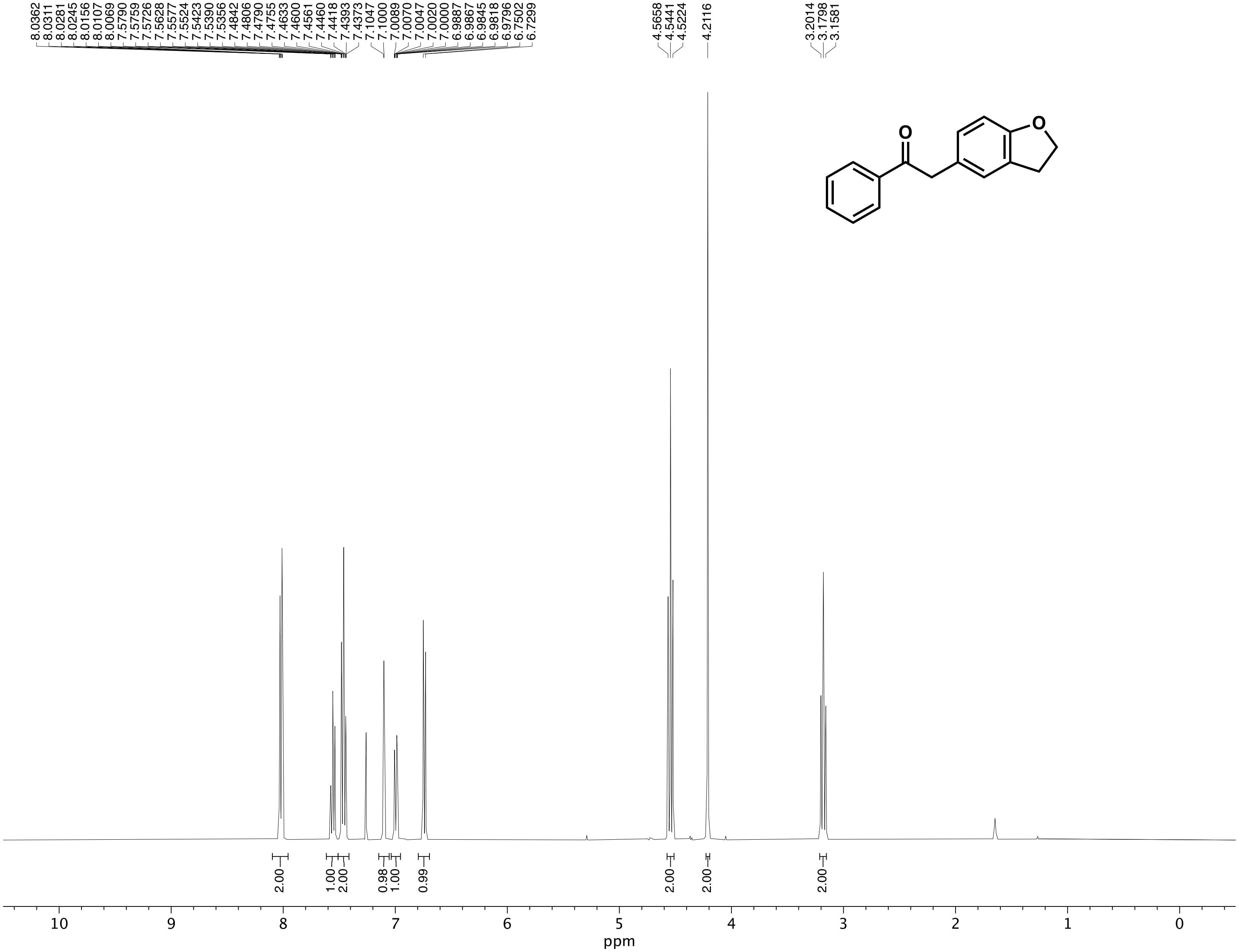

^{1}H NMR (400 MHz, $CDCl_3$) of compound **SI-21**.

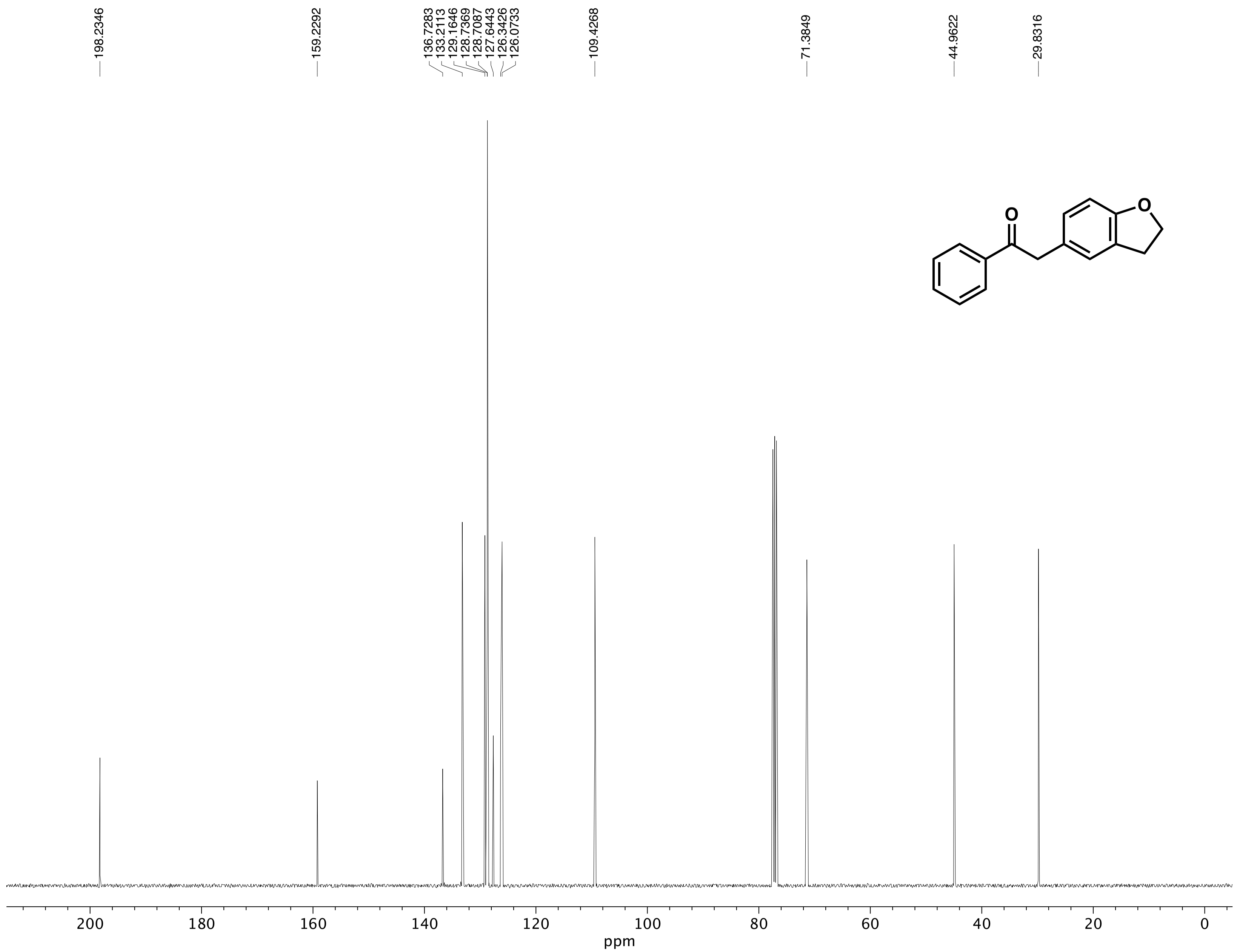

^{13}C NMR (101 MHz, $CDCl_3$) of compound **SI-21**.

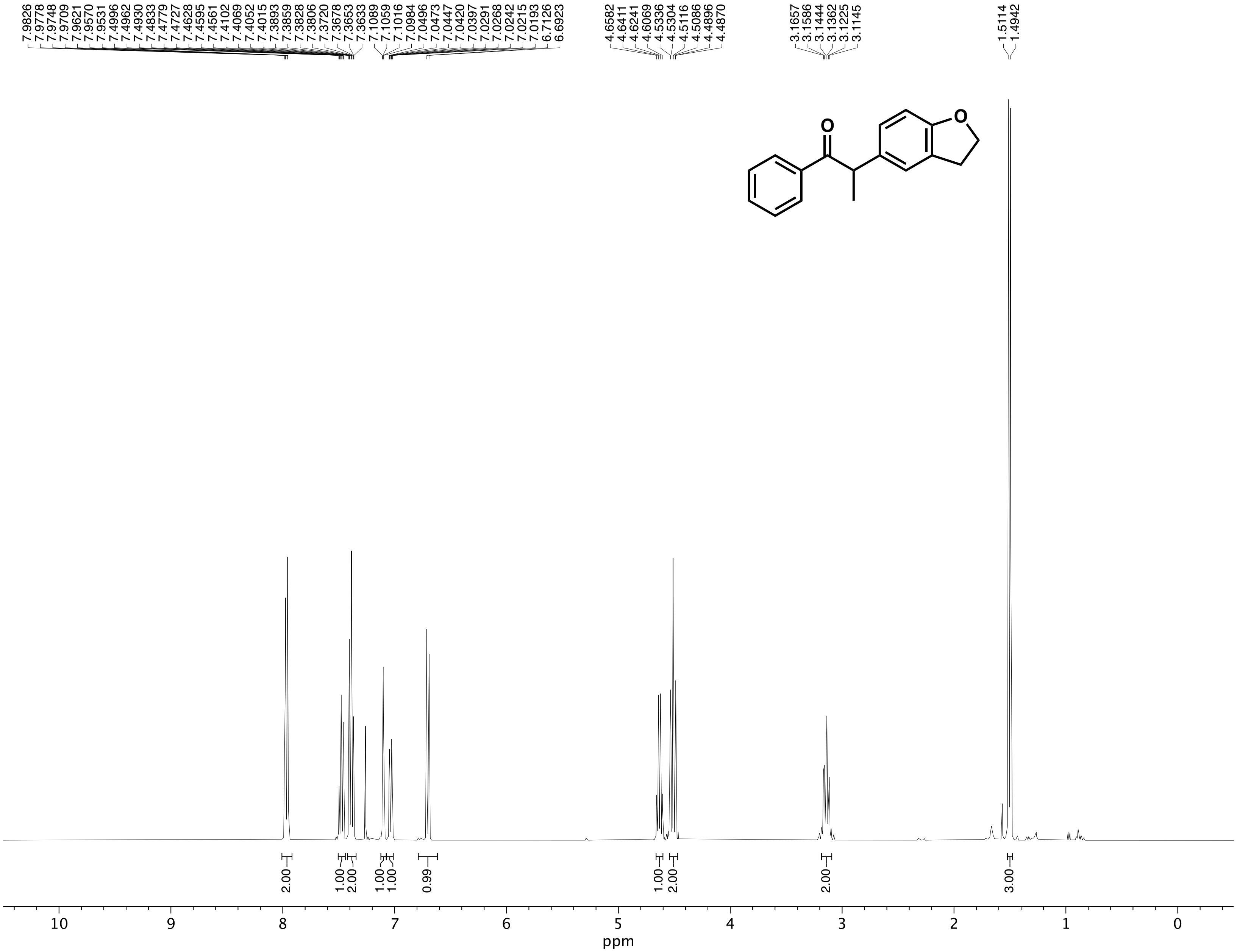

^{1}H NMR (400 MHz, $CDCl_3$) of compound **SI-22**.

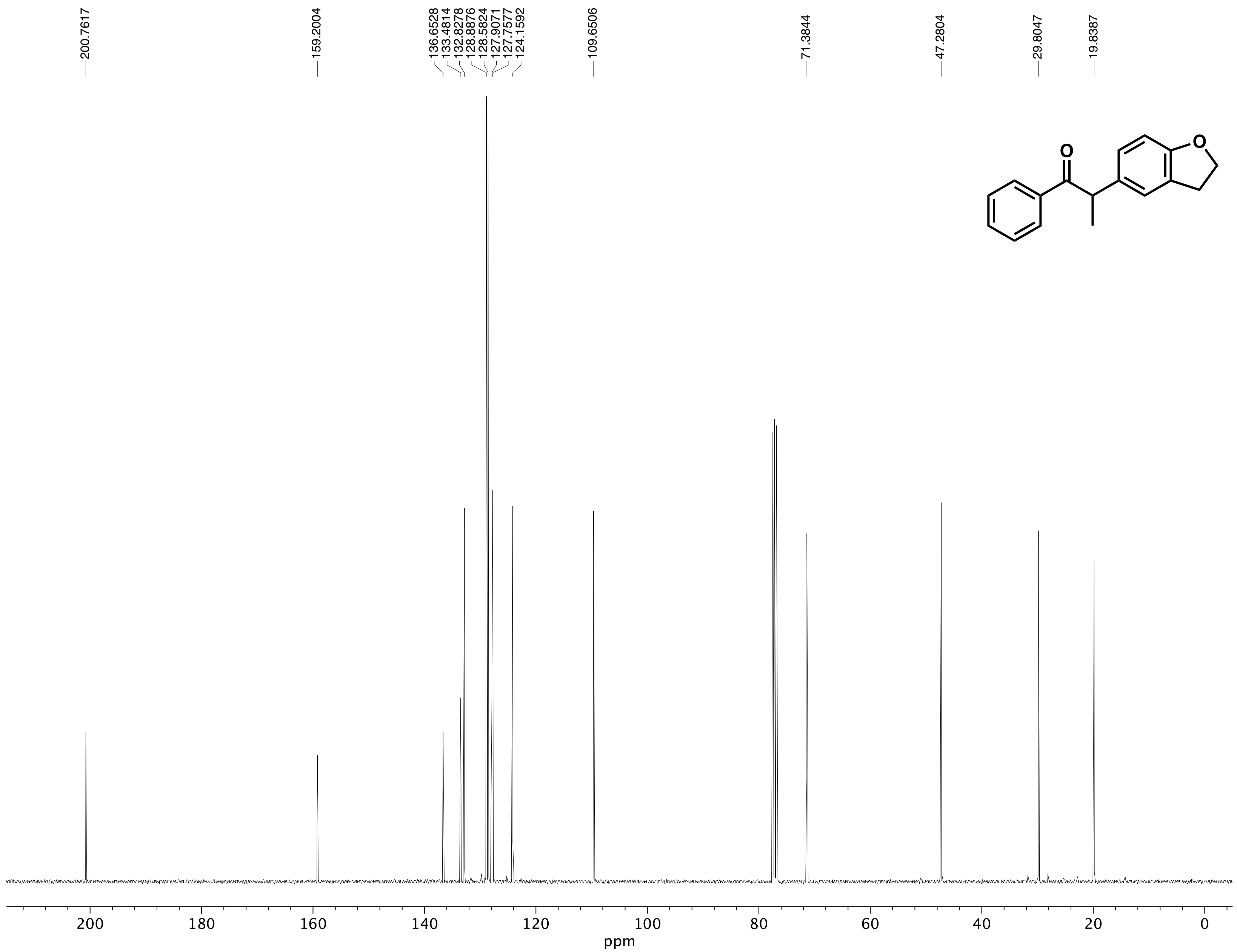

^{13}C NMR (101 MHz, $CDCl_3$) of compound **SI-22**.

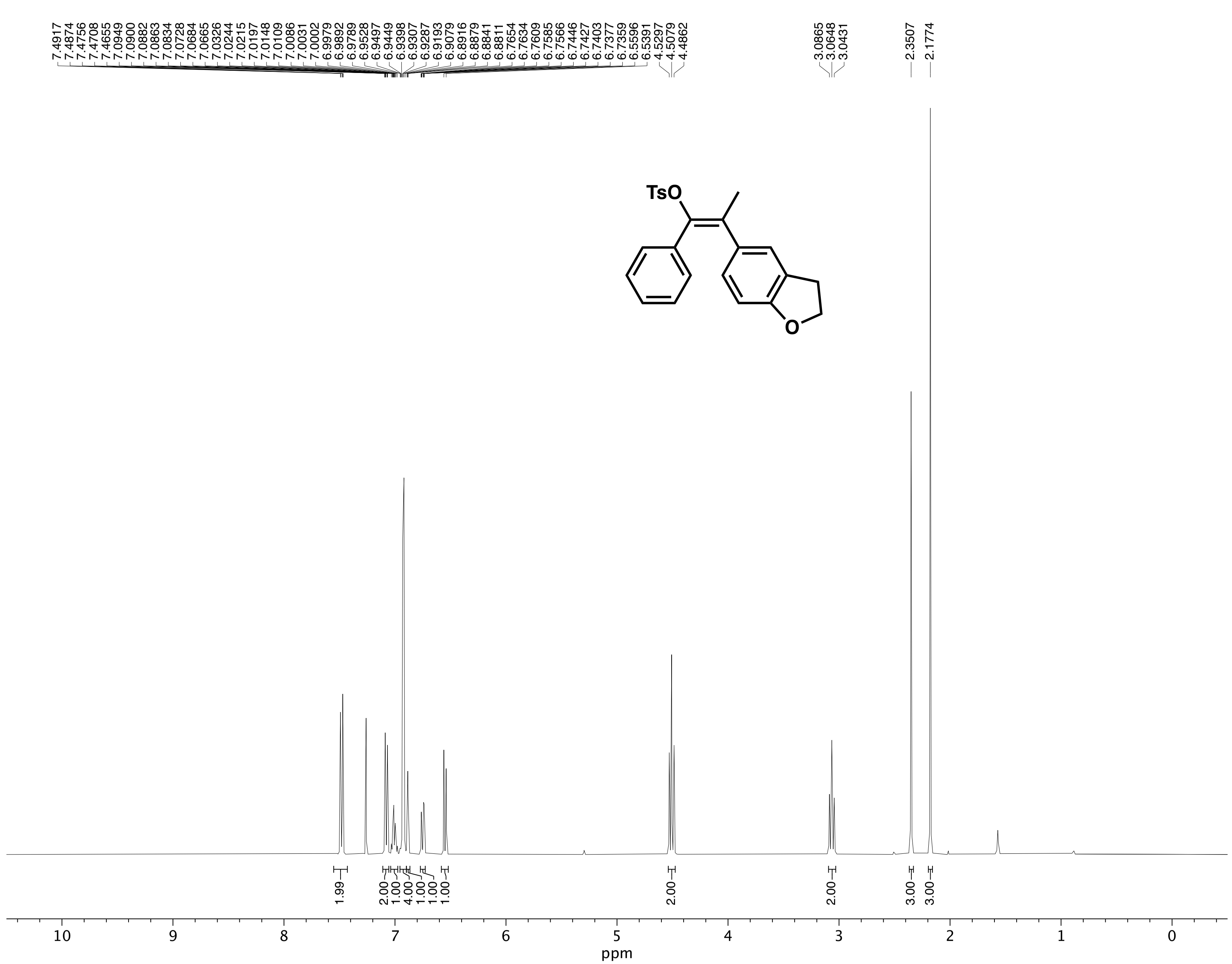

^{1}H NMR (400 MHz, $CDCl_3$) of compound **SI-23**.

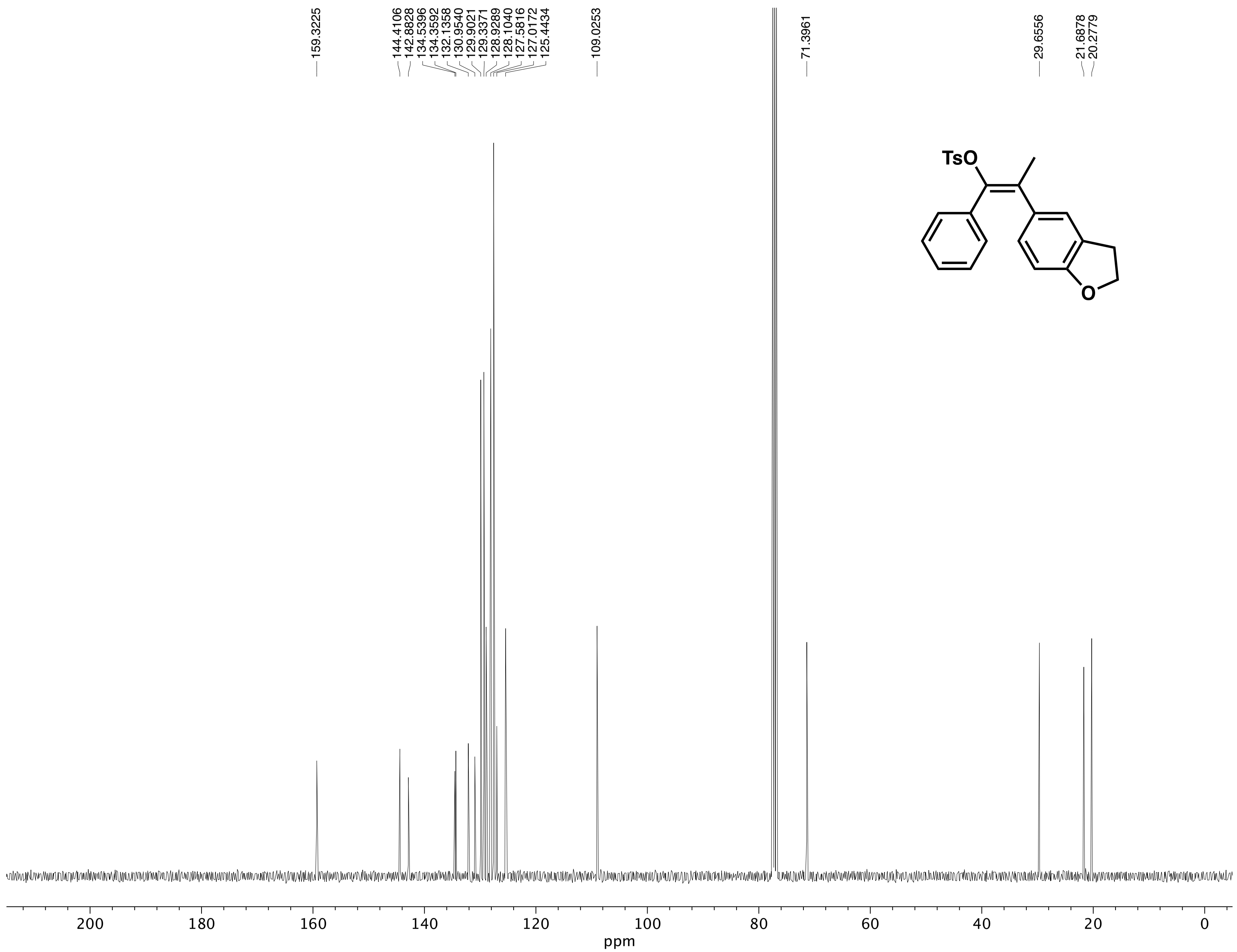

^{13}C NMR (101 MHz, $CDCl_3$) of compound **SI-23**.

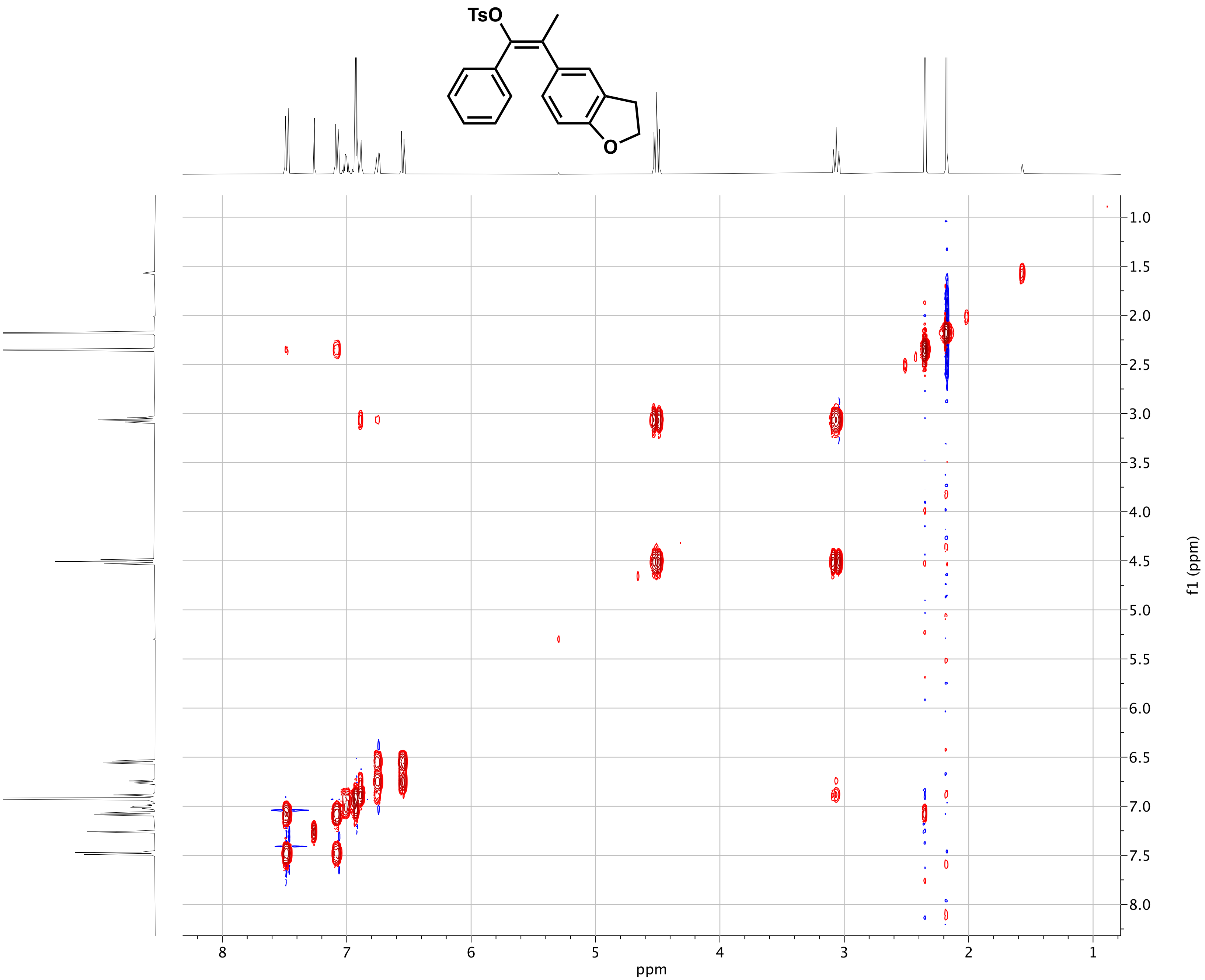

COSEY (400 MHz, $CDCl_3$) of compound **SI-23**.

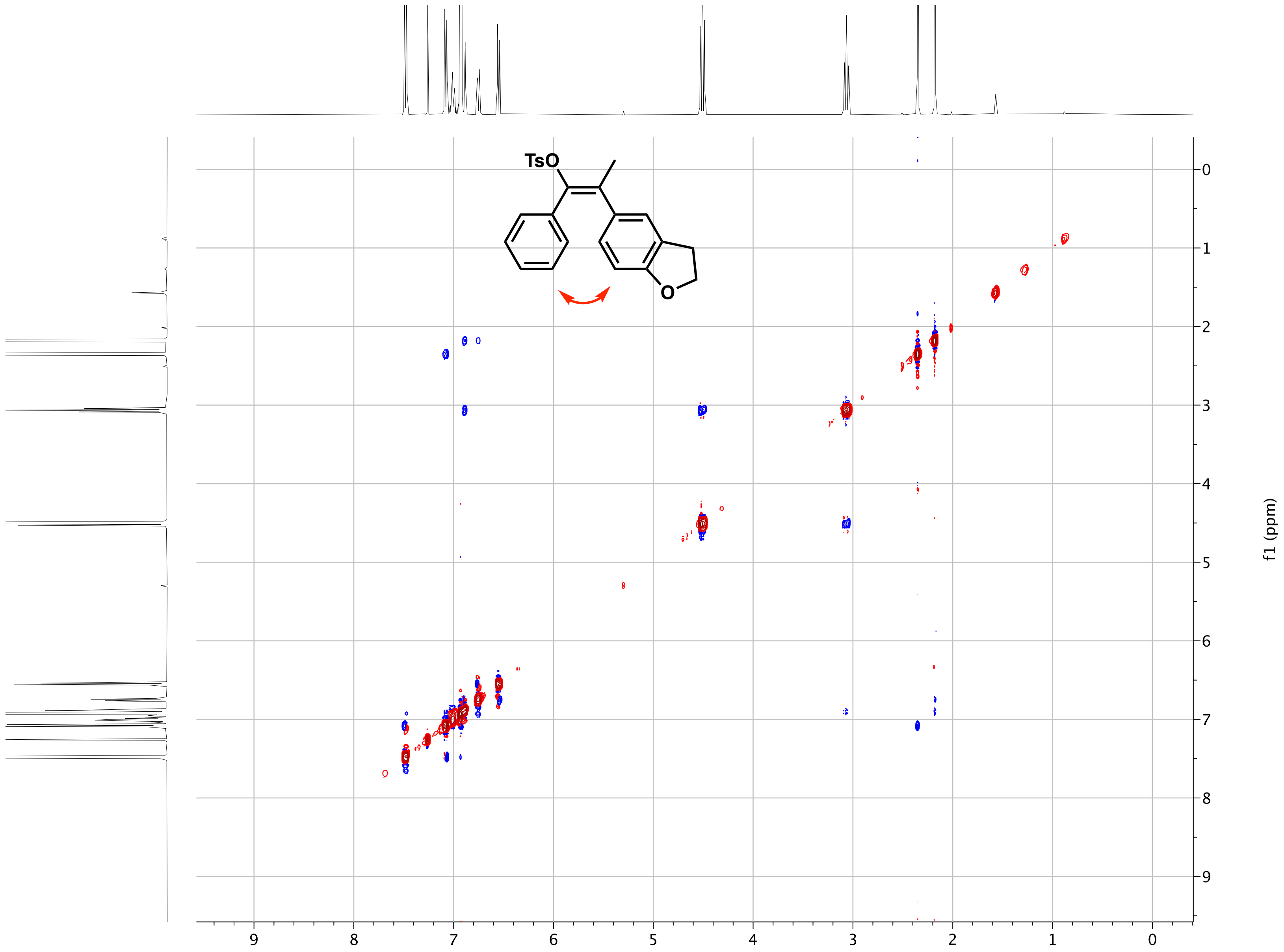

NOESY (400 MHz, $CDCl_3$) of compound **SI-23**.

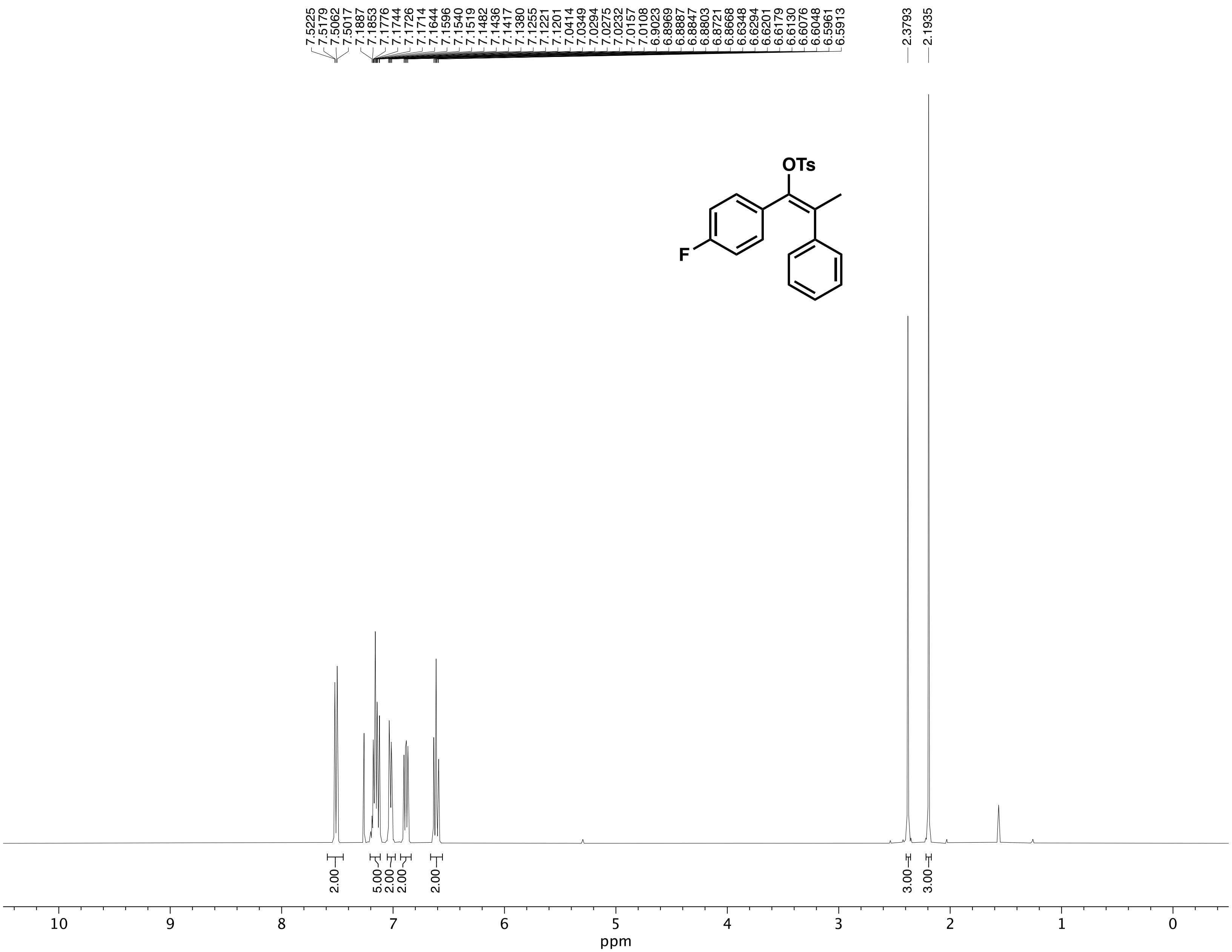

^{1}H NMR (400 MHz, $CDCl_3$) of compound **SI-26**.

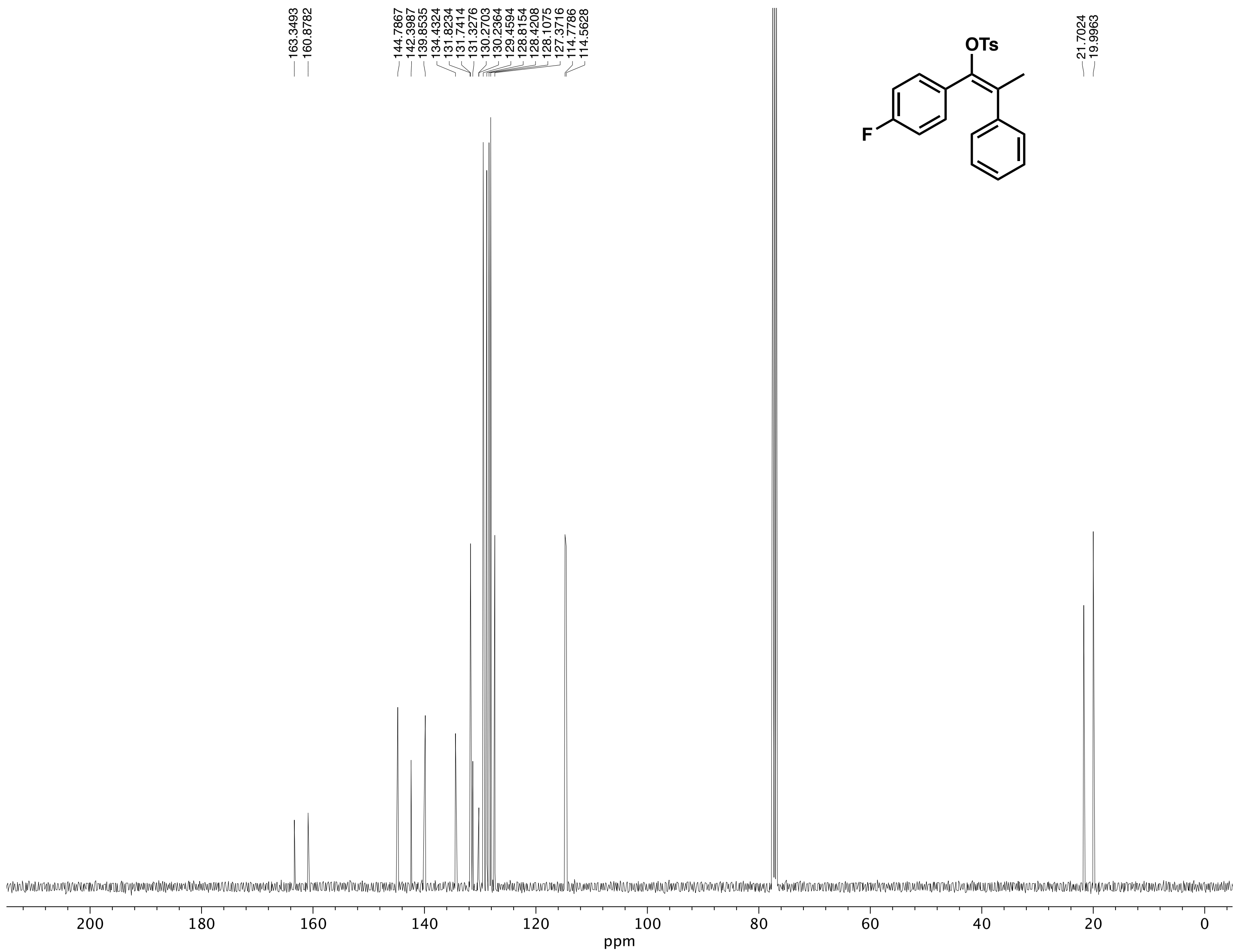

^{13}C NMR (101 MHz, $CDCl_3$) of compound **SI-26**.

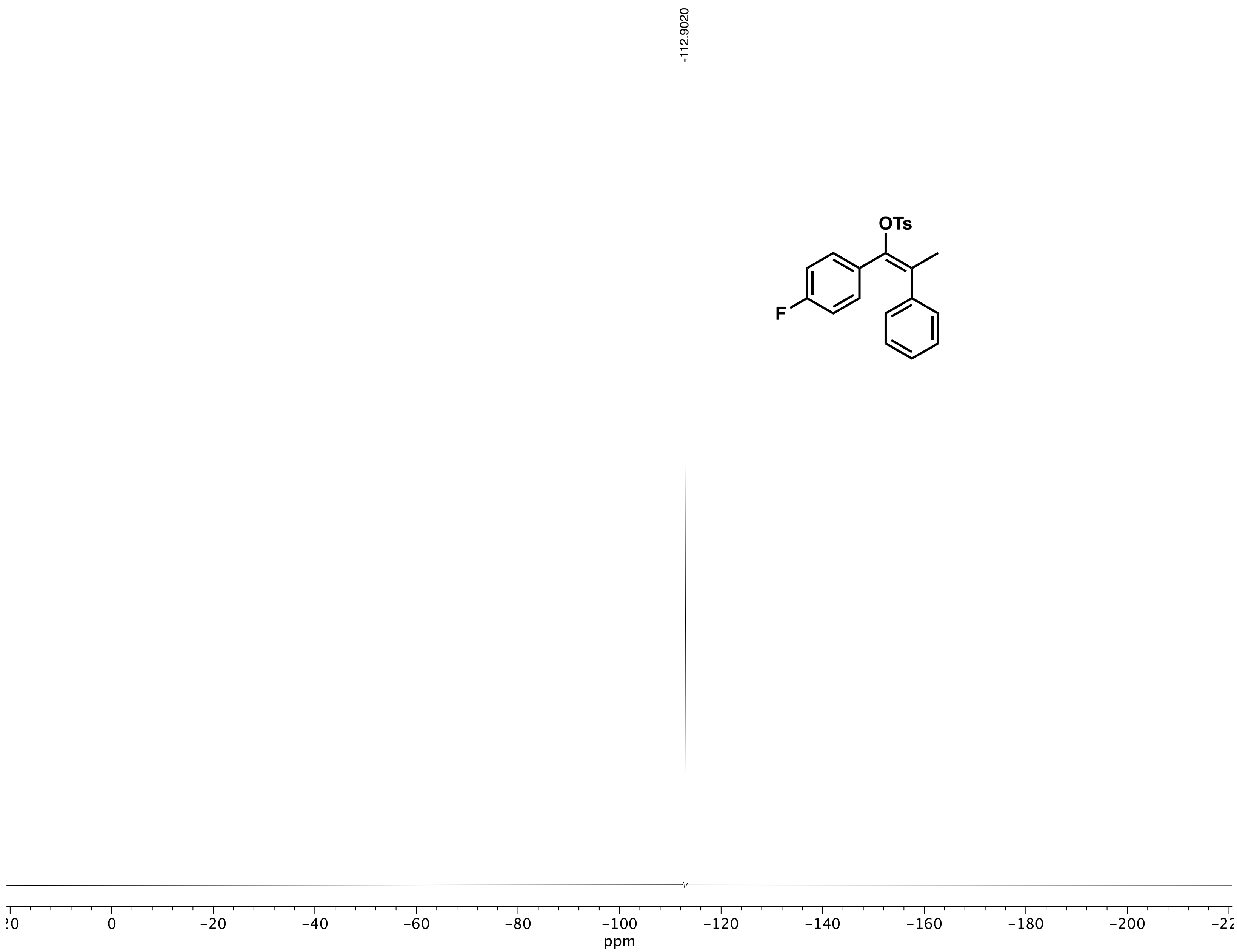

^{19}F NMR (376 MHz, $CDCl_3$) of compound **SI-26.**

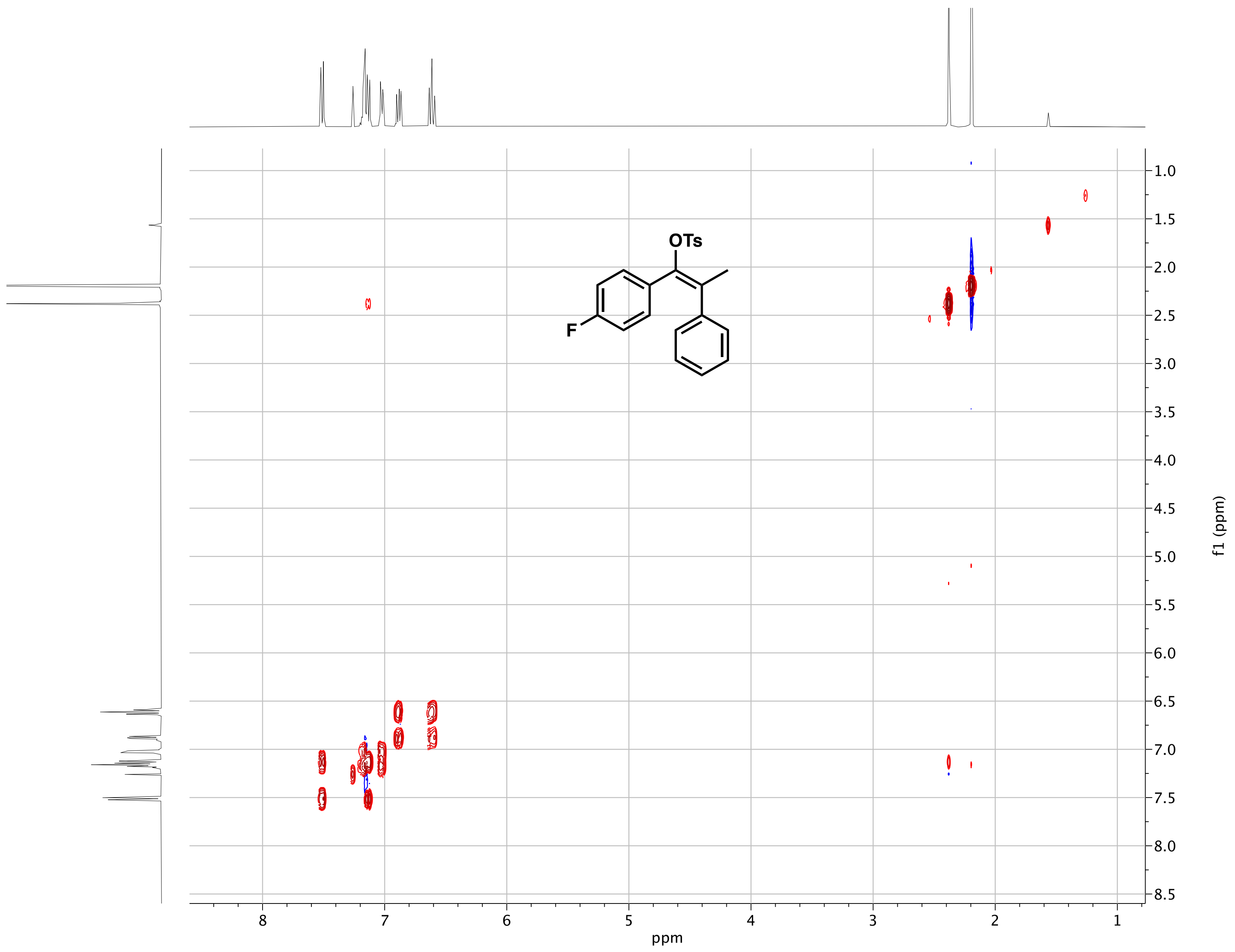

COSEY (400 MHz, $CDCl_3$) of compound **SI-26**.

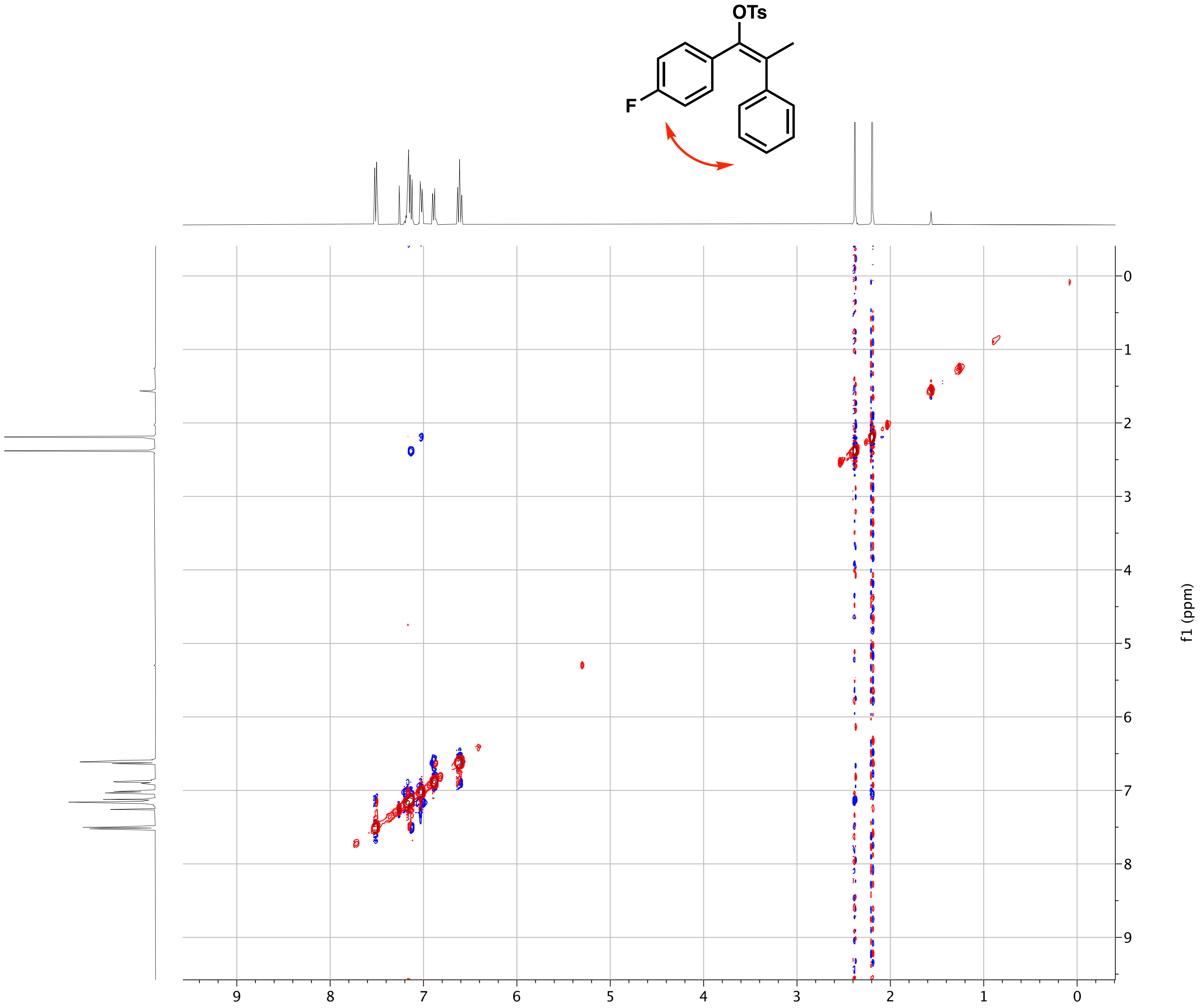

NOESY (400 MHz, $CDCl_3$) of compound **SI-26**.

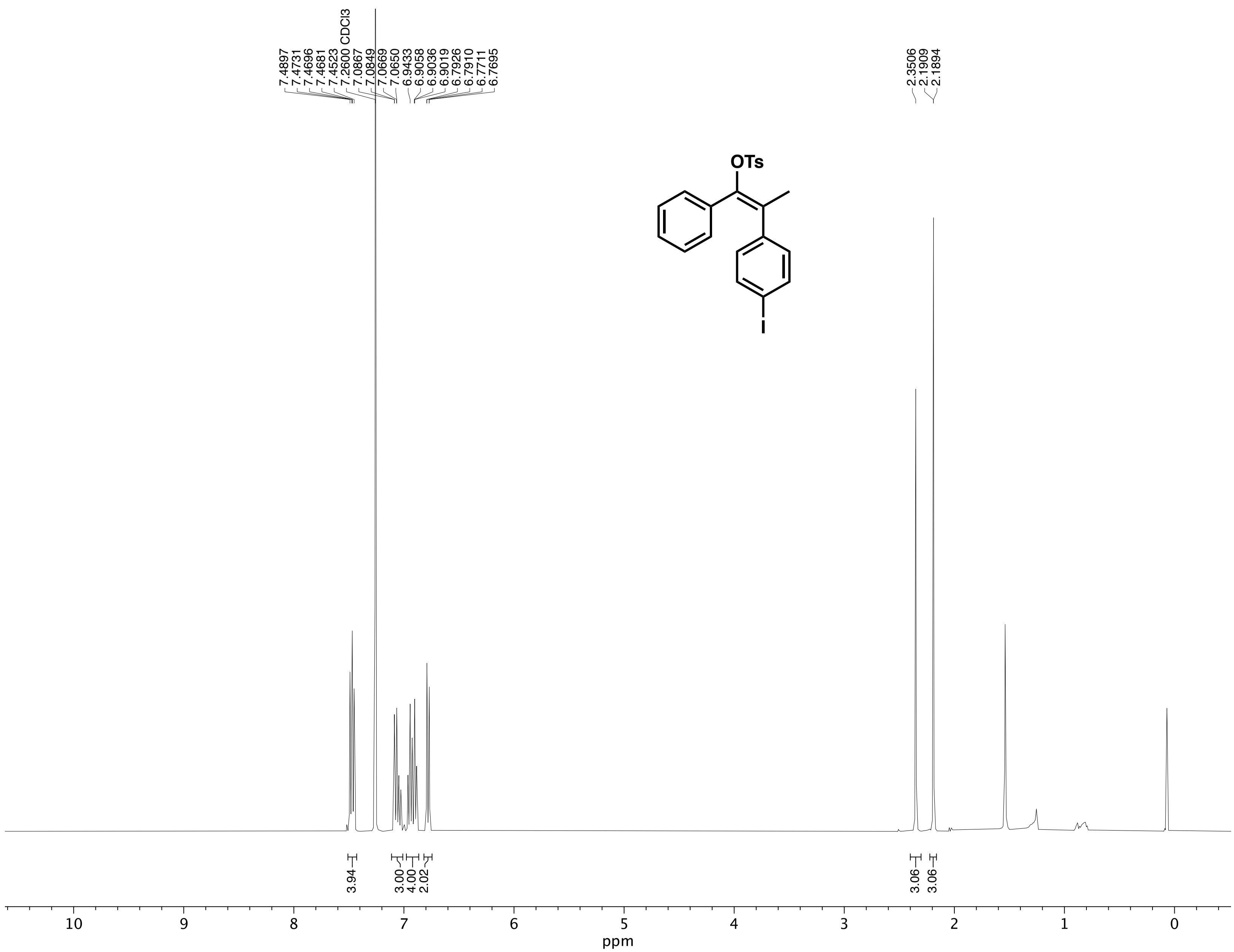

^1H NMR (400 MHz, $CDCl_3$) of compound **SI-30**.

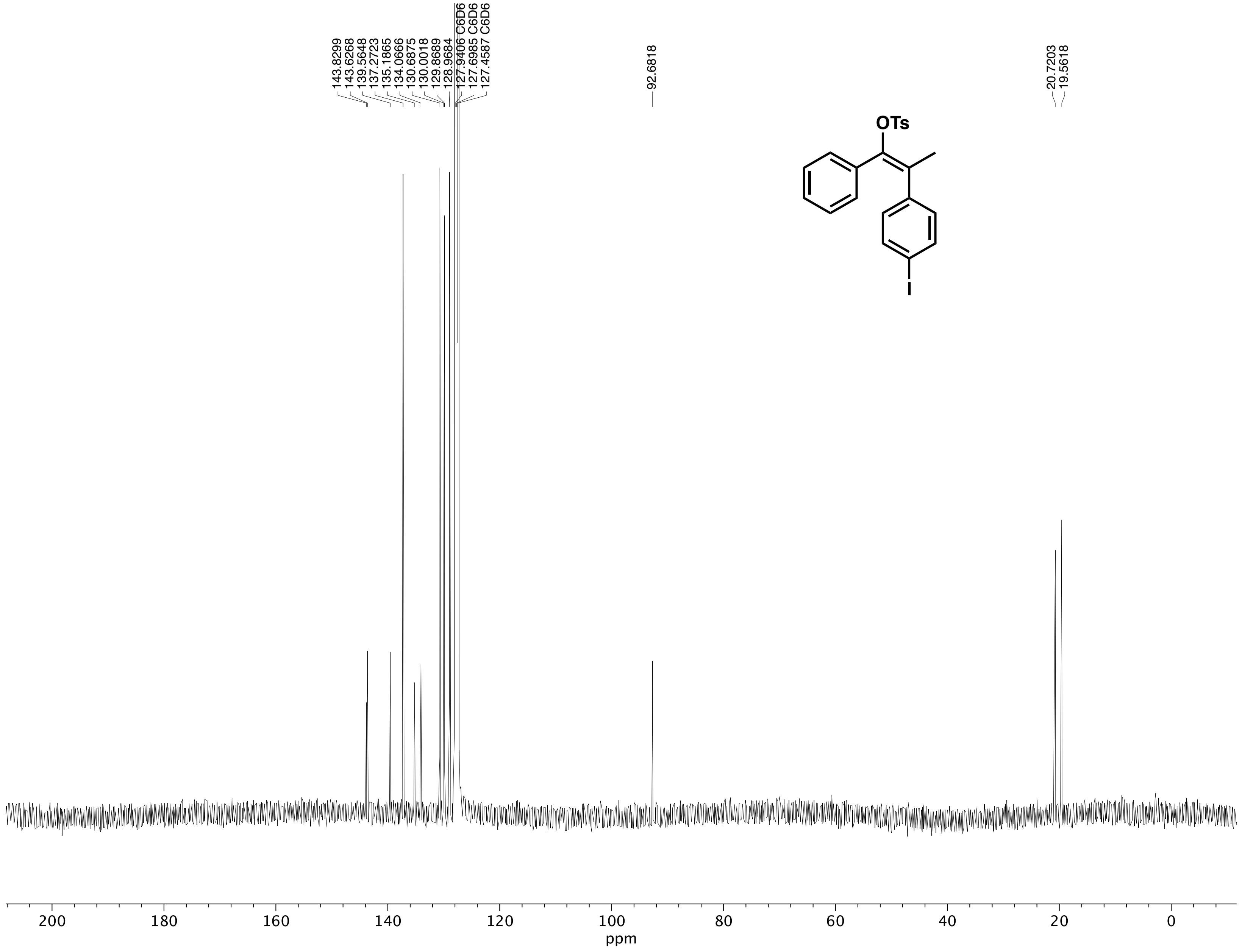

^{13}C NMR (101 MHz, $CDCl_3$) of compound **SI-30**.

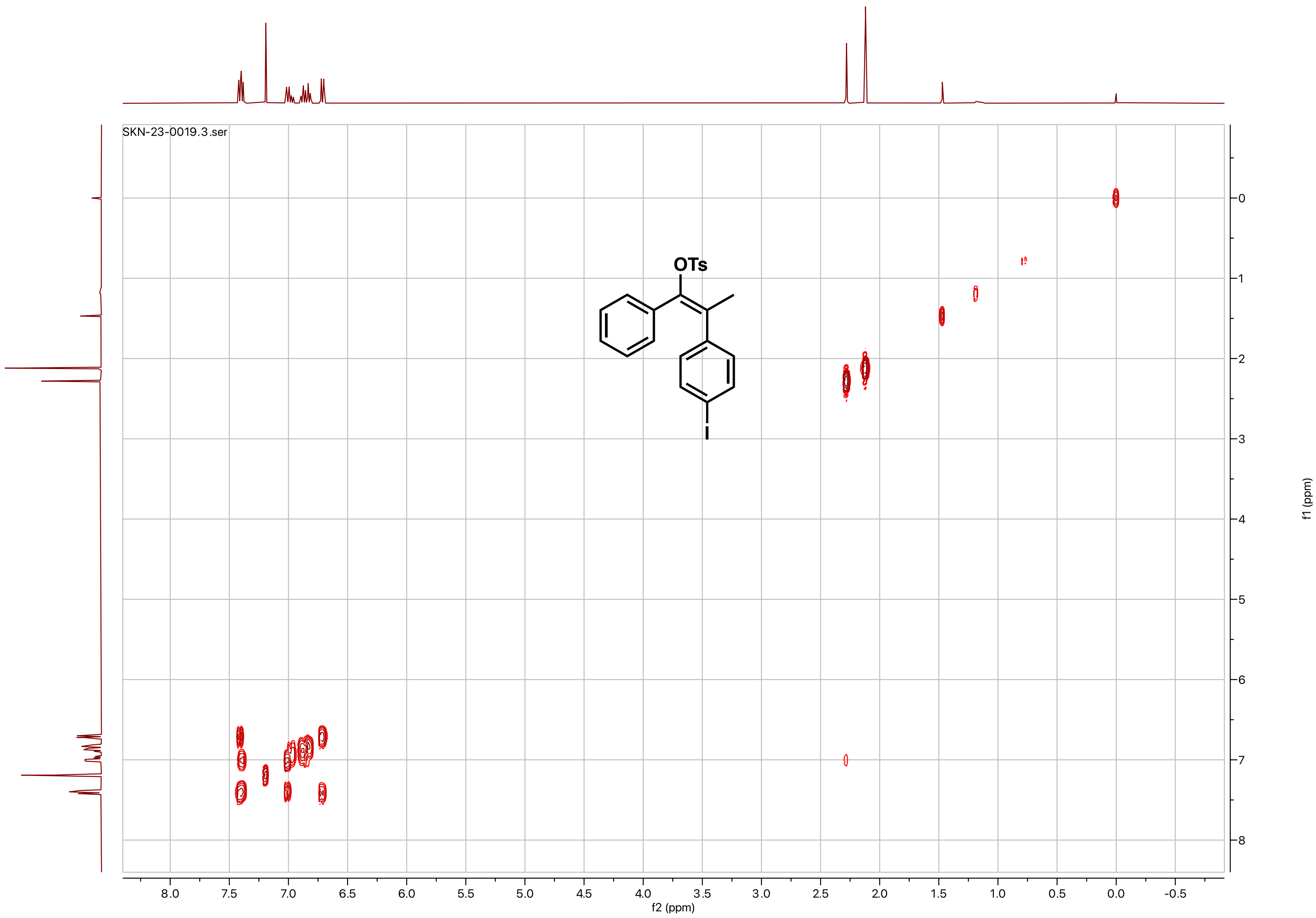

COSEY (400 MHz, $CDCl_3$) of compound **SI-30**.

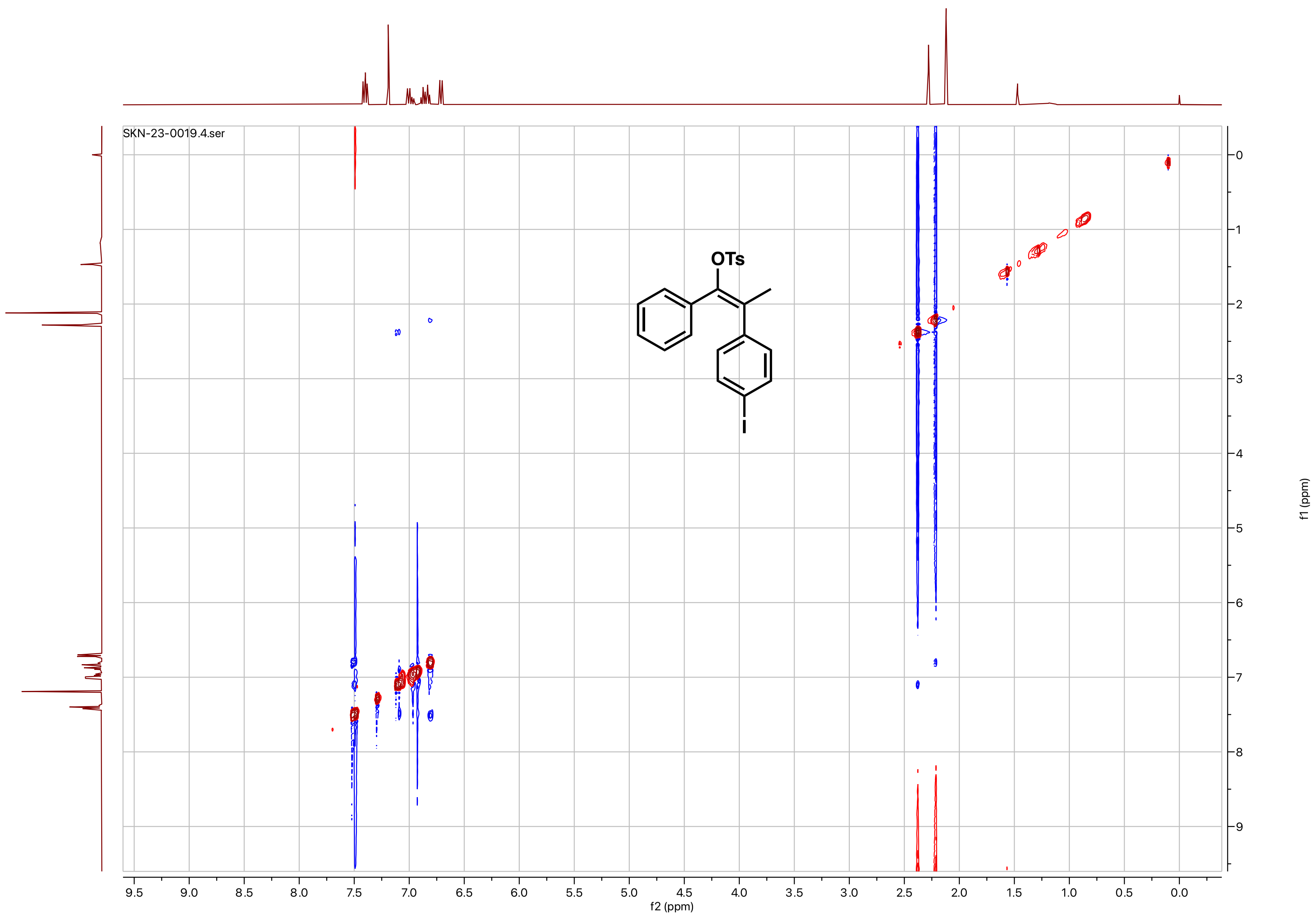

NOESY (400 MHz, $CDCl_3$) of compound **SI-30**.

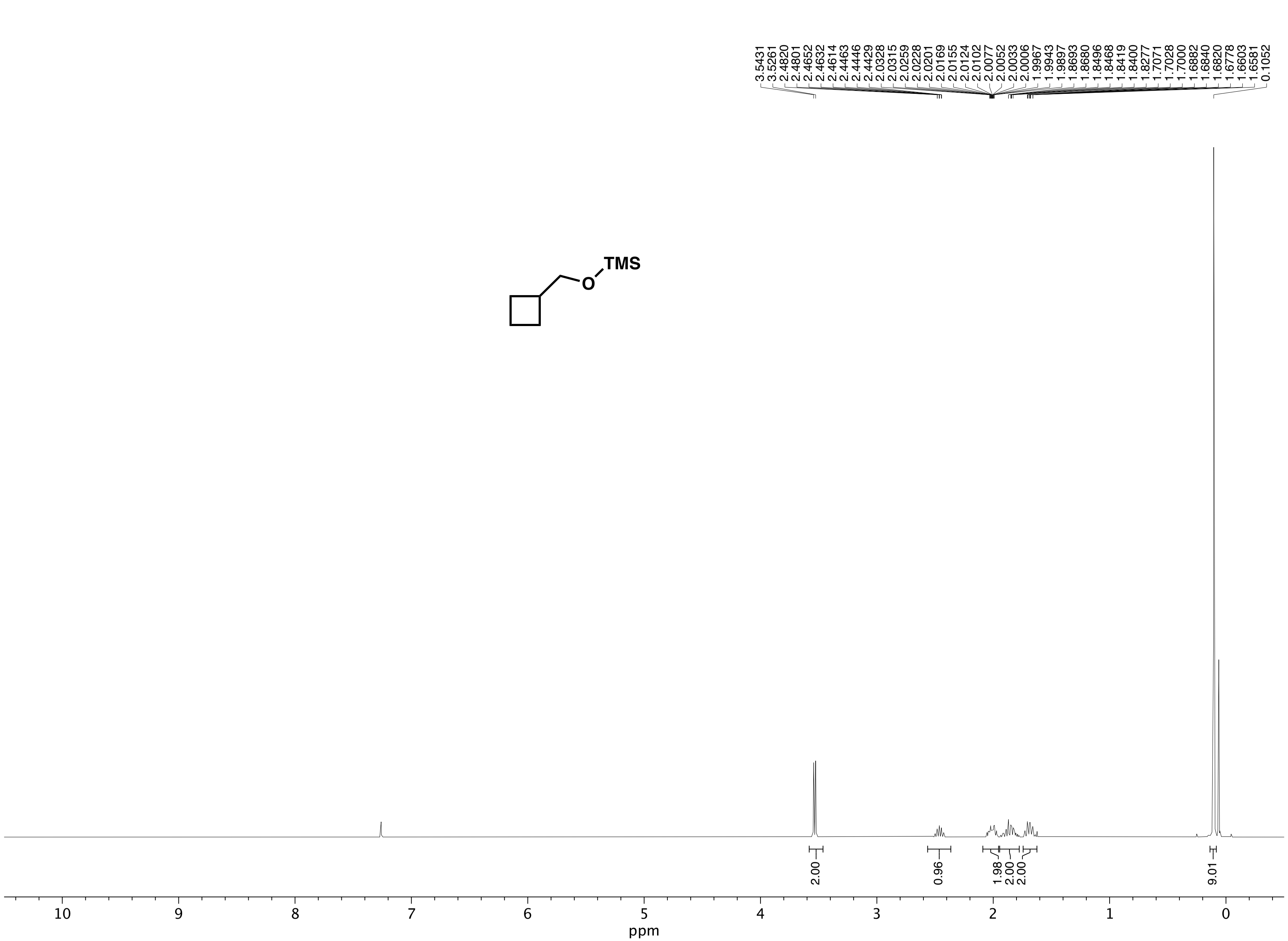

^{1}H NMR (400 MHz, $CDCl_3$) of **SI-35**.

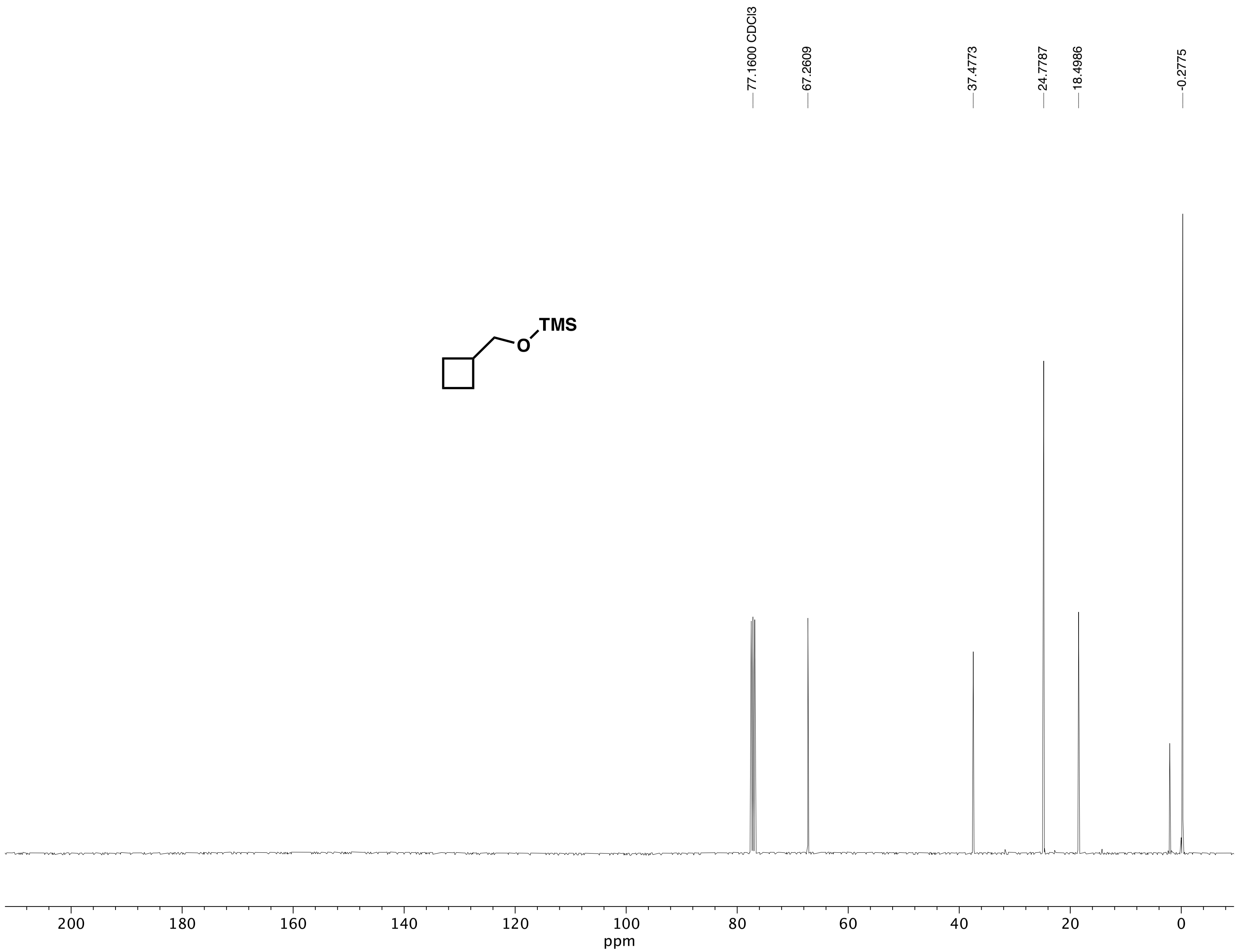

^{13}C NMR (101 MHz, $CDCl_3$) of **SI-35**.

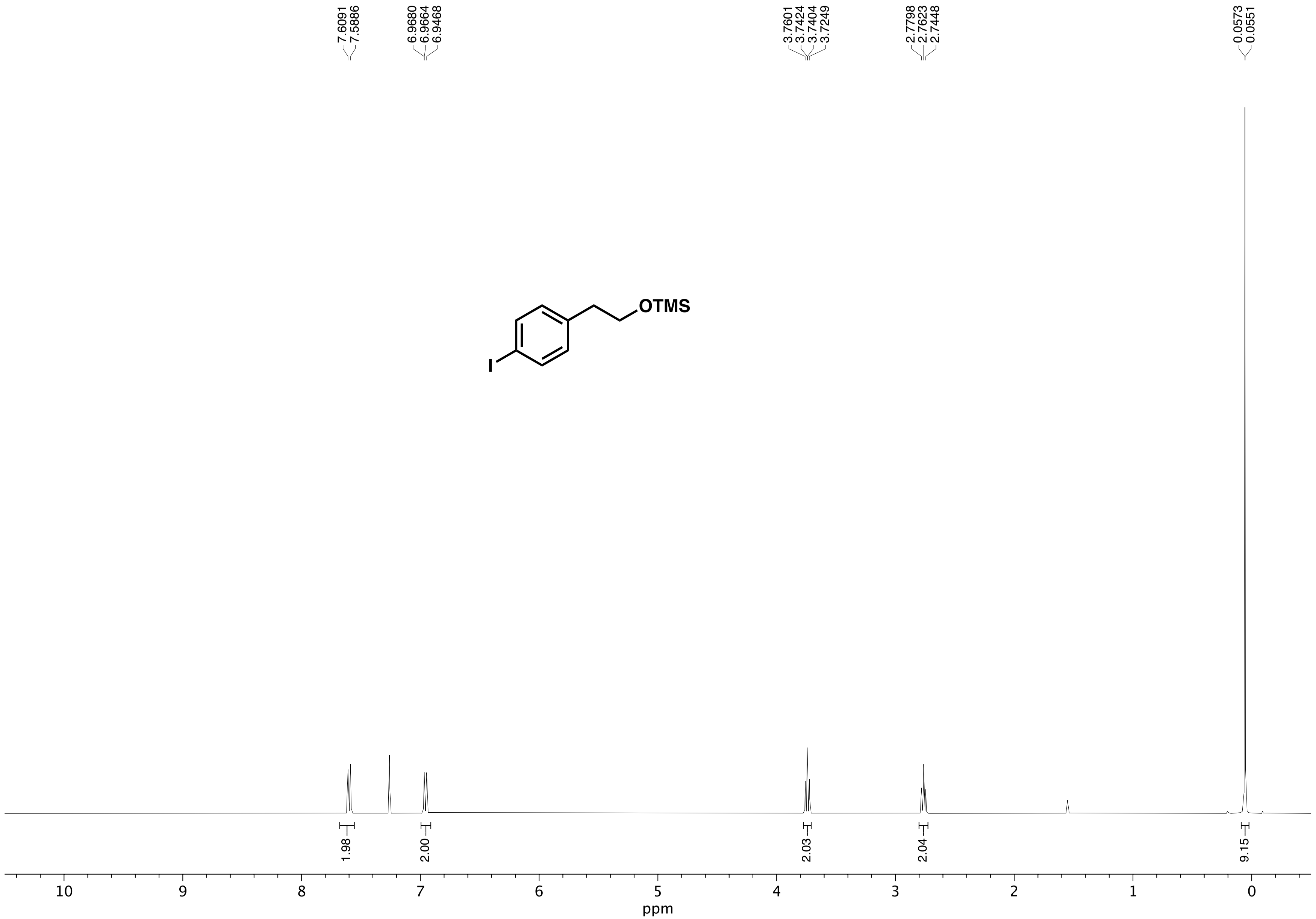

^{1}H NMR (400 MHz, $CDCl_3$) of **SI-36**.

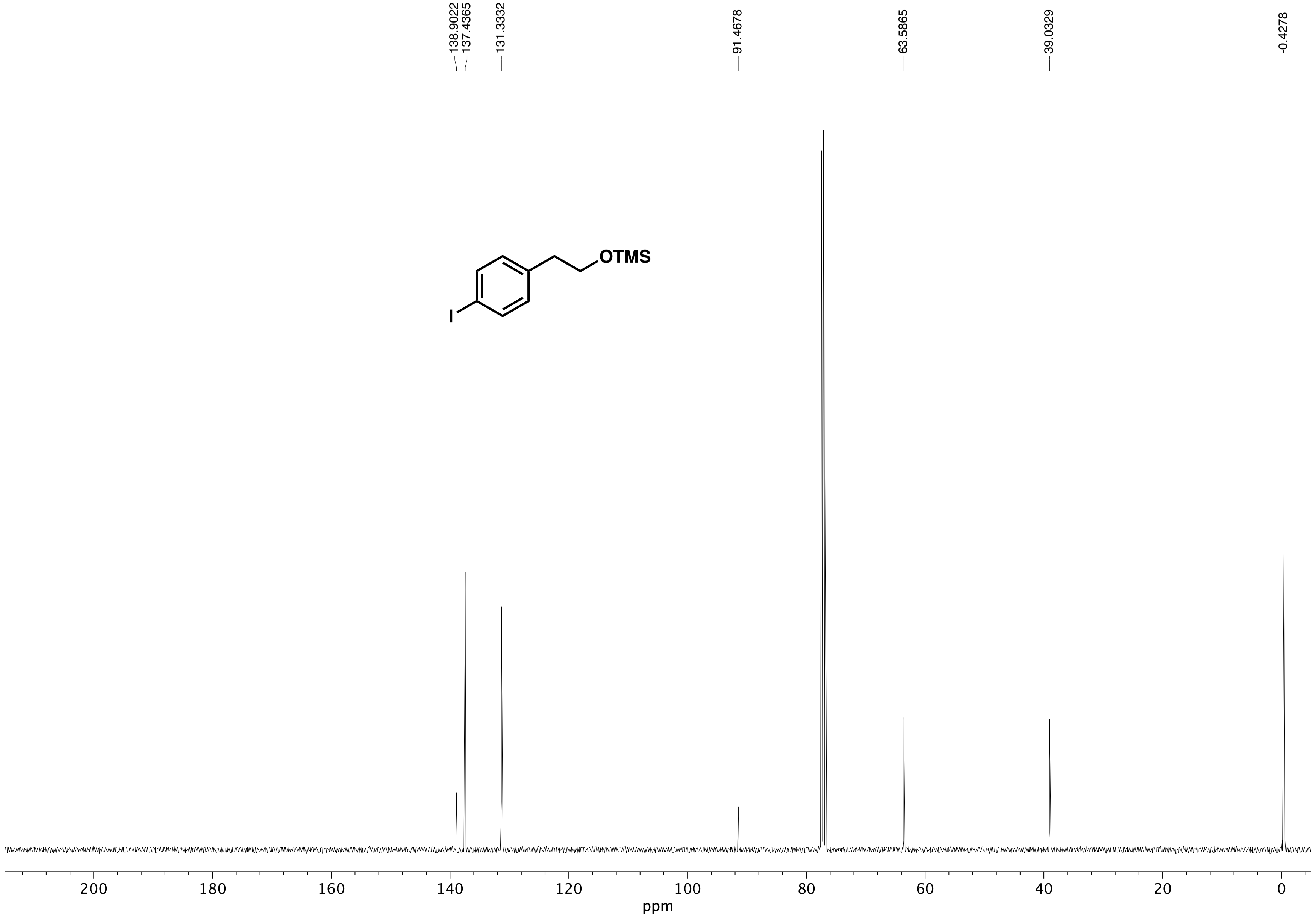

^{13}C NMR (101 MHz, $CDCl_3$) of **SI-36**.

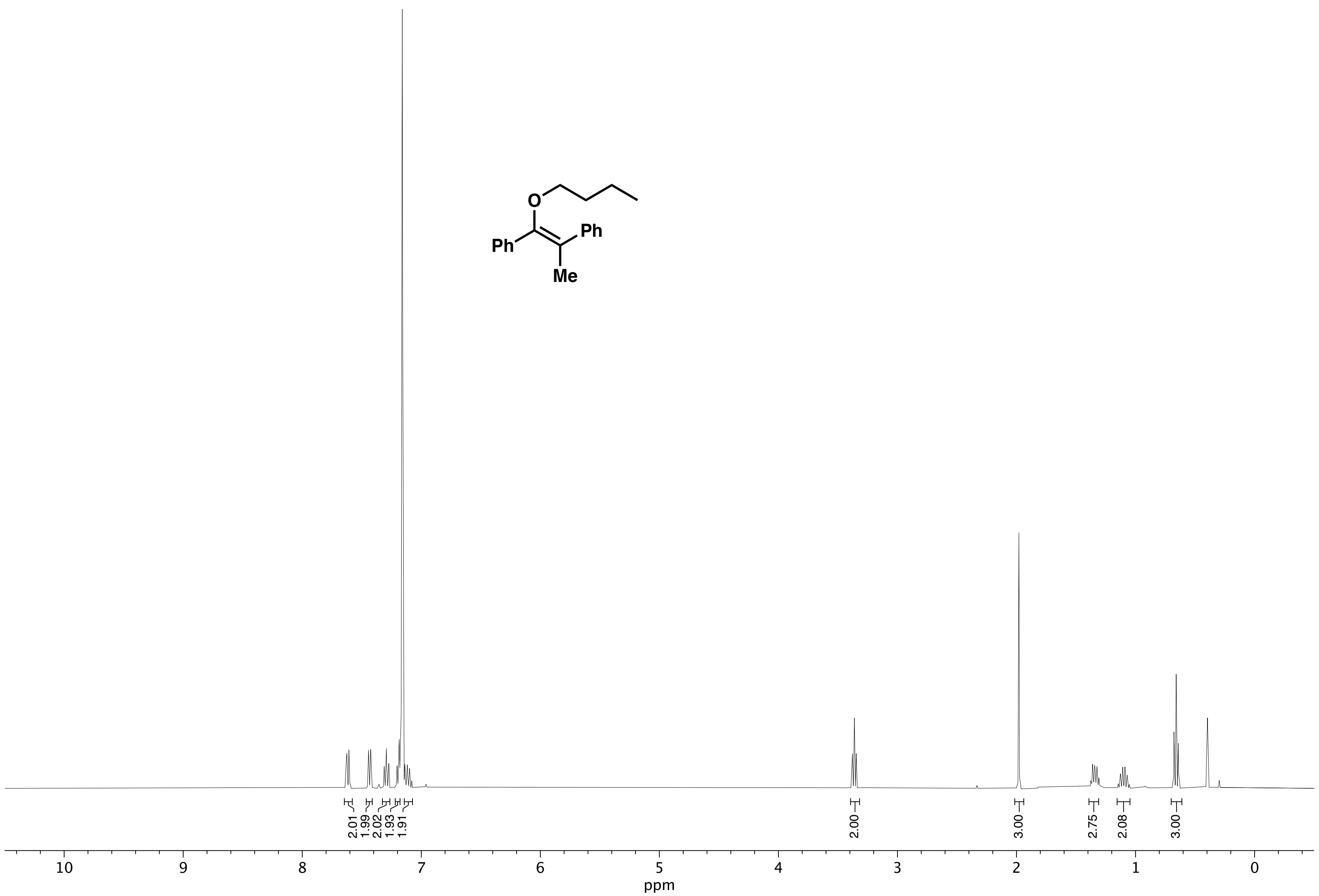

^{1}H NMR (400 MHz, C_6D_6) of **SI-39**.

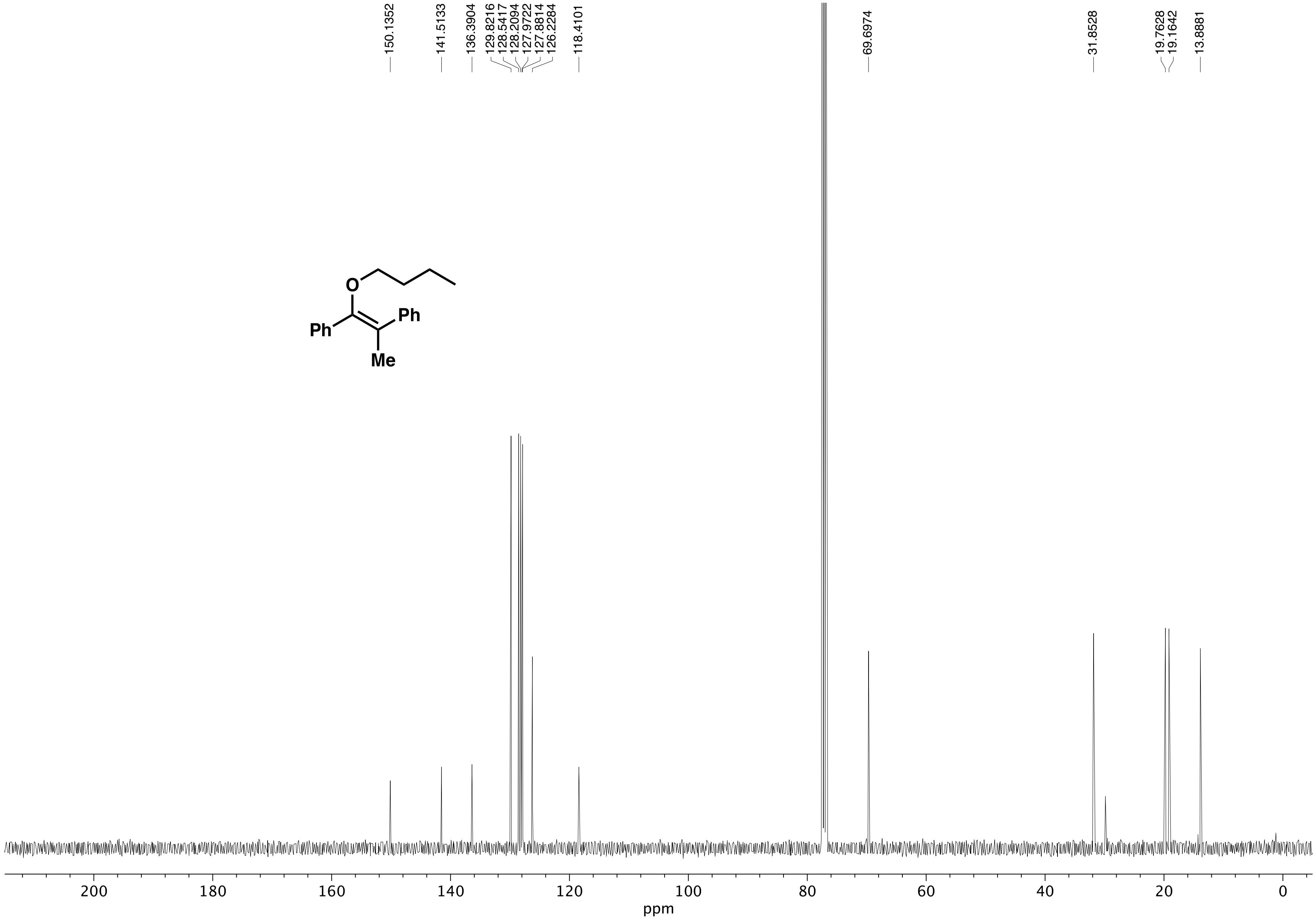

^{13}C NMR (101 MHz, $CDCl_3$) of **SI-39.**

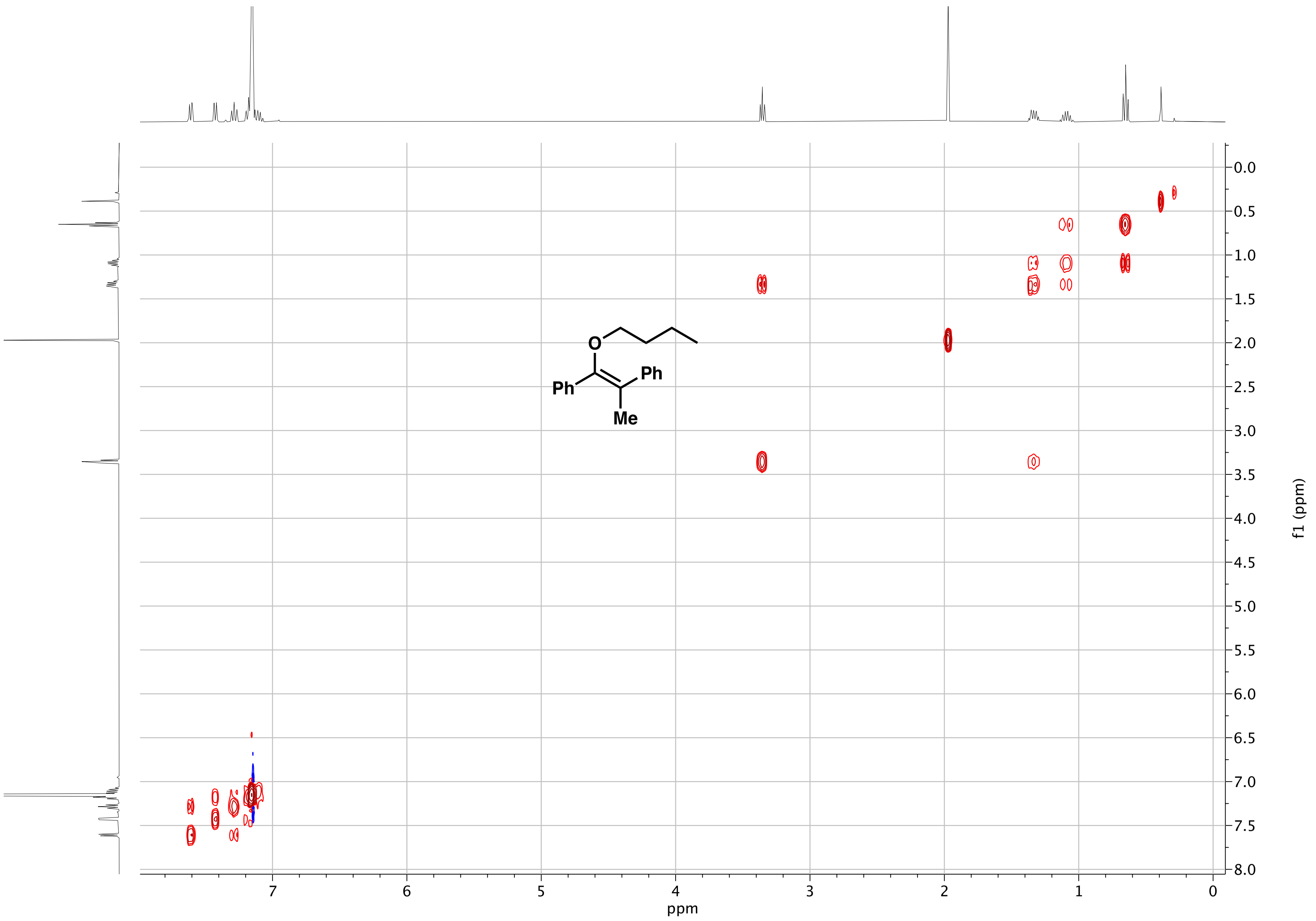

COSY NMR (400 MHz, C_6D_6) of **SI-39**.

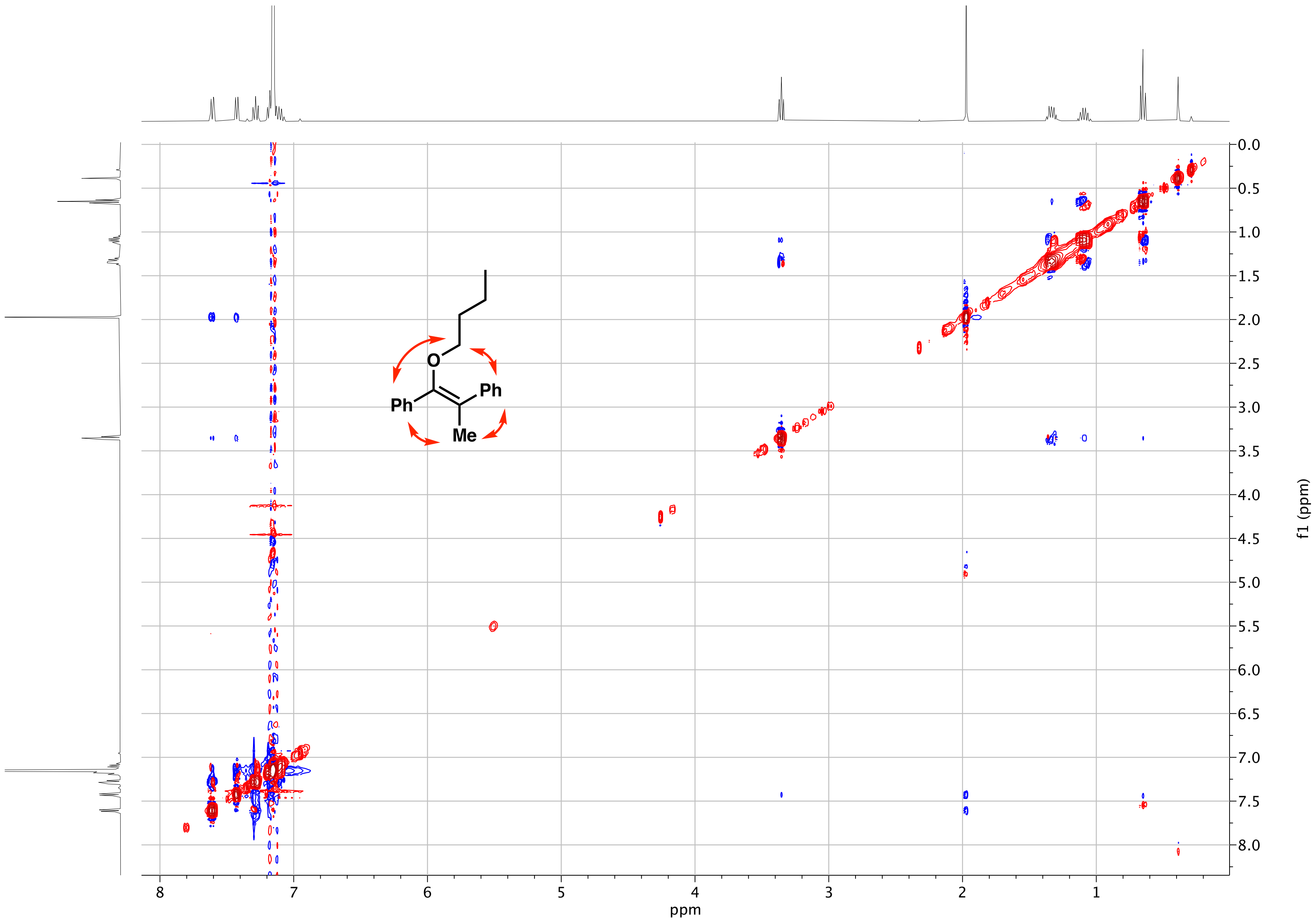

NOESY NMR (400 MHz, C_6D_6) of **SI-39**.

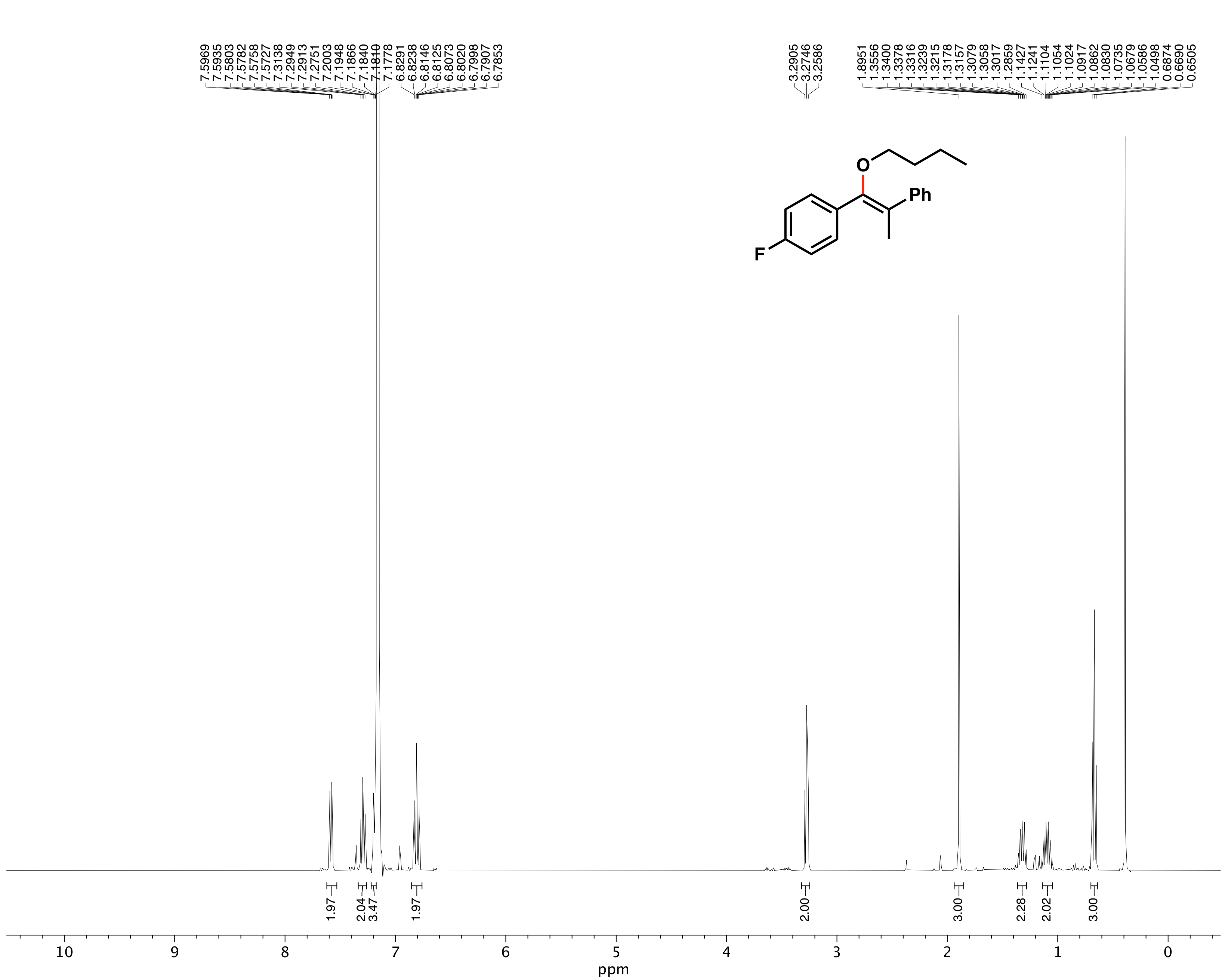

^{1}H NMR (400 MHz, C_6D_6) of compound **SI-40**.

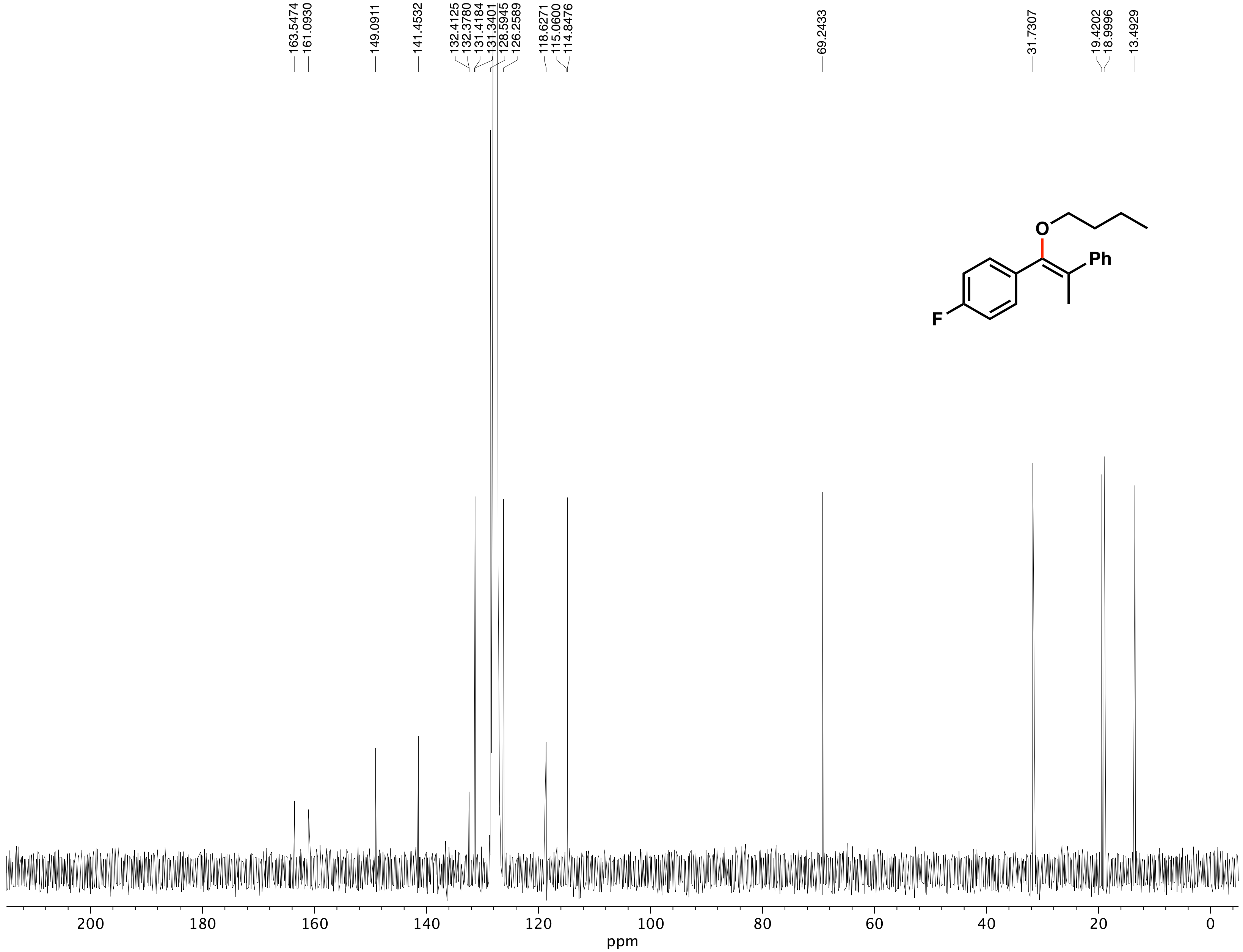

^{13}C NMR (101 MHz, , C_6D_6) of compound **SI-40**.

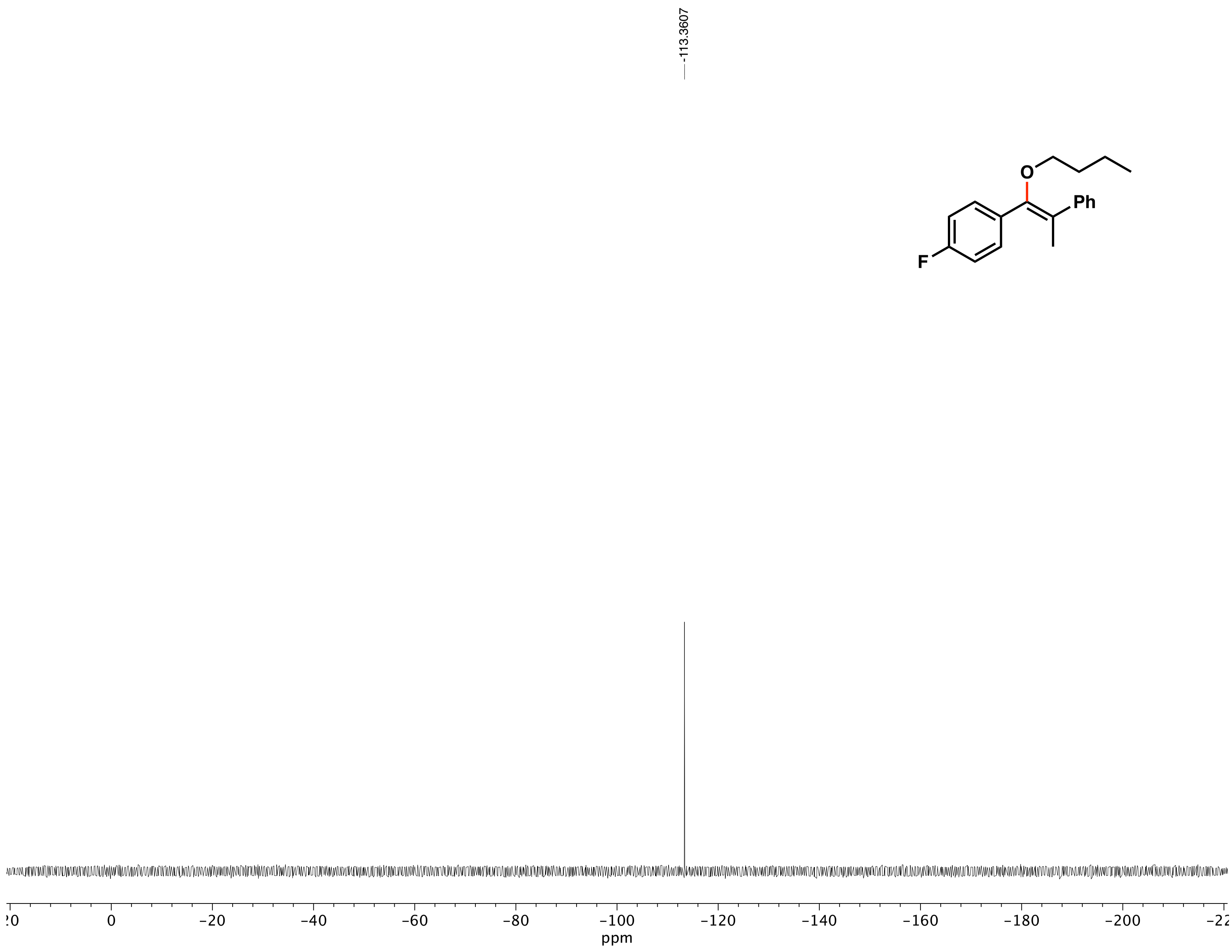

^{19}F NMR (376 MHz, , C_6D_6) of compound **SI-40**.

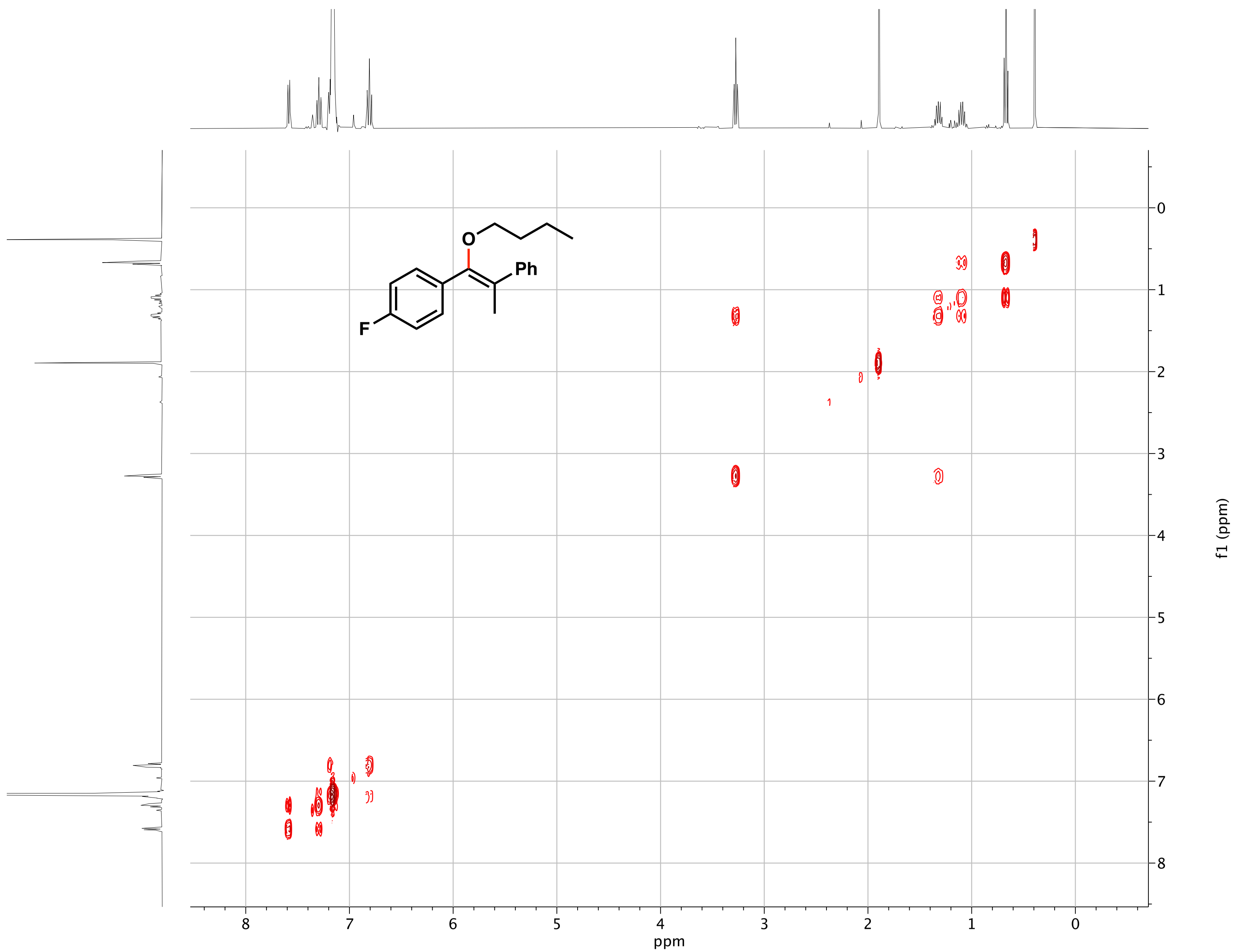

COSY (400 MHz, C_6D_6) of compound **SI-40**.

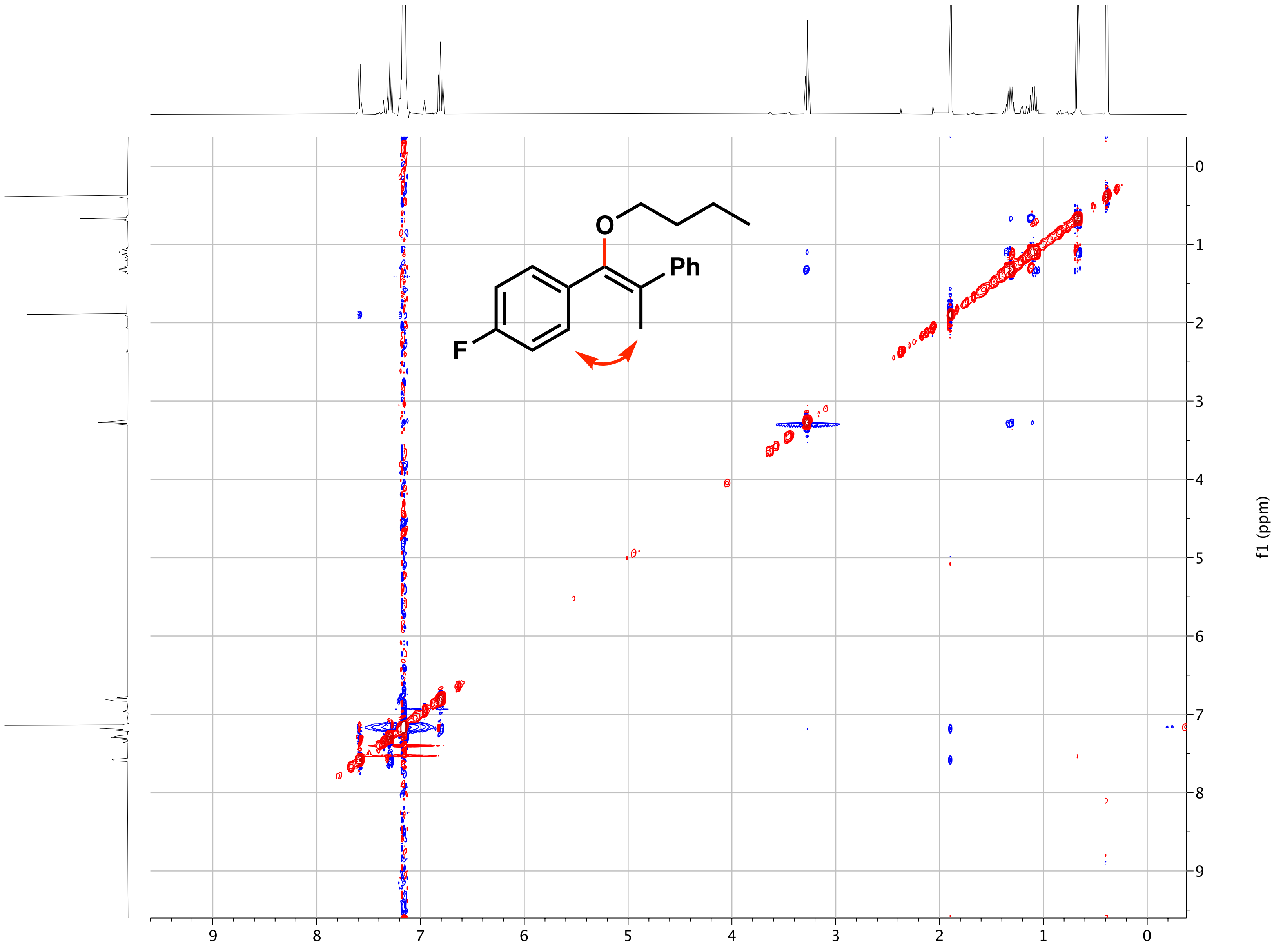

NOSEY (400 MHz, C_6D_6) of compound **SI-40**.

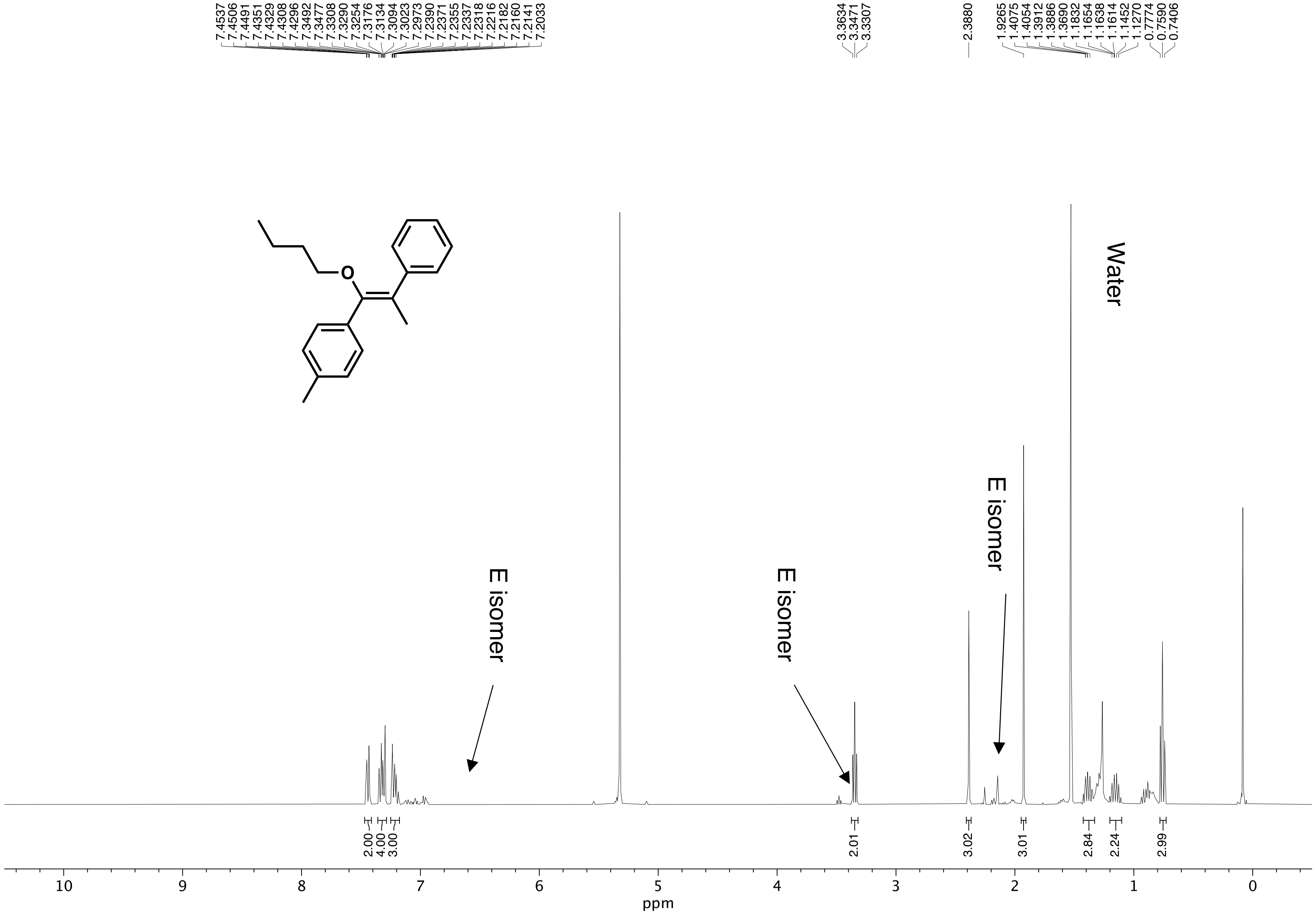

^{1}H NMR (400 MHz, d_2-DCM) of **SI-41**.

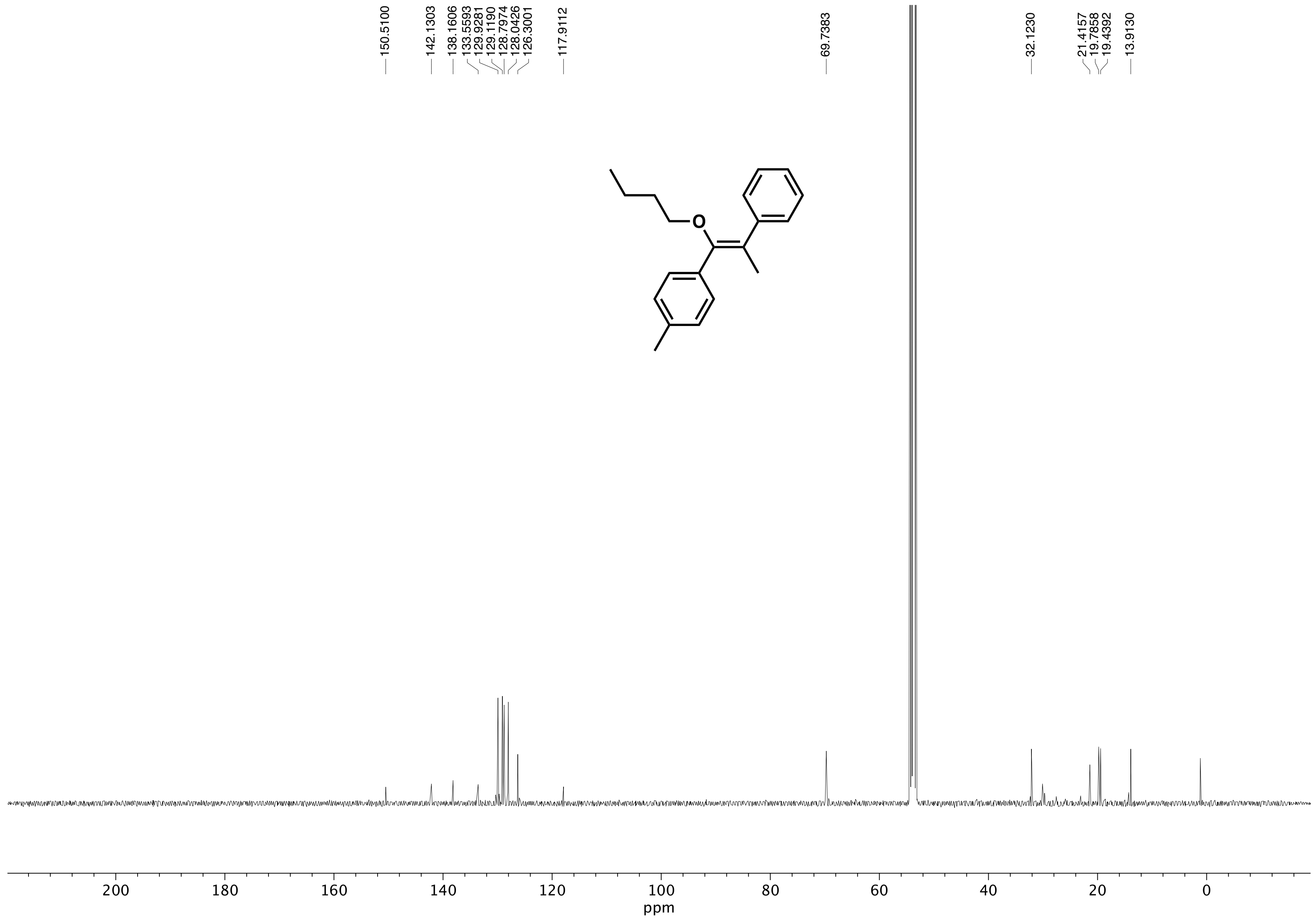

^{13}C NMR (101 MHz, d_2-DCM) of **SI-41.**

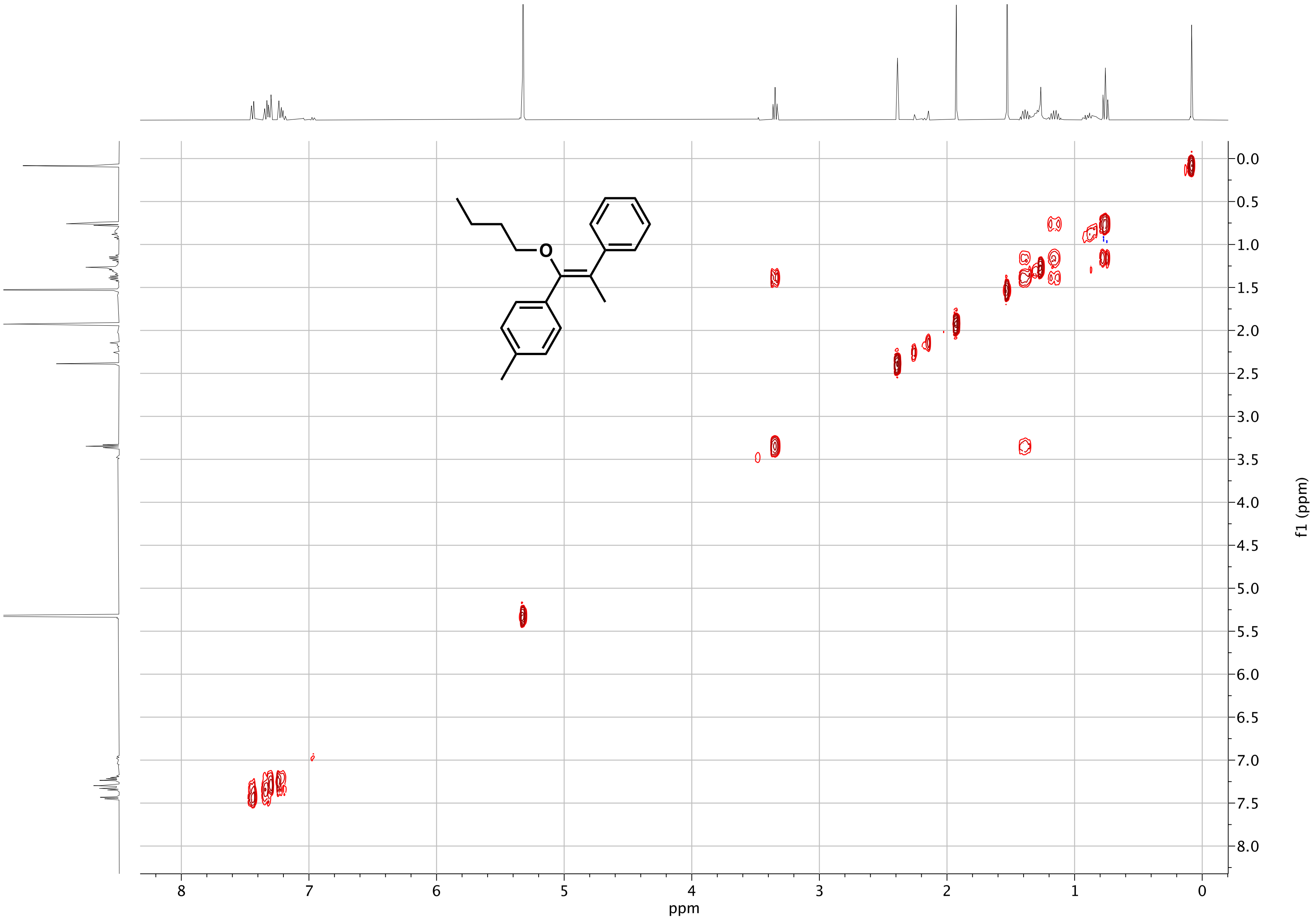

COSY NMR (400 MHz, d_2-DCM) of **SI-41**.

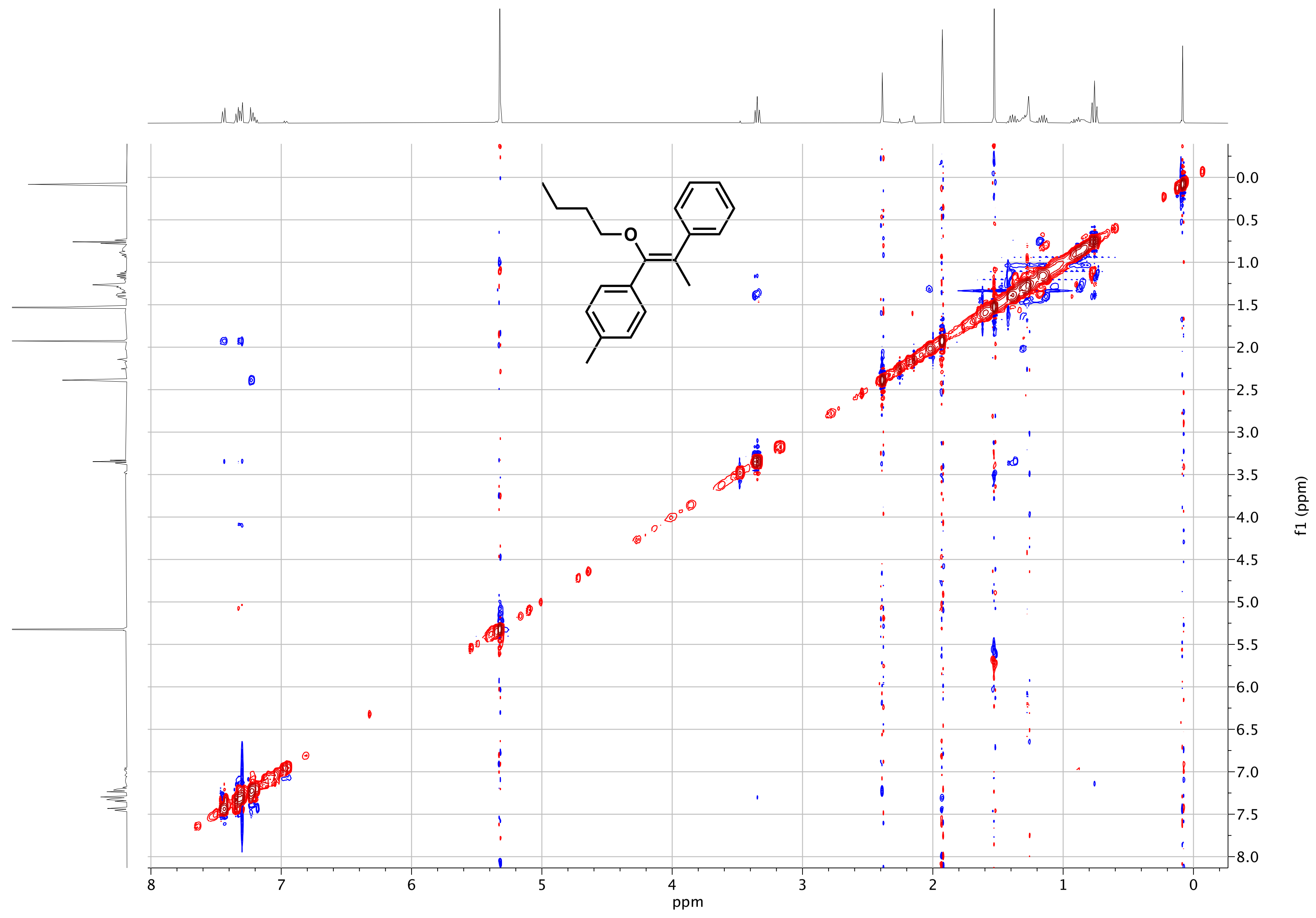

NOESY NMR (400 MHz, d_2-DCM) of **SI-41**.

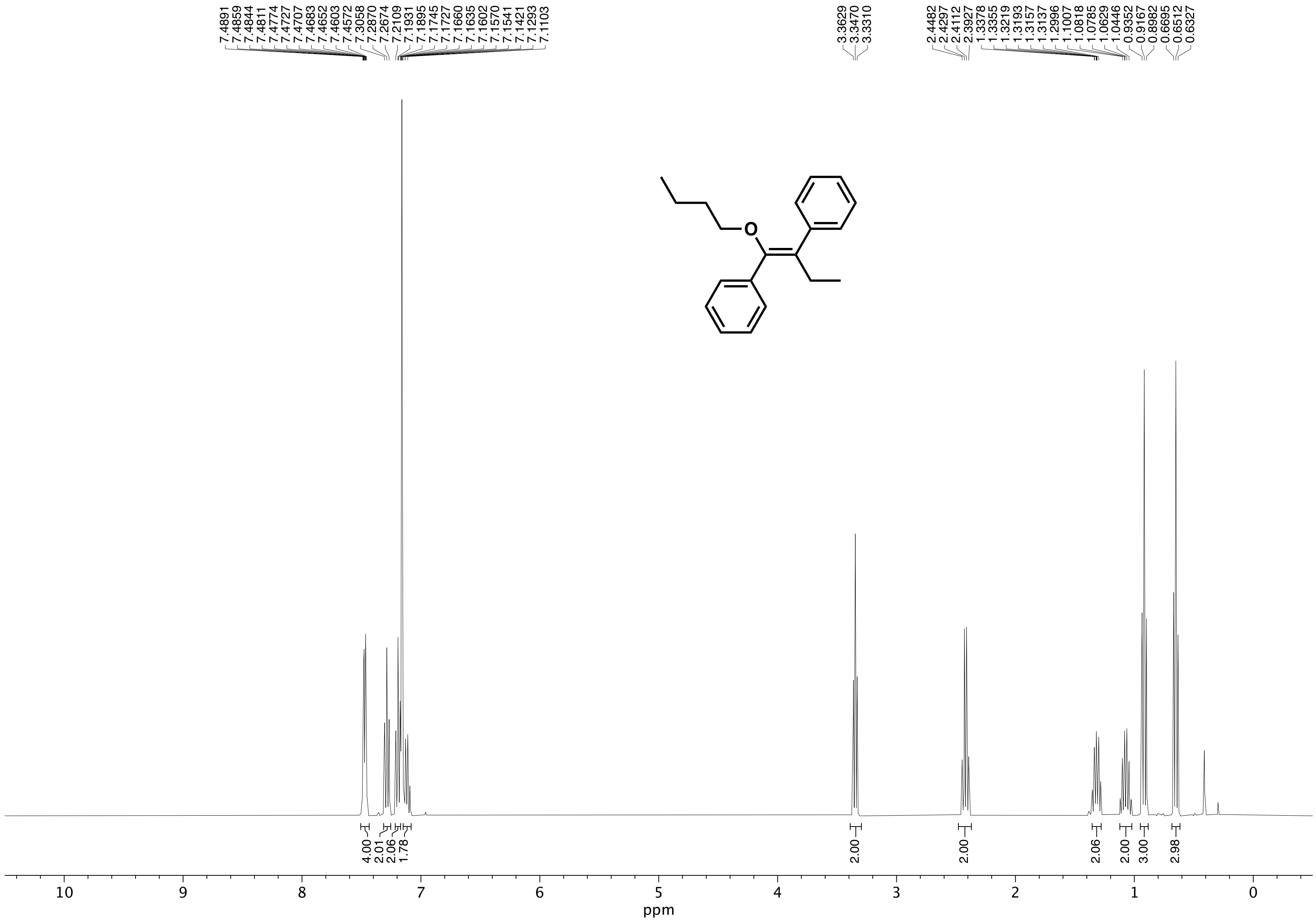

^{1}H NMR (400 MHz, C_6D_6) of **SI-42.**

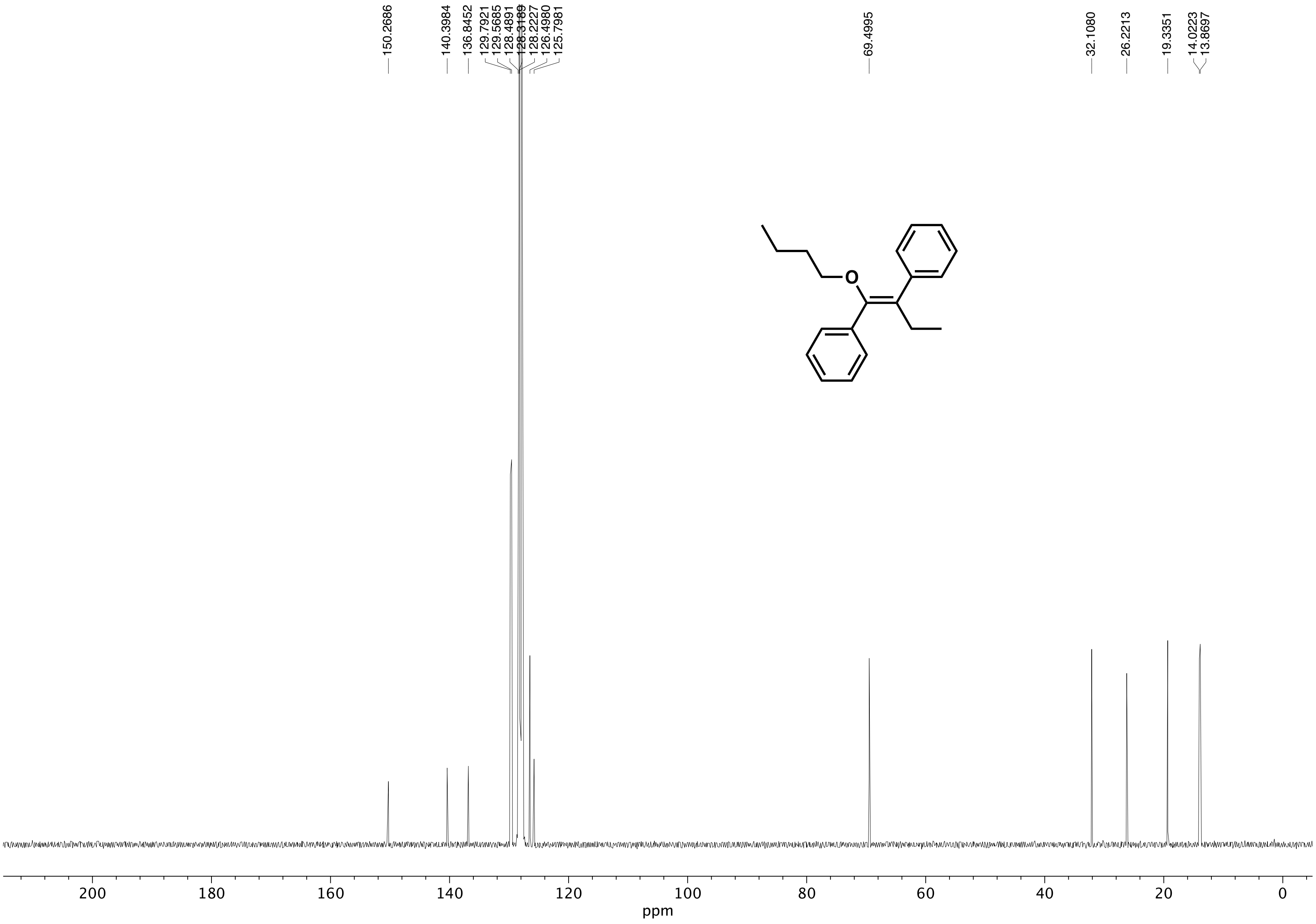

^{13}C NMR (101 MHz, C_6D_6) of **SI-42**.

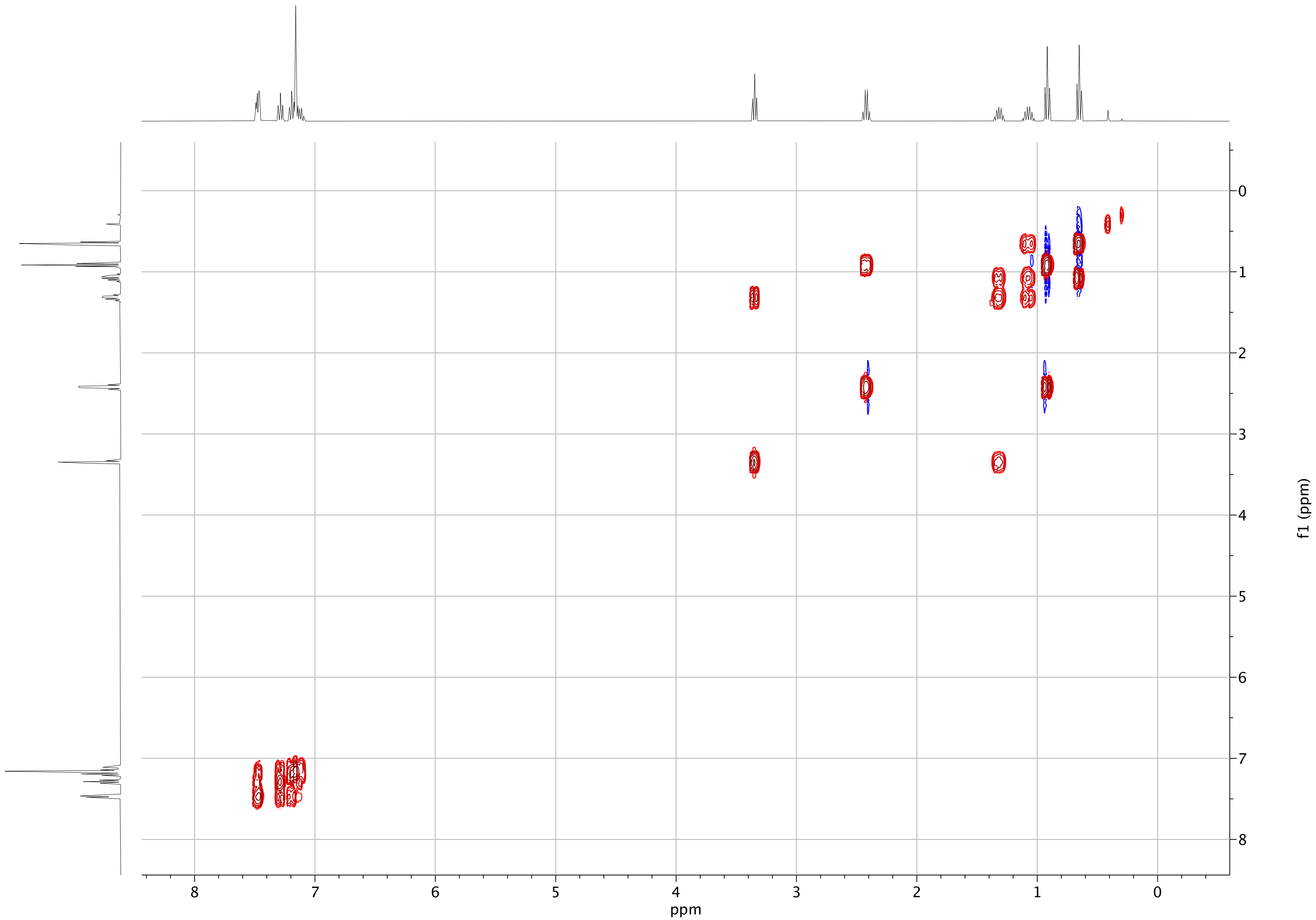

COSY NMR (400 MHz, C_6D_6) of **SI-42**.

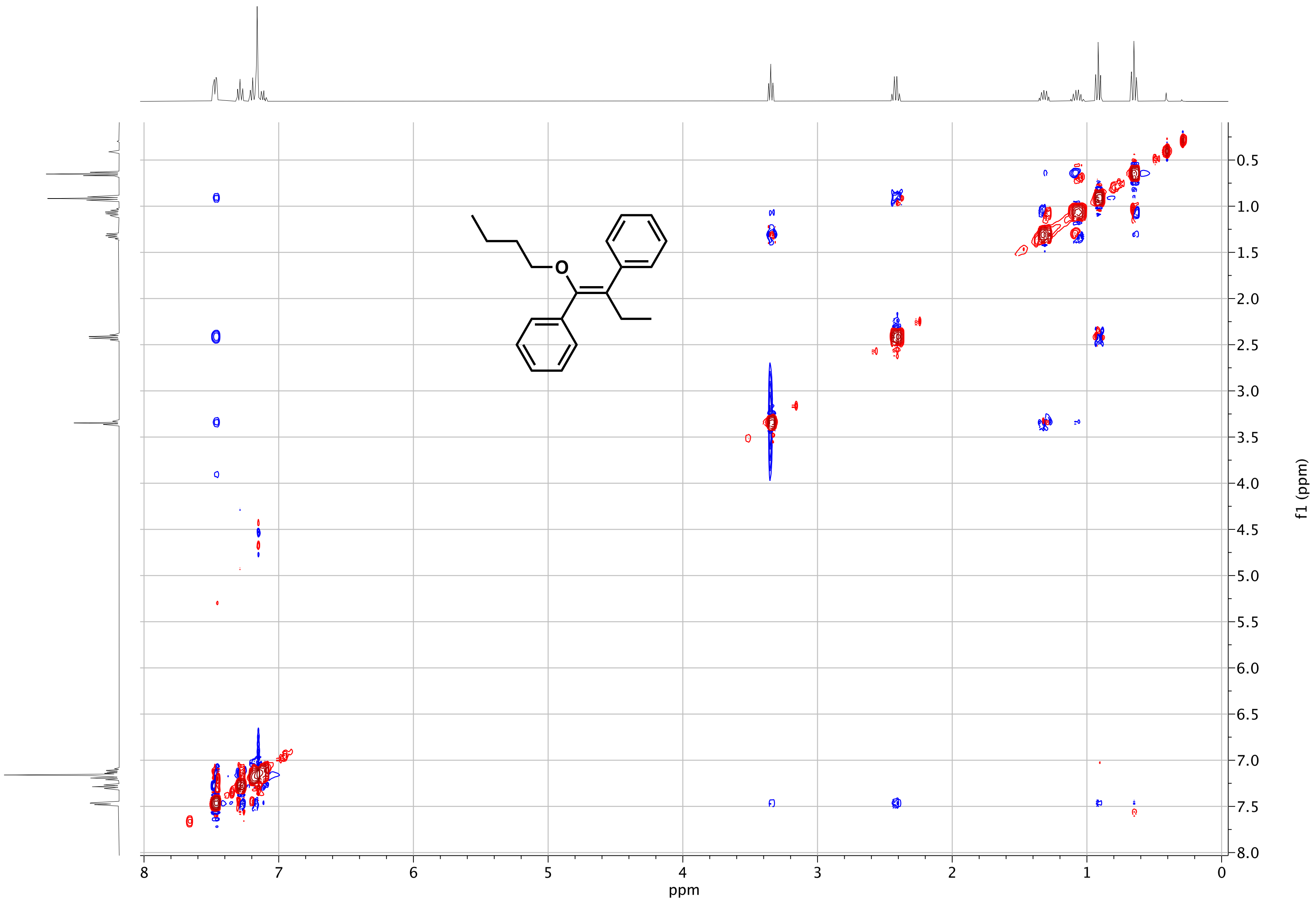

NOESY NMR (400 MHz, C_6D_6) of **SI-42**.

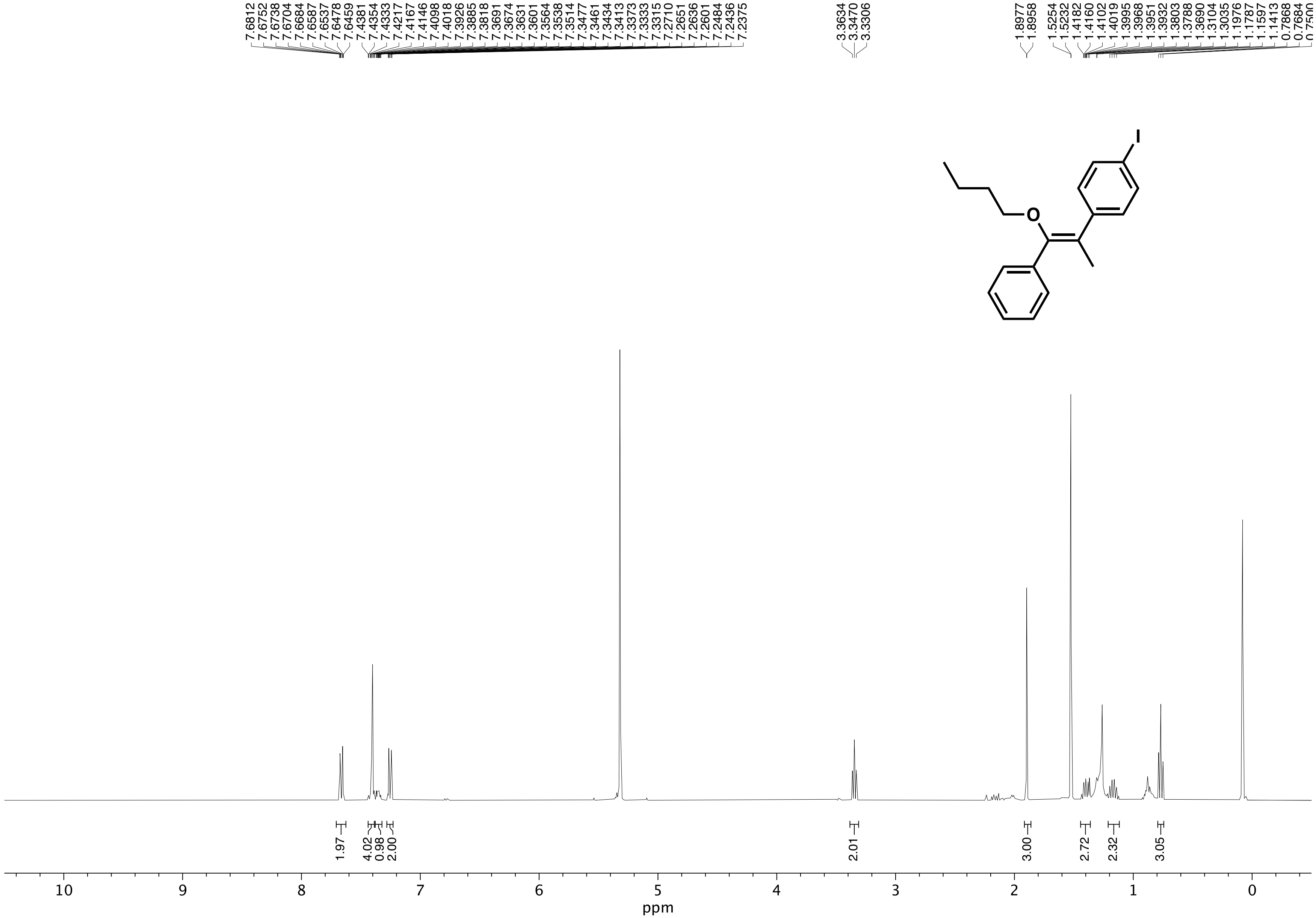

^{1}H NMR (400 MHz, d_2-DCM) of **SI-43**.

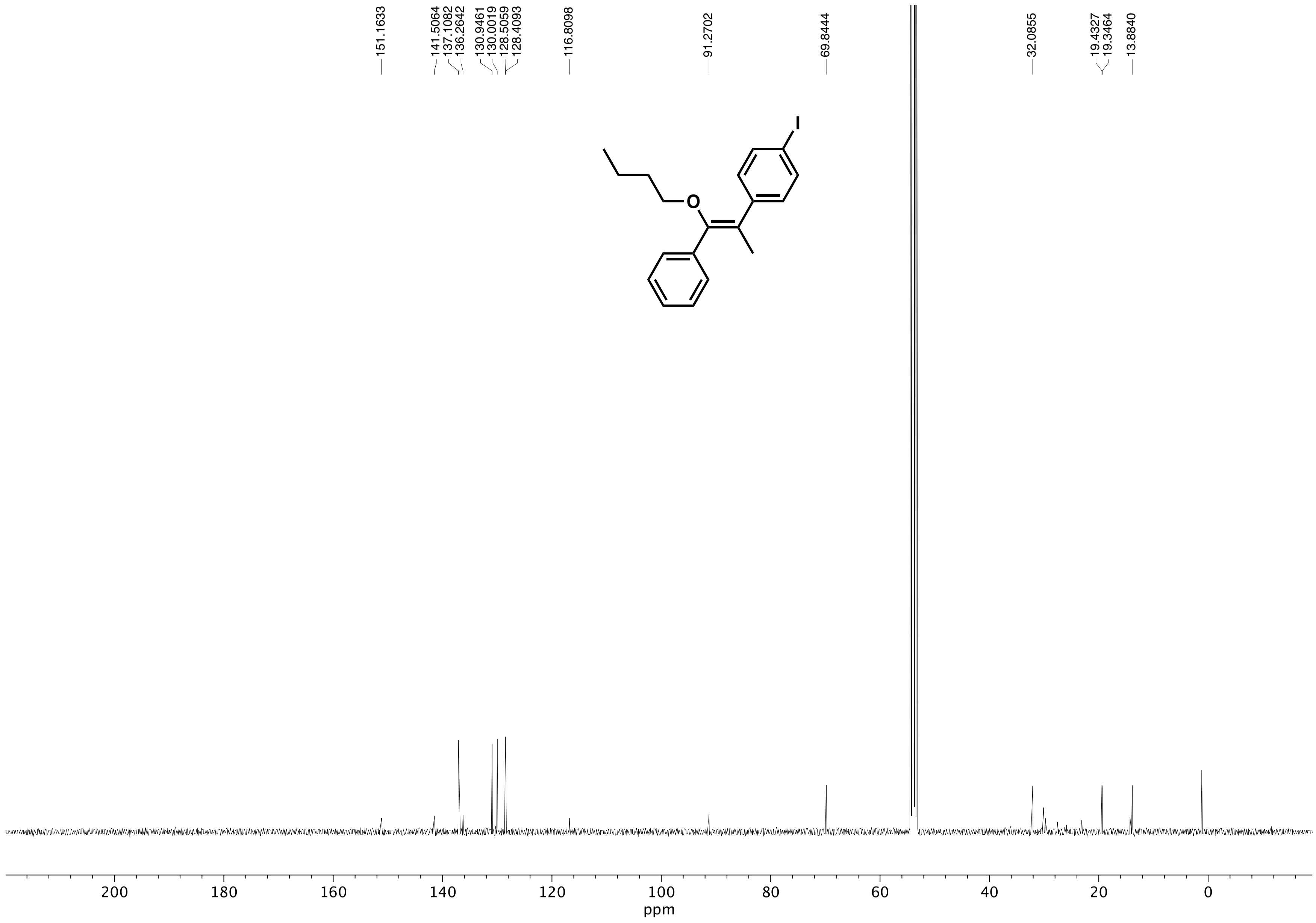

^{13}C NMR (101 MHz, d_2-DCM) of **SI-43**.

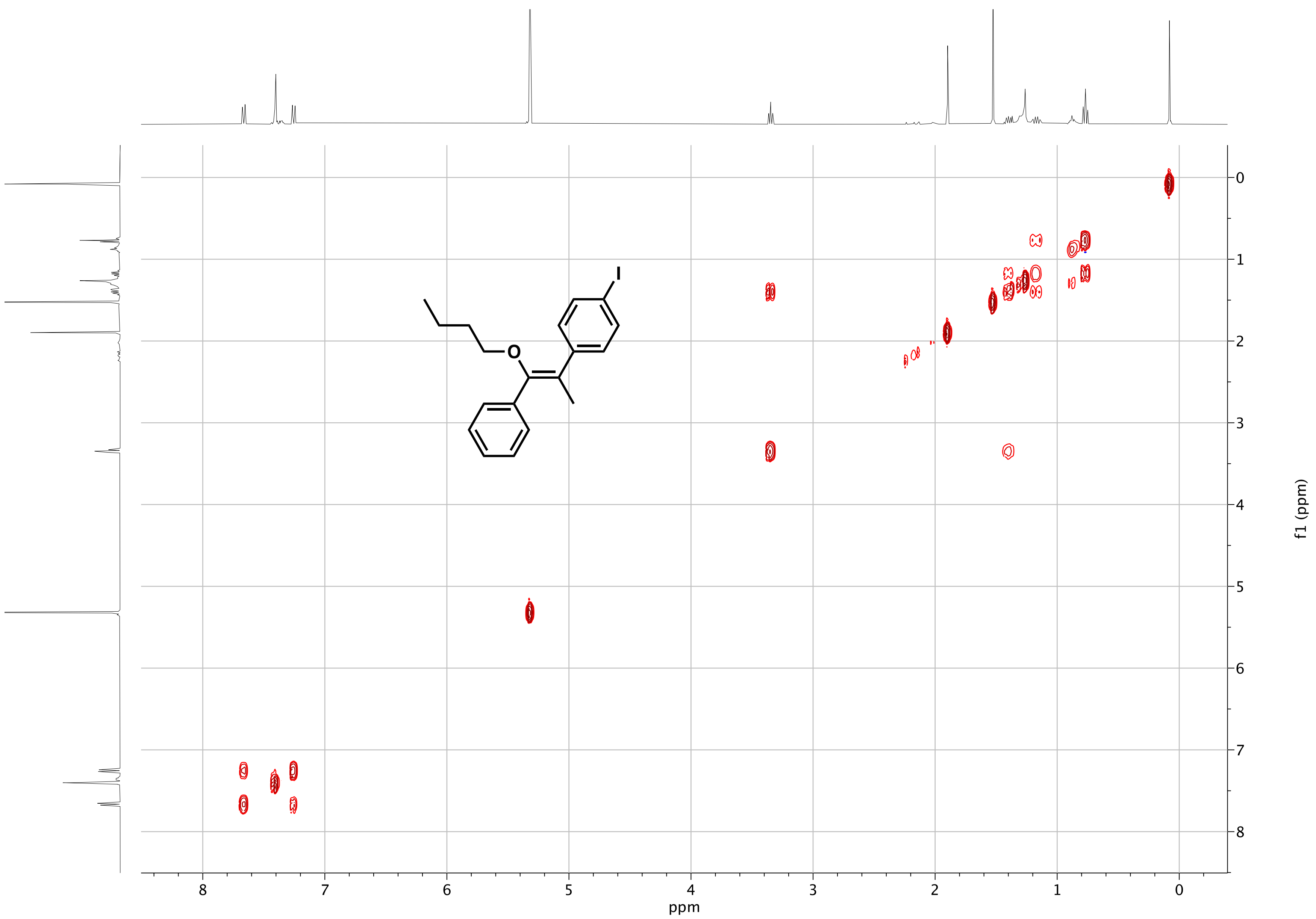

COSY NMR (400 MHz, d_2-DCM) of **SI-43**.

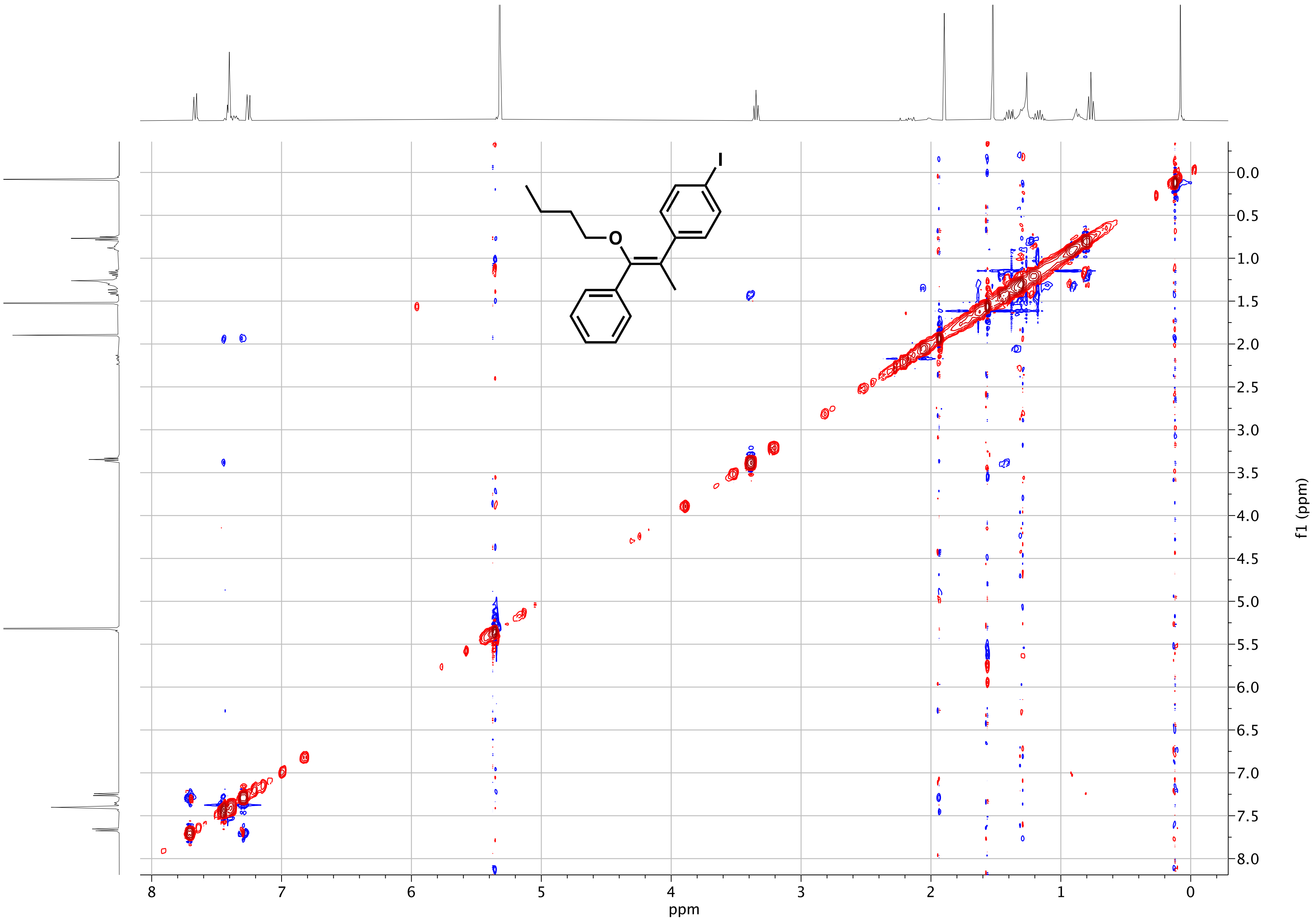

NOESY NMR (400 MHz d$_2$-DCM) of **SI-43.**

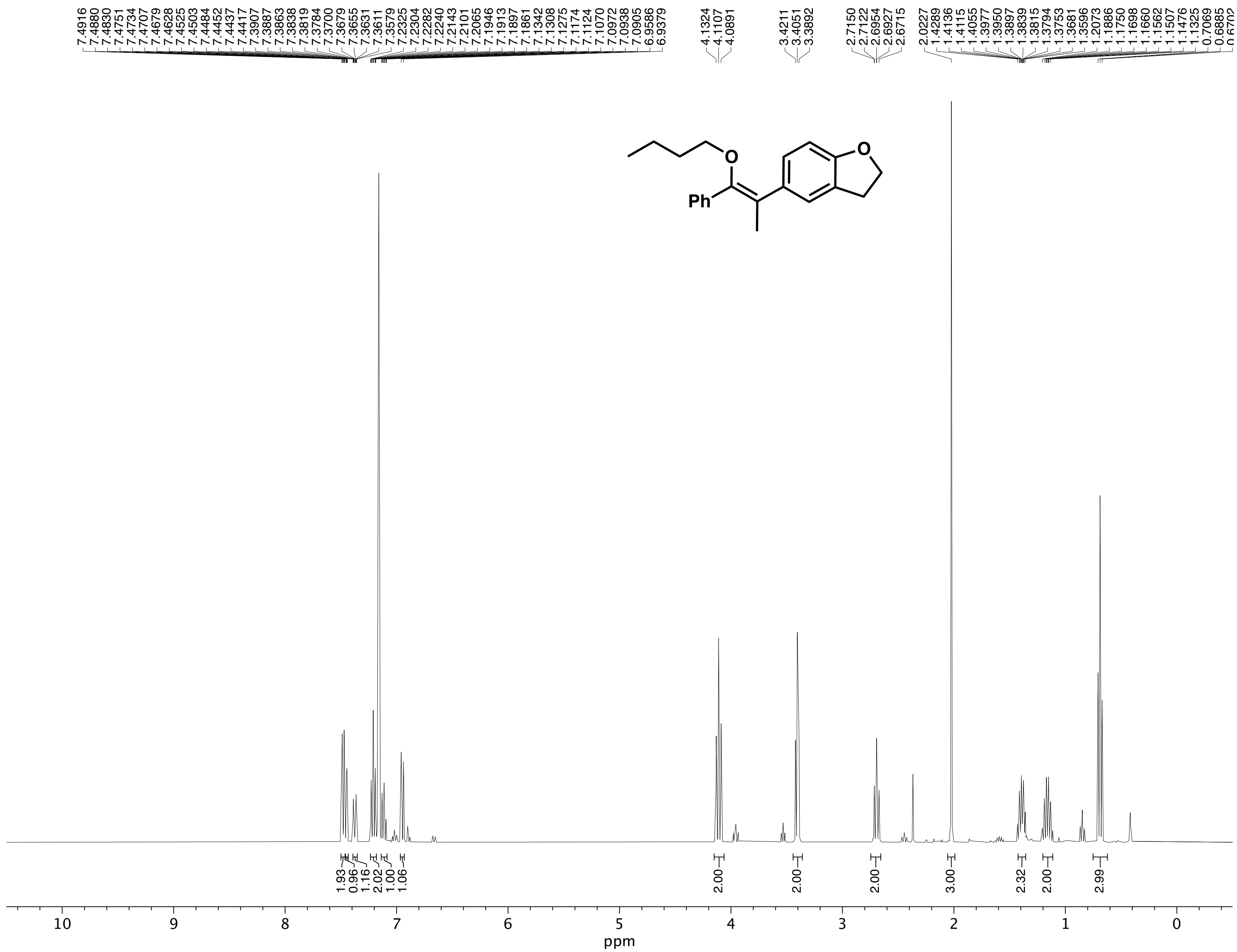

^1H NMR (400 MHz, C_6D_6) of compound **SI-44**.

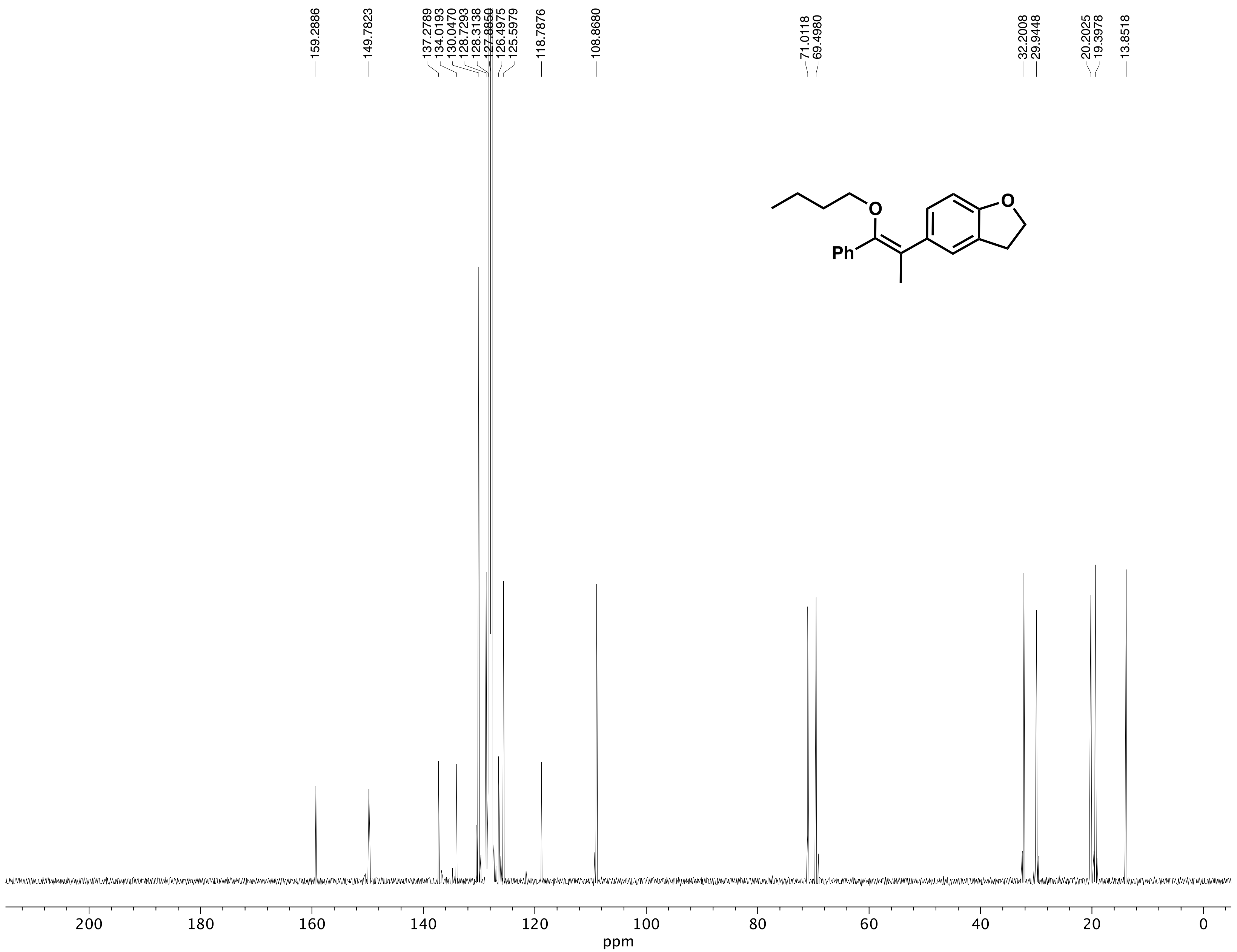

^{13}C NMR (101 MHz, , C_6D_6) of compound **SI-44**.

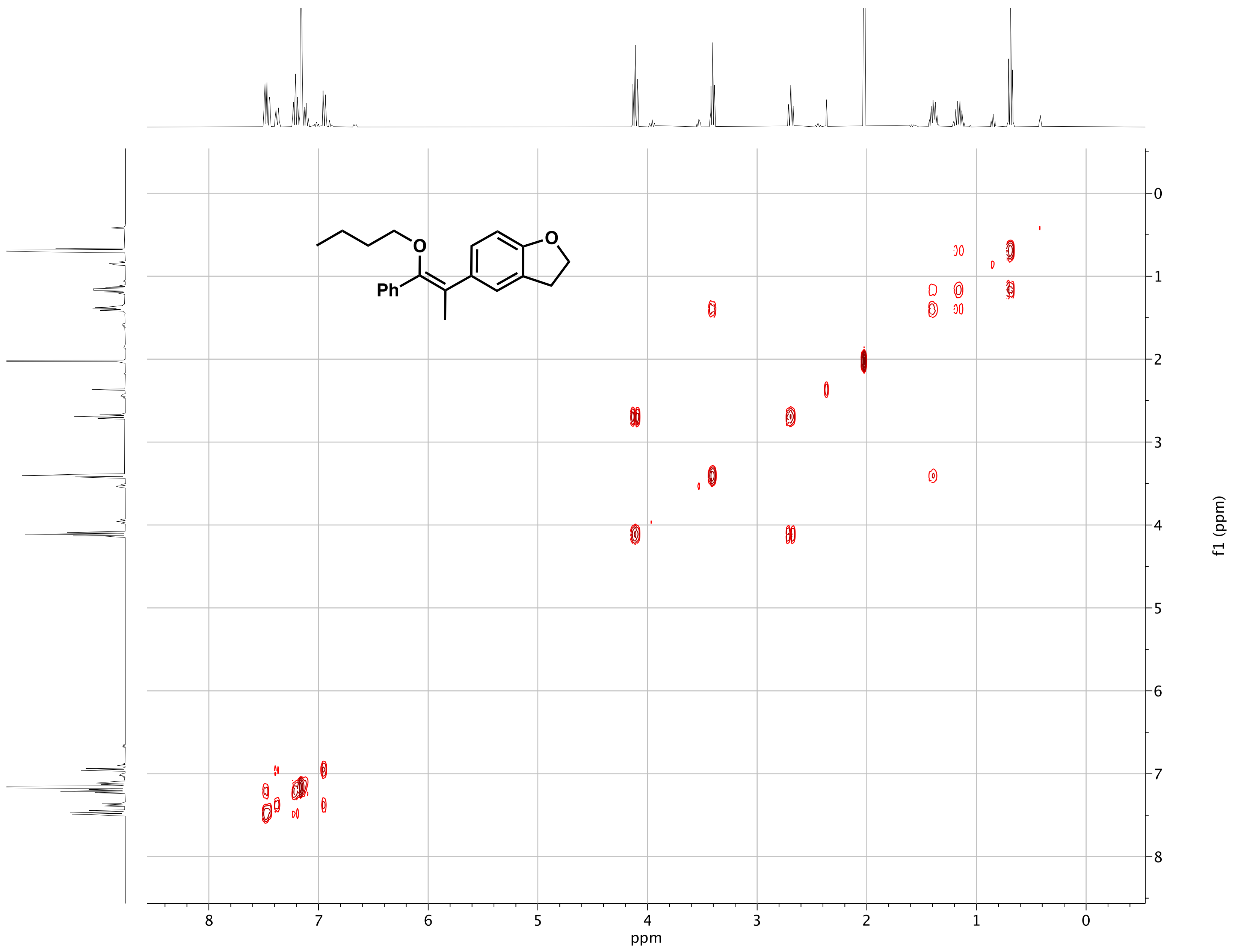

COSY (400 MHz, C_6D_6) of compound **SI-44.**

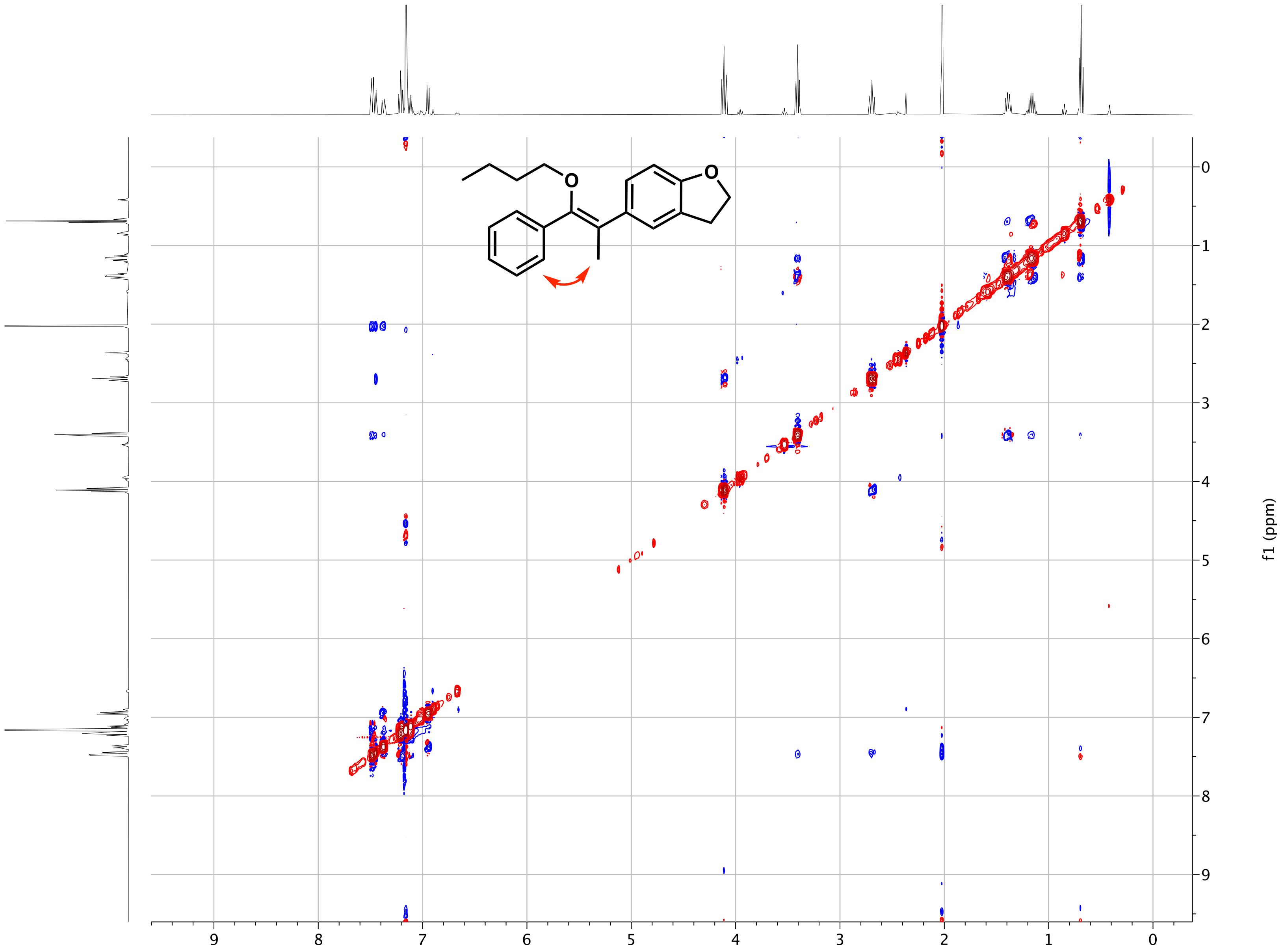

NOSEY (400 MHz, C_6D_6) of compound **SI-44.**
